# These children can become independent thanks to artificial intelligence! – A qualitative analysis of pre‑service teachers’ views on the use of artificial intelligence in special education

**DOI:** 10.1371/journal.pone.0355713

**Published:** 2026-08-28

**Authors:** Hale Cotuk Yucel, Ali Kaya, Merve Arslan

**Affiliations:** Department of Special Education, Nevsehir Haci Bektas Veli University, Nevsehir, Türkiye; Lucian Blaga University of Sibiu: Universitatea Lucian Blaga din Sibiu, ROMANIA

## Abstract

The integration of artificial intelligence (AI) into educational settings has accelerated in recent years, with growing implications for special education. However, the perceptions and evaluations of pre-service special education teachers, who will deliver AI-supported instruction in the near future, remain insufficiently examined. Objective. This study aimed to interpret the meanings that prospective special education teachers attribute to the use of AI in special education and to articulate the structures of significance through which those meanings are organized in their accounts. Methods. Employing an interpretive phenomenological design, we conducted semi-structured individual interviews with 14 pre-service teachers who had previously participated in artificial intelligence-based courses and projects. Content analysis of the data yielded four themes: (a) Teacher, (b) Child and Family, (c) Education, and (d) Artificial Intelligence and Future. Results. Findings indicate that participants conceptualized AI not merely as a tool perceived to individualize instructional processes, but also as a potential agent perceived to transform pedagogical roles. Participants articulated the view that AI could support critical domains for learners with special needs, such as independent living skills, social interaction, and decision-making, while also questioning risks such as social isolation, data privacy, dependency, and ethical uncertainty. Conclusion. The participants’ accounts collectively suggest a need for ethics-driven AI literacy in special education that foregrounds critical thinking and person-centered support.

## Introduction

### Artificial intelligence (AI)

Artificial intelligence (AI) is a field developed to enable intelligent machines and computers to understand human intelligence and perform analogous tasks [1]. For many scholars, AI has gone beyond computer science, emerging as a discipline that draws attention across philosophy, psychology, and education [2]. By modeling human processes of perception, learning, reasoning, and action, AI advances and reconfigures these processes in distinctive ways [3]. AI is also described as a technological capability through which machines can interact with humans, carry out tasks, and communicate in everyday life [4]. This technology provides opportunities to enhance quality of life in diverse domains, such as advanced medical diagnostics, new scientific discoveries, weather forecasting, and safe‑driving technologies [5]. Accordingly, AI can be defined as a technology by which computers or computer‑controlled machines perform human‑like mental processes, reasoning, inference, generalization, and learning from past experience [6]. In recent years, AI has become a central and popular concept in scientific and technological research [7]. Its use across economics, education, health, engineering, statistics, agriculture, security, and transportation is said to reshape society and enable a new era [8,9]. Although AI’s impact is increasingly visible today, it is especially prominent in education, where it supports teaching and learning processes [10]. Indeed, the effects of AI‑mediated technologies on teaching and learning have become a major focus of scholarly inquiry [11]. Research and applications of AI in education focus on cognition, learning, teaching, and student development, supporting talent cultivation and instruction in the age of AI [12]. In this regard, AI contributes to education by training individuals, building their skills, increasing their performance, and enabling them to be more successful in their tasks [13]. Consequently, AI‑enabled support in education is regarded as a revolutionary shift [14].

### Education and AI

Integrating technologies into teaching and learning processes enhances student experiences by promoting meaningful and effective use [15]. Students who are supported with technology in schools are observed to demonstrate greater interest in learning [16]. AI technologies, in particular, provide learners with diverse learning experiences and thus play an important role in modern education [17]. In education, AI can guide collaborative learning processes for students while easing teachers’ administrative workloads, developing curriculum outlines, preparing lesson plans, creating assessment materials, and grading, thereby allowing teachers to devote more time to learners [18,19]. Recent years have witnessed notable advances in AI for education [20]. The integration of AI and education can significantly improve the quality of teaching and learning [21]. AI‑supported education stands out for its adaptability and personalization, enabling students to learn and develop skills aligned with their needs and experiences [22]. Alongside the advantages, however, AI applications raise ethical risks and concerns related to personal data and learner autonomy [23]. For example, issues in student assessment and support for metacognitive processes may arise in terms of ethics, transparency, fairness, or accountability [24]. Despite such concerns, AI is being used effectively across educational institutions at all levels [25].

In early childhood, AI technologies are important in fostering children’s creativity and imagination [26]. With well‑designed technological tools, children can explore and effectively use AI from an early age [27]. For instance, PopBots was developed to enable children to learn about AI by creating, programming, training, and interacting with a social robot [28]. The literature emphasizes the importance of developing educational programs to foster AI awareness starting in early childhood [29]. Beyond contributing to understanding AI, machine learning, computer science, and robotics, AI‑enabled activities can play a significant role in creativity and emotion regulation [30]. Notably, when interacting with AI‑enabled robotic toys, children are said to strengthen problem‑solving skills by questioning issues robots may encounter and generating solutions [31]. Classroom‑based AI‑supported robotics applications also promote children’s cognitive skills and social interaction [32]. With the development of age‑appropriate teaching tools in basic education classrooms, AI support has become more widespread [33]. Robots used in elementary education have been observed to increase students’ interest in instruction [34]. Similarly, integrating AI into secondary education has been found to support students’ needs and enhance learning [35]. There are also studies on higher education students and AI tools. For example, an instructional program was designed for university students on AI use, with embedded practical applications [36]. Another study emphasized that AI tools positively affect students’ academic performance and that educators’ competence with AI tools is important [37].

Finally, research into teachers’ views of AI shows that they hold positive attitudes toward AI education but have limited skills in this area [38]. Because teacher competence in technology use is necessary to support students’ learning and engagement, it is critical that teachers receive the requisite training and support [39]. Teachers need to learn how to use AI tools effectively to improve their students’ academic achievement [40]. Accordingly, integrating awareness of AI tools and their effective use into teacher preparation is important [41].

### Special education and AI

While AI is radically transforming people’s lives, it provides robust support for the education of students with special needs [42]. When traditional approaches fall short in educating children who require special education, AI offers solutions tailored to individual needs [43]. By enabling early identification and intervention for learning difficulties and developmental disorders, AI represents a major advance for special education [44]. Once identified via AI tools, appropriate intervention strategies can be developed [45]. In education, AI support can produce various materials—such as text and video—that facilitate access for students with disabilities, transforming education by providing equitable learning opportunities [46]. AI applications can make the daily lives of individuals with special needs easier [47]. AI is also emphasized as important in supporting interactions and learning processes for students with special needs [48]. For example, AI‑supported applications are being developed to promote independent living skills for individuals with visual impairments [49]. Through AI support, individuals with visual impairments meet needs across diverse domains, reading text, walking on the street, engaging in artistic activities, thus improving quality of life [50]. An AI system designed for people with visual impairments enables independent mobility through image recognition, collision detection, and obstacle detection [51]. For individuals with hearing loss, AI‑supported hearing aids offer innovative solutions that can improve quality of life [52]. AI can also contribute by diagnosing hearing loss at an early age and providing individualized interventions [53].

Beginning in early childhood, AI‑enabled smart robots contribute substantially to the education of children with social interaction challenges such as autism spectrum disorder (ASD) [54]. However, when using robots in the education of children with ASD, it is crucial to personalize applications according to each child’s needs [55]. With AI technologies, special education teachers can calibrate the difficulty level of learning content and provide support for individualized instruction that meets students’ needs [56]. Researchers report that in the education of children with ASD, smart robots are widely used in social interaction, communication, and play and are regarded as valuable tools [55]. AI can ease special education teachers’ workload, enabling them to focus on students’ strengths [57]. Accordingly, teacher preparation programs emphasize using AI as a supportive tool [58]. To increase pre‑service teachers’ willingness to use AI in education, it is important to provide the necessary training on effective use of AI tools [59,60]. In this respect, AI offers innovative and personalized learning experiences that align with student needs, increase engagement, and support effective teaching–learning processes in special education [61].

In sum, developments in AI have significant repercussions in special education, as in many other fields [24]. Beyond the existing body of AI research, the literature underscores the need for more studies not only on learning and classroom management but also on teacher education and school leadership [62]. A review of current research shows that it is important to identify pre‑service special education teachers’ views on AI concepts, applications, roles in special education, and future trajectories. Within this context, the phenomenon addressed in the present study is not AI use as a technical application alone, but the meanings, expectations, concerns, and future-oriented evaluations that pre-service special education teachers construct around AI in special education. This context is especially relevant because these teacher candidates are preparing for a professional field in which individualized instruction, accessibility, family support, ethical decision-making, and the promotion of independence for children with special needs are central concerns. Examining their views can therefore help identify the conceptual, ethical, and practical support needed to increase the effective use of AI in special education. The current study aims to interpret the meanings that pre-service special education teachers attribute to the use of artificial intelligence (AI) in special education, and to render explicit the implicit structures of significance through which those meanings are organized.

## Method

### Design

This study employed an interpretive phenomenological qualitative research design to explore how pre-service special education teachers make sense of AI in special education. This design was appropriate because it enabled the researchers to examine the meanings that participants attributed to the phenomenon and the ways in which these meanings were organized in their accounts [63,64].

### Participants

The study employed criterion-based purposive sampling to recruit participants who were directly relevant to the research aim. This sampling strategy is used in qualitative research to identify information-rich cases that meet predefined criteria [63]. The inclusion criteria were: (a) being enrolled in an undergraduate special education teacher education program; (b) having taken a technology course in special education for one semester; and (c) having completed a course assignment on special education and AI. All participants met these three inclusion criteria. The additional variables reported in Table 1 were collected only to describe the participant group and were not used as inclusion criteria. Interview durations in Table 1 are reported in minutes. The strategy is not intended to support statistical generalization; rather, it is intended to support analytic transferability in the sense developed by Lincoln and Guba [65], in which the reader is offered sufficient contextual detail to judge for themselves the relevance of the findings to other settings. The participant recruitment process took place between May 6 and May 13, 2024. Verbal consent was obtained from all participants, and audio recordings documenting these consents were retained. Participants were coded as Participant P01, P02, …, P14. All demographic information is presented in Table 1.

**Table 1:** Descriptive data of the participants.

Code	Demographic information	General information				Interview Duration
	Gender	Works with a child with disabilities?	Has a relative with special needs?	Uses AI?	Follows current AI-in-SE studies?	
P01	Male	No	No	Yes	No	24
P02	Female	No	Yes	Yes	Yes	42
P03	Female	Yes	No	Yes	No	36
P04	Female	No	No	Yes	No	54
P05	Male	Yes	No	Yes	No	31
P06	Male	No	No	Yes	Yes	28
P07	Female	No	Yes	Yes	Yes	46
P08	Male	Yes	Yes	Yes	Yes	50
P09	Female	No	Yes	Yes	No	34
P10	Female	No	No	Yes	Yes	86
P11	Female	Yes	No	Yes	Yes	25
P12	Female	Yes	Yes	Yes	Yes	40
P13	Male	Yes	No	Yes	No	18
P14	Male	No	No	Yes	No	12

### Reflexivity

Reflexivity, that is, the researcher’s active engagement with the ways in which their own social position, prior experience, and professional commitments may shape data generation and interpretation, is a central quality consideration in qualitative inquiry [63,64,66]. We provide here a brief account of the team’s positionality and of the steps taken to mitigate researcher influence on the data and on the analysis.

All three authors are members of a special education department at a Turkish public university. The first author holds a doctorate in special education, has carried out several studies focused on children with special needs, and taught the technology-in-special-education course that the participants had completed prior to the interviews. The second author, who conducted all interviews, holds a doctorate in special education and brings twelve years of pre-academic field experience working with children with special needs and their families. The third author graduated from the undergraduate special education program at the same university and is at the time of writing a master’s student in autism spectrum disorders.

Three features of our positionality required ongoing reflexive attention. First, our shared professional commitment to the field of special education predisposes us to view technological developments through the lens of their possible contributions to children with disabilities and their families and could in principle have biased the analysis toward a benefits-oriented reading. Second, the first author’s prior instructional relationship with the participants, although it provided the contextual grounding for the technology-in-special-education course referenced in the Procedure, also created a potential source of social desirability in the participants’ accounts. Third, the team’s location within a Turkish national and institutional setting shapes the cultural assumptions through which we read participants’ statements about technology, the state, and the future of education.

Several steps were taken to mitigate the influence of these positionalities. The interviews were conducted by the second author, who had not been the participants’ course instructor, and the verbal-consent script explicitly stated that participation was independent of any course-related evaluation. Open and non-leading question formulations were used in the interview protocol, with follow-up probes invited only after the participant had completed their initial response. Analytic memos were kept throughout the coding process, and the developing code structure was reviewed across team members so that no single author’s interpretation dominated. The inter-coder reliability procedure (described in the Coding Reliability subsection below) included an external doctorate-level expert who was not part of the research team, providing a further check against in-team interpretive drift. We also note, as a transparent limitation, that our reflexive efforts cannot eliminate the cultural and institutional embeddedness of our reading, and we return to this point in the Limitations.

### Procedure

To elicit up‑to‑date views prior to the study, each participant prepared a project on special education and AI and completed a semester‑long course on technology in special education. As seen in the demographic data, the mean participant age was 23; accordingly, participants belong to a generation that uses technology intensively in daily life. At the end of the course and project process, individual interviews were conducted using semi‑structured questions. For the interview protocol, expert feedback was obtained from three scholars with doctorates in special education and one scholar with a doctorate in information technologies. Following a pilot interview, all interviews were scheduled and conducted face to face with participant consent. Interview durations are summarized in Table 1. The shortest interview lasted 12 minutes and the longest 86 minutes, with an average of approximately 37 minutes. The full semi-structured interview protocol, including the lead questions and the follow-up probes used to elicit deeper accounts during the interviews, is provided in Turkish original and English translation as S2 File.

### Ethics

The study was reviewed and approved by the Scientific Research and Publication Ethics Committee of Nevsehir Hacı Bektaş Veli University (Decision No. 2024.05.100; Date: 30 April 2024). Given the low-risk, interview-only, non-clinical nature of the study and in order to minimize procedural burden on participants, the Ethics Committee specifically approved the use of audio-recorded verbal informed consent in lieu of written consent, as well as the open public deposition of de-identified interview transcripts as supplementary material conditional on participants’ explicit, on-record consent.

The verbal-consent procedure was operationalized as follows. Prior to each interview, the first author read aloud — verbatim — a standardized Participant Information and Consent Script (provided in Turkish original and English translation as S1 File) describing (i) the aims of the study, (ii) the voluntary and withdrawable nature of participation, (iii) the right to skip any question or terminate the interview without justification, (iv) the audio-recording protocol, (v) the participant-coding and anonymization procedure, (vi) the data-storage and -retention plan, and (vii) the intended open deposition of de-identified transcripts as supplementary material. Participants were then asked, on record, whether they (a) had understood the information provided and (b) consented to participate, to be audio-recorded, and to the open deposition of their de-identified transcript. Each participant’s affirmative verbal response, captured as the opening segment of the interview audio file, constituted the documented record of consent; this audio-recorded affirmation, made by the participant in their own voice, served as the sole and primary form of consent documentation, in accordance with the Committee-approved protocol. No separate witness signature or witness log was used, as the audio recording itself was deemed by the Committee to provide adequate, durable, and verifiable documentation of the consent transaction.

The voluntary, withdrawable nature of participation was reiterated at the start of each interview. Participants were informed that withdrawal at any subsequent stage would result in the destruction of their interview data and the removal of the corresponding transcript from any supplementary deposition. The verbal-consent audio segments are stored separately from the substantive interview data on a password-protected institutional drive accessible only to the research team and will be retained for five years following study completion, in accordance with the institutional ethics protocol.

### Data analysis

We analyzed the interview material using qualitative content analysis, which is a method for drawing replicable and valid inferences from textual data [67] and in which codes, categories, and themes are generated through the researcher’s iterative close reading of the corpus [63,68]. The analysis combined the conventional approach (in which initial codes are derived inductively from the text) with the summative approach (in which frequencies of coded segments are used to index salience), as described by Hsieh and Shannon [69].

The procedure unfolded in six sequential steps, drawing on Saldaña’s [70] two-cycle coding framework.

First, all 14 audio recordings were transcribed verbatim into Turkish, producing a corpus of approximately 92 single-spaced pages across interviews of an average duration of 37 minutes. Each transcript was checked against the audio recording by a second member of the research team to ensure transcription fidelity.

Second, the first author conducted an initial close reading of all transcripts to gain a holistic sense of the corpus, recording analytic memos on emerging patterns without yet assigning codes.

Third, first-cycle coding was undertaken using descriptive and in-vivo coding [70], in which meaningful units of participants’ speech were highlighted in Microsoft Word and assigned short descriptive labels that stayed close to the participants’ own terms. This first-cycle pass produced an initial pool of 254 codes across the 14 transcripts.

Fourth, the initial codes were consolidated through a process of comparison and merging: codes referring to the same underlying meaning were grouped, near-synonyms were aligned, and codes that lacked sufficient grounding in the data (appearing only once and without a clear conceptual anchor) were flagged for review. This consolidation reduced the initial pool to a working set of approximately 150 codes.

Fifth, second-cycle coding [70] grouped semantically related codes into categories. Categories were defined inductively from the data rather than imposed a priori, and each category was given a working definition and a representative exemplar quote. This step produced 45 categories.

Sixth, the 45 categories were organized into four overarching themes (Teacher, Child and Family, Education, and Artificial Intelligence and Future) on the basis of shared conceptual concerns. Theme labels were generated inductively, and the final theme structure was cross-checked against the original transcripts to ensure that no substantive content was lost in the move from codes to themes.

Throughout the procedure, analytic decisions were recorded in a running memo log, and the developing code-category-theme structure was iteratively cross-checked against the transcripts. The full codebook, with theme definitions, category descriptions, code labels, and exemplar quotes, is provided as S3 File. Frequency counts are reported alongside themes, categories, and codes throughout the Findings. Their analytic role follows the summative variant of qualitative content analysis as described by Hsieh and Shannon [69], in which counts of coded segments are used not as inferential statistics, and not as estimates of prevalence in the wider population of pre-service special education teachers, but as an internal-to-the-sample indicator of the salience of each theme and category in participants’ accounts. In this study, frequencies therefore answer the question, “where did the participants’ attention concentrate?”, and they function as a structural scaffold for the interpretive reading that follows. We did not conduct statistical tests on these counts, and we caution the reader against treating them as such. This dual role of qualitative coding, in which counts support but do not displace interpretation, is consistent with the broader qualitative-evaluation tradition [63].

### Anonymization for open deposition

Following coding and analysis, all interview transcripts were prepared for open public deposition (S4 File) through a multi-step anonymization procedure adapted from Saunders, Kitzinger, and Kitzinger [71] and Kaiser [72]. First, all direct personal identifiers — participants’ names, the names of family members, instructors, classmates, and supervising teachers, and the names of the schools and field-placement sites referenced by participants — were replaced with generic descriptors (e.g., “the participant,” “an instructor,” “a placement school”). Second, all institutional identifiers (department name, course code, university name where directly named) were generalized. Third, third-party references — including any specific child with a disability discussed by participants from their field-placement experience — were generalized to remove biographical detail that could enable identification. Fourth, the anonymized transcripts were cross-checked against Table 1 by a research-team member not involved in the original interviews to identify and remove any residual combinations of demographic and contextual cues that could enable indirect re-identification. The final deposited transcripts retain the substantive content of participants’ accounts while precluding identification of participants and third parties.

### Coding reliability

Confirmability was examined for validity and credibility. To ensure coding reliability, Miles and Huberman’s [73] formula (Reliability = Agreements/ Agreements + Disagreements) was applied, which suggests inter‑coder agreement should exceed 80%. In this study, an external expert with a doctorate in special education re‑coded transcripts from 7 randomly selected interviews. Comparison of codes yielded 220 agreements and 34 disagreements, resulting in a reliability of 85.45%. Disagreements were discussed; consensus was reached on 13 codes, and the remaining 21 codes were excluded from the study.

## Findings

**Fig 1:**
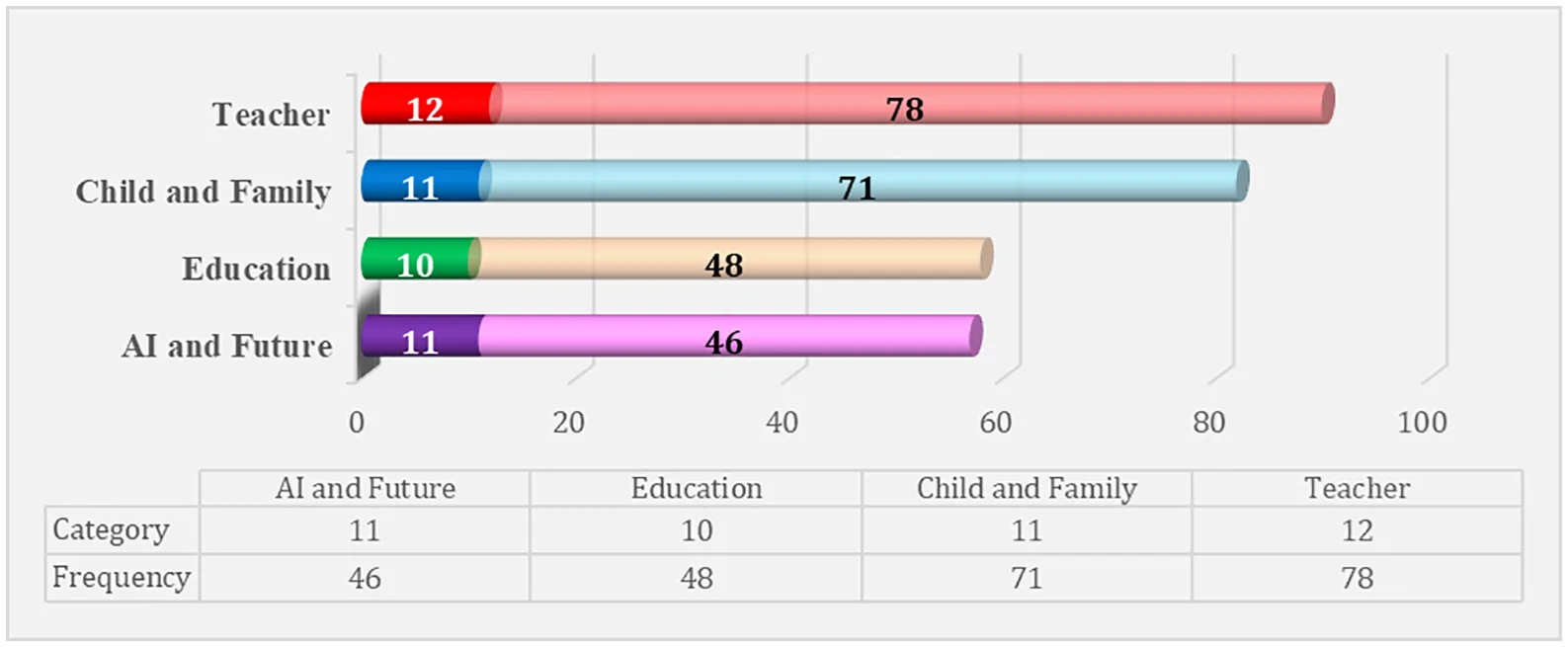
Themes, categories, and frequencies.

Analysis of responses to the semi‑structured interview questions yielded four themes. The themes, categories, and frequencies derived from content analysis are summarized in Fig 1. The full codebook including the four themes, their constituent categories, definitions, exemplar quotes, and frequency counts is provided as S3 File. The complete anonymized Turkish-language transcripts on which the analysis is based are available as S4 File.

As shown in Fig 1, four themes were generated from participants’ accounts: Teacher, Child and Family, Education, and Artificial Intelligence and Future. The Teacher theme produced the highest frequency of coded segments across participants, while the Artificial Intelligence and Future theme produced the lowest. These frequencies index the salience of each theme in participants’ accounts and are not intended as estimates of effect or prevalence in the wider pre-service teacher population.

### Teacher

**Fig 2:**
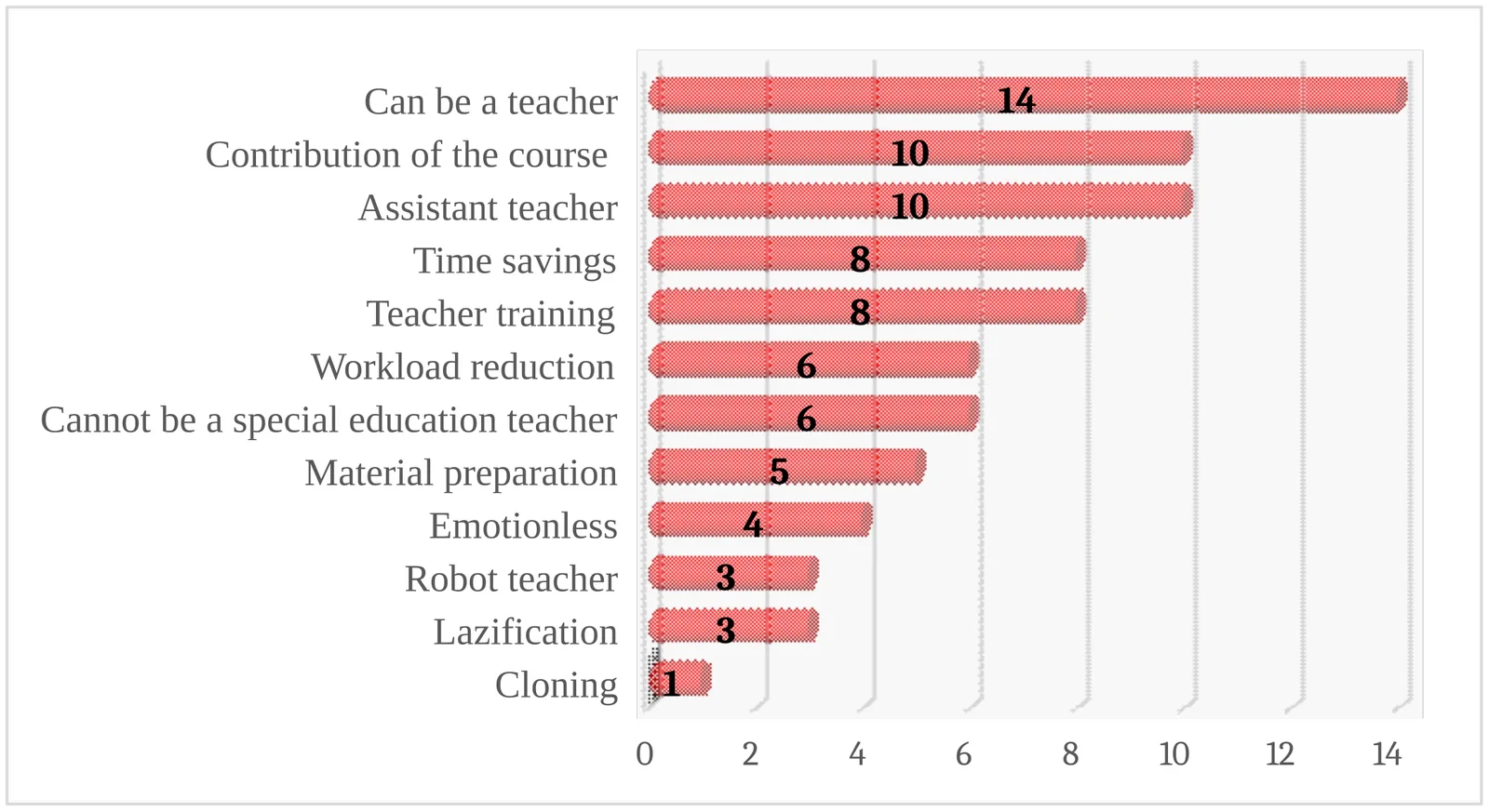
Distribution of categories and frequencies related to Teacher.

This theme focuses on participants’ perceptions of how AI may affect teachers in special education in the future; the categories and frequencies are depicted in Fig 2.

All participants in this study articulated the view that AI could come to occupy aspects of a teaching role in future years: “Once AI operates just like a human brain, I think it will replace us. It could happen; anything is possible” (P12). Participants also noted that AI could serve as a ‘shadow teacher’ in the near future: “If it becomes a shadow teacher in the future, it could support daily living skills—if it is always beside the child” (P04). They articulated the contribution of the course they took: “Honestly, I knew nothing about AI before your course. I learned about AI thanks to your course. And I learned this: these children can become independent thanks to AI” (P10). Participants indicated that AI would reduce special education teachers’ workload and save time, but might also make teachers complacent: “For a special education teacher, something that would take weeks could be reduced to a day or two—or something that would take a month could be done in three or four days” (P05); “It would reduce teacher stress and workload” (P12); “Since AI serves ready‑made information instantly, teachers might rely solely on that and avoid engaging in broader research” (P01). They emphasized that all teachers should receive training on AI: “In‑service training should be provided for teachers. Because AI is not yet highly popular, teachers lack sufficient knowledge” (P09). They also highlighted AI’s lack of emotion and argued that even if AI could perform other types of teaching, becoming a special education teacher would be difficult; nonetheless, it could generate functional materials: “I don’t think AI can replace a special education teacher. We build emotional bonds. AI cannot fulfill these” (P03); “…perhaps you could load emotions, perhaps it could recognize human emotions and mimic empathy and sympathy, but I don’t think it would truly feel that deep inside” (P09); “Instead of constantly searching for materials, AI‑generated videos, materials, games, and applications would make our job much easier” (P07). Finally, participants suggested that robot teachers might emerge and that teachers could be cloned: “If things become robotic, a robot could even come and deliver a lesson in the future” (P14); “Maybe there could be teacher cloning” (P10).

### Child and family

**Fig 3:**
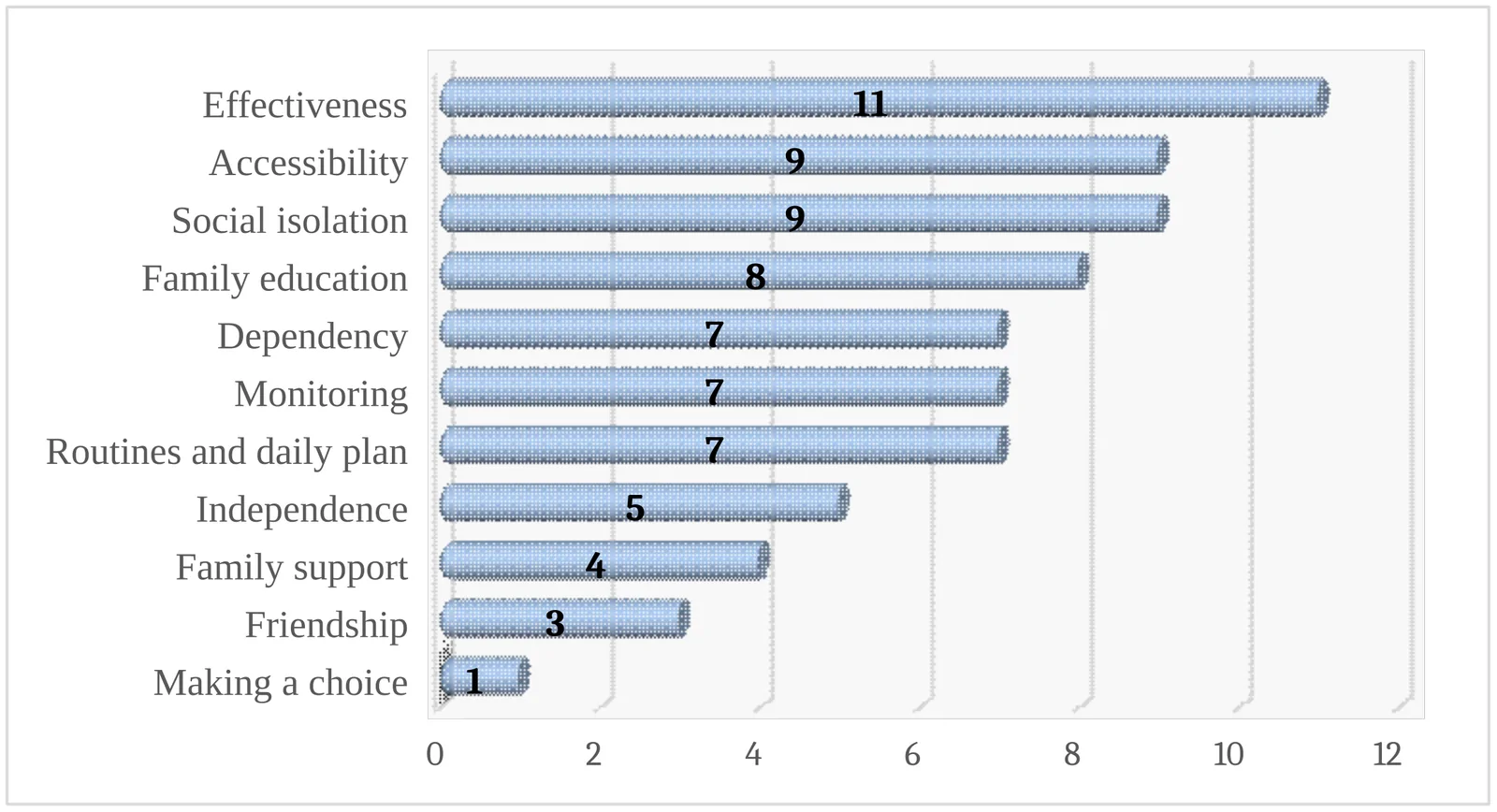
Distribution of categories and frequencies related to child and family.

The categories and frequency values associated with the Child and Family theme are presented in Fig 3.

Participants stated that AI could be particularly effective in the domains of intellectual disability and autism: “Because AI is artificial, for these children—whose decision‑making, thinking, and reasoning are more limited than typically developing peers—AI could help them learn decision‑making, reasoning, and similar skills” (P12). While noting that AI could facilitate families’ access to information, participants also warned of potential social isolation and AI dependency for children with special needs: “Families often need someone at every moment; when a problem arises, they can immediately ask AI. Teachers are not always available, but AI is always there” (P10). “It could reduce social interaction. The children we work with generally have social deficits; these could be exacerbated because, ultimately, they would be interacting with a robot rather than a living person” (P06); “A child may not be independent—relying solely on AI and encountering problems when it is unavailable” (P01). Participants emphasized the need to educate families about AI: “Families must be informed—but they also need to use it. Training for families is necessary” (P10). They further noted that AI could facilitate monitoring of children with special needs, plan routines, and thus support parents: “It would make it easier to track the student—if it could record the prior and subsequent levels” (P03); “With AI, one could easily create a daily plan for the student” (P09); “It could reduce family stress and anxiety and provide support” (P08). Within the Child and Family theme, independence-related perceptions were particularly visible. A salient pattern in participants’ accounts was the perception that, with AI support, children with special needs could come to develop independence, form friendships, and develop choice‑making skills: “And I learned this: these children can become independent thanks to AI” (P10); “AI could even be a friend—helping express and meet needs” (P01); “When a child with autism uses AI, they could learn to make decisions and engage in reasoning through AI” (P12).

### Education

**Fig 4:**
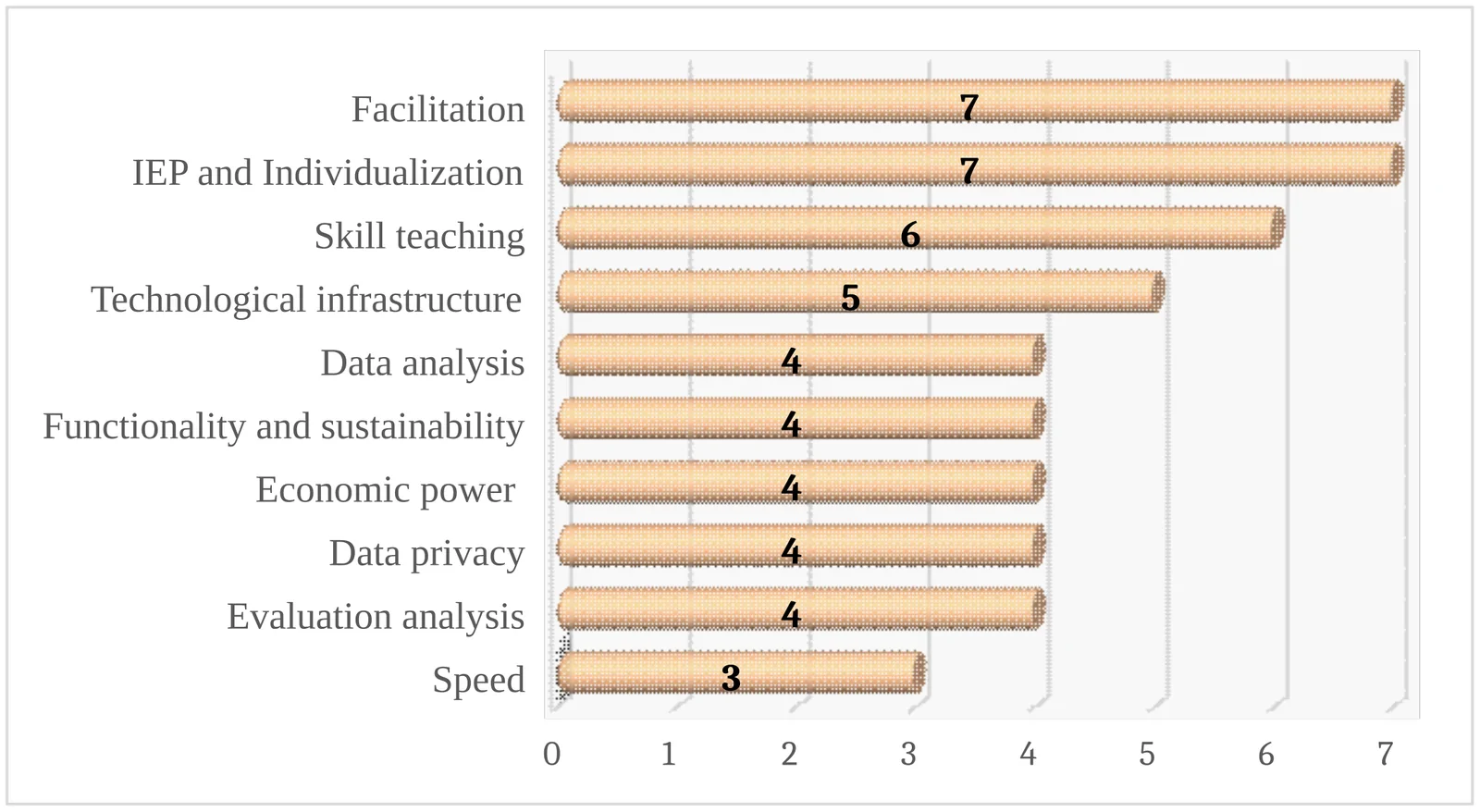
Distribution of categories and frequencies related to education.

The categories and frequency values associated with the Education theme are presented in Fig 4.

Participants stated that AI could readily support individualization, one of the core components of special education, thus facilitating educational processes: “Imagine AI preparing the IEP for you and developing strategies on your behalf” (P09); “We could leverage many applications during instruction; this would ease the work of both teachers and families” (P02). They also indicated that AI could be used to teach skills and sustain instruction: “Brushing teeth may seem simple, but a child must know to do it in the morning, at noon, and in the evening. Many families do not know how to teach this; applications could help” (P03); “We cannot observe students outside; families often struggle and teachers may not know what to do. With AI‑enabled applications, we could continue education at home, increase fluency, and facilitate generalization” (P02). Participants stressed the need for adequate technological infrastructure in educational settings and the economic resources this requires: “To be able to use it, there must be a digital device in every school. A system should be established starting from the most basic needs of the country” (P13); “A family with sufficient means could purchase a robot to act like a shadow teacher constantly by the child’s side-improving the family’s quality of life and saving time while benefiting the student tremendously” (P04). They also observed that AI could conduct data and assessment analyses in special education, accelerate processes, yet raise concerns about data privacy: “AI could easily analyze student data and perform automatic evaluations. Sometimes we are dissatisfied with an evaluation and repeat it; this creates workload for teachers and parents and can be exhausting for the child. If there were a scientifically validated AI tool, it would be great if families could obtain evaluations on a phone without the stress of doctor visits. But there are issues of privacy and data security-AI might share the child’s experiences or images with third parties” (P12).

### Artificial intelligence and future

**Fig 5:**
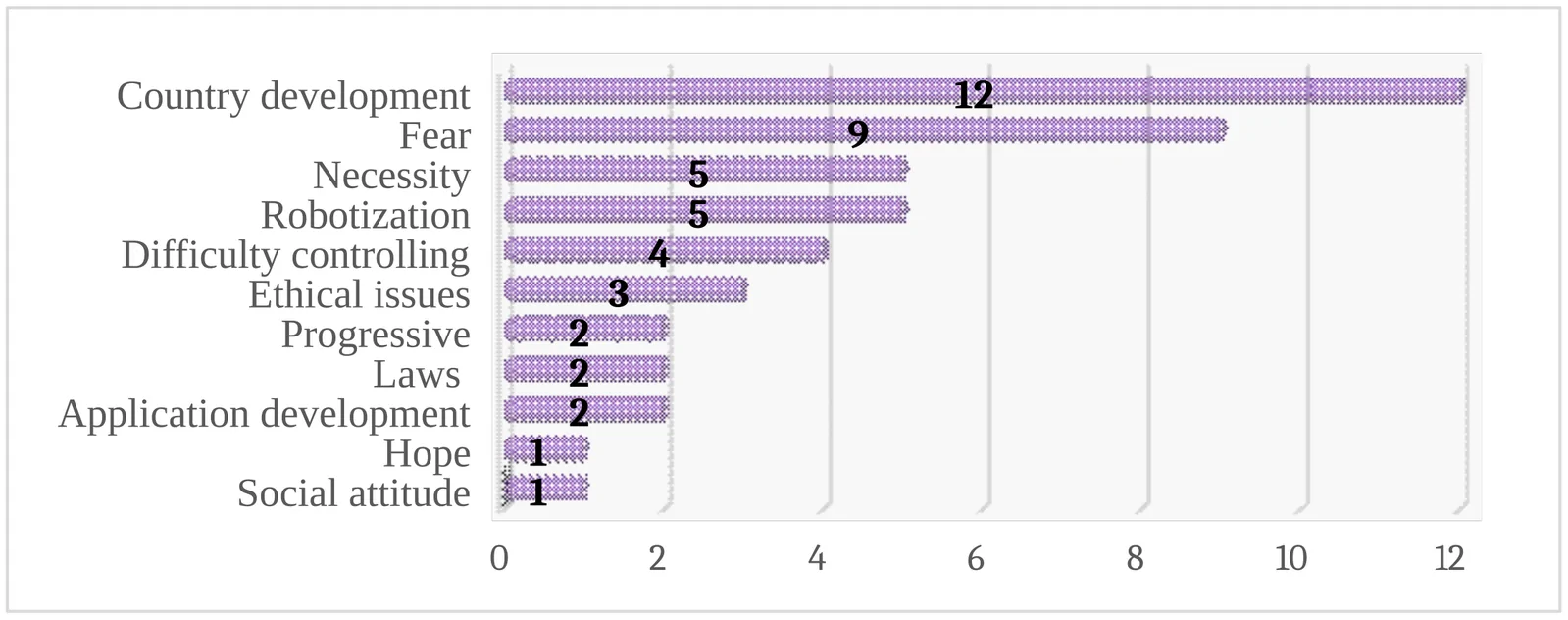
Distribution of categories and frequencies related to artificial intelligence and future.

The categories and their corresponding frequency values for the Artificial Intelligence and Future theme are presented in Fig 5.

Participants stated that AI is necessary, yet national development plays a major role: “I think our country is far behind in technology; I believe nothing has been done in AI” (P14). “I think it is now necessary to use AI in special education; it should be entrenched in the education system” (P05). They expressed fears regarding AI’s future, loss of control and increasing robotization: “The idea of AI becoming conscious truly scares me. I wouldn’t want that at all” (P09); “I do not believe we could survive against AI in the future” (P06); “Robots will come to the fore in education and may replace us, although I am not sure how their training would work” (P14). Participants stated that AI would develop gradually and highlighted the importance of law and ethics: “I do not find it ethical, but there is a program that learns many things much faster than we do” (P09); “It could violate privacy, if it records students’ videos or photos, which it might. We cannot know; we cannot trust how it is managed” (P04). They also suggested that highly advanced applications could be developed with AI, societal attitudes would need to change, and ultimately that AI represents a source of hope for special education: “We could design AI‑supported applications for concept teaching, autism, intellectual disability, and daily living skills” (P05); “There is a negative attitude, people say, ‘Can you even chat with ChatGPT?’, this is a bad situation in Türkiye” (P13); “I think AI is a hope for them; if used adequately, it could yield benefits beyond what we can imagine” (P10).

## Discussion

The findings reported below are interpreted within the inferential register appropriate to a qualitative-phenomenological study with a single-institution sample of 14 participants. They are presented as the meanings that these participants attributed to artificial intelligence in special education at the moment of interview, not as estimates of perceptions in the wider population of Turkish pre-service special education teachers [65]. Findings, presented here as participants’ perceptions rather than as predictions or causal claims, clustered under four themes. Within the Teacher theme, which produced the highest frequency of coded segments, most participants articulated the view that AI could come to assume aspects of the teaching role in the future. This aligns with Chan and Tsi [74], who report participants believing that AI could replace teachers. However, as emphasized by the majority in that study and noted by Nikitina and Ishchenko [75], AI is unlikely to fully undertake the role due to teachers’ emotional, creative, and pedagogical attributes. Thus, the participants’ divergent perceptions cohere with debates in the literature [76]. Their perspective that AI could soon serve as a ‘teaching assistant’ resonates with emerging approaches that position AI as a ‘teaching assistant’ [77]. Participants’ perception that AI could reduce teacher workload and accelerate routine instructional processes (through AI-supported materials, assessment tools, and data-analysis applications) is consistent with a sizeable body of work in this area [56,78,79], and likewise their perception that AI could support individualized instruction parallels findings reported elsewhere [80–82]. Conversely, their concerns that AI might ‘make teachers lazy’ align with critiques that over‑reliance on AI could constrain teachers’ pedagogical creativity [83,84]. Accordingly, participants’ dual stance reflects AI as a source of both opportunities and risks for the teaching profession. Finally, the emphasis that all teachers should receive AI training mirrors findings in the literature on teachers’ demands to develop digital competencies [85,86]. Notably, the OECD TALIS report indicates that many teachers are willing to enhance their digital skills to use information and communication technologies more effectively in instruction [87], suggesting that AI literacy should be integral to teacher education.

In the Child and Family theme, participants noted that AI could facilitate families’ access to information. This aligns with studies showing AI’s capacity to provide individualized information and support to families and to develop accessible solutions for daily living, social interaction, and communication in autism [88]. Similarly, Alanazi et al. [89] and Lameras and Arnab [22] report that AI‑based technologies can meaningfully support families’ daily experiences. At the same time, participants warned of social isolation and technology dependence, corroborating earlier research [90–92]. The dual character of participants’ accounts, hopeful about pedagogical augmentation yet alert to risks of dependence and disconnection, frames the use of AI in special education as a domain requiring sustained ethical and pedagogical deliberation. Within the Education theme, participants argued that AI simplifies individualization, enables sustainable skill instruction, and accelerates data and evaluation processes, findings consistent with prior work [18,19,22,44,55]. They also voiced concerns about privacy and data security, particularly risks of sharing students’ personal information and learning‑process data with third parties, which the literature identifies among the most frequently debated ethical issues in AI‑supported education [23,24,78,93]. The participants expressed the view that a sufficient technological infrastructure would be necessary for artificial intelligence to function effectively in educational settings, and that this would be directly related to economic capacity, which aligns with the literature. Vesna et al. [94] report that infrastructure deficiencies and socioeconomic barriers constitute significant obstacles to the access of AI-supported educational tools, and that these barriers risk widening educational inequalities through the digital divide. The participants’ awareness of these issues, voiced from a Turkish institutional setting, parallels concerns expressed in the international literature.

In the Artificial Intelligence and Future theme, participants expressed worries that AI could become uncontrollable, fuel robotization, and threaten human roles—concerns aligned with the ‘AI anxiety’ literature [95,96]. They further stressed the need for ethical principles and regulatory frameworks—consistent with studies highlighting ethical dilemmas and regulation needs in AI‑in‑education [97,98]. In addition, some pre-service special education teachers indicated that AI offers promising opportunities for special education and that societal attitudes should shift accordingly. This finding is consistent with studies in the literature demonstrating that AI can provide innovative solutions to the learning processes of individuals with special needs [18,19,22,44,55,84,88]. Therefore, this theme reflects that teacher candidates’ future-oriented perspectives encompass both risk and opportunity dimensions.

### Repositioning AI in special education: From deficit-fix to self-determination support

A second reading of the participants’ accounts suggests that the findings of this study are most generatively interpreted not through a deficit-fix logic in which AI is applied to a child with a disability in order to remediate the disability, but through the lens of Self-Determination Theory and the causal agency framework articulated by Shogren and colleagues [99] and developed in the recent synthesis of Wehmeyer [100]. Self-Determination Theory positions autonomy, competence, and relatedness as basic psychological needs whose satisfaction supports human flourishing across the lifespan, and the causal agency framework identifies the conditions under which individuals come to act as the primary causal agents of their own lives.

Three features of the participants’ accounts become legible in a different way under this rereading. First, the recurring participant statement that AI could enable children with special needs to become more independent, which prompted the title of the manuscript, can be read not as a claim that independence is conferred on the child by the technology, but as an early intuition that AI may function as an environmental support that lowers the cost of expressing autonomy. On this reading, the locus of agency remains with the child, and AI occupies the role of a scaffold that the child may, in collaboration with a teacher and family, choose to use. Second, the participants’ simultaneous worry that AI could foster dependence and social withdrawal, voiced repeatedly across the four themes, is consistent with a tacit recognition that environmental supports cease to support autonomy at the point at which they begin to substitute for it. The participants did not have the conceptual vocabulary of Self-Determination Theory at their disposal, but the structure of their ambivalence about dependency anticipates the boundary that the theory makes explicit. Third, the participants’ framing of AI as a possible ‘teaching assistant’ rather than as a replacement for the teacher [77] is in turn consistent with an ecological-supports model of self-determination, in which the teacher remains the principal pedagogical and relational agent and AI functions as one component of the support environment.

The implication of this rereading for teacher preparation is that pre-service special education teachers should be introduced to AI not as an instrument applied to children with disabilities, but as a tool to be co-directed with them. Co-direction here means that the child’s preferences, interests, and self-defined goals organize the use of the tool, that the child is supported in forming and expressing those preferences [101], and that the teacher’s role is to scaffold the child’s developing capacity to make decisions about whether, when, and how to use AI. This shift in framing has practical consequences for the design of AI-literacy components in teacher education programs, including the inclusion of person-centered planning case studies, of disability-led design examples, and of explicit instruction in the language and ethics of supported decision-making.

We note, as a reflexive observation, that the absence of this framing in the original version of the manuscript reflects a wider tendency in the international literature on AI in special education to center the technology rather than the person who is to use it. The repositioning offered here therefore represents a contribution to a broader reorientation that the field is, in our view, due.

### Limitations

Beyond the inferential limits that follow from the qualitative-phenomenological design with a small single-institution sample, three layers of contextual shaping bound the perceptions that the participants articulated, and we make those layers explicit so that the reader is in a position to judge the analytic transferability of the findings to other settings. The cultural layer comprises the Turkish national context in which the participants live and study. The regulatory frameworks for artificial intelligence, the public discourses surrounding disability and inclusion, the role of the state in special education provision, and the prevailing assumptions about the relationship between technology and the family differ in identifiable ways from those of other national settings, and these differences will have shaped the imagery, the examples, and the evaluative vocabulary that the participants drew upon. The institutional layer comprises the Turkish public university and the special education department within which the participants completed their undergraduate studies. The local academic discourse, the curriculum sequence, the available technological infrastructure, and the local norms of disability research will have shaped what counts in that setting as an obvious, as a controversial, or as an unconventional statement about AI in special education. The pedagogical layer comprises the specific technology-in-special-education course that all participants had completed prior to the interviews, and which was taught by a member of the research team. The conceptual vocabulary, the case examples, and the evaluative framings introduced in that course will have shaped the language in which the participants formulated their perceptions during the interview, in ways that the participants themselves may not have made explicit. Taken together, these three layers do not invalidate the findings but locate them: the perceptions reported here are the perceptions of pre-service special education teachers situated at a particular cultural, institutional, and pedagogical intersection, and the transferability of the findings to other intersections is for the reader to judge.

### Recommendations for future research

Three directions for future research follow directly from the inferential limits and the conceptual repositioning of the present study. First, future studies should incorporate the perspectives of children and young people with disabilities themselves. The present study sampled the perceptions of those who will, in the near future, deliver AI-supported instruction, but a complete account of AI in special education cannot be developed without the perspectives of those for whom that instruction is intended. We frame this commitment through the Lundy [101] model of children’s participation, which articulates the four conditions, namely space, voice, audience, and influence, under which children’s participation in research becomes meaningful, and which is anchored in Article 12 of the United Nations Convention on the Rights of the Child. Children with disabilities, including those whose communicative repertoires include non-speech modalities, are entitled to participate in research that shapes the use of technologies that will be applied in their education and in their lives. Second, future studies should complement the perception-level data of the present study with use-level data, specifically through classroom observation of actual AI use in special education settings. Observation studies would allow the comparison of what teachers say about AI with what teachers do with AI in interaction with children and would allow the documentation of moments in which AI use supports or undermines children’s autonomy in the sense identified in the Discussion. Third, future studies should sample more broadly across institutions, regions, and preparation programs, in order to address the cultural, institutional, and pedagogical layers of shaping that we acknowledge in the Limitations section, and to begin to chart the regional and national variation in pre-service teachers’ perceptions of AI in special education.

### Recommendations for practice

- In‑Service AI Literacy: Structure modular programs, through partnerships between the Ministry of National Education and universities, to develop teachers’ ethical, effective, and pedagogically grounded AI competencies.
- AI‑Supported IEP Platforms: Develop AI‑based individualized education program (IEP) software that analyzes student profiles and provides actionable recommendations; pilot and integrate into practice.
- Family Workshops and Digital Resilience: Offer workshops to educate families about the functions, limitations, and ethical use of AI tools, supported by guidance units and civil society organizations.
- Ethical Guidelines and Regulation: Establish clear, inclusive, context‑sensitive ethical guidelines for AI in special education (e.g., data privacy, recording policies, child assent).
- Equity in Access: Create public support mechanisms to ensure AI tools are not limited to higher socioeconomic groups and to prevent deepening of the digital divide.

## Conclusion

**Fig 6:**
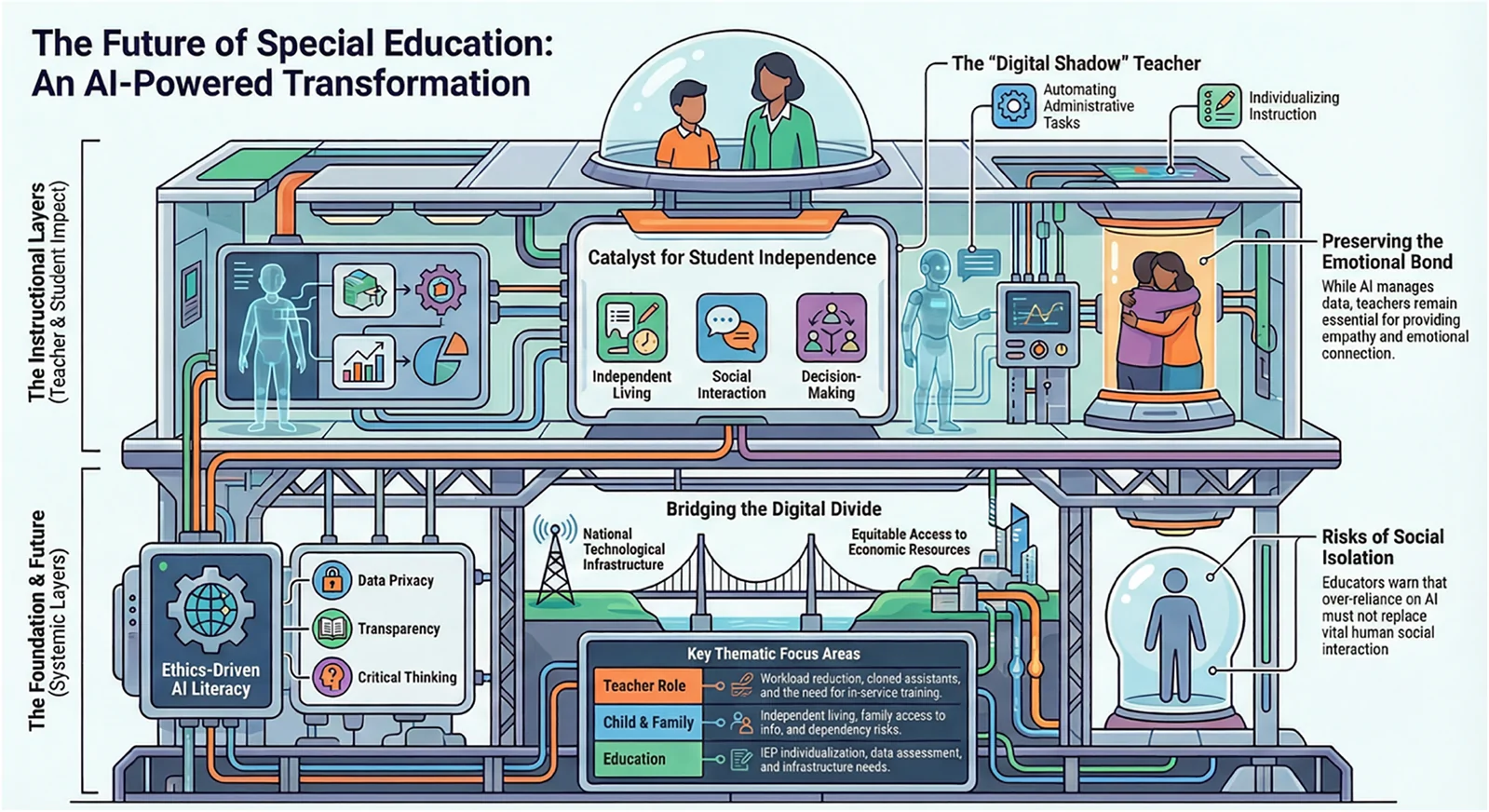
The future of special education: A conceptual framework of AI-powered transformation.

This study provides a multi‑layered interpretation of pre‑service special education teachers’ perceptions of AI in relation to professional roles, student and family interactions, instructional processes, and future expectations. Findings show that the participants in this study conceptualized AI not merely as a technological tool, but as a potential support that, in their accounts, could facilitate individualization in special education, support the development of independent living skills, and transform instructional responsibilities. The recurring participant statement that ‘these children can become independent thanks to AI’ should be read first and foremost as a perception and future-oriented hope articulated by the participants in this study. It signals not only hope, but also an alternative pedagogical imagination regarding the position of individuals with special needs in the digital age, envisioning AI evolving into roles such as ‘assistant teacher,’ ‘digital shadow,’ and ‘personalized companion.’ At the same time, participants articulated critical concerns, emotional limitations, ethical ambiguity, data security, and dependency, indicating a balanced perspective rather than uncritical techno‑optimism. This underscores the need to enrich teacher education not only with digital skills but also with ethics, critical thinking, and AI literacy. By amplifying both hopes and anxieties in an academic register, the study offers a holistic roadmap for AI integration in special education. This roadmap, derived from and synthesizing the instructional and systemic layers of the participants’ accounts of an AI-supported transformation in the special education ecosystem, is visualized in Fig 6.

The figure depicts the multi-dimensional impact of AI integration based on pre-service teachers’ perceptions. The upper section illustrates the “Instructional Layers,” highlighting the shift toward student independence and the evolving role of teachers. The lower section represents the “Systemic Layers,” focusing on the foundational requirements such as ethics-driven AI literacy, data transparency, and bridging the digital divide. Future research should deepen these voices with larger samples and interdisciplinary approaches, contributing to a special education ecosystem grounded not only in technology but in human dignity.

## Supporting information

S1 File Participant information and consent script in Turkish and English. (PDF)

S2 File Semi-structured interview protocol in Turkish and English. (PDF)

S3 File Full codebook including themes, categories, frequencies, definitions, and exemplar quotes. (DOCX)

S4 File De-identified Turkish-language transcripts of all 14 interviews. (DOCX)

S5 File De-identified English-language transcripts of all 14 interviews. (DOCX)
