# Supplementary material for: These children can become independent thanks to artificial intelligence! – A qualitative analysis of pre‑service teachers’ views on the use of artificial intelligence in special education
Source: PLoS One. 2026 Aug 28;21(8):e0355713. doi: 10.1371/journal.pone.0355713 (PMC13524254; doi:10.1371/journal.pone.0355713)
Supplement: Supplementary file 1 [file pone.0355713.s001.pdf]

Supplementary File S1

Participant Information and Verbal Consent Script

Manuscript: PONE-D-26-11239 (revision)

Study: Pre-service special education teachers’ perceptions of the use of artificial intelligence in special education

Ethics approval: Scientific Research and Publication Ethics Committee, Nevşehir Hacı Bektaş Veli University. Decision No. 2024.05.100; Date: 30 April 2024

> ***Note on documentation.*** *The present document sets out the standardised Participant Information and Verbal Consent Script used to operationalise the audio-recorded verbal informed consent procedure approved by the Scientific Research and Publication Ethics Committee of Nevşehir Hacı Bektaş Veli University. The substantive content reproduced below was communicated to each participant at the start of every interview, after the audio recording had been initiated and before any substantive interview questions were posed. Each participant’s affirmative verbal responses to the consent items listed in Section 10 were captured as the opening segment of the corresponding interview audio file and constituted the documented record of consent. The script is provided in both an English version (Part I) and the Turkish original (Part II).*

Part I. English Version

> *Note to interviewer: this script is to be read aloud at the start of each interview, after the audio recording has been initiated and before any substantive interview questions are posed.*

Dear participant,

This interview forms part of a research study examining the perceptions and evaluations of pre-service special education teachers regarding the use of artificial intelligence (AI) in special education. Before we begin, I will share with you a brief description of the study and request your verbal consent. Please feel free to ask me questions at any point during this script or during the interview itself.

1. Purpose of the study

This study aims to understand how pre-service special education teachers perceive and evaluate the use of artificial intelligence in the context of special education, with a particular focus on the possible implications of such use for teachers, for children and families, for instructional processes, and for the future of the field.

2. What your participation involves

You are invited to take part in a semi-structured individual interview of approximately 30 to 45 minutes in duration. I will ask several core questions

about your perceptions of AI in special education, and I may ask follow-up questions to allow you to elaborate on your responses.

3. Voluntary participation and the right to withdraw

Your participation in this study is entirely voluntary. You may decline to answer any question, you may pause or stop the interview at any time, and you may withdraw from the study at any subsequent stage without providing a reason. Should you choose to withdraw, your interview data will be permanently deleted, and your decision will have no academic, professional, or administrative consequences for you.

4. Audio recording

With your consent, our interview will be audio-recorded so that your responses can be transcribed and analysed accurately. The audio file will be stored on a password-protected institutional drive accessible only to the members of the research team. In accordance with the protocol approved by our ethics committee, the audio recording will be retained for five years from the completion of the study, after which it will be permanently destroyed.

5. Confidentiality and anonymisation

Your name, and any information that could directly identify you, will not appear in the published article or in any associated shared document. This includes the names of family members, instructors, classmates, schools, placement sites, or any other person or institution you may mention during the interview. Each participant is assigned a participant code (for example, P01, P02, P03), and all direct and indirect identifiers in your transcript will be replaced with generic descriptors prior to any form of sharing.

6. Open sharing of anonymised data for scientific purposes

With your separate consent on this specific point, the de-identified Turkish-language transcript of your interview will be deposited as a supplementary file alongside the published article. This open-access deposition serves the principle of scientific transparency and enables secondary analysis by other researchers. Before deposition, every direct and indirect identifier in your transcript will be removed according to the anonymisation procedure described above. The anonymisation will then be checked by a member of the research team who did not conduct your interview, in order to provide an independent review of the de-identification.

7. Possible risks and benefits

There are no known physical risks associated with participation in this study. Discussing your perceptions of artificial intelligence in your future professional field may involve a modest degree of intellectual and reflective effort. There is no direct material benefit to participants, although you may find the reflective conversation valuable. The findings are intended to contribute to the international

scientific discussion on the place of artificial intelligence in special education.

8. Contact for questions and concerns

Should you have any questions or concerns regarding this study, either now or at any later point, you may contact the corresponding author of the study or the Scientific Research and Publication Ethics Committee of Nevşehir Hacı Bektaş Veli University at bilimseletik@nevsehir.edu.tr.

9. Ethics approval

This study has been reviewed and approved by the Scientific Research and Publication Ethics Committee of Nevşehir Hacı Bektaş Veli University (Decision No. 2024.05.100; Date: 30 April 2024). The Committee specifically approved the use of audio-recorded verbal informed consent for this study, given its low-risk, interview-only design, and also approved the open-access deposition of the anonymised transcripts as supplementary material, conditional on the explicit consent of each participant.

10. Verbal consent questions

I will now ask you four questions. Please respond aloud, so that your responses are captured by the audio recording.

(i) Do you confirm that you have understood the information I have just provided?

(ii) Do you confirm that you voluntarily consent to participate in this study?

(iii) Do you consent to the audio recording of this interview, and to the use of the recording for transcription and analysis by the research team?

(iv) Do you consent to the open-access deposition of the de-identified transcript of your interview as a supplementary file alongside the published article, accessible to other researchers for secondary analysis?

If your answers to all four questions are affirmative, we will proceed with the interview. If you are uncertain about any item, or if you do not wish to consent to any item, please tell me now. You may decline any individual item, in which case I will record your decision and proceed only with the items to which you have given consent. You may also decline to participate altogether, in which case I will end the audio recording, ask no further questions, and retain no data.

Thank you. We will now begin the interview.

Part II. Turkish Original

> *Görüşmeci için not: bu metin her görüşmenin başında, ses kaydı başlatıldıktan ve görüşme sorularına geçilmeden önce katılımcıya sesli olarak okunmuştur.*

Sayın katılımcı,

Bu görüşme, özel eğitim öğretmen adaylarının özel eğitimde yapay zekâ (YZ) kullanımına ilişkin algılarını ve değerlendirmelerini inceleyen bir araştırmanın parçasıdır. Görüşmemize başlamadan önce çalışma hakkında kısa bir bilgi vereceğim ve sizden sözlü onam talep edeceğim. Bu metnin okunması sırasında ya da görüşmenin herhangi bir aşamasında soru sormaktan çekinmeyiniz.

1. Çalışmanın amacı

Bu çalışma, özel eğitim öğretmen adaylarının özel eğitim alanında yapay zekâ kullanımına ilişkin algılarını ve değerlendirmelerini, özellikle bu kullanımın öğretmenler, çocuklar ve aileler, eğitim süreçleri ve alanın geleceği açısından olası sonuçlarına odaklanarak anlamayı amaçlamaktadır.

2. Katılımınız neyi içeriyor

Sizinle yaklaşık 30 ile 45 dakika sürebilecek yarı yapılandırılmış bireysel bir görüşme yapacağım. Özel eğitimde YZ kullanımına ilişkin algılarınız hakkında temel sorular yöneltecek, cevaplarınızı genişletmeniz için ek sorular sorabilirim.

3. Gönüllü katılım ve çekilme hakkı

Bu araştırmaya katılımınız tamamen gönüllüdür. Herhangi bir soruyu cevaplamayı reddedebilir, görüşmeye herhangi bir aşamada ara verebilir ya da görüşmeyi sonlandırabilir, sonraki herhangi bir aşamada da gerekçe göstermeden çalışmadan çekilebilirsiniz. Çekilmeyi tercih etmeniz halinde görüşme verileriniz kalıcı olarak silinecek, kararınızın sizin için herhangi bir akademik, mesleki ya da idari sonucu olmayacaktır.

4. Ses kaydı

Onamınız doğrultusunda görüşmemiz, yanıtlarınızın doğru biçimde yazıya dökülebilmesi ve çözümlenebilmesi amacıyla ses kaydı ile kaydedilecektir. Ses kaydı yalnızca araştırma ekibi üyelerinin erişebileceği parola korumalı bir kurumsal sürücüde saklanacaktır. Etik kurulumuzun onayladığı protokol uyarınca ses kaydı, çalışmanın tamamlanmasından itibaren beş yıl süreyle saklanacak ve bu sürenin sonunda kalıcı olarak imha edilecektir.

5. Gizlilik ve anonimleştirme

Adınız ya da sizi doğrudan tanımlayabilecek hiçbir bilgi, yayınlanacak makalede ya da herhangi bir başka paylaşım belgesinde yer almayacaktır. Bu kapsama görüşme sırasında bahsetmiş olabileceğiniz aile bireylerinin, hocaların, sınıf arkadaşlarının, okulların, staj yerlerinin ya da başka kişi ve kurumların adları da dâhildir. Her katılımcıya bir katılımcı kodu (örneğin P01, P02, P03) atanmakta,

görüşme metninizdeki tüm doğrudan ve dolaylı tanımlayıcılar, herhangi bir paylaşımdan önce genel ifadelerle değiştirilmektedir.

6. Anonimleştirilmiş verilerin bilimsel amaçla açık paylaşımı

Bu konudaki ayrı onamınız doğrultusunda, görüşmenize ait anonimleştirilmiş Türkçe metin, yayınlanacak makaleye yan dosya olarak eklenecektir. Bu açık paylaşım, bilimsel şeffaflık ilkesine hizmet etmekte ve diğer araştırmacıların ikincil analiz yapmasına olanak tanımaktadır. Paylaşımdan önce görüşme metninizdeki tüm doğrudan ve dolaylı tanımlayıcılar yukarıda anlatılan anonimleştirme prosedürü ile çıkarılacaktır. Anonimleştirme, daha sonra, sizin görüşmenizi yapmamış olan bir araştırma ekibi üyesi tarafından bağımsız bir kontrolden geçirilecektir.

7. Olası riskler ve yararlar

Bu çalışmaya katılımın bilinen herhangi bir fiziksel riski bulunmamaktadır. Gelecekteki mesleki alanınızda yapay zekâya ilişkin algılarınızı tartışmak mütevazı düzeyde zihinsel ve düşünsel çaba gerektirebilir. Katılımcıların doğrudan maddi bir yararı söz konusu değildir; ancak düşünmeyi gerektiren bu sohbeti değerli bulabilirsiniz. Bulguların, özel eğitim alanında yapay zekânın yerine ilişkin uluslararası bilimsel tartışmaya katkı sağlaması hedeflenmektedir.

8. Sorular ve kaygılar için iletişim

Bu çalışmaya ilişkin herhangi bir sorunuz ya da kaygınız olursa, çalışmanın sorumlu yazarına ya da Nevşehir Hacı Bektaş Veli Üniversitesi Bilimsel Araştırma ve Yayın Etiği Kurulu’na bilimseletik@nevsehir.edu.tr adresinden ulaşabilirsiniz.

9. Etik kurul onayı

Bu çalışma, Nevşehir Hacı Bektaş Veli Üniversitesi Bilimsel Araştırma ve Yayın Etiği Kurulu tarafından 30 Nisan 2024 tarihinde 2024.05.100 sayılı kararla incelenmiş ve onaylanmıştır. Etik Kurul, çalışmanın düşük riskli ve yalnızca görüşmeye dayalı tasarımı göz önünde bulundurularak ses kaydına dayalı sözlü bilgilendirilmiş onam yönteminin kullanımını ve katılımcıların açık onamına bağlı olarak anonimleştirilmiş metinlerin yan dosya olarak açık erişimle paylaşımını özel olarak onaylamıştır.

10. Sözlü onam soruları

Şimdi size dört soru yönelteceğim. Yanıtlarınızı sesli olarak veriniz; böylece yanıtlarınız ses kaydına alınmış olacaktır.

(i) Sizinle paylaştığım bilgileri anladığınızı teyit eder misiniz?

(ii) Bu araştırmaya gönüllü olarak katılmaya onam verdiğinizi teyit eder misiniz?

(iii) Görüşmemizin ses kaydının alınmasına ve ses kaydının araştırma ekibi tarafından yazıya dökme ve çözümleme amacıyla kullanılmasına onam veriyor musunuz?

(iv) Görüşmenize ait anonimleştirilmiş metnin, yayınlanacak makaleye yan dosya olarak, ikincil analiz amacıyla diğer araştırmacıların erişimine açık biçimde paylaşılmasına onam veriyor musunuz?

Dört soruya da yanıtınız evetse, görüşmemize başlayacağız. Herhangi bir madde konusunda emin değilseniz ya da herhangi bir maddeye onam vermek istemezseniz, lütfen şimdi belirtiniz. Her bir maddeye ayrı ayrı yanıt verebilirsiniz; bu durumda kararınızı kayda alacağım ve yalnızca onam verdiğiniz maddeler doğrultusunda devam edeceğim. Çalışmaya hiç katılmamayı da tercih edebilirsiniz; bu durumda ses kaydını sonlandıracağım, başka bir soru sorulmayacak ve herhangi bir veri saklanmayacaktır.

Teşekkür ederim. Görüşmemize şimdi başlıyoruz.
