# Supplementary material for: These children can become independent thanks to artificial intelligence! – A qualitative analysis of pre‑service teachers’ views on the use of artificial intelligence in special education
Source: PLoS One. 2026 Aug 28;21(8):e0355713. doi: 10.1371/journal.pone.0355713 (PMC13524254; doi:10.1371/journal.pone.0355713)
Supplement: Supplementary file 2 [file pone.0355713.s002.pdf]

Supplementary File S2

Semi-Structured Interview Protocol

Manuscript: PONE-D-26-11239 (revision)

Study: Pre-service special education teachers' perceptions of the use of artificial intelligence in special education

Ethics approval: Scientific Research and Publication Ethics Committee, Nevşehir Hacı Bektaş Veli University. Decision No. 2024.05.100; Date: 30 April 2024

> ***Note on the protocol.*** *This document sets out the semi-structured interview protocol used in the present study. The protocol comprises fifteen lead questions, asked to all fourteen participants in the same sequence. Consistent with the conventions of semi-structured interviewing, the lead questions were complemented by spontaneous follow-up probes (for instance, 'Why?', 'How so?', 'Could you give an example?', 'Could you elaborate on that?') that arose in response to the substantive content of each participant's account and were not pre-scripted. The protocol is provided in an English translation (Part I) and in the Turkish original used during data collection (Part II). Interviews were conducted in Turkish.*

Part I. English Translation

> *The following fifteen lead questions were asked to all participants in the sequence given below. The interviewer used spontaneous follow-up probes to elicit elaboration where appropriate.*

1. Have you ever used any artificial intelligence tool before? If you have, what kinds of outcomes did you obtain?

2. What are your views on the use of artificial intelligence in the education of individuals with special needs?

3. In which categories of disability do you think artificial intelligence systems would be more effective, and why?

4. What kinds of effects do you think the use of artificial intelligence may have on the daily lives of children and individuals with special needs?

5. What kinds of contributions do you think the use of artificial intelligence could make to special education teachers?

6. What are your views and thoughts on how artificial intelligence systems could be integrated into special education services?

7. Do you think artificial intelligence systems can be used easily and functionally within the existing structure of special education services?

8. Do you think that, in the future, artificial intelligence systems will take the place of special education teachers?

9. What kinds of contributions do you think the use of artificial intelligence could make to the families of individuals with special needs?

10. Do you think the use of artificial intelligence systems would be beneficial in the collaboration between families and special education teachers? If so, what could those benefits be?

11. What kinds of negative aspects could artificial intelligence have for special education teachers, for students with special needs, and for parents?

12. How do you think the use of artificial intelligence in the education of children with special needs will be shaped in the future?

13. Where do you see our country positioned in the field of artificial intelligence?

14. Do you have an experience or an idea on this topic that you would like to share?

15. Is there anything else you would like to add?

Part II. Turkish Original

Aşağıdaki on beş ana soru, tüm katılımcılara aynı sırayla yöneltilmiştir. Görüşmeci, katılımcıların yanıtlarını genişletmesine olanak vermek için doğal seyir içinde takip soruları kullanmıştır.

1. Daha önce herhangi bir yapay zekâ aracı kullandınız mı? Kullandıysanız ne gibi sonuçlar elde ettiniz?

2. Özel gereksinimli bireylerin eğitimlerinde yapay zekâ kullanımı konusunda görüşleriniz nelerdir?

3. Yapay zekâ sistemlerinin hangi yetersizlik türlerinde daha etkili olacağını düşünüyorsunuz? Neden?

4. Yapay zekâ kullanımının özel gereksinimli çocukların ya da bireylerin günlük yaşamlarına ne tür etkileri olabileceğini düşünüyorsunuz?

5. Yapay zekâ kullanımının özel eğitim öğretmenlerine ne tür katkıları olabileceğini düşünüyorsunuz?

6. Yapay zekâ sistemlerinin özel eğitim hizmetleri içerisine nasıl entegre edilebileceği konusunda görüş ve düşünceleriniz nelerdir?

7. Yapay zekâ sistemlerinin mevcut özel eğitim hizmetlerinin yapısı içerisinde kolay ve işlevsel olarak kullanılabileceğini düşünüyor musunuz?

8. İleride yapay zekâ sistemlerinin özel eğitim öğretmenlerinin yerini alacağını düşünüyor musunuz?

9. Yapay zekâ kullanımının özel gereksinimli bireylerin ailelerine ne tür katkıları olabileceğini düşünüyorsunuz?

10. Yapay zekâ sistemlerinin aile ve özel eğitim öğretmeni işbirliğinde kullanımının yararlı olacağını düşünüyor musunuz? Yararlı olacağını düşünüyorsanız bu yararlar neler olabilir?

11. Yapay zekânın özel eğitim öğretmeni, özel gereksinimli öğrenci ve ebeveynler için ne gibi olumsuz tarafları olabilir?

12. Özel gereksinimli çocukların eğitimlerinde yapay zekâ kullanımının gelecekte nasıl şekilleneceğini düşünüyorsunuz?

13. Ülkemizi yapay zekâ alanında nerede görüyorsunuz?

14. Bu konuda paylaşmak istediğiniz bir anınız ya da bir fikriniz var mı?

15. Eklemek istediğiniz bir açıklamanız var mı?
