# Supplementary material for: These children can become independent thanks to artificial intelligence! – A qualitative analysis of pre‑service teachers’ views on the use of artificial intelligence in special education
Source: PLoS One. 2026 Aug 28;21(8):e0355713. doi: 10.1371/journal.pone.0355713 (PMC13524254; doi:10.1371/journal.pone.0355713)
Supplement: Supplementary file 3 [file pone.0355713.s003.docx]

**Supplementary File S3**

**Codebook: Themes, Categories, Definitions, and Exemplar Quotes**

**Manuscript:** PONE-D-26-11239 (revision)

**Study:** Pre-service special education teachers’ perceptions of the use of artificial intelligence in special education

**Ethics approval:** Scientific Research and Publication Ethics Committee, Nevşehir Hacı Bektaş Veli University. Decision No. 2024.05.100; Date: 30 April 2024

**Coding scheme:** 4 themes, 45 categories, 243 coded segments across 14 interview transcripts

***Note on the codebook.*** *This document presents the final codebook of the study, organised by the four themes generated through inductive analysis of the interview corpus. Each theme is presented in a table that lists the categories belonging to the theme, the frequency of coded segments per category across the fourteen participants, a working definition of the category, and one exemplar quote (in English translation; the Turkish originals are available in the de-identified transcripts of Supplementary File S4). The frequencies reported here correspond exactly to the numbers reported in the manuscript. The category labels are given in both their Turkish original and an English translation. Quoted material is attributed to participants using the codes P01 through P14, consistent with the codes used in the published article and in Supplementary File S4.*

**Summary of the coding scheme**

| **Theme** | **Categories** | **Coded segments (n)** |
| --- | --- | --- |
| Theme 1. Teacher (Öğretmen) | 12 | 78 |
| Theme 2. Education (Eğitim) | 10 | 48 |
| Theme 3. Child and Family (Çocuk ve Aile) | 12 | 71 |
| Theme 4. AI and the Future (AI ve Gelecek) | 11 | 46 |
| **Total** | **45** | **243** |

**Theme 1. Teacher**

*Turkish original: Öğretmen · Categories: 12 · Total coded segments: n = 78*

| **Category** | **n** | **Definition** | **Exemplar quote (English translation)** |
| --- | --- | --- | --- |
| **AI will replace the teacher** *Öğretmen olacak* | **14** | Perceptions that AI may in the future take over, or substantially displace, aspects of the special education teaching role. | *P14: "We need to be very intertwined with AI. Teachers who do not know technology will not have a place in the future."* |
| **Assistant teacher** *Yardımcı öğretmen* | **10** | Perceptions positioning AI as a teaching assistant that supports rather than replaces the teacher. | *P06: "AI could be of great help to special education teachers in terms of time and as a multi-purpose material assistant."* |
| **Contribution of the course** *Dersin katkısı* | **10** | Participants' reflections on how the technology-in-special-education course shaped their awareness and use of AI tools. | *P10: "I did not know anything about AI before your course. I learned through your course."* |
| **Time saving** *Zaman tasarrufu* | **8** | Perceptions that AI tools save teacher time across instructional preparation and delivery. | *P06: "Special education teachers will save significant time with AI. The work of ten people could be supervised by one AI-supported teacher."* |
| **Teacher training** *Öğretmen eğitimi* | **8** | Perceptions about the need for in-service and pre-service AI training for special education teachers. | *P09: "Before family training, teachers themselves need in-service training on AI, since AI has not yet become widely familiar."* |
| **Workload reduction** *İş yükünü azaltma* | **6** | Perceptions that AI reduces overall teacher workload, including material preparation, assessment, and routine administrative tasks. | *P01: "AI will save the teacher from unnecessary time spent, unnecessary effort, and exhaustion."* |
| **Special education teacher cannot be replaced** *Özel eğitim öğretmen olamaz* | **6** | Perceptions that AI cannot replace special education teachers because of emotional, relational, and pedagogical attributes specific to the human teacher. | *P03: "AI cannot replace the special education teacher. We build an emotional bond. We respond moment by moment to the student's reactions. AI cannot do this."* |
| **Material preparation** *Materyal hazırlama* | **5** | Perceptions of AI's role in producing instructional materials, videos, applications, and games. | *P07: "Rather than searching for materials, AI-produced videos, materials, games, and applications make this work much easier for us."* |
| **No emotion** *Duygu yok* | **4** | Perceptions that AI lacks emotional capacity, which limits its educational role with children with special needs. | *P09: "It may imitate emotions, but I do not think it would actually feel them deep inside."* |
| **Robot teacher** *Robot öğretmen* | **3** | Perceptions of future embodied robot teachers replacing or supplementing human instruction. | *P14: "In the coming years, robots could come and teach classes. We should not see this as impossible."* |
| **Making teachers lazy** *Öğretmeni tembelleştirme* | **3** | Concerns that over-reliance on AI may erode teachers' pedagogical effort, creativity, and skills. | *P06: "Could AI make teachers lazy? Definitely yes. Teachers may start thinking, 'AI already does it, why should I?'"* |
| **Cloning** *Klonlama* | **1** | The idea of replicating distinguished teachers through AI cloning of their expertise and style. | *P10: "Cloning of teachers may be possible. Would you want to take a lesson from a newly graduated teacher or from a senior teacher? I would choose to be taught by the clone of a senior teacher."* |

**Theme 2. Education**

*Turkish original: Eğitim · Categories: 10 · Total coded segments: n = 48*

| **Category** | **n** | **Definition** | **Exemplar quote (English translation)** |
| --- | --- | --- | --- |
| **IEP and individualisation** *BEP ve Bireyselleştirme* | **7** | Perceptions of AI's role in preparing Individualised Education Plans and in tailoring instruction to individual learners. | *P05: "When you enter the child's characteristics and needs into AI, it easily prepares an IEP for you."* |
| **Facilitation** *Kolaylaştırma* | **7** | Perceptions of AI as a general facilitator of educational processes for both teachers and learners with special needs. | *P05: "Once it is integrated, education will become much easier, both for teachers and for children with special needs."* |
| **Skill instruction** *Beceri öğretimi* | **6** | Perceptions of AI's role in teaching specific functional, academic, and daily-living skills. | *P10: "Skill instruction is essential for students with intellectual disabilities. The child needs to live independently, and AI can support that."* |
| **Technological infrastructure** *Teknolojik altyapı* | **5** | Perceptions of the technological infrastructure required for meaningful AI deployment in special education settings. | *P13: "We need a digital device in every school. Some schools in central Anatolia still lack the basics. We need to start there."* |
| **Assessment analysis** *Değerlendirme analiz* | **4** | Perceptions of AI's role in conducting student assessment, including pre-instructional readiness and diagnostic analysis. | *P08: "We can conduct a much more detailed student analysis using AI's broad capacity, which we could not do on paper."* |
| **Data privacy** *Veri gizliliği* | **4** | Concerns about the protection of student and family data in AI-supported educational settings. | *P04: "AI could violate privacy. It may record student videos and photos, and we cannot be sure how those are managed."* |
| **Economic resources** *Ekonomik güç* | **4** | Perceptions of how economic capacity, at family and at national level, shapes access to AI-supported special education. | *P04: "Families with strong financial means could purchase a robot, a shadow teacher of sorts, for their child. They would also gain time for themselves."* |
| **Functionality and continuity** *İşlevsellik ve süreklilik* | **4** | Perceptions of AI's capacity to provide functional, continuous support beyond the limits of human teacher availability. | *P12: "AI could support these children twenty-four hours a day, which a teacher cannot. A teacher gets tired; AI does not."* |
| **Data analysis** *Veri analizi* | **4** | Perceptions of AI's role in analysing student performance data to inform instructional decisions. | *P11: "AI determines where the child is ahead and where the child is behind in subjects, and offers tailored activities accordingly."* |
| **Speed** *Hız* | **3** | Perceptions of speed advantages provided by AI in information access and instructional processes. | *P01: "I obtained results quickly: gathering data, accessing core information without getting bogged down in unnecessary details."* |

**Theme 3. Child and Family**

*Turkish original: Çocuk ve Aile · Categories: 12 · Total coded segments: n = 71*

| **Category** | **n** | **Definition** | **Exemplar quote (English translation)** |
| --- | --- | --- | --- |
| **Effectiveness** *Etkililik* | **11** | Perceptions of the relative effectiveness of AI across different categories of disability and learner profiles. | *P09: "I think AI would be more effective for students with mild-level disabilities and for those whose relationship with technology is already good."* |
| **Social isolation** *Sosyal izolasyon* | **9** | Concerns that AI use may lead to social withdrawal and isolation of children with special needs from peers and family. | *P05: "If a child with autism becomes attached to the screen and to AI, they will be deprived of social life. Their social circle is already small; this would make it disappear."* |
| **Accessibility** *Ulaşılabilirlik* | **9** | Perceptions of AI's role in making educational and family support resources more accessible. | *P02: "AI-developed applications make it possible to continue the child's education at home, supporting fluency and generalisation."* |
| **Family education** *Aile eğitimi* | **8** | Perceptions of AI's role in educating and informing families of children with special needs. | *P14: "Families can take training about AI themselves and learn their rights through AI, so they can give their children better support."* |
| **Monitoring** *Takip* | **7** | Perceptions of AI's role in monitoring and tracking child progress over time, and in communicating this to teachers and families. | *P11: "AI tracks the progression of the student using both the teacher's input and its own analysis. It also evaluates whether the instruction was effective."* |
| **Dependency** *Bağımlılık* | **7** | Concerns that children with special needs may become dependent on AI, with negative effects on autonomy and screen exposure. | *P07: "If children with severe intellectual disability and autism are constantly exposed to screens, this could have negative effects, including phone addiction and excessive screen exposure."* |
| **Independence** *Bağımsızlık* | **5** | Perceptions of AI's role in supporting the independence of children with special needs. | *P10: "These children can become independent thanks to AI. I did not know this before your course; I learned this through your course."* |
| **Disability types** *Yetersizlik türleri* | **5** | Discussion of the disability categories for which AI is perceived to be more applicable. | *P05: "AI will be more effective for intellectual disability and autism. For sensory disabilities, the need is more easily addressed through other devices."* |
| **Support for families** *Aileye destek* | **4** | Perceptions of AI's supportive role for families navigating special education. | *P07: "AI-produced videos and AI-supported learning programs will be a great guide for parents who do not know how to support their children."* |
| **Companion** *Arkadaş* | **3** | Perceptions of AI as a companion or friend for children with special needs. | *P01: "AI could even be a friend. It could help the child meet their needs, express them, and convey them visually when speech is difficult."* |
| **Routines and daily plans** *Rutinler ve günlük plan* | **2** | Perceptions of AI's role in supporting daily routines and structured planning for children with special needs. | *P03: "AI can monitor the daily routines of these children and remind them what needs to be done, showing it visually and audibly."* |
| **Choice-making** *Seçim yapma* | **1** | Perceptions of AI as a tool that supports children's choice-making, including in everyday situations. | *P01: "When food comes, AI could show pictures of foods the child likes and does not like, helping the family understand the child's preferences."* |

**Theme 4. AI and the Future**

*Turkish original: AI ve Gelecek · Categories: 11 · Total coded segments: n = 46*

| **Category** | **n** | **Definition** | **Exemplar quote (English translation)** |
| --- | --- | --- | --- |
| **National position in AI** *Ülke gelişmişliği* | **12** | Comparative perceptions of Türkiye's standing in AI development relative to other countries. | *P13: "We are significantly behind. America, Japan, and Europe are far ahead. Our country needs to develop robots."* |
| **Fear** *Korku* | **9** | Fears and anxieties about the future trajectory of AI development. | *P09: "I hope AI grows in a controlled way. I feel as if robotic wars are awaiting us. I hope I will not see that future."* |
| **Robotisation** *Robotlaşma* | **5** | Perceptions of the future embodiment of AI in robotic form, including hybrid human-robot futures. | *P09: "It could be a robot, or a chip, or some other component people carry. Like the glasses I am wearing now. It could become part of one's body."* |
| **Necessity** *Gereklilik* | **5** | The belief that AI use in special education is necessary and inevitable. | *P01: "I definitely think AI should be used. I think we should become aware of it, and that it should enter our lives."* |
| **Control difficulty** *Kontrol güçlüğü* | **4** | Concerns about the difficulty of controlling AI as it develops. | *P04: "Perhaps AI will control us, without us realising. We may find ourselves having to manage something we cannot manage."* |
| **Ethical concerns** *Etik sorunlar* | **3** | Ethical concerns about the deployment of AI in special education. | *P07: "I do not find it ethically appropriate, but as you also said, there is a program in front of us that learns many things much faster than we do."* |
| **Application development** *Uygulama geliştirme* | **2** | The need for the development of dedicated AI applications for special education. | *P12: "Many applications could be designed with AI specifically for children's instruction."* |
| **Laws** *Yasalar* | **2** | The need for legal and regulatory frameworks governing AI in education. | *P06: "Decrees with the force of law could be issued. The state could enforce it through regulation."* |
| **Gradual implementation** *Aşamalı* | **2** | The recommendation that AI integration into special education should proceed gradually, through pilot programmes. | *P06: "This cannot happen all at once. It could be launched as a five-year plan, starting in pilot schools, and then expanded."* |
| **Social attitude** *Toplumsal tutum* | **1** | Societal attitudes toward AI in Türkiye. | *P13: "There is such a negative attitude in Türkiye. We are significantly behind."* |
| **Hope** *Umut* | **1** | Hopeful perceptions about AI's future in special education. | *P09: "I hope AI will not move in a direction that harms us, but supports us."* |
