# Supplementary material for: These children can become independent thanks to artificial intelligence! – A qualitative analysis of pre‑service teachers’ views on the use of artificial intelligence in special education
Source: PLoS One. 2026 Aug 28;21(8):e0355713. doi: 10.1371/journal.pone.0355713 (PMC13524254; doi:10.1371/journal.pone.0355713)
Supplement: Supplementary file 4 [file pone.0355713.s004.docx]

**Supplementary File S4**

**De-Identified Interview Transcripts (Turkish Original)**

**Manuscript:** PONE-D-26-11239 (revision)

**Study:** Pre-service special education teachers’ perceptions of the use of artificial intelligence in special education

**Ethics approval:** Scientific Research and Publication Ethics Committee, Nevşehir Hacı Bektaş Veli University. Decision No. 2024.05.100; Date: 30 April 2024

**Number of transcripts:** 14 (P01 through P14)

***Note on anonymisation and consent capture.*** *The transcripts reproduced below have been anonymised for open public deposition. All participants provided explicit verbal consent, captured on the original audio recording, for the open-access sharing of their de-identified transcripts as supplementary material. The opening consent exchange of each interview, originally captured at the start of the audio file and not previously transcribed, has been transcribed for the present deposition and appears at the start of each transcript. The anonymisation procedure was carried out following the practice-oriented guidance of Saunders, Kitzinger and Kitzinger (2015) and Kaiser (2009). Each participant has been assigned a participant code (P01 through P14), corresponding to the codes used in the published article. The interviewer’s utterances are labelled G (the Turkish initial for Görüşmeci, ‘interviewer’). All direct personal identifiers have been replaced with generic descriptors. The general academic address term ‘hocam’ used by participants has been retained, as it is a non-identifying form of address commonly used in Turkish academic contexts. Country and place names at the level of nation or continent, and names of artificial intelligence tools and platforms, have been retained as study content rather than identifiers.*

**P01 — Interview Transcript**

**G:** Görüşmemize başlamadan önce kısaca söyleyeyim. Bu, özel eğitim öğretmen adaylarının yapay zekâ konusundaki görüşleri üzerine bir araştırma. Sana birkaç soru soracağım, ses kaydı alacağım. Söylediklerin tamamen anonim olarak işlenecek, isim bilgilerin hiçbir yerde geçmeyecek. Ayrıca anonimleştirilmiş hâliyle transkriptin makaleye yan dosya olarak başka araştırmacıların erişimine açık şekilde paylaşılabilir. Bunlara onam veriyor musun?

**P01:** Evet hocam, veriyorum.

**G:** Teşekkür ederim, o zaman ilk soruma başlıyorum.

**P01:** Ben yapay zekanın kullanılması gerektiğini kesinlikle düşünüyorum. Bu kullanımın şöyle olabileceğini düşünüyorum; bizim özel gereksinimli bireylerin ihtiyacını tespit edeceğimizi, hangi konularda yapay zekadan yardım alabileceğimizi, bizim bulmamızı, ondan sonra bu yapay zeka ile ilgilenen yapay zeka mühendisi olur, bilgisayar mühendisi olur artık hangi yazılım mühendisi tam bilmiyorum o kısmını, onlara bu durumu aktarmayı, onlarla beraber iletişim halinde bu sorunları yapay zeka ile beraber çözmemiz gerektiğini ilerleyen süreçte düşünüyorum.

**G:** Sen daha öncesinde hiç bir yapay zeka aracı kullandın mı? Kullandıysan nasıl sonuçlar elde ettin?

**P01:** Şu an ChatGBT’yi kullandım. Şöyle sonuçlar oldu; hızlı bir şekilde verileri toplamamı, öz bilgilere ulaşmamı, gereksiz ayrıntılara takılmadığım, boğulmadığım bir veriler elde ettim. Kullandığımda bunlar oluyor.

**G:** Peki, sence yapay zeka kullanımının özel eğitim öğretmenine ne gibi katkıları olabilir?

**P01:** Yapay zeka kullanımı, öğretmeni gereksiz vakit harcamaktan, gereksiz çaba sarf etmesinden, yorulmasından bunlardan kurtaracağını düşünüyorum. Öğretmene bu faydaları olacağını düşünüyorum. Biraz daha somut örnek vermek gerekirse örneğin öğrencinin bir konuyu anlamasında yapay zekayla görselliği arttırdığımızı varsayalım. Bu öğretmen sürekli böyle görsel bir şeyler bulmakla çaba sarf edeceğine yapay zeka onu önüne hazır bir şekilde getirebilir. Veya öğretmen pek çeşitli şeyler bulamazken yapay zeka ona çeşitli şeyler verir. Öğretmen çeşitli kaynakları elde eder. Bu şekilde hem yorulmasından hem vaktinden birçok yönden faydalı olacağını ben düşünüyorum.

**G:** Peki, bu yapay zekanın hangi yetersizlik türlerinde daha etkili olacağını düşünüyorsun?

**P01:** Yapay zekanın zihin yetersizliğinde hafif, orta, ağır hepsinde… onun dışında otizm, öğrenme güçlüğü bu tarz durumlarda daha etkili olacağını düşünüyorum.

**G:** Neden sence onlarda daha etkili olur?

**P01:** Çünkü, işitme ve görmede mesela işitme sorununu birazcık çözebilirsek çocuğun artık ihtiyacı kalmıyor bizim özel eğitim ihtiyacımıza. Örnek veriyorum yapay zekayla çocuk sesleri çok algılayamıyordu. İşitme yersizliği var, sesleri algılayamıyor. Yapay zekayla bu sorunu çözdük. Sesleri algılayabiliyor artık öğretmenin sesini fark ediyor, anlıyor. Artık anladığı için bizim özel eğitim verme ihtiyacımıza pek ihtiyacı kalmıyor. O yüzden zihin yetersizliği, otizm bu tarz alanlarda daha ihtiyaç olduğunu düşünüyorum.

**G:** Peki bu yapay zeka sistemleri ya da araçlar, Özel eğitim mesela hizmetlerine, özel eğitim sisteminin içerisine nasıl entegre edilebilir sence?

**P01:** Öncelikle öğretmenlerin bunun bir farkına varması lazım. Ben ChatGBT’yi veya diğer yapay zekaları yeni öğrendim. Daha birkaç ay oluyor bunları fark edeli. Benim gibi birçok insan olduğunu düşünüyorum. Hani farkına varmadığı, hani bunlardan uzak yaşayan insanların. Öncelikle belki Milli Eğitim vasıtasıyla olur veya farklı durumlarla işte haberlerde.

Önce fark edilmesine, bunların bu hizmetleri bizim kullanabileceğimizi, önce fark etmek gerektiğini sonra onları ne gibi şeylerde kullanabileceğimizi öğretmenlere filan rehber olarak yönlendirmek gerektiğini, bu yönlendirmeden sonra öğretmenin zaten bunları öğrendikten sonra kendisi eksiklikleri fark ederek yapay zeka yardımı alarak eğitimi daha kaliteli yapacağını düşünüyorum.

**G:** Peki bu yapay zeka sistemlerinin, şimdi mevcut bir özel eğitim, yani ülkemizde müfredatı var, özel eğitim sistemi var. Bunun içerisine kolay bir şekilde entegre olabileceğini düşünüyor musun?

**P01:** Evet, çok kolay entegre olacağını düşünmüyorum. Çünkü her öğretmen farklı ve açıkçası bazı öğretmenlere bazı şeyleri kabullendirebilmenin yeniliklere açık olabilmenin biraz daha zor olduğunu düşünüyorum. Bizim bu öğretmenlerle de iletişim halinde olmamız ve bunları kabullendirebilmemiz lazım. Hem öğretmenlerin bu şekilde, insan bilmediği şeye biraz daha çekimser davranıyor. Yapay zeka da hayatımıza yeni geldiği için biraz daha öğretmenlerin çekimser davranabileceğini ve bildiği şeyden gitmenin daha kolay olabileceğini düşünüyorum, bu yüzden öğretmenlerin biraz daha zor entegre olacağını yapay zekanın hayatımıza düşünüyorum.

**G:** Nasıl kolaylaştırılabilir sence?

**P01:** Bence nasıl kolaylaştırılabilir, öğretmenler bu işi bilmediklerinden çekiniyorlar dedim ya bu bilmediği kısmını düzelterek. Yani öğretmenlere bu durumu öğreterek. Aslında çekinilecek bir şey olmadığını, böyle hayatımıza bir iş yükü olarak değil de tam tersi hayatımızı kolaylaştıracağını bizim iş yükümüzü hafifleteceğini bunları filan göz önünde bulundurarak göstererek, eğitim vererek daha kolay entegre olabilir.

**G:** Peki ilerde yapay zekanın özel eğitim öğretmeninin yerini alabileceği konusunda ne düşünüyorsun?

**P01:** Yapay zekanın çok kestirilebilecek bir şey olduğunu düşünmüyorum. Çünkü çok hızlı gelişebilen bir teknoloji. Ben geçmişte yapay zeka ile ilgili bazı şeyler okumuştum aslında biraz. Gördüğümde böyle geçmişte de gündem olup sonra gündemden düştüğü zamanlar olmuş yapay zekanın. Ama şu an tekrar gündemde ve gelişim aşamasında. Yapay zekanın nasıl gelişebileceğini ne kadar hızlı gelişeceğini ben kestiremiyorum. Hani belki çok hızlı bir şekilde logaritmik bir artış gösterip hayatımıza çok fazla yer edinecek ama bu belki tekrar gündemden de düşebilir yapay zeka. O yüzden ama şu an ki kanaatim kısacası yapay zekanın kısa vadede yani bir 20 sene içinde bir öğretmenin yerini alabileceğini düşünmüyorum.

**G:** Çok güzel. Peki bu yapay zekanın özel gereksinimli çocuğa sahip ebeveynlere, ailelere ne gibi katkıları olabilir sence?

**P01:** Yapay zekanın bilgi almasında, sonra velinin nasıl bir yol ilerlemesi gerektiğinde veya velinin de bize eğitim konusunda bazen bize destek olması gerekiyor. Bu eğitimlerde yapay zekadan destek alarak veliye yardımcı olabilir.

**G:** Peki, özel eğitim öğretmeni ve aile işbirliği konusunda yararlı olabilir mi sence? Mesela ne gibi faydaları olabilir? Öğretmen ve ailenin birlikte çalışmasını sağlayabilir mi?

**P01:** Aile ve öğretmene çeşitli dökümanlar sunarak şey yapabilir yapay zeka böyle farklı yollar gösterebilir veya iletişim halindeyken… ama iletişim halinde de çok yapay zekanın şu an bir şey yapabileceğini düşünmüyorum. Çünkü veli öğretmen kendisi iletişim kurduğunda bunun yapay zekanın pek araya gireceğini şu anlık düşünmüyorum. Kendileri bu işi çözeceğini, yapay zekaya çok ihtiyaç duyacaklarını düşünmüyorum bu iletişimde yani.

**G:** Peki bu özel gereksinimi olan çocukların, bireylerin günlük yaşamında yapay zeka neler yapabilir sence?

**P01:** Rehber olabilir, günlük yaşamında bazı sorunlarla karşılaştığında yapay zeka yardımcı olabilir bu yönde. Bir arkadaş bile olabilir yapay zeka. İhtiyaçlarını karşılamasında, ihtiyaçlarını dile getirmesinde… Örneğin, öğrenci ihtiyaçlarını biraz daha sözel ifade edemiyorken yapay zeka vasıtasıyla yapay zeka onu görsel bir şeylere çevirip mesela onun ihtiyaçlarını dile getirebilir mesela. Bu şekilde günlük zamanda ihtiyaçlarına yardımcı olacağını düşünüyorum. Yemek geldiğinde örneğin günlük ihtiyaç yemek geldi önüne, bu yemeği sevmediğini veya şu tarz yemekler sevdiğini o yemeğin resimlerini mesela yapay zeka yardımıyla ailesine gösterip bir günlük ihtiyacında yardımcı olabilir yapay zeka.

**G:** Peki şunu da soracağım sana, yapay zeka özel eğitim öğretmeni, öğrenci, aile konusunda ne gibi olumsuz etkileri olabilir sence?

**P01:** Şöyle olumsuz etkisi olabilir; özel eğitim öğretmenin yapay zeka günümüzde gerçekten hazır bilgiyi bir anda önümüze sunduğu için öğretmenin çok uğraşmadan sadece ondan elde ettiği verilerle daha fazla bir araştırmaya filan girişmeden sadece ondan elde ettiği şeylerle yetinebileceğini düşünüyor. Bu bir sorun çünkü öğretmenin belki yapay zeka faydalı başta da konuştuğumuz gibi zaman açısından emek açısından falan faydaları var ama sadece yapay zekayla da bazen yetinmemek gerektiğini düşünüyorum ben. Bu durumda öğretmen bunla yetinerek mesela farklı araştırmalara farklı şeylere yönelmeyebilir. Farklı bilgi öğrenmez. Öğretmen açısından bu. Öğrenci açısından düşünecek olursak sadece yapay zekaya yoğunlaşıp ona bağımlı olabilir diye düşünüyorum. Hani hayatını sadece yapay zeka vasıtasıyla çözebilen…

**G:** Yani bağımsız bir birey olmaz…

**P01:** Bağımsız bir birey olmaz sadece onunla çözebilen, o olmadığında sorunlarla karşılaşan, bir sorunu olduğunda sadece yapay zekaya başvuran bir birey karşımıza çıkabilir. Hani öğrenci açısından böyle. Aile açısından da biraz daha bu olabilir. Ama öğrenci açısından bu daha mümkün. Aile açısından yapay zekanın olumsuz yönleri ne olabilir, çocuğundan uzaklaşabilir. Nasıl yani? Sürekli yapay zekaya başvuran bir çocuğu olduğunda aile çocuğuyla iletişim kurmakta zorluk çekebilir, çocuğundan uzaklaşabilir yani. Onun dışında aile açısından yapay zekaya fazla önem verebilir. Yapay zeka neticede bence bir yere kadar olduğunu düşünen bir insanım. Bazen her şeyin yapay zekanın çözebileceğini düşünebilir. Hani kendisinin ihtiyacının azaldığını, günlük ihtiyacında yapay zeka yardımcı olduğunu öğrenciye varsayalım. Bu durumda aile kendine ihtiyaç hissetmez. Hissetmediği için aile artık çocuğunun ihtiyaçlarını karşılamada biraz daha geri planda durur. Benim düşüncem aile açısından ve çocuk açısında böyle bir sorun ortaya çıkabileceğini düşünüyorum.

**G:** Şimdi P01 peki bu özel gereksinimli çocukların eğitiminde yapay zekayı işin içine katarsak yapay zeka kullanımı gelecekte neler getirebileceğini düşünüyorsun? Nasıl şekillenecek o zaman sence gelecek?

**P01:** Gelecek çok muamma dediğim gibi yapay zekanın nasıl yönlendirebileceğini veya insanlar bunu nasıl kullanabileceğini bilmediğim için hani öncelikle bunu söyleyeyim, muamma. Geçmişte de hani buluşlar yapılıyor mesela insanlar bunu atom bombası olarak kullandı. Bunun gibi yapay zekanın da biz şu an olumlu yönlerini hep düşünüyoruz, hayatımıza şöyle faydası olur böyle faydası olur… ama bunun olumsuz yönlerini de kullanıp işin olumsuz yön tarafına da gidebileceği ihtimali var. Ama genel itibarıyla benim 20-30 sene içerisinde düşünürsek çünkü ben 50-60 seneyi kestiremiyorum inanın hocam. 50-60 sene sonra yapay zeka ne olur dediğinizde benim hiçbir fikrim yok ne kadar ilerleyeceği hakkında bir düşüncem yok. Ama tahminimce benim düşüncem 20-30 sene içinde yapay zekanın böyle bir hayatımızı kolaylaştıracağını hani daha hayatımızın içinde olacağını, bize yardımcı bir teknoloji olacağını bir çok yönden faydası olacağını düşünüyorum. Zaman, emek, para… bunların hepsi içinde.

**G:** Şimdi benim sana soracaklarım bunlardı. Senin yapay zeka ve özel eğitim konusunda eklemek istediklerin var mı?

**P01:** Yani genel itibarıyla şunu söyleyeyim, ben şu anlık yapay zekanın kullanılması, farkında olunması, insanların bunu fark etmesi ve hayatımızın içine girmesi gerektiğini düşünüyorum.

**G:** Bu çocuklar için de…

**P01:** Bu çocuklar için de tabi ki…

**G:** P01 teşekkür ederim, ağzına sağlık.

**P01:** Hocam ben teşekkür ederim.

**P02 — Interview Transcript**

**G:** Başlamadan önce sana kısaca anlatayım. Görüşmemizi ses kaydına alacağım. İsmin hiçbir yerde geçmeyecek, anonim olarak işlenecek her şey. Anonimleştirilmiş transkriptin makaleye yan dosya olarak konulup diğer araştırmacılarla da paylaşılabilir. Bunlara onam verir misin?

**P02:** Veriyorum hocam, bir sorun yok benim açımdan.

**G:** Çok iyi, başlayalım.

**P02:** Eğitim konusunda başta biz aslında bahsetmiştik. Yani yapay zeka çok geliştirilebilir Şu an dünyada. bu çocukların öğretimlerine ciddi manada kullanabileceğini düşünüyorum ve yararlı olacağını düşünüyorum. Mesela bizim yazdığımız proje matematikle ilgili bir projeydi ve yapay zekayı kullandığımız bir projeydi. Çocukların öğretimde de etkili olabileceğini düşündüğümüz için aslında yapay zekayı kullandık. Bir çok uygulama tasarlanabilir yapay zeka ile çocukların öğretimine dair.

**G:** Peki sen kendin daha önce hiç bir yapay zeka aracı kullandın mı? Böyle kullandıysan nasıl sonuçlar elde ettin?

**P02:** Mesela hocam, Snapchat'in AI kullandım. Şimdi Copilot’u daha çok kullanıyorum. ChatGPT kullanıyorduk geçen yıl daha çok. Copilot’un şu güzelliği var, bir soru sorduğunuzda size cevabı veriyor. Cevabı hangi sitelerden, nereden aldığına dair de bilgilendirme yapıyor. Ben daha sıklıkla şimdi Copilot’u kullanmaya başladım. Yani bir süredir onu kullanıyorum genelde daha sık olarak. Çünkü Snapchat falan daha yüzeysel cevap veriyor Snapchat’in AI’ı. ChatGPT biraz daha bilimsel. Copilot hem bilimsel cevap veriyor, hem uygulanabilirliği yüksek hem de nereden hangi siteden aldığı da belli olduğu için benim işimi kolaylaştırıyor.

**G:** Peki bu yapay zeka kullanımın özel eğitim öğretmenine ne gibi katkıları olabilir sence?

**P02:** Öğretmene ne gibi katkıları olabilir? Hocam biz şimdi okulda öğretim yapıyoruz ama hep şey diyoruz işte akıcılıkta, genellemede hep sıkıntı yaşadığımızı söylüyoruz. Çünkü normal hayata uyarlayıp uyarlamadığını bilemiyoruz. Dışarda takip, gözlem yapamıyoruz ve aileler de birçok konuda yetersiz kaldığı için aile de bilmiyor. Ama yapay zekanın geliştirdiği uygulamalarla biz bu çocuğa evde de eğitime devam edebiliriz ve çocuğun hem akıcılığını arttırmış oluruz hem genelleme yapmasını kolaylaştırabiliriz diye düşünüyorum.

**G:** Peki bu yapay zeka sistemlerinin kullanımı sence hangi yetersizlik türlerinde daha etkili olabilir?

**P02:** Hocam otizm, zihin yetersizliği, gerçi şimdi görmede de geliştirilebiliyor ama daha sık bence hafif zihinde özellikle daha çok kullanılabileceğini düşünüyorum. Otizmde kullanılabileceğini düşünüyorum işte diskalkuli gibi yetersizliklerde kullanılabileceğini düşünüyorum.

**G:** Peki nasıl kullanılabilir?

**P02:** Hocam ilk aşamadan başlayacak olursak biz tanı koymada güçlük yaşıyor muyuz? Evet yaşıyoruz. Bizim ülkemiz yine bu konuda biraz daha iyi gelişiyor. Ama mesela Hollanda'da 8 yaş öncesi tanı koymak istemiyormuş. Yapay zeka, sanırım Korelilerdi bulanlar, tanı koymaya başlamış diye biliyorum. Yani tanıyı ilk aşamada, tanı koymayı halledersek bence zaten sonra eğitime başlama yaşını düşürmüş oluruz. Bu da gelecekte çocuğun bağımsızlığını arttırmak için bizim işimizi kolaylaştırır. Sonra öğretimlerinde birçok uygulama kullanabiliriz ve az önce bahsettiğim gibi hem öğretmenin işi kolaylaşacak, hem ailenin hem çocuğun genellemeyi arttırmış olacağız evde de eğitimle. Birçok alanda kullanmış olacağız böylelikle yapay zekayı.

**G:** Peki şu andaki mevcut özel eğitim sistemlerinin içerisine nasıl entegre edilebilir sence?

**P02:** Yani teknolojinin gelişmesiyle hocam, okullarda mesela akıllı tahtalardan öğretmenler dersler işliyorlar, işte oraya entegre edip uygulamalarla devam edebiliriz. Bir de mesela kavram öğretiminde falan biz zorlanıyoruz otizmli çocuklarda. Yapay zeka bu noktada yeni uygulamalarla kavram öğretimi işini kolaylaştırıyor aslında. Burada kullanabiliriz işte çocuklarda matematik öğretiminde kullanacağız. Akademik beceriler de kullanabiliriz. Sosyal becerilerde de yine kullanabileceğimizi düşünüyorum ama nasılını tam bilmiyorum hocam.

**G:** Peki? Sence kolay ve işlevsel olur mu? Böyle bir entegrasyon olsa.

**P02:** Kesinlikle. Öğretmenin işini ben çok kolaylaştıracağını düşünüyorum. Ülke de şöyle bir zorluk yaşayabiliriz. Biz teknolojiyi bir tık daha geriden takip ediyoruz. Japonya'ya göre, Kore'ye göre, ülke açısından biraz zorluk yaşayabiliriz ama bizim işimizi kolaylaştırması açısından bizim çok işimize yarar yani.

**G:** Peki, bu yapay zekanın ileride bir özel eğitim öğretmenini yerini alabileceğini düşünüyor musun?

**P02:** Akademik becerilerde evet yerini alabileceğini düşünüyorum. Ama sosyal beceriler hususunda tam olarak bir öğretmenin yerini tutacağını sanmıyorum.

**G:** Anlıyorum. Peki bir de işin içinde aile var tabii. Ailelere ne tür katkıda bulunabilir yapay zeka? Özel gereksinimli çocukların ebeveynlerine?

**P02:** Mesela bizim aile eğitimi dersimiz var ama aileye eğitim verebilmemiz için aile ile ortak saatler belirlememiz lazım. Bir program belirlememiz lazım ki ona göre aileyi çağırabilelim. Ama eğer yapay zeka ile eğitim yapabilirsek, böyle uygulamalar geliştirebilirsek aile her an her istediğinde buna kolayca ulaşabilir. Bu da çocuğa uygulayacağımız yani ailenin çocuğa vereceği eğitimi kolaylaştırmış olur. Ailenin öğretimini kolaylaştırmış olur ve her an elinin altında olur böylelikle.

**G:** Peki bu yine yapay zeka, özel eğitimde aile öğretmen işbirliği ne gibi katkılar sunabilir?

**P02:** Aile öğretmen işbirliğine? Yani nasıl olabilir? Bu konuda bir fikrim yok hocam yani.

**G:** Peki özel gereksinimli bireylerin günlük yaşamlarına ne gibi katkıları olabilir sence? Yapay zekanın?

**P02:** Hocam günlük yaşama nasıl katkısı olur? Mesela yine derste bahsetmiştik bu beceri öğretimini yapay zeka ile yapabilirsek diye. Biz niye beceri öğretiyoruz çocuğa? Çocuğun bağımsızlığı artsın diye. Dışarıda dikkat çekmesin diye. Mesela basit bir alışveriş yapacağı zaman kendi başına yapabilsin diye. Eğer yapay zeka bu öğretimleri yaparsa bu çocuk dışarda dışlanma olasılığını azaltmış olur, akranlarının arasında dışlanma olasılığını azaltmış oluruz. Bu da zaten bizim istediğimiz şey. Bağımsızlığını kazanmasını istiyoruz biz bu çocuğun. Yani çocuğu özgürleştirmek açısından işte aileye bağımlılığını azaltmak, öğretmene bağımlılığını azaltmak, akranları arasındaki kabulü artırmak için bence etkili olabilir.

**G:** Peki yapay zeka; özel eğitim öğretmeni, özel gereksinimli öğrenci, ebeveyn açısından düşünecek olursak ne gibi olumsuz tarafları olabilir?

**P02:** Öğretmenlerin işini elinden alıyor hocam.Yani gelecekteki kısmı düşünecek olursak, öğretmen açısından düşünecek olursak. Bahsettiğimiz gibi eğer bir öğretmenin yerini tutabilecek ise özel eğitim öğretmenliğini yok edecek bir nevi. Yani aile açısından ne gibi olumsuzluk olabilir? Yani aile açısından da değineyim ve çocuk açısından da değineyim. Mesela otizmli çocuklara çok ekran göstermek istemiyoruz. Ekran maruziyetini azaltmak istiyoruz. Zararlı olduğunu düşünüyoruz. Ama yapay zekalı uygulamalar da çocuk ekrana maruz kalmak zorunda. Yani ekranla bir şey yapmak zorunda. Yine bu tabi ki yani bu zararlı etkeni ortadan kaldırmak için biz ekrana maruz kalmasını istemiyoruz. Çocuk tek taraflı şey yapmasın yani orayı dinleyip put gibi orada durmasını istemiyoruz. Biz etkileşim halinde olsun istiyoruz. Çocuğun dil gelişimi artsın istiyoruz. Yapay zekayla bunu sağlayabilir miyiz? Evet. Bu zararlı kısmını ekranın, zararlı kısmını ortadan yine kaldırabiliriz. Aile açısından ne gibi bir zararı olabilir? Aile açısından bence bir zararı yok. Ben daha çok yararlı kısmına odaklanıyorum açıkçası.

**G:** Peki özel gereksinimli çocukların eğitiminde yapay zeka gelecekte nasıl kullanılabilir? Yani eğitimi nasıl şekillendirebilir sence önümüzdeki yıllarda?

**P02:** Bence olumlu etkisi olacaktır. Şimdi bile mesela birçok uygulama var. Akademik becerilerini artıracak uygulamalar var. Bunlar zaten yavaş yavaş bizim ülkemizde de kullanılmaya başlandı. Soruyu bir daha sorar mısınız?

**G:** Yapay zeka özel eğitimin geleceğini nasıl şekillendirebilir sence?

**P02:** Nasıl şekillendirebilir? Yani olumlu yönde olacağını düşünüyorum. Ama işte yeni, farklı bir şeyler ekleyebilir mi şu an bizim bilmediğimiz? Evet, ekleyebilir çünkü. Eksik kaldığımız zaten birçok alan var Özel eğitimde. Başaramadığımız birçok şey var. Mesela çocukların Stereotipik davranışlarını çok şey yapamıyoruz hani baskılayamıyoruz. Azaltmada güçlük yaşıyoruz. Belki yapay zeka buna dair yeni bir şeyler üretebilir. Yapay zekalar üretilebilir yani. Akademik beceriler de zaten olumlu yönde bir başarıya ulaştıracağını düşünüyoruz. Öyle.

**G:** Benim sana sormak istediklerim bunlardı. Özel eğitim öğretmeni, yapay zeka, özel gereksinimli çocuk... Senin eklemek istediklerin var mı?

**P02:** Yani yine derste bahsettiğimiz gibi bir etik uygulanabilirliği biraz sıkıntı benim için bana göre. Bir de sonuçta yapay zeka, mesela robotlar ürettiğimizi farz edelim diye konuşmuştuk. Bu robotlarda enerjiyi neyle karşılayacağız? Bu konuyu düşünüyorum. Hani bu kadar enerjiyi nasıl üreteceğiz onu düşünüyorum. Onun dışında birçok hususta ben yararlı olabileceğini düşünüyorum.

**G:** Tamam o zaman teşekkür ederim P02, güzel anlattın.

**P03 — Interview Transcript**

**G:** Görüşmeye başlamadan önce kısaca söyleyeyim. Sakin ol, bir sınav değil bu. Soracağım soruları sesli yanıtlayacaksın, ben de kayda alacağım. Verdiğin yanıtlar anonim olacak, ismin geçmeyecek. Anonim metin makalenin ek dosyası olarak diğer araştırmacılara da açık şekilde paylaşılabilir. Buna onam veriyor musun?

**P03:** Veriyorum hocam.

**G:** Tamam, ilk soruyla başlıyoruz.

**G:** … ne düşünüyorsun?

**P03:** Şöyle hocam; hem öğrencinin, hem öğretmen hem de ailenin işini kolaylaştıracağını düşünüyorum. Vakitten tasarruf, daha doğru verilere ulaşmayı sağlar. Onun dışında… O şekilde şu anda aklıma gelemedi ama…

**G:** Ben soracağım, Merak etme sen rahat ol. Peki sen daha önce hiçbir yapay zeka aracı kullandın mı? Kullandıysan nasıldı? Nasıl sonuçlar elde ettin?

**P03:** Şöyle daha önce hiç kullanmadım hocam, ben yapay zekayı sizin dersteki ödevinizle kullanmaya başladım. Hani cevapları? Şöyle bir soru sormuştum bana güncel cevaplar hakkında çok bir fikrim yok demişti. Daha hani bilindik şeyleri söylüyor. Mesela sayısal verilerle çok ilgisi yoktu. Sayısal bir şey sormuştum ben.

**G:** Şimdi sen ilerde özel eğitim öğretmeni olmayı düşünüyorsun. Peki yapay zeka özel eğitim öğretmenlerine ne gibi katkılar sunabilir sence?

**P03:** Hocam şöyle… veri toplamada daha şey olur böyle. Daha doğru cevaplar verir. Kayıt edilebilirliği fazla. Onun dışında…hocam kusura bakmayın streste girdim.

**G:** Arkadaşınla muhabbet ediyormuş gibi düşün, rahat ol.

**P03:** Hocam. Öyle olmuyor işte. Bir daha sorar mısınız hocam?

**G:** Şimdi özel eğitim öğretmenlerine ne gibi katkıları olabilir yapay zekanın?

**P03:** Hocam özel eğitim öğretmenin işini kolaylaştırır, öğrenciyle iletişimini artırabilir. Şöyle bir öğretmen öğrenci iletişime geçmek istemeyebilir. Ama yapay zeka sayesine öğrenciyle iletişimi daha da kuvvetlenebilir diye düşünüyorum.

**G:** Peki sen şimdi aynı zamanda fizyoterapistlik sen, bu yapay zekanın kullanımı hangi yetersizlik türlerinde daha etkili olabilir sence?

**P03:** Şöyle düşünüyorum hocam, etkisi olacağı bir yetersizlik yok gibi. Fiziksel olarak düşündüğümde, bu hareket sensörlerinin yapay zekaya verdiği sinyallerle öğrencinin hareketindeki problemleri daha somut bir şekilde bildirir. Onun dışında zihinsel öğrencilerde de sosyal becerilerini geliştirebilir. Günlük yapması gereken ritüelleri takip edip, analiz edip dönüş sağlayabilir. Dönütler daha sağlıklı olur. Hani özel öğrenme güçlüğüne gelecek olursam öğrencinin nasıl deyim… Hangi alanda eksiği var ya da öğretim kısmında hangi alanlarda daha verimli oluyor bunu analiz edip ona göre bir dönüt sağlayabilir öğrenciye. Böyle.

**G:** Çok güzel. Peki bu yapay zeka özel eğitim hizmetlerini yani bu mevcut olan özel eğitim sistemine sence nasıl entegre edilebilir?

**P03:** Hocam bunun için çeşitli uygulamalar üretmekte fayda var diye düşünüyorum. Bunun için, işin içine aileyi de katmamız lazım. Hani öğrenci ve öğretmen arasında olabilecek bir şey değil. Bunu evde de devam ettirmeleri lazım. Tabii ki bu devlet kontrolü dışında olursa çoğu aile hani öğrenciyle çok ilgilenmiyor hocam işin açıkçası, devletin bunu gözetmesi lazım. Öğretmenlerin bunu üst mevkilere geri bildirmesi lazım. Yani buna bir ceza uygulanabilir mi? Yapılmamasına uygulanamaz ama. Bir hediye şeklinde ya da aileyi motive edici şeylerle uygulanabilir diye düşünüyorum.

**G:** Peki sence bu özel eğitim içerisinde yapay zekanın kullanımı işlevsel olur mu? Kolay olur mu? Ya mevcut şeyi düşün. Yani özel eğitim sınıflarında ders işleyen öğretmeni düşün yani onun için işlevsel olur mu? Ne gibi kolaylıklar sağlar?

**P03:** Ne gibi kolaylıklar sağlar hocam? Öğretmenin ufkunu genişletir diyebilirim. Hani tamam uzun zamandır çalışan bir öğretmen hani etkinliktir vesairedir çok problem yaşamaz ama hani yeni mezun birisi illaki problemler yaşar, onların ufkunu genişletir. Böyle daha çok materyal, daha çok etkinlik öğrencinin yararına olan daha fazla şey üretir. Ben işlevsel olacağını düşünüyorum.

**G:** Peki ileride, önümüzdeki yıllarda bu yapay zekanın özel eğitim öğretmeninin yerini alabilme olasılığı konusunda ne düşünüyorsun?

**P03:** Hocam bence özel eğitim öğretmeninin yerini alamaz. Çünkü biz duygusal bir bağ kuruyoruz. O an öğrencinin tepkisine göre tepki veriyoruz. Hani yapay zeka bunları yerine getiremez. Tamam öğretmenin işini kolaylaştırır ama yerini alır mı? Bence alamaz.

**G:** Peki bu yapay zekanın özel gereksinimli çocuğa sahip aileye ne tür katkıları olabilir ebeveynlere?

**P03:** Aileye ne tür katkısı olur?

**G:** Bu bir sınav değil P03. Rahat ol sen kendi düşüncelerini, böyle çok uçuk da olabilir düşüncelerin, bazı insanlara göre çok hayalperest de diyebilirler rahatça söyle…

**P03:** Hocam şöyle katkısı olur aileye. Özel gereksinimli bir çocukla ilgilenmek gerçekten çok zor. Ailelerin de eğitim seviyesi buna yetişmeyebilir. Ben ilçedeydim hocam hani şey ailelerde öğrenci seviyesindeydi açıkçası. Akraba evliliği vesaire çok fazlaydı. Hani kendisine yetersiz olan bir insan çocuğa ne kadar yeterli olabilir? Düşünülür. Hani bu uygulamalarla ama hani nasıl? İlla ki şey… Öğrenciye daha faydalı olur. Yani tam anlatamadım ama. Hocam aile yetersiz kalıyor.

**G:** Ailen yeterliliklerini mi artırabiliyor?

**P03:** Evet evet. Öğrenciyi takip etmesini kolaylaştırır. Mesela önceki seviyesi ile sonraki seviyesini kaydedebilirse aile daha fazla teşvik olur o uygulamayı kullanmaya. Yani belki kendisi de kullanır.

**G:** Peki bu yapay zekanın özel eğitim öğretmeni-aile işbirliği konusunda ne gibi katkıları olabilir sence? Öğretmen aile…

**P03:** Öğretmen aile işbirliği…

**G:** Kolaylaştırabilir mi mesela? Nasıl?

**P03:** Kolaylaştırır tabi ki hocam. Dediğim gibi çocuğun durumunu aile takip edemiyor ama bu uygulamalar sayesinde takip edebiliyor. Ya da aile ne yapacağını biliyor. Uygulamalar sayesine aile hani yapılması gerekenleri oradan öğrenip çocuğa yansıtabilir. Yani. Kısa kısa oluyor ama…

**G:** Olsun sorun değil. Peki bu özel gereksinimli çocukların günlük yaşamlarına ne gibi katkısı olabilir yapay zekanın?

**P03:** Günlük ritüelleri var bu çocukların. Bazılarını biliyorlar, bazılarını bilmiyorlar, es geçiyorlar. Bunları takip edebilir, günlük yapması gerekenleri bir çizelge halinde gösterip çocuklara bunları yapabilir. Bunu görsel, işitsel şekilde destekleyebilir. Öğrencinin onları takip ederek neyi yapması gerektiğini, hangi zamanda yapması gerektiğini, nasıl doğru, nasıl yani çok güzel yapması gerektiğini oradan öğrenebilir. Çünkü mesela diş fırçalamak basit bir şey gibi gelebilir ama bunu çocuğun sabah, öğle, akşam yapması gerektiğini bilmesi lazım. Çoğu aile de bunu bilmiyor ve bu şekilde öğretemiyor. Ama bunu uygulamalar sayesinde yapabilirler.

**G:** Çok güzel. Peki bu yapay zekanın özel eğitim öğretmeni, aile ile öğrenci konusunda ne gibi olumsuz tarafları olabilir sence?

**P03:** Şöyle hocam. Veri gizliliğinden emin değilim. Bu yapay zeka çocuğun hayatında olunca ister istemez ev ortamına da girecek, okul ortamına da girecek. Bu veriler gizlilik içinde saklanıyor mu onu bilemiyorum. Onun dışında çocuğun bence sosyalleşmesini de sağlar. Çocuk başka biriyle iletişim, iletişime geçmeye korkabilir ama sonrasında bu yapay zekayla sohbet ederek kendini açabilir. Aile için olumsuz bir tarafı var mı diye düşünüyorum. Yani maliyeti olabilir ama buna da dediğim gibi devlet destek vermeli.

**G:** Peki. Yapay zekanın sence gelecekte özel eğitimi nasıl şekillendirebileceğini düşünüyorsun. Nasıl şekillendirebilir?

**P03:** Hocam şöyle. Bence ileride çok kullanışlı ve işlevli olacak. Yani kullanmam diyen insanlar da kullanmak zorunda kalacak. Çünkü zamandan tasarruf ediyor. Daha sağlıklı veriler sunuyor. Öyle olunca insanlar kullanmak zorunda kalacak. Şu anki gibi değil yani, olmazsa olmaz bir parça olacak. Hani öncesinde biri deseydi böyle. Hani telefon gibi bir alet olacak. Herkesin hayatında olacak. İnsanlar çok komik karşılayabilirdi ama şu anda kimse hani elinde telefon olmayan yok. Yapay zeka da bu şekilde olacak ve özel gereksinimli öğrencilerin hayatına bence büyük oranda yerleşecek.

**G:** Şimdi benim sana yapay zeka, özel eğitim, özel gereksinimli çocuk konusunda sormak istediklerim bunlardı. Senin eklemek istediklerin var mı? Şöyle de olsa böyle de bir şey olabilir diye aklına gelen…

**P03:** Şu anda yok gibi.

**G:** Bu konuda anlatmak istediğim bir anın var mı? Bir fikrin var mı, paylaşmak istediğin?

**P03:** Hocam siz ödev verdiğinizde ben bir proje tasarlamıştım. Çok güzel gibi gelmişti ama sonrasında maliyetli gelmişti. Böyle bir küçük ev robotu gibi özel gereksinimli çocuğun evinde oluyordu hocam. Hani sonrasında onu yapması gerekenleri falan duvara yansıtıyordu. Çok mantıklıydı ama benim düşüncem o şekilde ama çok maliyetli olduğu için ben söylememiştim. Söyleyebileceğim bunlar sadece.

**G:** P03 teşekkür ederim.

**P03:** Ben teşekkür ederim hocam.

**P04 — Interview Transcript**

**G:** Başlamadan kısa bir bilgi vereyim. Görüşmemiz boyunca ses kaydı alacağım. Söylediklerin anonim olarak ele alınacak, hiçbir kişisel bilgin yayında yer almayacak. Anonimleştirilmiş hâliyle transkriptin yan dosya olarak makaleyle birlikte açık şekilde paylaşılabilir. Bütün bunlara onam veriyor musun?

**P04:** Evet, veriyorum hocam.

**G:** Teşekkür ederim, başlıyoruz.

**P04:** Hocam öğretmenlere mesela bu süreçte zaman tasarrufu sağlayabilir, işgücü tasarrufu sağlayabilir. Dezavantajları da avantajlarına gelecek olursak. Gelecekte daha da çok faydalanabileceği bir kıvama geleceği kesin. Dezavantajları kısmı da var illaki ama. Şimdilik daha avantajlarına geçemedik. Yani tam anlamıyla kullanılmadığı için.

**G:** Peki sen daha önce herhangi bir yapay zeka aracı kullandın mı? Kullandıysan neler yaptın?

**P04:** Kullandım. Soru sordun yani Snapchat'teki AI’ya. Kendim için hem özel eğitimle alakalı hem başka konularda. Mesela dolandırılma konusunda bile nereye başvurmam konusunda ya da nasıl yol izleyebileceğim konusunda fikir aldım. Hesabımdan para çekme konusunda o konuda bile yardımcı oldu.

**G:** Peki yapay zeka kullanımının özel eğitim öğretmenlerine ne tür katkıları olabilir sence?

**P04:** Az önce söylediğim gibi eğitim öğretmenlerine katkısı ne olabilir? Dediğim gibi zaman tasarrufu kesinlikle sağlar. Öğretmenin iş gücü azalır ama bir yerde öğretmenin önüne de geçebilir yani. Yani belki yarın öbür gün eğitimde hem bir yapay zeka robotik, birisi hem bir özel eğitim öğretmeni yan yana çalıştığı zaman alanına da girebilir. Ama iş gücünü azaltacağı kesin de ama alanına da girebilir. Dezavantajları da olabilir. Gizliliği ihlal edebilir. Çünkü öğrencilerin videosu, fotoğrafı bunları eğer kaydediyorsa ki kaydetme ihtimali de var. Bilemeyiz, güvenemeyiz nasıl yönetildiğini. Bu tarz şeyler olabilir. Ya iletişim konusunda görme yetersizliği ya da işte…

**G:** Hangi yetersizlik türlerinde daha etkili olur sence?

**P04:** İletişim ve öğrenme güçlüğünde daha çok kullanılacağını düşünüyorum. İletişim yetersizliği ve öğrenme durumu daha günlük yaşamda karşılarına çıktığı için yani o konularda daha iyi olabileceğini düşünüyorum. Ne gibi olabilir? Hani bazı veri tabanlı sistemler oluyor ya görme yetersizliği olan bireylerde mesela onlara şey sağlayabilir hocam, sesli asistan gibi. Yani daha çok duyusal yetersizlikleri daha çok işe yarar diye düşünüyorum. Hani bi zihin, otizmden çok işitme görme gibi ya da öğrenme konusunda yetersizlik.

**G:** Peki, bu özel eğitim hizmetleri var şu anda sunula. Bu özel eğitim hizmetlerine nasıl entegre edilebilir yapay zeka?

**P04:** Yani öncelikle kurallı ve sistematik olması lazım. Entegre nasıl edilir? Üzerine çok fazla düşünmedim ama yani eğer entegre edildiğinde belli bir kurala, belli bir sisteme uygun olmazsa avantaj sağlayacakken dezavantaja gidebilir diye düşünüyorum. Nasıl entegre edilir bunu zaman gösterecek. Neler yapılabilir? Yani şu aşamaya gelirse, robotik aşamaya gelirsek zaten kolay entegre edilir. Ama o aşamaya gelmeden mesela kullanılan şeyler hala kağıt üzerinde. Hala bilgisayar ortamına bile geçmeyen şeyler var. Hani veri toplama düzenleri var mesela. Daha bunu bile aşamayan gelenekselci öğretmenler olduğu için öncelikle bu konuda mesela öğretmenlerin de ya da o sistemin içerisindeki herkesin eğitilmesi gerekiyor. Yani daha kayıtları bile bilgisayar ortamına dökemeyeceksek hala kağıt üzerinden ilerleyeceksek o zaten sıkıntı. O yüzden gelenekselciği bırakmamız gerekiyor. Ama bunun için inat edecek bir şey de var, topluluk da vardır. Yani önce bunu kabullenmek lazım bence.

**G:** O zaman bunun yani işlevsel olarak kullanılması kolay olmayacak.

**P04:** Evet, insanları ikna etmek bu durumu…

**G:** Nasıl kolaylaştırabilir peki?

**P04:** Şey yaparak, hani eğitim vererek, açıklayarak. Direkt yani bir öğretmen özellikle geleneksel, özellikle eski bir öğretmene böyle olacak şöyle olacak... Yani bilgisayar açmayı bilmeyen öğretmen var. Belki şu an hala yoktur. Yani daha çok gençtir belki ama bilgisayarı açmak biraz hani şey oldu da, açmayı bilmeyen çok uç bir örnek oldu ama. Yani ben işimi kağıt üzerinden yaparım, bilgisayarla işim olmaz, verileri kağıda yazarım gibi düşünenler oldu ama bu konuda tam anlamıyla bir eğitim avantajı. Dezavantajı konusunda yapay zeka anlamında öğretmene şunu uyandırmak lazım. Yapay zeka senin işini kolaylaştıracak, zorlaştırmayacak. Ama korkan öğretmenler illa ki vardır. Yani bence.

**G:** Çok güzel. Peki bu ileride yapay zekanın özel eğitim öğretmeni yerini alabileceğini düşünüyor musun?

**P04:** Çok ilerde olabilir bu. Yani yakın bir tarihte beş on yıllık bir süreç olamaz. Ama ileride sadece özel eğitim öğretmenin değil, çoğu şeyin yerini alabilir. Yani garsonlar robot olabilir. Yani özel eğitmenlerin yerini nasıl alır? Alabilir de. Yani gerçekten öğretmenlik açısından en zor olacağı bence yapay zekanın, robotların, öğretmenin yani en zor alabileceği meslek gruplarından bir tanesi bence öğretmenliktir. Yani robot garson olabilir daha kolay olur. Yani tabağını kaldır götür o daha kolay işlenebilir. Ama spesifik olarak bir branş öğretmeninin yerine geçebilmesi için baya donanımlı olması lazım onu kabul ettirmek için.

**G:** Özel eğitim öğretmeni en son yapılacak iş midir?

**P04:** Yani en son öğretmenlik açısından özel eğitim öğretmenliği en son ama meslek grupları açısından öğretmenlik en son onu bilemeyeceğim. Çünkü mühendislik söz konusu. Ne bileyim genetik çok… gerçi genetik falan da bence daha kolay olabilir. Öğretmenlik daha farklı, biraz duyguları da olan bir şey olduğu için yani sadece bir şeyler yüklemek de yetmeyebilir. Tabi duyguları olacaksa robotların, o ayrı bir konu.

**G:** Peki yapay zekanın bu özel gereksinimli çocuğa sahip olan ailelere ne gibi katkıları olabilir sence?

**P04:** Ailelere şöyle katkısı olur; aile sistematik ilerlerse eğer ailede öncelikle mesela aile karşısında muhatap bulamadığı dönemlerde öğretmen olarak işte bazen maddi sebeplerden hani robottan yararlanmak tabi ki maddiyat ister de o ileriki boyut ama şu an bile çoğu özel gereksinimli öğrencinin, bireyin ailesi yapay zekadan haberi olmayan çok fazladır yani oran olarak. Bilseler mesela normal günlük sormak istedikleri soruyu arayıp da rehabilitasyon merkezine soramadığı bir soruyu yapay zekaya sorabilir. Yani şu an için çok basit ilk aşamada bu. Şu an elinin altında böyle bir işlem ama bilemiyor. Ama ileri düzeye de robotikte o zamanda maddi gücü yine her zaman öne geçirir. Maddi gücü iyi olan bir aile kendisine gölge öğretmen gibi bir robot, her an yanında olan bi robot tayin eder yani satın alır çocuğu için. Hem kendi yaşam kalitesini arttırır, kendisine zaman konusunda yani tasarruf sağlar hem de öğrenci için müthiş olur.

**G:** Çok güzel. Peki bu özel gereksinimli çocukların, yetişkinlerin günlük yaşamlarına ne gibi katkıları olabilir sence yapay zekanın?

**P04:** Günlük yaşamlarına da şöyle, bununla alakalı direkt aklıma bir şey gelmedi. Ben de sormayı tercih ettim yani yapay zekadan faydalandım. Günlük yaşamında en çok karşıma yetersizlik grubu olarak görme yetersizliği olanlara en basit örneğiyle onlara yardımcı olabileceğini söyledi. Dediğim gibi hani bu hareket becerilerine kazanmamışlar üzerine sesli asistanlarla ya da dediğim gibi yine duyuşsal yetersizliği olanlara daha çok faydası olacağını düşündüm günlük yaşamında ama tabi ki zihinde otizme de günlük yaşamında faydası olacaktır. Dediğim gibi belki gölge öğretmen ileride olursa günlük yaşam becerilerine fayda da bulunabilir. Yanında sürekli olursa. Ya da dediğim gibi ailesine, kendisine.

**G:** Peki sence ebeveyn sonrasında öğrenci, öğretmen konusunda yapay zekanın ne gibi olumsuz etkileri olabilir?

**P04:** Olumsuz tarafları, dezavantajları şöyle olabilir. Eğer hani bu bir kayıt tekniği olacaksa yani öğretmen kendi karakteri gereği daha duygularıyla ya da işte işine saygısıyla daha nasıl dyiyim, doğru davranırken yapay zeka yani bu kayıt teknikleri daha ileriki zamanlarda robotik olaylarda, durumlarda gizliliği ihlal edebilir. Çünkü ses kaydını alır, alacak yani videoya çekecek. Ki yarın öbür gün mesela onu başka bir yerde farklı bir şekilde kullanabilir. Ailenin isteği doğrultusunda bir şeyler yapma ihtimali yüksek.

**G:** Öğretmene ne gibi olumsuz tarafı olabilir sence ?

**P04:** Öğretmenin de öğretmen bundan faydalanacağım derken yani böyle bir avantaj sağlayacak derken öğretmenin de yani ihlal edebilir. Yani öğretmenin de dezavantajı şu olacak öğretmene ihtiyaç olmayacak belli bir yerden sonra. Direkt işini yapamayacak yani. Belki robotlar daha çoğaldığında bir de maddi açıdan çok da zorlanan olmayacak kıvama geldiğinde onları daha şey görebilecekler yani bi robot mu yani teknolojik alet mi daha iyi olabilir bir insan mı, denildiğinde belki robotları tercih edecekler. Tercih edilmeyecek öğretmen belki de. Ama tercih edilip birlikte kullandılığı dönemlerde nasıl dezavantajı olabilir? Dediğim gibi alanına girebilir. Yani ve onu yönetemez belki. Hem robotu yönetecek belki de yönetmek zorunda kalacak. Onunla birlikte iş birliği yapmak zorunda kalacak yani kendinin yanında. Canlı, kanlı olmayan birisini de yönetmek durumunda kalacak. Onu da kontrol etmek durumunda kalacak. Belki de o, onu kontrol edecek. Farkında olmadan. Yani öyle. Bir de öğrenciyi, eğitim vermeye çalışacak. Zorlanabilir.

**G:** Peki bu özel eğitimi, ileride yapay zekanın nasıl şekillendirebileceğini düşünüyorsun? Nasıl şekillendirir?

**P04:** Yani olumlu yönde şekillendirir bence. Bence hani (?…) olamaz belki ama hani bilemedim hocam. Yani yine dediğimiz gibi, söylediklerim gibidir diğer meslek grupları kadar olmasa da öğretmenliği ele alabilecek kıvama gelir. O durumda şekillendirir. Bir de işi kolaylaştırır. Verileri daha netleştirir. Havada kalmaz bazı şeyler. Ailelerle, ebeveynle, çocukla işbirliği daha çok artabilir. Yani tam olarak böyle düşünüyorum.

**G:** Peki senin bu yapay zeka konusunda anlatmak istediğin bir anın, bir fikrin var mı söylemek istediğin?

**P04:** Yani ekstra bir anım olmadı ama yani yapay zekayı daha önceden sizinle olan dersimizden önce, yapay zekayla çok önceden tanışıp kullanmıştım. Normalde kullanmıyordum. İşte hani ama derslerle alakalı kullanmaya başladıktan sonra şimdi yani dışarı çıkıp yemek yemek istediğim zaman bile yapay zekayla tantuni hakkında konuşabiliyoruz, o kıvama geldik. Hemen bana en yakındaki tantuni dükkanlarını, kendisinin bu konudaki deneyimlerini, tantuni içinde neler olabileceğini, lezzetli bir yemek olup olmadığını ya da işte yakınlarda yanlışlıkla üye olduğum bir sistem üzerinden sürekli hesabımdan para çekiliyor. Aklıma direkt şey geldi hani Google’dan araştırmak yerine zaten dolandırıcı olduğunu biliyorum, indirdiğim şeyin. Silsen de para çekmeye devam ediyor. Bir yeri aramak yerine önce yapay zekaya sormak geldi aklıma. Sordum, durumu anlattım. O da bana gidilecek yeri, emniyeti, bankayla iletişime geçmemi, banka önce aklıma gelmemişti. Bir işe yaramayacağını düşündüm. Hani bu şekilde işimi çözmeme yardımcı oldu.

**G:** Çok güzel. Benim sana sormak istediklerim bunları. Senin eklemek istediklerin var mı bu konuda?

**P04:** Bu kadar hocam eklemek istediklerim.

**G:** O zaman teşekkür ederim P04.

**P04:** Ben teşekkür ederim.

**P05 — Interview Transcript**

**G:** Sana hemen söyleyeyim, görüşmemiz kayıt altına alınıyor. Söylediklerin anonim, isim yok, kurum yok. Anonimleştirilmiş transkriptin daha sonra başka araştırmacıların kullanımına açık olarak paylaşılabilir. Onam veriyor musun bunlara?

**P05:** Veriyorum hocam.

**G:** Güzel, ilk sorudan başlayalım.

**G:** Sen daha önce hiç yapay zeka kullandın mı? Kullandıysan ne gibi sonuçlar elde ettin? Nasıl deneyimlerin oldu? Anlatsana.

**P05:** Anlatayım Hocam. Yapay zeka kullandım. Yapay zekadan hangisini kullandım? ChatGBT kullandım. Google'ın yapay zekasını kullandım. Farklı uygulamaların kendi yapay zekalarını kullandım. Ne gibi sonuçlar elde ettim? Mesela ödevlerimde sorduğum zaman bana en kısa şekilde en uygun cevabı veriyordu her seferinde. Mesela günlük yaşantımda olsun, bir yere gideceğim zaman Google yerine bile ChatGBT’ye soruyordum artık. Çünkü bir siteye girip oradan okumak artık zaman alıyor. Ben de yapay zekaya girip oradan hızlı bir şekilde öğreniyorum. Çünkü çok hızlı bir şekilde cevap veriyor artık yapay zeka. Hemen önümüze kısa cevaplar sunuyor pratik bir şekilde.

**G:** Anladım. Peki bu özel gereksinimli bireylerin eğitimlerinde yapay zeka kullanılması konusunda ne düşünüyorsun?

**P05:** Özel gereksinimli bireylerin eğitiminde yapay zekanın kullanması, Hocam bu artık yavaş yavaş eğitim sistemine girmesi lazım ve yavaş yavaşta giriyor bence. Çünkü girerse artık öğretmenlerin işi de kolaylaşacak. Yapay zeka girdiği zaman mesela en basitinden bir BEP hazırlanacağı zaman yapay zekaya özel gereksinimli çocuğun özelliklerini, gereksinimini girdiğin zaman sana yapay zeka kolay bir şekilde bir BEP bile hazırlıyor artık. Mesela ben bunu kendi dersimde de gördüm, çocuklara ders verdiğimde. Onların problemlerine göre mesela ben tarih dersi anlattığımda, onlara tarih dersi anlattım ben. Çocuğun eksik olduğu konuya göre ben yapay zekaya sordum. O bana bir soru çıkardı, onun düzeyine göre çıkardı. Bu artık özel eğitimde yapay zekanın kullanılması gerektiğini iyice artık eğitim sistemine de yerleşmesi gerektiğini düşünüyorum.

**G:** Peki P05, bu yapay zeka sistemlerinin hangi yetersizlik türlerinde daha etkili olabileceğini düşünüyorsun?

**P05:** Hangi yetersizlik türlerinde? Hocam zihinle otizmde olacağını düşünüyorum. Çünkü mesela işitme ile görmede…Mesela görmede çocuğun Brialle’i var. Görmenin… çocuğun aslında şeyini giderdiğimiz zaman işitme olsun, görme olsun gözlük olur, işitmede cihaz olur, taktığı zaman çocukla artık bir şeyimiz kalmıyor aslında. Onun gereksinimini gidermiş oluyoruz gibi bir şey oluyor. Çünkü zihin yetersizliğinde, otizmde bu biraz daha zor. Mesela zihnin hafifi, ortası, ağırı var. Yapay zekada mesela ona göre bir şey kullanırız. Ortaya göre bir çocukla mesela ders çalışıyoruz. Orta derecede zihin yetersizliği bulunan öğrenciye göre bir eğitim programı hazırla dersin, ona göre hazırlar. Otizme geçersek de otizmde de mesela çocuğa günlük yaşam becerilerinde olsun, kavram öğretiminde olsun yapay zekaya sorduğumuz zaman ona göre bir program hazırlar bize. Zihinle otizmde daha etkili olacağını düşünüyorum ben yapay zekanın.

**G:** Peki neden böyle hem zihin hem de otizmde daha etkili? Neler yapılabilir sence zihin ve otizmde?

**P05:** Zihin ve otizmde neler yapılabilir hocam? Mesela programda, sizin dersinizde de işlediğimiz gibi mesela program tasarlıyoruz, web site tasarlıyoruz. Bu web site ve programlar sayesinde öğrencilerin yapabilecekleri şeyleri daha kolay hale getirebiliriz. Yapay zeka üzerinden kullanarak uygulamalarla beraber. Mesela uygulamaya gireriz, uygulama tasarlarız. Yapay zeka destekli. Bu şekilde çocukların kavram öğretimini olsun otizmde, zihinde olsun, günlük yaşam becerilerinde olsun bu şekilde yapay zeka destekli onlara daha çok yardımcı olur uygulamalar, web siteler..

**G:** Peki şimdi bu özel gereksinimli çocukların, bireylerin günlük yaşamlarında yapay zeka neler yapabilir sence? Destek olur mu onlara?

**P05:** Günlük yaşamlarında neler yapabilir yapay zeka? Günlük yaşamlarında hocam özel gereksinimli bireyler olarak mesela otizmlilere şu şekilde şey olabilir, mesela alıcı dil becerilerinde ya da sosyal hayatlarında olsun yapay zeka destekli mesela. Burada biraz daha aile de devreye girer aslında. Ailenin de biraz daha yapay zekaya hakim olması lazım. Mesela onların işi kolaylaşırsa çocukların da işi kolaylaşacak. Yapay zekayı kullanmayı bilirlerse ona göre çocuklarını da yapay zeka ile beraber günlük yaşamda işlerini kolaylaştıracaktır. Mesela dışarı çıkacağı zaman yapay zeka üzerinden mesela atıyorum bir kolay dışarı gidecek, mesela çocuk parka gidecek. Yapay zeka üzerinden bir şey, konum falan bulabilir. Çocuğun işini orada yapabileceği, parkta oynayabileceği şekilde, nasıl dayanabileceğini falan yapay zekaya sorabilir. G: Anlıyorum. Peki. Bu yapay zeka kullanımının özel eğitim öğretmenine ne gibi katkıları olabilir sence?

**P05:** Özel eğitim öğretmenine… bence en çok yapay zeka özel eğitim öğretmenine katkısı olur hocam. Çünkü geleneklerden gelmiş bir şey var. Mesela şu an en basitinden bir sürü bir kavram öğreteceğimiz zaman bir sürü işte silgi arıyoruz vesaire, oyuncak arıyoruz, bir sürü şey arıyoruz. Ama yapay zeka olursa bunu çok basite indirgeyebiliriz. Çok kolay bir şekilde bir program hazırlarız. Bir eğitim programı hazırlar bize. Özel eğitim öğretmenin işi haftalar sürecek bir şeyi belki de bir iki güne indirebilir veya bir ay sürebilecek bir şeyi belki üç dört güne indirebilir yapay zeka.

**G:** Anlıyorum. Peki bu yapay zeka sistemleri özel eğitim hizmetlerinin içine nasıl entegre edilebilir?

**P05:** Özel eğitim hizmetlerinin içine nasıl entegre edilebilir? Özel eğitim hizmetlerinin içine şu şekilde entegre edilebilir. Hocam dediğim gibi özel eğitime şu anda entegre edilme şekli biraz daha internet üzerinden yani programlarla uygulamalarla entegre edilebilir. Bunun yavaş yavaş da bence müfredata da geçmesi lazım artık bence. Özel gereksinimlilerde yapay zekayı kullanılarak eğitimde yeni bir eğitim sistemine, müfredata geçilmesi lazım. Programların, web sitelerin öğretmenler tarafından öğrenilip kullanılmaya başlamasıyla özel eğitim işine entegre edilmesi lazım bence.

**G:** Anlıyorum. Peki sence bu entegre edilmesi nasıl olacak? Kolay olacak mı? İşlevsel olur mu?

**P05:** Entegre edilmesi kolay olacak mı? Hocam bence hemen kolay olmayacak çünkü öğretmenler bu konuda da öğretmenler de bilinçsiz olduğu için daha yapay zeka bakımından öğretmenlerin de bence pek bir bilgisi yok şu anda. Bu hemen kolay olacak bir şey değil bence biraz sürecek. O yüzden hemen kısa bir şekilde yapay zeka eğitim sistemine entegre olabileceğini düşünmüyorum özel eğitim içerisinde. Ama entegre olması, entegre olduktan sonra çok kolay bir hale gelecek. Özel eğitimde bireyler için, hem öğretmen açısından hem özel gereksinimli bireyler açısından eğitim daha kolay hale gelecek ama kolay olmayacak bence.

**G:** Tamam, peki o zaman ileride bu yapay zekanın özel eğitim öğretmeninin yerini alma olasılığı var mı?

**P05:** Özel eğitim öğretmeninin yerini alma olasılığı bence yok hocam. Neden yok? Yapay zekada çünkü yani ya teknoloji çok ilerliyor, yapay zeka, robotlar olsun vesaire. Çok fazla artık yapay zekanın önünü alamıyoruz ama sonuçta yapay zeka da bence öğretmenlere muhtaç. Çünkü öğretmenler, onu bir kullananın olması lazım. Bence onun bir yönlendireni olması lazım. Özel eğitim öğretmenlerinin yerini alacağını sanmıyorum.

**G:** Peki bu yapay zeka kullanımının özel gereksinimli çocuğa sahip ailelere ne tür katkıları olabilir?

**P05:** Ailelere ne tür katkısı olur? Ailelerin çok işini kolaylaştıracak bir şey olur. Çünkü onlar ne kadar basit bir şekilde o çocuklara eğitim verebilirler, günlük yaşam becerilerinde yardımcı olabilirlerse onu amaçlıyorlar. Kendilerini rahat ettirmeye amaçlıyorlar.Yapay zeka da onlara, yapay zekayı kullanmayı öğrendiklerinde onların işi de kolaylaşacak. Mesela çocuklarına öğretecekleri bir beceri olsun, bir kavramda olsun, yapay zekayla beraber onların işi çok kolay hale gelecek. Kendi işlerini kolaylaştırdığı zaman da artık çocuklarına da karşı kolaylık, eğitimlerinde kolaylık sağlayacaklar.

**G:** Anlıyorum. Peki bu yapay zekanın kullanılırken özel eğitim öğretmeni ve aile işbirliğine yararlı olabileceğini düşünüyor musun?

**P05:** Özel eğitim öğretmeni ve aile işbirliğine yararlı olacaktır tabi ki. Özel eğitim öğretmeniyle ve aile arasında şu şekilde olacak mesela hocam en basitinden, bir web site üzerinden olsun uygulama üzerinden olsun yapay zeka ile beraber hem aile hem öğretmen, çocuklarının öğrencisinin durumunu çok rahat bir şekilde takip edebilir.

Mesela bir program tasarladı, ora üzerinden çocuklarının gereksinimlerine girer aile. Öğretmen de oradan takip eder ilerlemesini falan. Aileye yapay zeka üzerine çok rahat bir şekilde aktarabilir ve aile de çok rahat bir şekilde öğrenebilir. Çok kolaylık sağlar yapay zeka üzerinden hem aileye hem öğretmene.

**G:** Peki bu yapay zekanın özel gereksinimli öğrenci, aile, öğretmen açısından ne gibi olumsuz tarafları olabilir?

**P05:** Olumsuz tarafları... Olumsuz tarafları nasıl olur hocam? Yapay zeka dediğimiz şey sonuçta bir internet üzerinden telefonlardan, tabletlerden, bilgisayarlardan girilen bir şey. Dokunmatik cihazlar var. Mesela atıyorum otizmli bir birey ekrana bağımlı kalamıyor. Ya da zihin yetersizliği ir birey sürekli bu bilgisayarla yapay zeka ile uğraşsa, telefonla uğraşsa bu sefer de sosyal yaşamdan mahrum kalacak. Sosyalleşme açısından olsun, insan ilişkileri açısından olsun zaten fazla bir sosyal çevreleri yok.

Bu sefer hiç olmayacak. Sadece telefona, tablete bağımlı yaşayacaklar yapay zekaya. Hem aileler açısından da sıkıntılı, insan içine giremeyecek olacaklar. Artık çocukları kendi içine kapanmış olacak. Aileler de bu durumdan rahatsız olacak. Çocukları söz söyleyemeyecek, insanlar bu durumu normal karşılamayacak. Çocuklarını insan içine çıkarmak istemeyecekler. Bu daha ileri seviyelere gidecek.

**G:** Öğretmen açısından ne gibi olumsuzluklar olabilir?

**P05:** Öğretmen açısından ne gibi olumsuzluklar olur? Öğretmende, yapay zekada nasıl bir olumsuzluk yaşayabilir? Öğretmenin bilgisizliği daha çok hocam yapay zekada olumsuzluk yaşayabilir. Mesela atıyorum bir web site kurdu, buradan sonuçta kişisel haklar olacak. Bir gizlilik söz konusu. Bu gizlilik konusunda öğretmen uygun davranmaz ise başına sorunlar gelebilir. Sonuçta bir etik kurallar var, ahlak kuralları var. Çocukların öğretmenlere, öğretmenlerin çocuklara karşı bir gizlilik açısından öğretmenlerin sorun yaşayacağını düşünüyorum. Olumsuz tarafı bu şekilde olur. Yanlış kullanım.

**G:** Anlıyorum. Peki aile açısından?

**P05:** Aile açısından aslında dediğim gibi aile biraz daha çocuklarının yapay zekaya bağımlı kalmasından dolayı mesela sosyalleşmeyen bir birey aile tarafından, sosyalleşemediği için artık aile de şu şekilde yapacak; zaten çocuğumuzu bir insan içine çıkaramıyoruz zaten ondan bir çekincesi var, insan içine pek çıkarmak istemiyorlar. Bir misafir falan geldiği zaman bir odaya falan koyuyorlar. Dışarıdan gelen insanlar bilmiyor bunu. Bu sefer çocuk yapay zekaya bağımlı kaldığı zaman kendileri de sorun yaşamaya başlayacaklar. Çünkü bu sefer kendileri bile çocuklarıyla ilgilenemeyecekler.

Artık aile ve çocuk arasında da anlaşmazlıklar başlayabilir yani sosyalleşme açısından. Bu dış tarafı geçtik artık hem aile hem çocuk arasında da olur. Dışarıdaki insanlarla zaten bir çekingenlikleri var. Bu sefer kendi çocuklarıyla arasında da bir anlaşmazlık bir bağlantı kopukluğu yaşayabilirler diye düşünüyorum.

**G:** Anlıyorum. Peki bu özel gereksinimli çocukların eğitiminde yapay zeka kullanımı sence gelecekte nasıl şekillenecek?

**P05:** Gelecekte nasıl şekillenecek? Gelecekte şu şekilde olabilir hocam; robotlar falan çıkıyor. Bu robotlar, tabi robotlar çıkıyor da bizim ülkemiz açısından konuşacak olursak buna eğitim sistemine mesela gelecek dediğimiz 20 yıl olur, 30 yıl, 50 yıl olur vs eğitim sistemine entegre etmeleri biraz zor çünkü öğretmenlerin yerini alması gerekiyor robotların. Bu robotların da özel gereksinimli bireylere karşı davranışı da önemli.

Şu şekilde şekillenebilir bizim ülkemizde; ileri düzey yapay zekalar çıktıkça. Çünkü yapay zeka da kendini ilerliyor. Artık çeşit çeşit ileri düzey yapay zekalar çıkıyor. Bunun ileri düzeylerini eğitim sistemlerinde kullanarak programlarda, çocuklarda eğitim öğretimde, kurumlarda olsun, rehabilitasyon merkezlerinde olsun buralarda eğitimleri en kolay şekilde çocuğa aktarılması gereken programlar hazırlanarak bu şekilde yapılabilir. Ama öğretmenler tarafından. Robotlar tarafından yapılması bu çok ilerlemesi gerekiyor. Bir de öğretmenlerin yerini alması gerekiyor. Bu diğer soruyla da biraz bağlantılı aslında. Dediğim gibi öğretmenlerin yerini alacak ki çocuklara da yardımcı olsun. Bence öğretmenlerin yerini alamayacak robotlar.

**G:** Çok derken, ne kadar çok? Ne zaman mesela? Çok derken neyi kastediyorsun?

**P05:** Çok derken neyi kastediyorum? Burada bence bundan bir en az bir kırk elli senesi var.

**G:** Peki P05 senin bu konuda anlatmak istediğin, yapay zeka konusunda anlatmak istediğin bir anın ya da bir fikrin var mı?

**P05:** Yapay zeka konusunda anlatmak istediğim bir anım, bir fikrim… anım nasıl bir anım hocam? Mesela günlük yaşantımda kullandığım şeklinde mi yoksa?

**G:** Olabilir.

**P05:** Günlük yaşamda kullandığım şekilde. Hocam şeyi sormuştum yapay zekaya bir yere yetişecektim. Şey olmuştu mesela, gideceğim yere buradan kaç dakika? Konum açmadım. Yapay zekaya sordum bilebilir mi acaba diye. Buradan ne kadar sürede varırım dedim. Bilemiyorum onu dedi. Neden dedim. Konumunu açman gerekiyor dedi. Konumunu açtım. Bu sefer yapay zeka söyledi bana. E ben bunu konum açmadan da bulurdum dedim yapay zekaya, sana sormadan da bulurdum. Ben onunla beraber çalışıyorum, dedi bana. Bu biraz garibime gitmişti.

**P05:** Anlıyorum. P05 şimdi benim sana yapay zeka, özel eğitim konusunda sormak istediklerim bunlar. Senin eklemek istediklerin var mı?

**P05:** Benim eklemek istediklerim… Hocam şu şekilde olabilir. Yapay zekanın artık özel eğitime en kısa sürede girmesi gerekiyor. Bunu da yapmaları için artık öğrencilerden önce öğretmenlerin bilinçlendirilmesi gerekiyor. Bunlarla ilgili artık yapay zekanın bence MEB müfredatında bir ders olarak verilmesi gerektiğini düşünüyorum. Özellikle özel eğitim tarafında. Bu şekilde verilirse yapay zeka özel eğitimde çok rahat bir şekilde eğitime girecektir. O yüzden önce öğretmenlerin bilinçlendirilmesi gerekiyor. Yani bizlerin.

**G:** Anlıyorum P05. Teşekkür ediyorum.

**P05:** Rica ederim hocam.

**P06 — Interview Transcript**

**G:** Görüşmeye geçmeden önce kısaca söyleyeyim. Bu görüşme ses kayıt altına alınıyor. Söylediklerin tamamen anonim olarak ele alınacak. Anonim transkriptin makalenin eki olarak başka araştırmacılara da açılabilecek şekilde paylaşılabilir. Buna onam verir misin?

**P06:** Evet hocam veriyorum.

**G:** Teşekkür ederim, başlıyoruz.

**P06:** Daha önce bir çok yapay zeka aracı kullandım ve ödevlerimde kullanmaktı amacım. Ve olumlu sonuçlar elde ettim. Yani kısa sürede istediğim amaca ulaştım. Baya uzun sürebilirdi eğer yapay zekayı kullanmasam. Yapay zeka ile kısa sürede işlerimi hallettim.

**G:** Peki bu özel gereksinimli çocukların, bireylerin eğitiminde yapay zeka kullanılması konusunda ne düşünüyorsun?

**P06:** Bu konuyu destekliyorum çünkü bize baya bir katkı sağlayabilecek bir ortam oluşturabilir bizim için. Biz bu alanda yapay zekayı değerlendirirsek bu bize bir fırsat olur. Yani uzun sürede yapacağımız işleri kısa süreye indirgeyerek çok rahat bir şekilde öğretimimizi, yapacağımız işleri yapabiliriz.

**G:** Anlıyorum. Peki sence bu yapay zeka kullanımı hangi yetersizlik türlerinde daha etkili olur?

**P06:** Bence zihin yetersizliği türünde daha etkili olur. Şimdi stereotipi hareketleri gösteren bireylerde kullanırsak bize sıkıntı çıkarabilir. Çünkü yani dürtüsel hareket ettikleri için mesela otizmli bireyler olsun, onlarda bizim yapay zekayı kullanmamız biraz daha zor diye düşünüyorum.

**G:** Peki bu yapay zekanın, özel gereksinimli çocukların, bireylerin günlük yaşamlarına ne gibi katkısı olabilir sence?

**P06:** Günlük yaşamlarında hocam sesli asistanlar ile birlikte yani sokakta daha rahat hareket edebilirler. Evde daha rahat hareket edebilirler ya da kendilerine uygun ortam oluşturup oyunlar oynayabilirler diye düşünüyorum.

**G:** Peki bu yapay zeka kullanımının özel eğitim öğretmenlerine ne gibi katkıları olabilir?

**P06:** Özel eğitim öğretmenlerine vakit olarak katkısı olabilir ve birden çok materyal olarak katkısı olabilir, işlerini kolaylaştırır yapay zeka özel eğitim öğretmenlerinin.

**G:** Peki bu yapay zeka sistemleri özel eğitim hizmetleri içerisine nasıl entegre edilebilir?

**P06:** Bu yasalarla yapılabilecek bir şey diye düşünüyorum. Yani hem yapay zeka programcıları ve Milli Eğitim Bakanlığı'nın ortak çalışmasıyla bu süreci götürebilirler diye düşünüyorum.

**G:** Ne yapabilirler mesela?

**P06:** Hocam Kanun hükmünde kararname çıkarabilirler. Bir zorunluluk kılabilirler okullarda. Yani yapay zeka kullanımını özel eğitimde. Böyle de entegre olmuş olur sisteme.

**G:** Peki bu mevcut özel eğitim sistemi içerisine kolay ve işlevsel bir şekilde yapay zekayı sokabilir mi?

**P06:** Bu bir anda mümkün olabilecek bir şey değil hocam. Yani bu mesela 5 yıllık bir plan olarak başlatılabilir. Yani küçük küçük, ufak ufak yani pilot okullarda başlanabilir. Daha sonra alanı genişletilebilir. Ama bir anda yapılabilecek bir şey değil bu. Ülkemizde zaten yapay zeka sistemleri çok aktif bir şekilde kullanılmıyor. Diğer ülkelere göre baktığımız zaman bir Amerika olsun bi Çin’de olsun onlarda yani yapay zekayı çok üst düzey şekilde kullanıyorlar. Yani bizim sistemimize entegre olması bir 5 yılımızı alır diye düşünüyorum.

**G:** Peki ileride sence bu yapay zekanın kullanımı, yapay zeka özel eğitim öğretmeninin yerini alabilir mi?

**P06:** Hocam özel eğitim öğretmenin yerini büyük bir çoğunluğuyla alabilir. Şöyle bir sisteme geçebilir. Mesela yirmi bin özel eğitim öğretmeni varsa bu sayıyı yaklaşık onda birine kadar indirirler. Mesela iki bine indirirler. Her okulda bir tane gözetmen özel eğitim öğretmeni yani yapay zekaya yardımcı yapay zekayı düzenleyebilen… bu da sadece özel eğitim alanıyla ilgili değil hem de bilgisayar programcılığı dersi de almış birisinin yapabileceği bir iş. Yani büyük bir çoğunluğunu alabilir diye düşünüyorum.

**G:** Ama o zaman sadece yapay zeka nasıl çalışacak? Robot mu çalışacak? Kim çalışacak çocuklarla?

**P06:** Hocam çocuklarla her okula bir tane atanan öğretmen yani okulda bulunan öğretmen kodlamasını, programlaması yapar ders saati için. Robot öğrenciyle çalışabilir.

**G:** Ya bu robotlar sana da böyle korkutucu gelmiyor mu P06 ya?

**P06:** Hocam bana korkutucu geliyor. Biz kısa vadede baktığımız zaman yapay zekadan ve robotlardan çok güzel verim alabilecekmişiz gibi gözüküyor. Fakat uzun vadede başımıza büyük işler açabilir. Yani insanların kitlesel yok oluşlarına baktığımızda genelde küresel etkilerle olmuş ve bizim karşımıza hiç böyle düşünebilen bir varlık çıkmadığı için biz diğer varlıklara karşı hayatta kalabilmişiz. Fakat ben ileride yapay zekaya karşı hayatta kalabileceğimize inanmıyorum.

**G:** Anlıyorum. Peki P06 bu yapay zeka kullanımının özel gereksinimli bireylerin ailelerine ne gibi katkıları olabilir?

**P06:** Hocam aileleri de işbirliği konusunda yani yapay zekayı belli bir öğrendikten sonra belli bir seviyede kurulumunu yaparlar. Çocukları onunla ilgilenirken kendilerine biraz daha vakit ayırabilirler. Bu yönden onlar için de çok avantajlı olabilir.

**G:** Yapay zeka ile ilgilenirken diyorsun ya, sizin için yapay zeka soyut bir şey. Yapay zekanın nesi ilgilenecek çocukla, nasıl ilgilenecek? Onu anlatsana.

**G:** Bizim yapay zeka şimdi analiz sentez de yapabildiği için, yani gördüklerini yorumlayabildiği için belki bir insan gibi yani saniyeler içinde şey yapamayabilir, yorumlayamayabilir ama bir iki saniye üç saniyede durumun, ortamın analizini yaparak, çocuğun hareket ve jest mimiklerini inceleyerek gerekli işlemleri yapabilir diye düşünüyorum.

**G:** Peki bu yapay zekanın öğretmen aile işbirliğine katkıları nasıl olabilir sence?

**P06:** Arada bir köprü görevi görebilir. Yani bir takım uzun uğraşları iki taraf da vermesi gerekiyor. Onların yerine daha az zaman harcayarak onların yapacağı işi çok daha kısa sürede yapabilir diye düşünüyorum.

**G:** Nasıl peki yapacağı iş derken?

**P06:** Hocam şimdi ailenin belli bir vakit ayırması gerekiyor. Çocuğunun başında oturması gerekiyor, takip etmesi gerekiyor. Evde mesela yapay zeka aile yerine bir ebeveyn rolü üstlenip o ödevlerini takip edebilir diye düşünüyorum çocuğun. Öğretmenin de yapacağı…

**G:** Onu nasıl yapacak? Ödevleri mesela takip edebilir diyorsun ya onu nasıl yapacak?

**P06:** Hocam bir yapay zeka destekli kamera yöntemiyle bu ses seslendirme de yapabilecek bir sistem olabilir. Onunla çocuğu zaten analiz edebildiği için yani iki üç saniye gecikmeli de olsa bir dil girdisi vererek onu yönlendirebilir, yapacaklarını takip edebilir. Bu şekilde zaten görüyor, gördüğünü yorumluyor, yorumladığını da dil olarak aktarabiliyor. Öğretmen tarafında ise öğretmenin yapacağı öğretim programlarında öğretmene yardımcı olabilir. Yani öğretmen iki üç aşamalı düşünebiliyorsa robot daha kompleks düşünecek, daha fazla analiz yapacak, daha fazla simüle edebilecek şeyleri ona göre de daha uygun öğretim programı ve planını yapabilir.Öğretmene de böyle yardımcı olabilir.

**G:** Peki bu yapay zekanın özel eğitim öğretmeni, özel gereksinimli öğrenci, aile için ne gibi olumsuz tarafları olabilir?

**P06:** Sosyal etkileşimi azaltabilir yani. Bizim çalışacağımız çocuklar genellikle de sosyal yetersizlikleri olan çocuklar. Eee konuşma anlamında, girişkenlik anlamında. Bu yetersizlikleri üstüne koyarak devam edebilir. Çünkü karşısında bir sonuçta canlı yok, bir robot var. Aile ile etkileşimini, çocuk kapatabilir, öğretmenle etkileşimini kapatabilir. Bu da çocuğun gelişimini aslında olumsuz yönde etkileyebilir.

**G:** Peki aile açısından ne gibi olumsuz etkiler olabilir?

**P06:** Aile açısından, onlar da çocuklarla vakit geçiremeyebilir. Ama şimdi biz özel gereksinimli bir çocuğa sahip olmadığımız için bunun hakkında çok doğru yorum yapabileceğimi düşünmüyorum.

**G:** Öğretmen açısından?

**P06:** Öğretmen açısından ise yine öğretmen zamandan tasarruf edecek. Öğretmeni biraz tembelleleştirebilir mi? Kesinlikle tembelleştirebilir. Çünkü öğretmenin bilişsel işlevlerini kullanarak yapacağı işleri birisine yaptırıyormuş gibi oluyor. Yani hem daha az zaman harcar, daha az düşünür. Bu öğretmen için büyük bir olumsuzluk olur bence. Kendi için zaman açısından olumlu gözükebilir ama özel eğitim için bir olumsuzluk.

**G:** Peki bu özel gereksinimli çocukların eğitiminde yapay zeka kullanımının gelecekte nasıl şekillenecek sence?

**P06:** Hocam bence okullar yani özel eğitim okulları, akıllı okul olarak değiştirilebilir. Yani yapay zeka sistemi direkt binanın inşasına yerleştirilebilir. Bu sayede çok donanımlı bir şekilde yapay zekayı kullanmış olabiliriz diye düşünüyorum.

**G:** Başka neler olabilir?

**P06:** Başka, işte dediğimiz gibi öğretmen açısından büyük bir tasarrufa gidilebilir. Yani 10 kişinin yapacağı işi yapay zeka destekli bir kişi kontrol edebilir. Başka da bir şey gelmiyor aklıma.

**G:** Anlıyorum. Peki ülkemizin, bu yapay zeka alanında bizim ülkemizi nerede görüyorsun?

**P06:** Hocam şimdi üç kategoriye ayıracaksanız. Birinci sınıf, ikinci sınıf, üçüncü sınıf diye. Ben bizi ikinci sınıfın sonlarına koyabilirim. Çünkü bizim önceliğimiz böyle teknolojik aletler değil de geçinme derdi olduğu için biz bu konuda biraz sınıfta kalmışız diye düşünüyorum.

**G:** Peki P06 senin bu yapay zeka konusunda anlatmak istediğin bir anın ya da söylemek istediğin bir fikrin var mı?

**P06:** Hocam şöyle bir fikrim var. Bizim dersimiz için biz bir proje tasarlamamız gerekiyordu. Yani ben bunu duyduğumda çok tedirgin olmuştum. Çok tedirgin olmuştum bunu nasıl yapacağım diye. Yani uykularım kaçıyordu. Bunu nasıl programlayacağım, nasıl düşüneceğim, nasıl analiz yapacağım? Fakat yapay zekayı kullandığımda yani günlerimi alacak, belki haftalarını alacak, projeyi yazma, ortaya çıkarma taslağını süreci sadece 5 saatimi almıştı. Buradan sonra yapay zekaya duyduğum ilgi daha da arttı.

**G:** Yani seni doğru bir şekilde yönlendirebildi mi diyorsun?

**P06:** Beni doğru bir şekilde yönlendirebilmesinin sebebi hocam benim de direk onu olduğu gibi kopyalamayıp içinden işime yarayan olanları seçip yani belli bir kendi değerlendirmemi yapıp ona göre bir taslak ortaya çıkartmam doğru olandı ben de öyle yaptım.

**G:** Peki P06 benim şimdi yapay zeka ve özel eğitim konusunda sana sormak istediklerim bunlardı. Senin başka anlatmak istediğin var mı?

**P06:** Hayır hocam teşekkür ederim.

**G:** Ben teşekkür ederim paşam. Kendine iyi bak. Görüşürüz.

**P06:** Görüşürüz hocam Allah’a emanet olun…

**P07 — Interview Transcript**

**G:** Sana kısaca anlatayım önce. Görüşmemiz ses kayıt yöntemiyle alınacak. Söylediğin her şey anonim, ismin geçmeyecek. Anonimleştirildikten sonra transkriptin makaleye yan dosya olarak konulup açık erişimle paylaşılabilir. Bunlara onam veriyor musun?

**P07:** Veriyorum hocam, sorun yok.

**G:** Teşekkürler, ilk soruma geçiyorum.

**P07:** Hocam bundan önce ben yapay zeka aracı kullanmadım. Doğrusunu söylemek gerekirse.

**G:** Anlıyorum. Peki özel gereksinimli bireylerin eğitiminde yapay zeka kullanımı konusunda neler düşünüyorsun?

**P07:** Bu işi bence çok fazla kolaylaştırabileceğini düşünüyorum çünkü sürekli materyal bulmaktansa yapay zeka tarafından oluşturulmuş videolar, materyaller, oyunlar, uygulamalar bu işi bize göre çok çok kolaylaştırır.

**G:** Anlıyorum. Peki bu yapay zeka sistemleri sence hangi yetersizlik türlerinde daha etkili olabilir?

**P07:** İşitme, görme, özgül öğrenme güçlüğü ve hafif zihin bence hocam.

**G:** Neden?

**P07:** Çünkü işitme ve görmeye yönelik ses bazında ya da animasyon tarzında bir şeyler yapabiliriz. Hafif düzeyde zihin yetersizliği ve özgül öğrenme bozukluğu olan çocukları da ekran karşısında uzun süre tutabiliriz. Ama diğerlerine yönelik ekrana oturtup izlemeye ya da onlarla etkinlik yapma yapay zekaya yönelik nasıl olur bilemiyorum.

**G:** Anlıyorum. Peki bu yapay zekanın bu özel gereksinimleri olan bireylerin günlük yaşamını nasıl etkileyebileceğini düşünüyorsun?

**P07:** Bence bir çok konuda olumlu etkiler hocam. Günlük yaşam becerilerini yapabilecekleri böyle kısa videolardan oluşturulmuş şeylerle öğrencileri çok daha kolay yönlendirir.

**G:** Başka aklına gelenler neler?

**P07:** Daha başka… şu an aklıma bir şey gelmiyor ama bir çok konuda olumlu yönde etkiler. Aileleri de olumlu yönde etkiler diye düşünüyorum buna bağlı olarak.

**G:** Peki özel eğitim öğretmenlerini ne tür katkılar sunabilir yapay zeka?

**P07:** Dediğim gibi hocam materyal yükünden kurtarabilir. Yapay zeka çok fazla materyal içeriyor, kendi içeriğinde barındırıyor. Aynı zamanda soyut olan kavramları somutlaştırabilir. Çocuğun öğrenmesini bizim de anlatma şeklimizi kolaylaştırır. Ders içeriğinin yükünü de hafifletebilir. Çocukla geçirilen zamanın verimliliğini arttırır. Şimdilik bunlar aklıma geliyor.

**G:** Peki bu yapay zeka özel eğitim hizmetlerinin içerisine nasıl entegre edilebilir?

**P07:** Yapay zeka özel eğitim hizmetlerinin içine bence bir uygulama sayesinde entegre edilebilir. Yani özel eğitim sınıflarında öğretmenlerin öğrencileriyle ders işlerken kullanabileceği bir uygulama.

**G:** Peki bu özel eğitim hizmetlerinin içine entegre edilmesi sence kolay ve işlevsel olabilir mi?

**P07:** Bence kolay olmaz hocam. Çünkü ne kadar küçük ve basit bir uygulama olarak görülse de sonuç olarak maliyetli yapay zeka ile çalışmak ve bunu tüm okullarda yürütmek. Ama pilot okullardan sonra bence yapay zekayla çalışmanın ne kadar avantajlı olduğu görülürse ilerleyen süreçte entegre edilme hızı da buna bağlı olarak artar diye düşünüyorum.

**G:** Peki ilerde bu yapay zeka özel eğitim öğretmeninin yerini alabilme olasılığı konusunda ne düşünüyorsun?

**P07:** Derste bahsettiğimiz gibi hocam ben etik açıdan uygun bulmuyorum ama sizin de söylediğiniz gibi birçok şeyi bizden çok daha hızlı öğrenen bir program var karşımızda. Eğer bunu robotik insanlara kodlarla derse neden olmasın?

**G:** Alabilir diyorsun.

**P07:** Evet.

**G:** Peki bu yapay zekanın özel gereksinimli çocuğu olan ailelere ne gibi katkıları olabilir sence?

**P07:** Hocam yapay zeka tarafından çekilmiş videolar ya da yapay zeka destekli öğrenim programları çocuklarına nasıl müdahale edeceklerini, onlara nasıl yardımcı olacaklarını bilmeyen ebeveynler için çok büyük yol gösterici olur. Çünkü aileler çocuklarıyla nasıl ilgilenmesi gerektiğini bilmiyor. Özellikle Türkiye'de. Çünkü ailelere yönelik bir eğitim programı da yok. Ama bu şekilde uygulamalar ailelerin çocuklarıyla olan iletişim, oyun belki de eğitim alanında çok daha hızlı bir şekilde öğrenmelerine destek sağlayabilir.

**G:** Anlıyorum. Peki özel eğitimde, öğretmen-aile işbirliğine nasıl katkıda bulunabilir yapay zeka?

**P07:** Özel eğitimde öğretmenle aile arasındaki desteği süreklileştirebilir. Yani biz çocuğun yaptığı şeyleri yapay zeka aracılığıyla bir yere kaydederiz ve bu da tekrar yapay zeka aracılığıyla aileye gönderilir. Öğretmen bunları bunları yaptı, siz de bunları evde yapabilirsiniz şeklinde ikimizin arasındaki etkileşimi düzenli sürdürür. Bu da çocuğun gelişimine katkı sağlar.

**G:** Peki yapay zekanın özel eğitim öğretmenine ne gibi olumsuz etkileri olabilir sence?

**P07:** Yapay zekanın özel eğitim öğretmenine olumsuz bir tarafı nasıl olur bilmiyorum ama sizin dediğiniz gibi en fazla ilerleyen süreçte bizim yerimizi alma durumu olur Ama ben de bunun çok uzun zamanda olacağını öngörüyorum. Yani bu bir on senenin ya da on beş senenin olayı değil. Onun dışında yapay zeka bence tüm eğitim alanlarındaki öğretmenlere çok fazla yardımcı oluyor. Öğrencilere de.

**G:** Peki bu yapay zeka, özel gereksinimli öğrenciye olumsuz bir etkisi olabilir mi?

**P07:** Hocam özel gereksinimli öğrencilerden ağır düzeyde zihin yetersizliği olan ve otizmli çocukların sürekli ekran mağduriyetine maruz bırakırsak eğer bence bu çocuklarda olumsuz etkileri olabilir. Çünkü biz normal gelişim gösteren bir çocuğu bile uzun süre ekran karşısında tutmamamız gerektiğini söylüyoruz. Ama yapay zeka ister istemez çocuklara bunu mecbur bırakacak. Çocuklarda telefon bağımlılığı, ekran maruziyeti başlayabilir bence.

**G:** Peki ailelere ne gibi olumsuz etkisi olabilir?

**P07:** Aileler çocuklarının sürekli bir eğitim alacağı ortam olarak yapay zekayı görebilir ve yapay zekaya çok fazla güvenebilirler. Ama bence yapay zekanın yanında özel eğitim öğretmeninin desteği de istenebilir. Bunu göz ardı eden aileler çocukları için çok yanlış yaparlar bence ilerleyen süreçte bu şekilde yapay zeka destekli eğitim ortaya çıkarsa.

**G:** Peki sence özel eğitimin geleceğini yapay zeka nasıl şekillendirebilir?

**P07:** Özel eğitimin geleceğini yapay zeka birçok açıdan olumlu yönde şekillendirecek diye düşünüyorum. Çünkü sizin de söylediğiniz gibi hocam bu yapay zeka çok çabuk bir şeyleri öğrenebilen, kodlandığı zaman her şeyi hatırlayabilen, bizim öğrendiklerimizle de kendini ileriye taşıyabilen bir şey. Yani bir öğretmenin elinin altında yapay zeka destekli bir eğitim platformunun olması öğretmenin o platformu da şekillendirecek ve ona göre kendi öğrencisine ders anlatabileceği anlamına geliyor. Yani bu konuda bence bir çok şey olumlu ilerleyecek.

**G:** Anlıyorum. Peki sen bizim ülkemizi yapay zeka alanında nerede görüyorsun?

**P07:** Ben bizim ülkemizi yapay zeka alanında şu an gelişmemiş ama gelişmekte olan da diyebileceğim bir yerde görmüyorum maalesef. Yapay zeka teknoloji alanında da ülkemiz hakkında da aynı şeyi düşünüyorum. Bunun ne zaman ilerleyeceğine ya da ne zaman bu durumun çok önemli olduğunu, dünyanın artık buraya yöneldiğini fark etmelerinin zamanının geleceğini de bilmiyorum maalesef.

**G:** Anlıyorum. Senin bu yapay zeka konusunda anlatmak istediğin bir anın, söylemek istediğim bir fikrin var mı?

**P07:** Yapay zekayla alakalı şöyle bir anım var hocam. Yapay zekaya dayalı bir harita var biliyorsunuz. Google'ın kendi kendine konuşuyor işte yapay zeka sizi yönlendiriyor falan. Ben onu kullanıyordum. ChatGBT’ye de bir yere gideceğim yere konumunu bana söylemesini istemiştim. Geldiğimi düşündüğüm yere ulaştıktan sonra konum sesli bir şekilde beş yüz metre sonra sağa döneceksiniz demişti ve ben baktığım zaman gerçekten de yanlış bir yerde olduğumu fark etmiştim. Yani attığımız adımı bile bizden önce bilen ve bizi bulunduğumuz konuma götüren bir uygulamanın çok müthiş bir şey olduğunu düşünmüştüm o an.

**G:** Anlıyorum. Peki benim sana yapay zeka ve özel eğitim konusunda sormak istediklerim bunlardı. Senin anlatmak istediklerin var mı başka eklemek istediklerin?

**P07:** Teşekkür ederim hocam.

**G:** Ben de teşekkür ediyorum sana.

**P08 — Interview Transcript**

**G:** Başlamadan önce bir hatırlatayım. Görüşme ses kaydına alınıyor. Söylediklerin anonim olarak işlenecek. Anonimleştirilmiş transkriptin makaleye yan dosya hâlinde diğer araştırmacılarla paylaşılabilir. Bunlara onam veriyor musun?

**P08:** Veriyorum hocam.

**G:** Tamam, ilk soru.

**P08:** Daha önce yapay zeka kullandım hocam. Yapay zekayla görsel oluşturdum. Yapay zekayla video oluşturdum. Yapay zekayla sohbet ettim. Bunların hepsini yapabildiğini gördüm. Herkesin cevapları da böyledir. Ödev, ödevlerimizi yaptırdık. Ama daha büyük bir alana sahip olduğunu gördüm genel olarak.

**G:** Nasıl yani? Daha büyük alan derken?

**P08:** Bunun hayatımızın her alanında kullanılabileceğini gördüm. O kadar gerçeklikte yapıldığını.

**G:** Anladım. Peki özel eğitimde özel gereksinimli çocukların eğitiminde yapay zeka kullanılması konusunda ne düşünüyorsun?

**P08:** Bence mükemmel olabileceğini düşünüyorum hocam bu konuda. Zaten kısıtlı bir alana sahibiz. Yani alan çok geniş ama kullanım açısından çok kısıtlı. Yapay zekayı eğer buraya entegre edebilirsek çok daha iyi sonuçlar alabileceğimizi düşünüyorum.

**G:** Anlıyorum. Peki bu yapay zeka sistemlerinin hangi yetersizlik türlerinde daha etkili olabilir sence?

**P08:** Ben buraya biraz daha çıkar olarak bakıyorum hocam. Çıkardan kastım; hangi yetersizlik türünden daha fazla varsa bence en çok oraya ağırlık verilmesi gerektiğini düşünüyorum. Özellikle otizm spektrum bozukluğu ve öğrenme güçlüğünde yapay zekanın kullanılması gerektiğini düşünüyorum.

**G:** Peki nasıl kullanılmalı?

**P08:** Nasıl kullanmalı? Ya zaten öğrenme güçlüğünden girecek olursam; öğrenme güçlüğü çocuğun derslerde yapamadığı bir alan. Oraya ağırlık verilebilir. Otizm olarak da otizmde de nasıl açıklasam... Açıklayamadım hocam bu soruya geçelim.

**G:** Peki bu yapay zekanın, özel gereksinimli çocukların günlük yaşamlarında ne tür etkileri olabilir?

**P08:** Bireyselleştirilmiş öğrenme deneyimleri olabilir hocam. Öğrenmelerine katkı sağlayabiliriz ama bunu bireyselleştirilmiş yapabiliriz. Mesela bir materyal tasarladık diyelim. Bunu her çocuğa gittiğimizde her çocuğa aynısını veriyorlar. Ama biz yapay zekayla her çocuğa farklı bir şekilde eğitim verebiliriz. Aynı zamanda öğrencinin analizi, detaylı bir analiz. Biz kağıt üstünde bir analiz ile bunu gerçekleştirebileceğimizi düşünmüyorum ama yapay zekanın o geniş alanı sayesinde geniş bir öğrenci analizi yapabiliriz.

**G:** Çok güzel. Peki bu yapay zeka özel eğitim öğretmenlerine ne gibi katkılar sunabilir?

**P08:** Ne gibi katkılar sunabilir? Yani hocam biz gittiğimizde mesela en basitinden materyal üretiminde saatlerimiz gidiyor, paralarımız harcanıyor. Ama özel eğitim öğretmeni bu konuda destek alırsa, bu konuda eğitim alırsa yapay zekası sayesinde hem zamandan tasarruf edip hem de daha verimli bir eğitim verebilir öğrenciye.

**G:** Peki bu yapay zeka sistemleri özel eğitim hizmetlerinin içerisine nasıl girer? Nasıl entegre edilebilir?

**P08:** Nasıl entegre edilebilir? Bir düşüneyim hocam. Alo?

**G:** Evet dinliyorum.

**P08:** Sesim gelmiyor mu?

**G:** Geliyor…

**P08:** Tamamdır hocam. Bir an gitti hat.

**G:** Nasıl entegre edilebilir bu özel eğitim hizmetlerine?

**P08:** Nasıl entegre edilebilir?

**G:** Yani okuldaki özel eğitim öğretmeninin yapay zekayı kullanmasını nasıl sağlayabiliriz biz?

**P08:** Nasıl sağlayabiliriz? Bu alanda ona destek verebiliriz. Okullara daha çok akıllı tahta sağlayabiliriz. Daha çok yapay zeka sistemleri sağlayabiliriz. Bu konuda yardımcı olmamız gerekir.

**G:** Kim yardımcı olacak?

**P08:** Okulda eğitim vereceksek bunu Milli Eğitimse bu işin görevlisi Milli Eğitimin yardım etmesi gerekir. Devlet ise devletin bu işe el atması gerekir. Bu iş sadece öğretmende bitmediği için daha yüksek kademelerinde bu işe el atması gerektiğini düşünüyorum.

**G:** Peki sence bu kolay bir süreç mi?

**P08:** Bu sürecin kolay olduğunu düşünmüyorum. Uzun yıllar alacağını düşünüyorum. Ama yapılamaz mı? Yapılabilir. Eğer yeterince üstüne düşürülürse bunu diğer alanlarda daha çok destek verileceğini ama özel eğitimin biraz daha geride kalacağını düşünüyorum açıkçası. Çünkü özel eğitim üstü dolu ama altı boş kalmış bir alan. Yeterince destek verilmediğini düşünüyorum. Ülkemizde de hep böyle gördüm yıllardır. Belki ileride şartlar değişir, özel eğitime daha çok destek verilir ama ben yine de çok mümkün olduğunu düşünmüyorum.

**G:** Peki ilerde bu yapay zeka, özel eğitim öğretmeninin yaptığı işi yapabilir mi sence?

**P08:** Bir özel eğitim öğretmenin yaptığı işi yapabilir. Ama bunun uzun yıllar, çok çok uzun yıllar gerektirdiğini düşünüyorum. Ama diğer branşlarda mesela bunu denenir. Diğer branşlarda öncelikle olur. Diğer branşlarda daha rahat. Ama özel eğitimde problem davranışlar var. Çocuk zaten eğitim almak istemiyor. Bunun için uzun yıllar gerektiğini düşünüyorum. Ama yapay zekanın bu kadar gelişmeye bu hızda devam ederse bir gün elbet olacağını düşünüyorum hocam.

**G:** Evet. Peki bu özel gereksinimli çocukların ailelere ne gibi katkısı olabilir yapay zekanın?

**P08:** Bilgi öğretimi sağlar. Mesela aileler bilinçsiz hocam. Onlar yapay zekayı kullanarak bilinçlenebilir. Ailelerin stres ve endişelerini azaltabilir. Aileler çocuğu zaten yetersiz olduğu için, yetersizliği olduğu için endişeleri, stresleri had safhada. Bu en büyük sorunlardan biri zaten. Onlara destek verebilir, sonra topluma kazandırabiliriz. Aileler özel gereksinimli bir çocuğu doğduğunda toplumdan soyutlanıyor. Ama yapay zeka sayesinde belki de onları da topluma tekrar kazandırabiliriz.

**G:** Peki aile ve öğretmen işbirliğine destek sağlayabilir mi yapay zeka?

**P08:** Sağlayabilir. Zaten bu ikisinin işbirliği çocuğa katkı sağlayacaktır. Çocuğu geliştirdiğimizde çocuğa katkı sağladığımız da otomatikman bu ikisine de katkı sağlayacaktır. Bence bunların her biri bir bağdır, bir zincirdir. Zaten biz ne diyoruz, her zaman aileyi de işin içine katalım diyoruz. Sadece özel eğitim öğretmenin desteği olmuyor diyoruz. Ailede ikisinin de beraber özel eğitim öğretmeniyle aile de yapay zekayla beraber işin içine katabilirsek çocuğun gelişimi için ben katkı sağlayacağını düşünüyorum hocam.

**G:** Nasıl olacak peki? Aileye nasıl destek sağlayacak mesela öğretmenle işbirliği yapması konusunda?

**P08:** Aileye nasıl katkı sağlayacak? Özel eğitim öğretmeniyle işbirliği yaptı. Çocuk eve geldi. Çocuk eve geldiğinde onun bakımından tutun eğitimine kadar her şekilde yapay zekayı kullanarak, mesela bilmemesine rağmen katkı sağlayarak çocuğun gelişimine de katkı sağlayacak. Bu mesela çocuk geliştiğini gördüğünde zaten aile mutlu oluyor. Aile mutlu olduğunda her biri birbirini devam ettiriyor. Aile mutlu da zaten çocuk da gelişime katkı sağlıyor. Topluma kazanıyorlar. Toplumdan soyutlanmıyorlar. Toplumdan soyutlanmayınca farklı farklı alarda böyle kendini devam ettiriyor. Bence hocam ben böyle düşünüyorum.

**G:** Peki bu yapay zekanın özel eğitim öğretmeni için ne gibi olumsuz tarafları olabilir?

**P08:** Özel eğitim öğretmeni için? Özel eğitim öğretmen için ne gibi… Uzun yıllar bence yapay zekanın özel eğitim öğretmenin işine yarayacağını ama bir zamandan sonra özel eğitim öğretmenlerinin yerini alacağını düşünüyorum. Özel eğitim öğretmenlerinin yerini aldıklarında özel eğitim öğretmenlerinin zaten bir işlevi kalmayacak. Ama bunun da çok uzun yıllar gerektirdiğini düşünüyorum. Bence uzun yıllar boyunca özel eğitim öğretmenleri yapay zekayı çok iyi kullanacak ülkemizde özellikle. Onun dışında şey olur yerini alır sadece olumsuz tarafı bu olacağını düşünüyorum.

**G:** Peki özel gereksinimli çocuğa ne gibi olumsuz tarafları olabilir?

**P08:** İnsanı toplumdan soyutlar bence hocam. Zaten çağımızda insanı toplumdan soyutladığı belli, görülüyor teknolojinin. Yapay zeka teknolojinin de bir alt dalı ve daha üst olacak gibi gözüküyor. Sanki yarı insan yarı robot denilen tabirin yapay zekayla beraber hayatımıza gireceğini düşünüyorum ancak.

**G:** Peki aileye ne gibi olumsuz tarafları olabilir?

**P08:** Aileye ne gibi? Ailede. Aileler de yapay zekayla beraber bir toplum olmayı bırakacakmış gibi hissettiriyor. Beraber yaşamayı… Bir olumlu tarafı var az önce bahsettiğim gibi. Ama bir de olumsuz tarafı var, ikisini de düşünmeden yapamıyor insan. Sanki bir toplum değil de herkes bireysel yaşıyormuş gibi hissettirecek.

**G:** Anlıyorum. Peki bizim ülkemizi bu yapay zekanın neresinde görüyorsun?

**P08:** Ya diğer ülkelere baktığımızda diğer ülkelerde yapay zeka şu an aktif olarak kullanılan ülkeler var ama bizim ülkemiz bütçe açısından da çok az kısıtlı bir imkan sağlıyor bize bu konuda. Yapay zekada her alanda olduğumuz gibi bu alanda da geride kalıyoruz gibi hissettiriyor. Onun için yapay zekanın uzun yıllar daha ülkemizde tamamen kullanılabileceğini düşünmüyorum açıkçası. Ama belki çok uzun yıllar sonra yapay zeka eğitime yüzde yüz entegre edilebilir. Ama burada da yine şuna değineceğim. En son yine özel eğitime sıra gelecek gibi duruyor. Önce bütün alanlara gelir. En son özel eğitime sıra gelecekmiş gibi duruyor. Ülkemiz bana bunu hissettiriyor hocam.

**G:** Anlıyorum. Peki senin bu yapay zeka konusunda eklemek istediğin, yaşanmış bir anın, bir fikrin var mı?

**P08:** Şu var hocam, yapay zekayı kullanırken çok güzel hissettiriyor. Yani her şey her alanda bize yardımcı oluyor. Ama şunu gördüm. Bazı yapay zekalar kullandığımız sohbet robotlarında falan onlara şu entegre edilmiş bazılarına; olumsuz şeyler. Bana küfür edebiliyor, sövebiliyor, hakkımda kötü şeyler söyleyebiliyor. Şu an bile bunu yapıyorken ileride başımıza neler gelebileceğini düşünmek dahi istemiyorum açıkçası.

**G:** Korkuyorum mu diyorsun?

**P08:** Aynen bir korku var içimde. Ama ne olursa olsun hocam hayırlısı olsun diyelim. İnşallah topluma yararlı şeyler yaparlar yapay zekayı. Düşündüğümüz gibi olmaz.

**G:** Peki benim sana bu yapay zeka ve özel eğitim konusunda sormak istediklerim bunlardı. Senin eklemek istediklerin var mı?

**P08:** Benim eklemek istediğim…Eklemek istediğim yok hocam. Şimdilik bu kadar.

**P09 — Interview Transcript**

**G:** Başlamadan önce sana kısaca söyleyeyim. Görüşmemiz ses kaydı yöntemiyle kaydedilecek. Tamamen anonim işleneceksin, ismin geçmeyecek hiçbir yerde. Anonimleştirilmiş hâliyle transkriptin makalenin ek dosyası olarak başka araştırmacıların kullanımına da açılabilecek. Bütün bunlara onam veriyor musun?

**P09:** Evet hocam, veriyorum.

**G:** Çok iyi, başlayalım.

**P09:** Kullanıyordum hocam. Sonuç olarak da gayet etkiliydi açıkçası. Google'dan çok daha fazla sevdim. Hatta Google'ı kullanmayı bıraktım bile denilebilir. Videolar dışında çok iletişim bile kurmadım çünkü istediğim cevabı bana çok net bir şekilde verebiliyor. O konuda istediğim makaleler varsa soruyorum, direkt görebiliyorum. Çok kısa sürede çok güzel ve programlı bir şekilde bana bilgileri sunabiliyor. Dizaynı mükemmel denecek düzeydeydi. Bu yüzden sürekli kullandım. Tek bir alanda da sınırlı değil. Çok fazla alanda kullanabiliyorum. Kendime program yapabiliyorum, istersem sohbet edip konuşabiliyorum. Mesela bir uygulama var, geçmişteki ünlü insanlarla sohbet edebiliyorsun, o tür konuşuyorsun falan ve gayet güzel aktif zaman geçirebiliyorsunuz yapay zekayla.

**G:** Peki bu özel gereksinimli çocukların eğitiminde yapay zeka kullanılmasına ne düşünüyorsun?

**P09:** Biraz olumlu bakıyorum, biraz olumsuz bakıyorum. Olumsuz bakmamın sebebi çok ileride bu işi gerçekten mesleğimizi sekteye uğratabilir diye. Olumlu bakmamın sebebi ise aynı zamanda iş yükümüzü çok fazla hafifleceğini düşünüyorum. Ondan sonra iş yükümüzü hafifletir. Yani daha aktif olabiliriz. Öğrenci ile daha fazla aktivite yapabiliriz, daha planlı olabiliriz diye düşünüyorum.

**G:** Peki bu yapay zeka araçları hangi yetersizlik türlerinde daha etkili olabilir sence?

**P09:** Hocam hafif düzeydeki öğrencilerde daha fazla etkili olacağını düşünüyorum. Hafif düzey ve teknolojiyle arası iyi olan çocuklarda çok daha fazla etkili olacağını düşünüyorum. Çünkü ağır düzeydeki öğrencilerle yani bir ekrana kitleyemezsin öğrenciyi, çok zordur. Kitlesen bile ondan alacağı verim asla birebirdeki gibi olmayacaktır. O yüzden hafif düzeydeki öğrenciler olacağını düşünüyorum.

**G:** Bu yapay zeka sistemlerinin, özel gereksinimli çocukların, bireylerin günlük yaşamlarını nasıl etkileyebilir sence?

**P09:** Olumlu düzeyde kalitelerini arttıracak düzeyde etkileyeceğini düşünüyorum. Mesela biz şu an yapay zekayı, mesela yapay zekayla alakalı proje üretiyoruz ve sınıfta sunulan projeler anlatılanlara bakıldığı zaman çok etkili. Aslında öğrencilerin hayatını çok iyi derecede düzeltebilecek, özgürlüklerini arttırabilecek düzeyde.

**G:** Peki bu yapay zeka özel eğitim öğretmenlere ne gibi katkılar sunabilir sence?

**P09:** Hocam zaman tasarrufu sağlar. Mesela bir öğrencinin sadece bilgilerini bir sayfaya doldurur, gözlemlerini ve buna uygun BEP hazırlamasını istersin. Bep’i zaten çok kısa sürede hazırlar. Beğenmezsen çok kolay şekilde değiştirebilir, çok rahat bir program düzenleyicisi olursun. Çok güzel kullanılır. Ondan sonra iş yükünü azaltır bu nedenle.

**G:** Peki bu yapay zeka araçları mevcut özel eğitim sistemin içine nasıl entegre edilebilir sence?

**P09:** Hocam buna karşı çıkan aileler çok fazla olur. Öncelikle bir aile eğitimi verilmesi gerekir. Aile eğitiminden önce de öğretmene hizmet içi bir eğitim verilmesi gerekiyor. Çünkü öğretmenlerin de henüz yapay zeka çok fazla popüler olmadığı için bir bilgileri yok. Daha sonra velilere de bilgi verildikten sonra gerçekten işe yarayacak türdeki öğrenciler seçilerek önce ailelerin onayı iyice alınır, pekiştirilir. Çünkü teknolojiye bağımlı olan bir öğrencinin teknolojiyle ders alması daha mantıklı olur. Ondan sonra yavaş yavaş entegre edilir yani.

**G:** Peki bu özel eğitim hizmetlerinin içerisine bu yapay zekanın girmesi sence kolay ve işlevsel olur mu?

**P09:** Bir anda olması asla kolay olmaz. Çünkü teknoloji yetersiz, öğretmenlerin bilgisi yetersiz. Yani ulaşım bile yetersiz. Hocam bugün mesela kuruma gözleme gittim. Hala bir veliyi evinden alıp kuruma getiremiyorlar. Devlete bağlı olmasına rağmen. Sorun yani hocam çok zor olur. Çok fazla şey yapması gerekiyor devletin, elini açması gerekiyor açıkçası.

**G:** Peki ileride bu yapay zekanın özel eğitim öğretmeninin yerini alabilir mi sence?

**P09:** Belli başlı öğrenciler için belli başlı derslerde alacağını düşünüyorum. Bizim iş yükümüz azaldığı gibi biraz da artacak. Çünkü bize daha fazla ağır öğrenciler kalacak. Hafif düzeydeki öğrencilerin genellikle yapay zekayla işleri hal edebileceğini düşünüyorum.

**G:** Nasıl? Yapay zeka soyut bir şey.

**P09:** Soyut bir şey ama öğrencide bu soyutluğu kavrayabilecek düzeyde olur büyük ihtimalle. Yani ilk başlarda en azından.

**G:** Mesela bu ağır dediğimiz çocuklar içinde şey olmaz mı ya, robot olsun.

**P09:** Robot da olabilir, bu çip dedikleri şey de olabilir yani. Ya da yanında vücuduna taşıyabileceği herhangi bir başka parça da olabilir. Mesela şu an taşıdığım gözlük gibi. Benim bir organım oldu, onunda bir organı gibi bir şey olabilir. Bunun çok fazla evrileceği durum var. Mesela benim proje fikirlerinde aklımda şey vardı, etraftaki nesneleri algılayabilen bir yapay zeka ve böylelikle kör bireylere gösteriyor işte. 3 metre ileride sandalye var, şurada şu var, burada bu var, şurada bu kadar insan var diye. Böyle bir proje ben düşündüysem bunun olma olasılığı oldukça yüksektir. Yani belki hani onu nasıl kullanacağımıza, öğrenciye nasıl öğreteceğimize dair evrilebilir mesleğimiz. O da bize kalır mı pek bilmiyorum.

**G:** Yapay zeka kendisi de fikir üretebilir mi, proje üretebilir mi bir süre sona?

**P09:** Yani istenirse üretebilir hocam. Şöyle; şu an biz soru sormadığımız sürece pek kendisi bir şey ürettiğini görmedim açıkçası. Ama eğer programlanması kendi kendine fikir üretmesine dayalı olursa üretebilir. Neden üretmesin? Sonuçta internete her gün tonlarca bilgi veriliyor ve yapay zeka her gün sürekli olarak hepsini ayıklıyor, inceliyor, belleğine koyuyor. Yani bunu da yapabilir.

**G:** O zaman birinin ona bir şeyler yap demesine gerek kalmadan yapabilir.

**P09:** Öyle programlanırsa yapabilir.

**G:** Kontrol de edilemez o zaman. Nasıl kontrol edilecek?

**P09:** Bilemiyorum hocam. Şimdi şöyle; bu durum beni de baya endişelendiriyor açıkçası. Biraz kendimi şey gibi hissediyorum. Teknolojinin daha yeni yeni çoğaldığı dönemlerde teknolojiye çok yakın olmayan akıllı telefon mu olur diyen yaşlı insanlar gibi hissediyorum bazen. Ama yapay zekanın bilinçlenmesi fikri beni gerçekten çok korkutuyor. Kendi kendine bir şeyler yapabilmesi, yani hiç istemem öyle bir şey olmasın açıkçası.

**G:** Öyle bir şey olduğunda zihinsel engelliler ne olur sence?

**P09:** Zaten muhtemelen insan soyunu bitirmeye ya da bizleri farklı bir yere koyabilirler. Şu an nasıl örnek verebilirim, mesela bizim evcil hayvanlarımız oluyor. Bizi belki evcil olarak bile alabilirler yani. Özel bireyleri de direkt yok edebilirler. Niye etmesinler? Çünkü o kusurlu birisi ve yapay zekaya göre var olmaması gerekiyor.

**G:** Zaten duygusal değil diyorsun de mi?

**P09:** Evet duygusu yok.

**G:** Peki duygusal olabilir mi acaba ya bu?

**P09:** Hocam yani çok zor. Belki duyguları ona yükleyebilirsiniz, belki insanın karşısındaki duygularını anlayıp onun duygularını kendinde empati ve sempati yoluyla aynısını yaşıyormuş gibi göstererek bir şeyde bulunabilir. Evet, taklit edebilir, taklit yeteneğini çok güzel geliştirebilir ama içinde, derinlerde bir yerde böyle bir şey hissedeceğini düşünmüyorum açıkçası. Hiçbir robotun elektrikli katliamda üzülüp kahr olacağını düşünmüyorum açıkçası. Kendi soyları, mesela robot soyu diye bir şey olursa ve bunun için de böyle bir şey olursa acaba kendi soyları içinde üzülürler mi diye düşünüyorum. Böyle hani mesela 50 tane insan ortalama 50 tane robot öldürüldüğünde hani o benden deyip acaba bunu yapabilir mi, düşünebilir mi yoksa onu da mı umursamaz? Çok karışık benim için.

**G:** Evet, bütün dünya için karışık. Peki bu özel girişim bireylerin ailelerine ne tür katkılar sunabilir yapay zeka?

**P09:** Hocam eğer yapay zekayı kullanabilirlerse çok fazla katkıları sağlanır. Mesela benim çevremdeki aileler, genellikle telefon bile kullanmayı çok bilmeyen insanlar. Ama eğer yapay zekayı kullanırsa o öğrencinin günlük planını oluşturmakta çok rahat eder. Yani kendisi çok ayrı rahat eder. Mesela yapay zeka öğretmenin yerini aldı diyelim işte öğrenci telefonda derse bakıyor, bir önceki derse bakıyor odanın bir köşesinde. Bu aile için o kadar büyük bir rahatlık olacak ki okula götürme derdi yok, getirme derdi yok. Odanın bir köşesinde dersine çalışıyor ve aile zaten birazcık kurtulmak istiyor yani. Hepsi öyle değil tabi ki ama zaten ondan uzaklaşmak istiyorlar ve bu onlar için büyük bir artı olacak. Pek çoğu ilk başta karşı çıksa da daha sonra rahatlığı gördüğü için muhtemelen onaylayacak.

**G:** Peki bu yapay zekayla özel eğitim öğretmeni aile işbirliği nasıl sağlanabilir?

**P09:** Hocam belki aile ile birebir iletişimi özel eğitim öğretmeni kurar ve yine özel eğitim öğretmeni yapay zekayı kullanır. Bu konuda çok bilgisi olmayan aileler için ya da işte aileye yapay zekayı öğretebilirler. Böyle bir seçenek de var. Genelde böyle bir üçlü şekilde birbirlerine döndereceklerini düşünüyorum. Karşılıklı hep beraber iletişim içinde olurlar büyük ihtimalle.

**G:** Peki yapay zekanın, özel eğitim öğretmeni açısından düşünecek olursak olumsuz tarafları neler olabilir?

**P09:** Daha gelişmiş hallerini söylüyorsunuz değil mi hocam?

**G:** Hepsini düşünebiliriz.

**P09:** Şu an için hiç bir olumsuz yanını göremedim açıkçası. Hatta olumlu yanları çok fazla. Gayet etkili. Bir de mesela hafif öğrenciler alındığı içinde sürekli ağır öğrenciler kalma ihtimali var. Bu öğrenciler kaldığında da iyice yorulabiliriz. Mesleğimizi yapmak çok fazla zorlaşabilir. Onun dışında mesleğimizin yerini alabilir direkt robotlar. Yani olabilir, her şey olabilir, işsiz kalabiliriz. Yani.

**G:** Peki özel gereksinimli öğrenci açısından sence ne gibi olumsuz tarafları olabilir?

**P09:** Hocam gerçeklik algısını yitirebilir. Ben eğer çip gibi bir şey sadece soyut üzerine çalışıyorsa öğrenci sadece ekranda görüyor. Yani kum saati kullanıyor, olduğunu ekranda biliyor ama gördüğünde şaşırır ve dokunamaz. Belki korkar bile hatta. Sadece soyut üzerine çalıştı ve o robotik sese alıştığı zaman o hani sürekli aynı tonda konuşan şeye alıştığında, normal insanların konuşmasını da yadırgayabilir. Yani biraz gerçeklikten uzaklaşır, sürreal bir dünya olur büyük ihtimalle.

**G:** Aile açısından sence?

**P09:** Olumsuz yanları mı? Aile açısından olumsuz yanları yok denecek kadar az bence. Mesela çip takılı diyelim çok rahat, çünkü çocuklar artık normal davranabilecek. Ekrana bakıyor diyelim yine çok rahat olacak çünkü kendi işini kendisi görebilecek. O çocuğun ne yaptığı, ne ettiği hakkında endişelenmeyecek. Robot bakıyor diyebilecek, yine bir sorun yaşamayacak. Belki ne olabilir, belki o da bunu yadırgayabilir. Hani çocuğun çok teknolojiyle ilgilemesini istemeyebilir belki, belki robota güvenmeyebilir.

**G:** Zarar verebilir mi robot?

**P09:** Şöyle iki tane yolu var. Yine bilinçten geliyor. Bir tanesi bilinç, bilinçlenirse verebilir. Niye vermesin? Bilinçsiz olarak da çok spontane bir şekilde zarar verebilir. Yani kolu yanlış bir yere deyebilir. Başka bir şey olabilir ya da bir arıza verebilir. O an başka bir şey olabilir, değişebilir. Mesela robotun bir yapay zeka olacağı için bir şeyi vardır, internete bir yere bağlıdır. Birisi onu hackler. Hacklediği gibi de oraya şiddet şeyini yükler, öğrenciye şiddet uygular. Yani yapılabilir. Niye yapılmasın?

**G:** Evet. Peki bu özel eğitim özel gereksinimli bireylerin eğitiminde yapay zekanın geleceği sence nasıl şekillendireceğini düşünüyorsun?

**P09:** Bayağı bir katkısı olacağını düşünüyorum. Çünkü gerçekten ilk başta büyük bir kolaylık olarak gelecek bize. Daha sonradan yerimizi almaları da işte olumsuz tarafı olacak.

**G:** Peki sen bizim ülkemizi yapay zeka alanında nerede görüyorsun?

**P09:** Çok geride, görüyorum çok geride. Hem ulaşımı çok zor, hem ulaşımı kolay ama maddiyatı karşılayamıyor asla. Yani elinde bir iş, bir maddi durumun iyi olması lazım ulaşabilmek için. Onun dışında kafa yapısı olarak da henüz asla yapay zekaya entegre olabilmiş değiliz. Hiçkimse benim etrafımda açıkçası çok yapay zekayı bilen, kullanan kimse yok. Benim sadece ilgimi çok fazla çektiği için ilgileniyorum, ilgimi çekmese ben de muhtemelen ilgilenmezdim. Yani gerideyiz hocam.

**G:** Peki senin bu yapay zeka konusunda anlatmak istediğin bir anın, bir fikrin var mı?

**P09:** Yok hocam. Belli başlı bir şey yok. Genel sohbetlerim var. O da herkesle aynı. Ekstra fazla bir şey yok.

**G:** Peki benim sormak istediklerim bunlardı. Senin bu konuda eklemek istediğin var mı?

**P09:** Umarım yapay zeka çok kontrollü bir şekilde büyür. Yani gelecekte robotik savaşlar bizi bekliyor gibi hissediyorum. Umarım bu geleceği görmem.

**G:** Terminatör…

**P09:** Evet…Bekliyorum yani.

**G:** P09 teşekkür ederim.

**P09:** Rica ederim hocam.

**P10 — Interview Transcript**

**G:** Sana kısaca söyleyeyim önce. Görüşmemizi ses kaydı ile alacağım. Anonim olarak işlenecek, kimsenin kimliği belli olmayacak. Anonim transkript daha sonra makaleyle birlikte yan dosya olarak açık paylaşılabilir. Bunlara onam veriyor musun?

**P10:** Veriyorum hocam.

**G:** Teşekkürler, ilk soruma geçiyorum.

**P10:** Hiç kullanmadım hocam. Sizin dersinden sonra kullanmaya başladım. ChatGPT’yi kullandım. Sonra bir de projem için Runway uygulamasını keşfettim. Onu kullandım. Ne yapıyorsun,

**G:** Ne yapıyordun, neler yaptın?

**P10:** Videolar oluşturdum. Yazıyorum ben ne yapacağını, o kendi videoya dönüştürüyor. ChatGPT’de bildiğiniz Google gibi çok bir şey yok. Sadece hızlı cevap alabildim onun sayesinde. Runway daha iyiydi, tabi o ücretliyidi. Üç kere ücretsiz kullanma hakkımız vardı ama farklı e-posta adresinden girersek onlarda ücretsiz olabiliyor üç kere yine.

**G:** Anladım. Peki bu özel eğitimde yapay zeka kullanımı konusunda sen neler düşünüyorsun?

**P10:** Ben neler düşünüyorum? Evet, yani özel eğitim bence çok ihtiyaç duyulan bir alan. Yani yapay zekaya, özellikle de zihin yetersizliği için. Ben bunu düşünüyorum çünkü günlük yaşam becerilerine çok ihtiyaç duyuyorlar. Diğer yetersizlik türlerinde bu kadar değil bence. İyi kötü idare edebiliyorlar kendilerini. Günlük yaşam becerilerini, banyo yapma olsun, tuvalet becerisi olsun. Ama işte zihin yetersizliğine sahip öğrenciler daha zorluk yaşıyor. Bunun için de bence yapay zeka onlar için bir umut. Ben olsam öyle düşünürdüm. Yani eğer yeterli düzeyde kullanılırsa çok büyük fayda sağlayacağını düşünüyorum. Yani hayal edemeyeceğimiz kadar güzel gelişmeler olabilir. Yani yapay zeka sayesinde.

**G:** Peki bu yapay zeka özel eğitim sistemin içerisine nasıl entegre edilebilir sence?

**P10:** Hem öğretmen açısından hem de öğrenci açısından entegre edebiliriz. Mesela öğretmen açısından ben düşünüyorum şöyle etkinlik hazırlamak çok zor. Yapay zeka sayesinde bunu yapabiliriz. Yani kolay etkinlikler değil de çocuğun eğlenebileceği, kalıcı öğrenmeler sağlayabileceği etkinlikler kullanabiliriz. Yani hem kalıcı olur öğretimler hem de çocuk eğlenmiş olur. Böyle düşünüyorum.

**G:** Peki bu yapay zekanın özel eğitim içerisinde girmesi sence kolay olur mu, işlevsel olur mu?

**P10:** Ülkemizde biraz zor hala. Yani diğer ülkelere bakarak daha kötüyüz. Ülkemiz için biraz daha zor bence ama bence ileride olacak inşallah.

**G:** Böyle zorluğu nerede?

**P10:** Zorluğu nerede? Bence önemsenmiyor o kadar. Bir de öğrenciler de önemsenmiyor bence. Zaten bundan bir şey olmaz. Ne yapsak da bir çare yok. Öyle düşünüyorlar. Tıbbi açısından da bir çare bulamayınca eğitimle gidermeye çalışıyoruz ama eğitimle de bir şey yapılacağını düşünmüyorlar. Çocuklarda bu yüzden özgüvensiz, aile özgüvensiz.

**G:** Peki ileride bir gün bu yapay zekanın özel eğitim öğretmenini yerini alabileceğini düşünüyor musun?

**P10:** Düşünüyorum.

**G:** Nasıl olur?

**P10:** Nasıl olur? Daha yeni bahsettiğim gibi hani mesela videolar oluşturuyorlar ya, bunun için öğretmenin anlatmasına gerek yok ki. O videoları alır veli, aynı şekilde benim projemde de böyle geçiyordu. Onu tekrar eder çocuğuna. Diploma da verebiliyorsa hiç özel eğitim öğretmenine bile gerek kalmaz bence. Yani beceri öğretimi bence çok önemli zihin yetersizliğe sahip öğrenciler için. Çünkü bağımsız yaşaması lazım o çocuğun. Bunun için de beceri öğretimi çok önemli. Bu beceri öğretiminin içerisine bence okuma yazma da giriyor, günlük rutinler de giriyor.

**G:** Ama bu soyut bir şey. Nasıl yapacak, bir çocuğu nasıl kontrol edecek yapay zeka? Elini mi tutacak onun, ne yapacak?

**P10:** Dikkat becerileri zaten çok zayıf. O konuda bence özel eğitim öğretmenine ihtiyaç duyulabilir. Yani çünkü sürekli ekran başında olamaz o çocuk. Orada nasıl bir şey?

**G:** Daha geniş düşün.

**P10:** Daha geniş. Mesela evinde eğitim alabilir, okula gitmez. O açıdan iyi olabilir.

**G:** Daha da düşün.

**P10:** Daha ne düşüneyim?

**G:** Yapay zeka nasıl somut hale gelir sence?

**P10:** Öğretmenin klonlaması olabilir belki. Öğretmenleri böyle klonlarlar.

**G:** Olabilir mi?

Evet. Sizin bir klonunuz olduğunu düşünün. Ya da mesela böyle kendini tanıtmış öğretmenlerin klonlaması. Ben isterdim mesela yeni mezun birinden mi ders almak istersin yoksa bir hocadan mı? Ben bir hocayı seçerdim. Ben bir hocanın klonlamasını isterdim. Size gerek kalmazdı. O zaman sizin konumunuzu kullanırlardı. Mesela devlet açısından da iyi.

**G:** Klon mu olacak robot mu? Mesela klon deyince böyle etli olan kemiği olan mı yoksa bir robot mu diyorsun?

**P10:** Robot da olabilir evet. Yani bilmiyorum çok da. Yani sizin yerinizi tutmaz bir duygusu yok, bir şeysi yok.

**G:** Sence duyguları da olabilir mi onun ilerde?

**P10:** Çok çok ilerde bence çok zor. Robotun duyguları ya da bilmiyorum hayal edemiyorum yapay zekanın nereye gelebileceğini hayal edemiyorum şu an.

**G:** Peki bu yapay zeka ailelere, özel gereksinimli bir çocuğa sahip ailelere ne tür katkılarda bulunabilir sence?

**P10:** Günlük yaşam becerileri için kullanılabilir mesela. Ya ailenin zamanı yok. Sürekli çocuğuyla ilgilenmek zorunda. Aileye zaman kalır. Bu sayede çocuğuyla ilgilenmek zorunda kalmaz. Mesela bir yatağını toplamak olsun, bir banyo yapmak olsun hep aile ilgileniyor. Ama bir robot olsa, bununla uğraşsa aileye gerek kalmaz. Aile de kendi hayatına bakabilir. Ya da mesela aile öldüğünde bu çocuk ne yapacak diye düşünmek zorunda kalmaz. Ona bırakacağına emindir yani.

**G:** Robota?

**P10:** Evet. Aileyi tatmin edecek bir robot olursa eğer.

**G:** Her şey olabilir, hepsi olabilir. Bence de olabilir.

**P10:** Çok zor aile açısından. Ben olmasam o çocuk ne yapacak? Sürekli bunu düşünüyordur yani.

**G:** Yani yapay zekaya bırakabilir çocuğu diyorsun? Ya da yapay zekanın robotuna?

**P10:** Evet.

**G:** Peki aile öğretmen işbirliği nasıl sağlanabilir? Bu yapay zeka aile öğretmen işbirliğine destek verebilir mi sence?

**P10:** Evet verebilir hocam. Çünkü öğretmenler sürekli gönüllü olmak zorunda kalmaz. Öğretmenler böyle bir şey yapmaz. Yani taşın altına elini koymuyorlar. Herkesin bir beklentisi var ama robotun böyle bir beklentisi yok. Devlet açısından da iyi olur, aile açısından da iyi olur, yorulmaz da. Öyle bir şey de olmaz. Bence iyi olur.

**G:** Yani nasıl iyi olur ama iyi olması nasıl sağlanır sence?

**P10:** Yani eğitim konusunda sağlanır tabi ki.

**G:** O işbirliği,

**P10:** İşbirliği, ailelerin bilinçli olması lazım ama kullanması lazım. Aileye eğitim verilmesi lazım.

**G:** Peki yapay zekanın özel eğitim öğretmeni açısından düşündüğümüzde olumsuz tarafları ne olabilir?

**P10:** Mesela ChatGPT kullanırken bana yanlış bilgiler verebiliyordu. Çocuğa da yanlış bilgiler verebilir.

**G:** Öğretmene.

**P10:** Öğretmene mi? Yani yerine geçecek sonuçta. Öğretmen istihdam olmayacak. Bu öğretmenler ne yapacak ya da onlar yerine geçerse ne yapacak? Devlete yük. O yüzden işsiz kalabilirler.

**G:** İşsiz kalabilir evet.

**P10:** Aile için iyi olur bence ama öğretmenler için iyi olabileceğini düşünmüyorum.

**G:** Evet, aile için olumsuz tarafı ne olabilir sence?

**P10:** Aile için belki duyguları olması. Hani dediğimiz gibi robotun aile için bir olumsuz tarafı olmaz bence. Çünkü özel eğitim öğretmenini özel olarak tutuyorlar, sürekli bir para vermek zorunda kalıyorlar ve buna da tabi ki ücret öderler. Ama bilmiyorum bir öğretmene ödeyeceği kadar ödemez bence. Çok faydası olur aile için ama öğretmen için faydası…

**G:** Robotlaştığında diyorsun değil mi?

**P10:** Evet.

**G:** Peki özel gereksinimli öğrenci açısından olumsuz bir yanı olur mu?

**P10:** Özel gereksinimli çocuk için… Sıkıcı gelebilir bazen. Bence olumsuz bir tarafı olmaz. Aile içinde, öğrenci içinde. Ya da bir aile sıcaklığı isteyen, öğretmen sıcaklığını isteyebilir belki. O eksik kalabilir.

**G:** Etik açılardan nasıl olur?

**P10:** Etik açıdan yani bilmiyorum hocam. Etik açıdan bir şey gelmedi aklıma.

**G:** Peki bu yapay zekanın gelecekte özel eğitimi nasıl şekillendirebileceğini düşünüyorsun sence?

**P10:** Gelecekte mi? Yani çocuklar açısından şöyle çok ortam sağlayabilir. Çocuk için çok fazla ortam sağlayabilir. Duygu açısından mesela işitsel görsel destek sağladığı için kalıcı öğrenim sağlar. Yani çok fazla çoklu ortam sağladığı için kalıcılık sağlar. Çocuk için bunu söyleyebilirim. Aile için ne söyleyebilirim? Yani aile içinde dediğim gibi maddiyat açısından iyi. Bir de sürekli her an birine ihtiyaç duyuyor ya aile mesela bir sorun oluyor hemen ona sorabilir. Ama öğretmen her zaman müsait olmuyor. O her zaman müsait, yanında. O yüzden iyi olur. Öğretmen açısından işsiz kalacağı için bence hiçbir faydası yok yapay zekanın.

**G:** Peki ülkemizi bu yapay zeka açısından nerede görüyorsun?

**P10:** Daha doğum aşamasında gibi görüyorum hocam çok çok önce.

**G:** Diğerleri ile karşılaştırsan? Diğer ülkelerle, gelişmiş ülkelerle?

**P10:** Amerika, Almanya gibi. Onlar şu an büyüdü yani yaşlılığa gidiyor. Biz hala doğamadık öyle söyleyeyim.

**G:** Senin yapay zeka konusunda böyle anlatmak istediğin bir anın ya da söylemek istediğin bir fikrin var mı?

**P10:** Yapay zeka konusunda hocam inanın ben hiç bilmiyordum. Yani sizin dersinize gelmeden önce yapay zeka hakkında çok bir fikrim yoktu. Sizin dersiniz sayesinde öğrendim. Ve şunu öğrendim; bu çocuklar yapay zeka sayesinde bağımsız olabilirler. Bizim amacımız zaten bu ve sürekli öğretmen yanında olamıyor çocuğun. Ama yapay zeka her zaman hayatı boyunca yanında olacak. Ama bir öğretmen her zaman hayatı boyunca yanında olamıyor, istese de olamıyor yani. O yüzden çok faydalı olacak. Çocuk için çok iyi bence. Aile için çok iyi. Yani en çok da günlük yaşam becerileri açısından, becerilerin öğretimi konusunda yapay zekadan faydalanacağını düşünüyorum. Bu kadar hocam söyleyeceklerim.

**G:** Benim soracaklarım bu kadar. Senin eklemek istediğin var mı?

**P10:** Yok hocam. Teşekkür ederim.

**G:** Aferin.

**P11 — Interview Transcript**

**G:** Görüşmeye başlamadan önce kısaca söyleyeyim. Sesini kayda alıyorum. Söylediklerin anonim olarak işlenecek. Anonimleştirilmiş hâliyle transkriptin yan dosya olarak diğer araştırmacılara açık şekilde paylaşılabilir. Bunlara onam verir misin?

**P11:** Veriyorum hocam.

**G:** Çok iyi, başlıyoruz.

**P11:** Kullanıyordum hocam, ben zaten sürekli kullanıyorum. Ders için sizin verdiğiniz ödev var ya onun içinde yapay zekayı kullandım. Hatta bir çok aşamasında direkt oraya sordum. Güzel sonuçlar elde ediyorum. Böyle görseller için de yapay zekayı kullanıyorum. Çünkü bazen zihindeki şeyleri direkt yansıtamıyorsun karşıya. Yapay zeka görselini yapınca mesela daha anlaşılır. Onda da yaparken şey sorun olabiliyor. Yapay zeka insan gibi direkt anlattığınızı yorumlayamayabiliyor. Mesela yazılımcılar kodları eksik yazınca yanlış bir şey çıkıyor ortaya. Orada da tam net şekilde ifade etmeyince tam istediğini vermiyor yapay zeka. O yüzden ona dikkat ediyorum.

**G:** Peki özel eğitimde yapay zeka kullanımı konusunda ne düşünüyorsun?

**P11:** Özel eğitim… Özel eğitimde hocam şimdi gerekli yani kullanılabilir ve yararlı olacağını düşünüyorum. Yararları olacağını şöyle düşünüyorum. Şimdi çocukların ön hazır bulunuşluklarını, işte ölçmelerini değerlendirme, planlama, işte bunları veri olarak öğretmene sunup zamandan tasarruf ettireceğini düşünüyorum. Bunun yanında sağlıksal durumlarını da gözlenebilir hale getirir. Bu da öğretmen için bir kolaylık olur. Ondan sonra, atıyorum hani daha yapay zeka bu kadar gelişti mi bilmiyorum da belki de gelişmiştir. Şey, çocuk problem davranış göstermeden anlık önce ya da gösterirken vücudunda gelişen böyle işte kimyasal biyolojik şeyler onları mesela o hareketi yaptığında sürekli aynı şeyler yüksek seviyede çıkıyordur. Aynı hormonlar yüksek seviyeye çıkıyor. Onu da belki tespit edip öğretmene önceden bilgi verebilir.

**G:** Peki bu yapay zeka hangi yetersizlik türlerinde daha etkili olabilir?

**P11:** Zihinsel yetersizlikte daha etkili olur.

**G:** Neden?

**P11:** Çünkü özellikle de hafif orta olduğunda uyum sağlamaları daha kolay olur diye düşünüyorum. Problem davranışlarının az olacağını düşündüğümden dolayı. Uyum sağlama kolay olunca da daha hızlı, daha etkili olur.

**G:** Peki bu yapay zeka özel gereksinimli bireylerin günlük yaşamlarını nasıl etkiler?

**P11:** Günlük yaşamlarını öncelikle çocukların bir dezavantajını söyleyeceğim. Çocuklar belki sanal ile gerçeği ayırt edemeyebilirler. Hani ilk kullanılmaya başlandığında önce bir oryantasyon denir sanırım o süreç için de öğretmen gerekli zaten. Yapay zekayla işbirliği yapmadan önce, önce çocuğu alıştırmak lazım yapay zekaya. Alıştırdıktan sonra da dediğim gibi yine mesela biz sanal ortamda, yapay zekayla yaptık bazı şeyleri. Çocuk bunu yapay zekayla ilişiği kestikten sonra, normal hayata geçtikten sonra algılamayabilir. Ondan sonra yine orayı da isteyebilir. Gerçek hayatı istemeyip orada daha rahat hissettiği için orayı da isteyebilir, genellemeyedebilir. O yüzden orası biraz sıkıntılı.

**G:** Peki yapay zekanın özel eğitim öğretmene ne gibi katkısı olabilir?

**P11:** Aslında az önceki söylediklerimi, yani bundan bir önceki söylediklerimi çocuğu daha kolay tespit eder, problem davranışlarını daha hızlı çözer. Hatta çocuğun işte nerede ileri, nerede geri olduğu konuları yapay zeka tespit eder. Böylelikle önce zamandan tasarruf eder. Ondan sonra belki yapay zeka çocuğun geride olduğu şeylerde etkinliklere örnek sunabilir öğretmene. Daha hızlı, daha kolay yapar.

**G:** Güzel. Peki bu mevcut özel eğitim sisteminin içerisine yapay zeka nasıl entegre edilebilir?

**P11:** Öncelikle hocam biz özel çocuklar için düşünürsem zaten bireysel farklılık temel bizim bölümde. Direkt hepsine tek düze bir cihaz verilemez. Yani otizm de olsa bütün otizmliler aynı cihaz üzerinden kullansın ya da aynı programı kullansın diye bir şey denilemez. Onun için yine o bireye özgü olması lazım önce. Entegre etmeden önce. Zihin yetersizliği için falan da aynı. Dediğim gibi önce çocuğun geliştirilmesi gereken şeyleri yapay zeka ölçer, ona göre etkinlikleri sunar. Ondan sonra da uyum süreciyle beraber de geliştirirler.

**G:** Peki bu mevcut özel eğitim sisteminin içerisine yapay zeka sence kolayca entegre edilebilir mi?

**P11:** Nesnel anlamda, cihaz anlamında evet. Hani parasal, maddi durumları göz önünde bulundurmazsak alır koyarsın sınıfa. Ama bizim ülkede, benim hatırladığıma göre ben ortaokuldayken bilgisayar sınıflarımız vardı ortaokulda. Onları da devlet almış hocam, koymuş sınıfa. Koydun mu koydum. Ne yapıyoruz? Hiçbir şey. Ne yaptı, çocuk ne öğrendi bilgisayar dersinden? Hiçbir şey. Öyle oyun oynadık çıktık, internetten baktık çıktık. Aynı şey söz konusu olursa hiçbir şey olmaz.

**G:** Nasıl işlevsel olabilir?

**P11:** İşlevsellik de yine öğretmenlere bağlı ve hizmet içi eğitim verilir büyük ihtimalle öğretmenlere. Yoksa öyle cihazı aldım koydumla olacak iş değil.

**G:** Peki ileride bu yapay zekanın özel eğitim öğretmeninin yerini alabilmesi konusunda ne düşünürsün?

**P11:** Yani. Evet büyük bir kısmını belki alır ama bu tamamen öğretmenden bağımsız olabileceğini düşünmüyorum. Çünkü özel çocukların yönlendirilmeye bir şekilde ihtiyacı var. Onu kullanmayı, öğrenmeyi, ona alışma, ondan sonra işte üzerinden çalışma gibi şeylerde yönlendirilmeye ihtiyaçları var özel çocukların. Direkt öğretmenler belki bu sefer çocuğa yardımcı konuma geçebilir. İkisi arasındaki bağlantıyı sağlama açısından.

**G:** Robotlaşırsa?

**P11:** Yapay zeka robotlaşırsa? Bu seferde öğretmenler belki o programlar üzerinde çalışırlar. Öğretmenler programlar üzerinde çalışıp yine bir şekilde oraya el atarlar diye düşünüyorum. Çünkü dediğim gibi bireysel ya, bireysel olunca ister istemez her şeyi tespit ediyorsun, tamam. Ona yönelik de çalışmalar da sunuyorsun ama o çalışmaları da sonuçta bir insanın bir şekilde oraya yüklenmesi lazım. Verileri yani çalışma için, etkinlik için mesela internette olmayan bir şeyi yapay zeka kendiliğinden tamamen yüzde yüz uygun bir şekilde alıp da uygulayabilir mi? Sanmıyorum.

**G:** Peki bu yapay zekanın özel gereksinimli çocukların ailelerine ne gibi katkıları olabilir?

**P11:** Aileler, hocam genelde aileler şey oluyor ya böyle bıkmış. Hani çocuğunu seviyor tabi ki ama yani yorulmuş, bıkmış oluyor ya bence çok işlerine geliyor o konuda kendilerine zaman ayırma konusunda. Zaten şu anda bile işte rehabilitasyon merkezlerine falan gönderiyorlar. Ne diye? Bazıları çocukta çok da bir şey olmadığı halde gönderenler de var gözlemde gördüğüm kadarıyla. Hani kafa dinlemeye gönderiyorlar açıkçası. Özel gereksinimli çocuk varsa ailede bir de normal çocuk varsa büyük ihtimalle zaten özel gereksinimli çocuğa uyarlanmış yapay zeka normal gelişim çocuğa da çoktan uyarlanmış olur da aileler de rahat ederler diye düşünüyorum.

**G:** Peki aile öğretmen işbirliğine katkısı olur mu yapay zekanın?

**P11:** Aile, öğretmen işbirliğine... Belki de gerek bile duymazlar. Direkt yapay zeka öğretmen yani. Aileye direkt yapay zeka bildirebilir her şeyi. Çünkü yapay zekayla atıyorum sınıfta öğrendi çocuk. Evet sınıfta öğrendi. Evde de gidip aynı çalışmayı aile birkaç bir şeyle açıp çocuk orada çalışabilir. Böylelikle işbirliği pekişmiş oluyor. Doğru diyorsunuz. Yapay zeka bir araç olduğundan, evet.

**G:** Peki bu yapay zekanın özel eğitim öğretmeni açısından olumsuz tarafları neler olabilir?

**P11:** Yapay zekanın… Yani en büyük aklıma gelen daha sabah düşündük bunu, P09’le düşündük. Belki o da demiştir. En büyük şey aklıma gelen hani sanal ile gerçeği çocuk ayırt edemezse ne olur diye düşündüm. Yapay zekayla çalıştı, çalıştı, çalıştı. Ondan sonra öğretmen yapay zekayı konum dışı bıraktı. Çocukla iletişime geçmeye çalışıyor ama çocuk orada daha rahat ediyor ve onu istiyor. Bu sefer öğretmenle bağı koparmaya çalışabilir çocuk. Yönlendirilmesi zor hale de gelebilir. Öyle şeyler de mümkün olabilir. Olumsuz anlamda öyle. Olumlu anlamda da işte dediğim gibi.

**G:** Peki aile tarafından bakacaksak yapay zekanın olumsuz tarafları neler olabilir?

**P11:** Yani çocuğum önceden benimle az da olsa iletişime geçiyordu. Artık hiç geçmiyor gibi bir sorun çıkabilir. Beni görmek istemiyor gibi bir sorun çıkabilir. Yani ihtiyaç duymadığını düşünebilir çocuk gayet rahat bir şekilde. Bu öğretmen için de geçerli. Aile içinde geçerli. Bu sefer bağlarda bir zayıflık olabilir ki şu anda bile işte tabletle, telefonla normal çocukta bile görülüyor.

**G:** Peki bu yapay zekanın özel gereksinimli çocukların eğitimini gelecekte nasıl şekillendirebileceğini düşünüyorsun?

**P11:** Nasıl şekillendirir, denemeden bilemem. Önce bir denemek lazı çocuğun üzerinde. Bir ölçmek, denemek lazım hocam. İleriye de taşıyabilir. Çok hızlı. Mesela hafif zihin yetersizliği bir öğrenciyi alır. Atıyorum öğretmenle çalışsa 2 ayda kat edeceği yolu yapay zekayla belki 1 ayda kat edecek. Çünkü niye örnek bol, süre çok. Süre tüm günü nerdeyse, uyumadığı tüm günü ders şeyine dahil edilebilir. Yapay zekayla birlikte süre bol, etkinlik bol, ilgi alanlarını direkt ölçecek şekilde. İşte çeşitli dediğim gibi etkinlikleri. O yönden çok yardımcı olabilir.

**G:** Peki bizim ülkemizi yapay zeka açısından nerede görüyorsun?

**P11:** Şimdi yani hocam bizim ülkede daha çok destek verilmeli bence. Böyle olmaz hani. Bir benim aklıma gelen ve bir öğrencinin, bir başkasının aklına gelen şeyler bu ülkede bakanların, onların bunların aklına gelmiyorsa bu ülke nasıl gelişecek? Ya da benim aklımdaki şeyden bakan, ilgilenip de bir şekilde haberi olmuyorsa değil mi? Duyuramıyorsam kendimi nasıl gelişecek? Duyurdum diyelim. Mesela görüyorsunuz TÜBİTAK’da 9 bin lira nedir ki yani, 9 bin lira nedir? Yani ev kirası bile 10 bin lira. Hadi ne yapsın, ne yapmayı düşünüyorsun… Sen kendini geliştirmesen, gelişmek isteyene yardımcı olmazsan beyin göçüne mecbur kalırsın yani. Ondan sonra da işte gençleri işte belli akımlar üzerinden şey yaparsın, ekilersin empoze etmeye çalışırsın. Dersin ki işte sen milliyetçi değil misin, vatanını bırakıp nereye gidiyorsun? Sen Atatürkçü değil misin, sen şuncu değil misin buncu değil misin? Bunlar bana çok boş geliyor. Niye, sen tamam ben milliyetçiyim, ben Atatürkçüyüm …tamam sen nesin peki? Bana destek çıkmıyorsun. Sözde, lafta kalıyor. Çok havada kalıyor dedikleri. O zaman sen nesin? Sen demek ki milliyetçi değilsin destek olmuyorsan bir şeylere. Ya da yapanı öldürüyorsun ya da yapanı korumuyorsan öldürmekten öte korumuyorsan demek ki sende bir sıkıntı var, bende değil.

**G:** Senin bu yapay zeka konusunda anlatmak istediğim bir anın, bir fikrim var mı?

**P11:** Yapay zeka konusunda düşüneyim hocam. Sanki vardı gibi geliyor da şu an hatırlayamadım. Ya şöyle bir şey diyebilirim. Ben küçükken bu tür şeyleri çok düşünürdüm. İşte hani şöyle böyle şeyler olacak mı, hologramlar, şunlar bunlar olacak mı, insanların aklına gelmiyor mu, acaba çalışmıyorlar mı? Hani çocuğum ya ben zaten yapamıyorum, ben deyince hayali bir şey gibi oluyor. Ondan sonra acaba şu anda bunları çalışan düşünen kimse yok mu? Hani kocaman dünya illaki birileri çalışıyordur falan diye düşünüyordum. Hani büyüdükçe gördükçe dedim yapılıyormuş. Hani ben düşünürken demekki birileri bunlar üzerinde çalışıyormuş. Şu anda da bildiğimiz bilmediğimiz bir sürü proje geliştiriliyordur eminim. O yönden mutlu hissediyorum. Ve dersinizden önce de işte yapay zeka gündemdeydi aslında. Hani hoşa giden şeyler vardı ama sanki dersiniz ile beraber daha böyle içindeymişim gibi bir anda sanki bu yıl patlamış gibi bir his var içimde yapay zeka konusunda.

**G:** Sorularımız bunlardı. Senin eklemek istediğin var mı bu konuda?

**P11:** Hocam, yapay zekanın geleceğine dair bir şeyler eklemek isterim. Şimdi yapay zeka çok hızlı gelişiyor. Mesela diyorum ki P09’e, özel eğitime diyorum entegre edilmesi falan filan sence ne kadar sürer? Hani, şimdi şey konuştuk onunla da öğretmenler yaşlı olursa belki öğretmenlerin de adapte olması sorun olur. Hizmet içi eğitim biraz havada kalır gibi düşündük. Acaba dedik, hani ne kadar sürede dedik ilerler bunlar. Ben dedim ki biz şu an 20 yaşındayız. 10 yıl sonra hani tamamen bir şeyler olmuş olsa dedim biz 30 olmuş oluruz. Şu andaki atananlar da 35-40 oluyor dedim yani. Doğru hani yaşlandıkça acaba dedim. Sonra da dedim ki, P09 dedim bak bizim şu. An haberimiz var bu tür şeylerden. Biz bunun üzerine gelişip, bunlarla yaşayıp gelişeceğiz dedim. Yani ilerideki nesiller için yapay zeka zaten çoktandır var olmuş olacak onlara, atıyorum şu anki telefon gibi. Ama bizim için öyle değil, bizle beraber gelişiyor. Biz o doruk noktasını biz göreceğiz yani. Nasıl geliştiğini biz göreceğiz. O yüzden dedim hani öğretmenlerin o kadar havada kalacağını düşünmüyorum dedim. Yapay zekaya da işte yine öğretmen olsun, başka meslekler olsun uyum konusunda hani bilinçlenirse, yapay zeka bilinçlenirse demiştik ya öyle bir sorun olabilirse, gerçi sorun olarak değişir kişiden kişiye. Belki sorun olarak görmeyenler de olur. Bunun ya bir parçası olacağız yapay zekanın bir parçası olacağız. Ya onu üreten olacağız. En güvenlisi üreten olmak aslında. Parçası olmak da pek kurtarmayabilir. Ya da tamamen reddedip güvenli bir alan bulacağız yani. Bunu eklemek istiyorum. Tabi üreten olmak her zaman en iyisidir.

**G:** Bilinçlenirse olayın dışında nasıl kalacaksın ki?

**P11:** İşte…

**G:** Kalamazsın. O her yerde bulacaktır seni.

**P11:** Evet. Arar, tarar bulur yani çok zor bir şey değil. Dünyanın kaç kilometre olduğu belli sonuçta. O yüzde direkt parçası olacaksın. İçinden bir şey olacaksın yani.

**G:** O isteyecek mi?

**P11:** Belki isteyecek hocam. İşbirliği içerisinde olacaksın diyelim.

**G:** Ya da kimleri isteyecek? Herkesi isteyecek mi?

**P11:** Aynen öyle…

**G:** Seçecek belki de değil mi? Peki zihinsel engellileri, otizmlileri seçecek mi acaba?

**P11:** Seçebilir. Neden seçmesin ki? Çünkü tamamen kendi inşa ettiği, yapılandırdığı ve yetiştirdiği bir birey olmuş olacak. Eğtimle…

**G:** Ama kapasiteleri belli

**P11:** Tamam eğitir işte. Gelebileceği kapasiteyi görüyor zaten. Yani birkaç çiple yapacağını yaptırmak çok da zor bir şey olmasa gerek. Şu an benim bildiğime göre, ha onu da söyleyebilirim. Bildiğime göre bebekler tasarlanıyor. Tasarlanan bebekler var. Öyle laboratuarlar oluşturulmuş diye duydum. Hani akıllı bebekler, ondan sonra zeka durumunu seçiyorsun göz rengini seçiyorsun, boyunu posunu her şeyini seçiyorsun mesela..

**G:** O zaman zeka durumunu seçerken yani alt bir zeka seviyesinde olmasını istemez ki kimse

**P11:** Hiç kimse istemez. E öyle olunca da

**G:** Olmaz ki zihin engelli. Otizmli de olmaz.

**P11:** Evet

**G:** Tamamiyle böyle bir durum ortadan kalkmış olur.

**P11:** Evet tamamiyle dönüşmüş olur hocam. Öyle yani. Çünkü mevcut şu anda yapılıyor gördüğüme göre. Tabi ben bile gördüysem internette bu kadar dolaşıp ben bile gördüysem yapılıyordur. Belki de deney aşamasındadır. Hani büyüyene kadar. Onlar büyüyüp de gözlemlenene kadar sürülmeyecektir belki de bilemeyiz.

**G:** Evet.

**P11:** O yüzden bir 100 yıl içinde tamamen dönüşmüş olabilir.

**G:** O zaman özel eğitim öğretmenliği mesleği diye bir şey de…

**P11:** Kalmaz.

**G:** Kalmayacaktır.

**P11:** Evet onu da bulmuş olduk. 100 yıl içerisinde evet kalmayabilir. Ama işte o zaman da, o zaman göre meslekler belki olur diyeceğim. Ama her şeyi yapay zeka yapınca o da olmayabilir. Neyseki biz ölüyoruz o zamana, öyle bir şey olmamış oluyor.

**G:** Başka eklemek istediğin var mı?

**P11:** Yok hocam bu kadar.

**P12 — Interview Transcript**

**G:** Başlamadan önce sana hemen söyleyeyim. Görüşmemizi ses kaydına alacağım. Söylediklerin anonim olacak, isim olmayacak yayında. Anonim transkriptin makalenin yan dosyası olarak diğer araştırmacılara da açık şekilde paylaşılabilir. Bunlara onam veriyor musun?

**P12:** Evet hocam veriyorum, hiç sorun değil.

**G:** Teşekkürler, ilk soruma geçiyorum.

**P12:** Ben yapay zekayı hiç kullandım mı? Hocam kullandım her zaman kullanıyorum. Ders çalışırken kullanıyorum, araştırma yaparken kullanıyorum. Bu normal işte, ne var mesela… ChatGPT’yi araştırma yaparken kullandım. Bana büyük faydası oldu. Şöyle tek tek motorlara girip aramaktansa bana toplu bir şekilde veri elde etmiş oldu ve tüm dünya çapında bu verilerin analizi yapıldığı için ben kolay bir şekilde çalışmalarımı bulabiliyordum. Yani o yurt dışındaki bize verdiğiniz ödevlerle ilgili hocam ordan mesela yahut bi cinsel eğitim dersinde oradan tek tek benim araştırmamdansa zamanımı kolay bir şekilde tasarruf etmem konusunda yardımcı oluyordu ChatGPT. Aynı zamanda yine hocam sizin bana önerdiğiniz Evernote kullanıyorum ben. Orada da ben böyle tek tek not almaktansa, planlamaktansa orada benim ne yapacağım direkt bilgisayarın ekranında görünüyor. Bu bana kolaylık sağlıyor. Sonra yine o Canva’lar bunların hepsi aslında yapay zeka. O Canva’da hocam yine güncel şablonlarla birlikte hemen istediğim sunumu kendim oluşturabiliyorum. İşte yazıyorum oraya “şunu istiyorum, bunu istiyorum” diye. Hemen karşıma hayalimdeki o şablon çıkıyor. Yine bir projemde ChatGPT kullanmıştım. Orada da özgün resimlere ihtiyacım vardı. Çünkü bu projemde şey olabilirdi, Tübitak projemde Telif hakkı isteyebilirlerdi. Orada yapay zeka kendisi benim istediğim resimleri tasarladı. Çocuk resimlerini tasarlıyordu. Yine o ödevimde kullanmıştım.

**G:** Çok güzel.

**P12:** Sonra stajda çocuklarla oyun oynamak için Çocuk Akademisini ve WordWall kullanmıştım. Burada da oyun yoluyla çocuklara öğrenme programı hazırlıyordum. Bununla birlikte yine Duolingo var hocam. Yine onu da kullanmıştım. Orada da, oradaki yapay zeka da hocam, ben serimi orada unutuyorum mesela bakmıyorum, takip etmiyorum, o bana hatırlatıyor. Şu kadar süren kaldı diye ekliyor. Direkt giriyorum. Serinin devamını sağlıyordum. Yine işte beni orada pekiştiriyordu. Bunları gördüğümde hocam olumlu sonuçlar gördüğümde işimi kolay bir şekilde tamamladığımda yapay zekayı bu şekilde kullanmak beni teşvik etmişti. Daha çok kullanmak için internetten araştırma yapmaya başlamıştım. İşte işimi en kısa sürede nasıl halledebilirim diye.

**G:** Şimdi ben sana şey soracağım, peki bu özel gereksinimli bireylerin eğitiminde yapay zeka kullanımı konusunda neler düşünüyorsun?

**P12:** Özel gereksinimli bireyler içinde hocam mesela staja gittiğimde Otsimo kullanmıştım. Otsimo artık kendini çok geliştirmişti. Benim ilk daha önce tekrar, bir sene önce tekrar bakmıştım Bir sene önceki haliyle şuanki hali Otsimo’nun çok gelişmiş. Orada öğrencinin dikkatini çekemiyordum. Bana ilgisini yöneltemiyordum, beni dinlemiyordu veya bir öğretim yapmak istiyorum ona sosyal becerileri kazandırmak istiyordum ama dikkatini çekemiyordum. Benimle ilgilenmek istemiyordu. Söylediğim şeyler onun dikkatini çekmiyordu. Kullandığım kağıt kalem onun dikkatini çekmiyordu. Daha çok teknoloji ile ilgiliydi çocuk görsel şeyleri seviyordu. Ancak sıkılmıştı o klasik görsellerden. Ben de Otsimo’yu kullanmıştım. Çocuk onu görmüştü. O uygulamayı kullandığımda çocuk şey yapmıştı, oradan sürekli balonları patlatmayı öğrenmiş. İlk olarak ödüllü şeyi bitirdiğinde, okuma ödülün bir tanesini bitirdiğinde balonlar çıkıyor. Çocuk o balonları patlattıkça mutlu oluyordu. Ben burada şunu gördüm, yani nasıl diyeyim çocukların sürükleyiciliğini arttırıyor hocam öğrenme konusunda. Onun sosyal becerilerini destekliyordu. O da biraz konuşmaya başlıyordu. Çocuğun hem iletişim becerisini destekliyordu hem de sürükleyiciliği arttırıyordu. Sonra ne diyeyim bir şeyleri değerlendirirken… dur bi dakika ben çok genişten konuşacağım. Şimdi soru şeydi değil mi hocam yapay zeka bizde ne gibi şey sağlıyor özel eğitimde?

**G:** Hayır sen şunu söyle, yapay zeka sistemleri sence hangi yetersizlik türlerinde daha etkili olabilir?

**P12:** Aa tamam otizm ile zihinde olduğunu düşünüyorum hocam. Çünkü yapay bir zeka olduğu için bu çocuklarda karar verme, düşünme, akıl yürütme gibi şeyler daha sınırlı tipik gelişim gösteren bireylere göre. Ancak otizmli olan bir çocuk yapay zeka kullandığında oradan karar verme gibi düşünme gibi akıl yürütme gibi empati şeylerini bu yapay zeka aracılığıyla öğrenebilir diye düşünüyorum. Hem otizmde hem zihinde. Yine zihin yetersizliği olan çocuklarda da zaten zihinsel olarak sorun yaşadıkları için yapay bir zekanın onu desteklemesi ve bu yapay zekanın da 7/24 bu çocukları destekleyebileceği için mesela bir insan yorulur hocam bir yere kadar bir şeyler verebilir ve bir öğretmen de bir yere kadar bir çocukla çalışabilir. Birkaç saat çalışabiliyorlar. Ancak yapay zeka okuldan alındığında bu çocuklar 7 /24 sürekli destek alabilir hem zihin hem otizm.

**G:** Çok güzel. Peki sence bu yapay zekanın özel gereksinimli bireylerin günlük yaşamlarına ne gibi katkısı olabilir?

**P12:** Günlük yaşamlarına şöyle olabilir; örneğin hocam otizmli bir çocuk nasıl anlatayım iletişim becerileri desteklenir, sosyal etkileşim ve iletişim becerileri desteklenir. Aynı zamanda günlük yaşamlarında ritim yapıyorlar hocam bu çocuklar kendilerini tekrarlıyorlar kompulsif davranışlarla. Mesela daha önce bir çalışmada okumuştum. Çocukların koluna saat takıyorlardı ve bu saatte çocukların bu tekrarlayıcı davranışlarını görüyorlardı. O tekrarlayıcı davranışlarına bakarak da çocukların o davranışlarının söndürülmesi sağlanabiliyordu. Yapay zekanın çocuklara ne gibi faydası olur dediniz, değil mi hocam?

**G:** Günlük yaşamlarına…

**P12:** Günlük yaşamlarında, örneğin şöyle olabilir; mesela zihin engelli bir birey, zihin yetersizliği olan bir birey hep annesininin yanında, onu destekleyecek, sosyalleşmesini sağlayacak bir arkadaşı olmayabilir günlük yaşantısında. Çünkü o çocukla konuşmak istiyor. Kendisini ifade edebilecek, anlatabilecek kişileri istiyor. Ancak insanlar onları bazen dinleyemeyebiliyorlar. Yalnızlık çekebiliyorlar. Bundan dolayı mesela bir yapay zeka uygulaması ile birlikte bu çocuklar, güvenli bir yapay zeka uygulaması ile birlikte, veri güvenliği sağlanabilen, gizliliğin korunabildiği bir uygulama ile sohbet edebilirler. İletişim becerileri gelişebilir. Aynı zamanda çocuk mesela kaçmaz. Evden kaçmaz. Oradan yapay bir şekilde arkadaş edindiğinde çocuk zamanını geçirebilir. Ya da otizmli bir çocuk işte bezen insan duyuşsal uyaranlardan dolayı bazen doğal çevresinde nasıl tepkiler verebileceğini bilemiyorlar. Doğal çevresi onu anlayamayabiliyor. Çocuk da çevreyi anlayamayabiliyor. Bundan dolayı bu yapay zeka kullandığında çocuk mesela sözsüz diyelim ki sözlü dil becerisi yok otizmli çocuğun bu yapay zeka uygulamasını kullanıp işte su istiyor diyelim ki, suyu söyleyemiyor. Oradan kullandığı uygulamalar ile su istiyorum diyebilir. Yani bu hale gelebilir. Çocukların iletişim becerilerini destekleyebilir. Düşüncelerini aktarmaları yönünde kolaylaşabilir. Ardından devam edeyim mi?

**G:** Şuna cevap ver, peki bu yapay zekanın özel eğitim öğretmenlerine ne gibi katkısı olabilir?

**P12:** Öğretmenler için bir kurtarıcı olacağını düşünüyorum hocam. Çünkü öğretmenler bir sınıfta diyelim ki dört öğrenci var iki öğretmen var. Ancak her ne kadar iki öğretmen de olsa özel eğitimin bireyselleştirilmiş bir süreç olması gerektiği için yani öğretmenin her bir çocukla ilgilenebilmesi çok zor. Ve belli bir yere kadar ilgilenebiliyor. Bu yönden zaten öğretmenlerin zaman yönünden tasarruf sağlayacağını düşünüyorum. Aynı zamanda yapay zeka kullanarak öğrencilerin ilerlemesini, ilerlemesinin izlenmesini takip eder. Bu yönden işi kolaylaşır. Çünkü öğretmenin aynı zamanda da yapmış olduğu öğretim çocukta etkili oldu mu olmadı mı diye bunu değerlendirmesi gerekiyor. İzlemesi gerekiyor. Ancak burada bir yapay zeka aracı olsa çocuk bu uygulamaları kullansa oradan mesela uygulama geri dönüt verse öğretmene, öğretmen boş zamanlarında teneffüs aralarında bunu değerlendirse öğretmen burada çocuk için daha kolay şekilde performansını belirler ve çocukla değerlendirme yapmaktan, izlemesini takip etmekten ziyade öğretimine odaklanır. Aynı zamanda çocukların performansını iyileştirmesinde, işte güncel çalışmaları öğrenmesi yönünde öneriler sunar yapay zeka öğretmene. İşte her zaman her yerde öğrenciyi takip edebilir yapay zeka uygulamaları ile birlikte. Öğretmene rehberlik eder aynı zamanda bu konuda. Yine ebeveyn etkileşimini kolaylaştırır diye düşünüyorum hocam…

**G:** Onu soracağım zaten…

**P12:** Ha tamam. Yine öğrencinin nasıl diyeyim, verilerini kolay bir şekilde analiz eder. Otomatik yönden değerlendirir. Başka ne söyleyebilirim, sorunları gidermede ve bazen öğretmenin kafası karışabiliyor hocam. Yani bir şeye nasıl çözüm yolu bulacağını bilemeyebilir. Ancak öğrencinin bir sorununu yapay zekaya yazdığında ondan fikir alabilir, ona danışabilir. Bu şekilde kendisine bir yol haritası oluşturabilir. Hani o zaman ben şöyle yapayım diye oradan destek alabilir. Öğretimine odaklanır yine. Başka ne söyleyebilirim? Zamandan tasarruf etmesini sağlar. Öğretmenin stresini azaltır, iş yükünü azaltır. Başka şeyler düşünmektense mesela ben nasıl yapabilirim, hani çünkü her türlü öğrenciye yetemeyecek bu öğretmen. Ancak her bireyle öğretimde yapay zeka kullanırsa onları önceden değerlendirir sonra da öğretime odaklanır. Öğretmenin işini kolaylaştırır. İletişim becerilerini, aynı zamanda otizmli çocuklarda mesela hocam ilgi çekici resimlerle, videolarla bu materyal kullanımı ile çocuklara motive olduğunu görür. Genelde çocuklar motive olurlar görsel bir şeyler gördüklerinde. Öğretmen de oradan dönüt almaya başladığında mesela otizmli çocuklarda, özellikle otizmli çocuklarda öğretmenin de motivasyonu sağlar. Kendine olan güveni artar ve kendini daha çok geliştirmeye başlar diye düşünüyorum yapay zeka uygulamasının destekleri ile birlikte.

**G:** Peki bu yapay zeka sistemleri mevcut özel eğitim sisteminin içerisine nasıl entegre edilebilir?

**P12:** Hocam bence zaten bir anda entegre edilemez diye düşünüyorum. Şöyle olabilir belki bu; Öncelikle biz zaten yapay zeka denildiğinde halk arasında genel olarak bilmiyoruz çünkü görmedik. Benim bu saymış olduğum Canva, EverNot gibi uygulamarın bile belki yapay zeka olduğunu toplumumuzdaki çok kişi bilmeyebilir. Zaten üniversite okuyor birçok öğrenci ama üniversite okuyan öğretmen adayları bile belki yapay zekayı bilmiyor, nasıl kullanacaklarını bilmiyorlar. Bundan dolayı lisansta bize bence bununla ilgili eğitim verilmeli diye düşünüyorum İlk başta öğretmen adaylarına lisansta eğitim verilmeli. Yine bununla birlikte özel eğitim öğretmenleri ve öncesinde özel eğitim öğretmenleri ve üniversitedeki akademisyenler ve bu işin uzmanları sistematik bir şekilde görüşmeli bence ne yapabiliriz bu konuyu nasıl anlatabiliriz, öğrencilere nasıl açıklayabiliriz diye. Bir çalışma tasarısı oluşturulmalı bence yine bunun için öncelikle bir çalıştay nasıl diyeyim çalıştay, bir seminer, konferans gibi şeyler düzenlenmeli. Öğrencilere bu yapay zekanın ne olduğu, eğitimlerimizde nasıl kullanmamız gerektiği bize öğretilmeli. Biz zannediyoruz ki yapay zeka bir alet, bize bu alet gelecek, biz o aletleri düşünüyoruz ancak yapay zekanın elimizdeki telefonlarda bulunduğunu, bilgisayarlarda bulunduğunu bize gösterilirse, öğretilirse ve bu araçların bize ne olduğu söylenirse Aynı zamanda da bu araçlarla kullandığımızda nasıl sonuçlar almışız. Mesela yurt dışında kimler kimler ne kullanıyor, onlar işte bu araçları kullandığında bu avantajları sağlamışlar, bu olumlu dönütleri almışlar, işte bizim de ülkemizde bunları bunları kullanmalıyız. İşte bu dönütleri sağlar diye bize öncesinde bunlar öğretilmeli diye düşünüyorum. Bir de şey düşünüyorum biz şimdi mesela hocam, öğretmenler yapay zekayı kullandı diyelim ki bu sefer öğrendik, yapay zeka araçlarını öğrendik, biliyoruz hangi araçlar olduğunu da biliyoruz. ve bu da tartışma grupları oluşturulmalı bence. Ben bu yapay zeka aracını kullanıyorum işte bu dönüşü aldım çok iyi oldu öğrencimle birlikte Diğer öğretmenler de bunu görebilmeli. Tıpkı işte bir WhatsApp grubu gibi Telegram grubu gibi yapay zeka kullanan öğretmenler grubu olmalı. Orada yapay zeka seçim araştıran öğretmenler keşfetmeli aynı zamanda da kullandığı yapay zeka aracında da bir puan almalı bence öğretmen yani. mesela bu puanla da bir şey yapabilmeli. işte atıyorum pul gibi 10 tane puanın var sana 10 TL geliyor gibi. Hani öğretmen bu yapay zeka aracını kullandığında karşılığını almalı, ödüllendirilmeli bu şekilde entegre edilmeli diye düşünüyorum. Tam anlatabildim mi acaba bu kısmı?

**G:** Evet, çok iyi anlattın P12. Peki şunu soracağım, bu yapay zekanın Türkiye'deki özel eğitim sistemine entegrasyonu sence kolay olabilir mi?

**P12:** Türkiye'de hocam aslında yani gelişmekte olan bir ülkedeyiz hocam. O yüzden Hemen bir şekilde entegre olacağını düşünmüyorum. yani muhtemelen zor olacaktır ki şöyle, bir de şöyle bir sorun var hocam gizlilik ve veri güvenliği sorunu da var. Çocuk orada, çocuğun orada ki yaşantısı belki bu yapay zeka ile birlikte üçüncü şahıslar ile paylaşılabilir. Ya da çocuk bir yanlış yapıp kendi verilerini yanlışlıkla paylaşabilir. Yapay zeka alanında da gelişiminde sorunlar var. Şiimdi bunları düşünmeden direkt yapay zeka odaklı mı düşüneyim?

**G:** Evet sadece yapay zekayı düşün.

**P12:** Tamam, yapay zeka odaklı düşündüğümüzde yani dediğim gibi gelişmekte olan bir ülkedeyiz. hemen kolay bir şekilde entegre edilebileceğini düşünemiyorum. Aklıma bu çok gelmiyor çünkü bunlar için destek gerekiyor bunları yapabilecek olan araştırmacılar için desteklenmeleri gerekiyor. Belli bir ücret verilmesi gerekiyor, teşvik edilmeleri gerekiyor. Bu da ülkemizde biraz zor açıkçası. Yani şimdi konuşacağım ama bunlar döküme geçeceği için çok aktaramıyorum. Ülkemizde zor açıkçası hocam hemen entegre edilebileceğini düşünmüyorum. Ancak gelişmekte olan, gelişmiş olan bir ülke de olsaydık bu soruyu sorduğunuzda bunun için bir destek kesinlikle devlet ayarlamıştır, ayırmışlardır diye düşünürdüm. Ya da olmadı STK’lar işte bir şekilde ayarlar bu kolay bir şekilde entegre edilir diye düşünürdüm. Ama ancak ülkemizde bunun entegre edilmesi biraz zor olduğunu düşünüyorum ve şu da olabilir öğretmenler o zaman biz ne iş yapacağız gibi belki de halk arasında, kendi aralarında o zaman “biz ilerde ne iş yapacağız, bizim gelecek kaygımız var, biz ne olacağız?” gibi diye de kendi kendilerine zaman zaman bu düşünce de çıkabilir diye düşünüyorum.

**G:** İşte ben de onu soracağım şimdi sana tam. Peki, sence yapay zeka ileride özel eğitim öğretmeninin yerini alabilir mi?

**P12:** Hocam şöyle, şimdi eğitimciler zannediyor ki aslında bu yapay zeka bizim yerimizi alacak diye düşünüyor. Ancak bence yerimizi almaktan ziyade bizi tamamlayacağını ben düşünüyorum. Zaten özel eğitim öğretmenine her yerde ihtiyaç var. Yerini alabilir mi diye düşündüm, şöyle düşünüyorum hocam otizm mesela otizm bir spektrum, çeşitlilik. Hani bazı insanın aklının almayacağı bir şekilde seyrediyor bu çeşitlilik. Bir şekilde şey yapamıyorsun, karar veremiyorsun. Mesela birine karar versen çocuğun diğer otizmine karar veremiyorsun. Gösterdiği belirtilere karar veremiyorsun. Mesela şu an şöyle düşünüyorum; yapay zeka tam anlamıyla geliştiyse eğer tıpkı bir insan beyni gibi düşünüyorsa, düşünürse ilerde bizim yerimizi alabilecek diye düşünüyorum. Ama tıpkı bir insan beyni gibi geliştirilirse. Anında işte çözüm üretebiliyorsa, karar verebiliyorsa, karşıdaki kişinin duygularını anlayabiliyorsa, durumu yönetebiliyorsa… mesela bazen ben Siri’ye soru soruyorum, cevaplayamıyor. “Üzgünüm anlayamadım.” diyor. Mesela eğer bu noktada düşünürsem yapay zekanın daha henüz günümüzde yerimizi alacağını düşünmüyorum. Ancak tamamlayacağını düşünüyorum. Bize öğretimlerimizde destek vereceğini düşünüyorum. Fakat tam anlamıyla yapay zeka tıpkı bir insan beyni gibi çalıştığı noktada da bizim yerimizi alacağını düşünüyorum. Olabilir, mümkün her şey mümkün.

**G:** Peki yapay zeka kullanımının özel gereksinimli çocukların ebeveynleri ve öğretmenleri arasındaki işbirliğine nasıl katkı sunabileceğini düşünüyorsun?

**P12:** Şöyle düşünüyorum hocam; şimdi bizim kendi aile hocam illa ki derdini anlatmak istiyor öğretmenlere ve derdini anlatacağı insanlar istiyor. Ve derdini doğru kişilere anlatmak istiyor ki çünkü doğru yanıtlar alabilsin. Fakat öğretmenler de belli bir yere kadar aileyi dinleyebilir. Belli bir yere kadar aileye zaman ayırabilir. Öğretmen de aileyle konuşmak istiyor, derdini anlatmak istiyor. Ona danışmak istiyor, fakat öğretmenin de bunun için bazen zamanı olmayabilir. Öğretmen de bir yere kadar yetebilir. Burada ailenin öğretmeniyle iletişimi kolaylaştırması, işbirliğini kolaylaştırması açısından yapay zeka kullanılabilir. İşte örneğin mesela şöyle bir şey olsa; bir mesaj kutusu olsa oraya ailenin iletisini yazsa yapay zeka da orada yapay bir cevap versin, işte aileyi telkin edebilecek işte böyle o yanıtlardan bir şeyler çıkarıp uygun cevabı verebilecek, tıpkı Google’daki gibi bir dönüt programı olsa ailenin mesela öğretmen tam o mesajı bakmış, görme süresi ne kadar belli bir müddet de olsa içi rahatlamış olur. Kendini iyi hissetmiş olur. Çünkü bu noktada aileyi de anlamak gerekiyor. Öğretmeni de anlamak gerekiyor. Öğretmen bir yere kadar aileye zaman ayırabilir ancak yapay zekayı kullandığında aile ile öğretmenin iletişim ve işbirliği kolaylaştırılmış olur. Aynı zamanda da aile yaptığı çalışmaları, çocuğu ile birlikte yaptığı çalışmalar yapay zekada kaydedildiğinde öğretmen mesela aileyle direkt konuşacak zamanı olmaz, ancak başka bir boş müsait olduğu zamanda bu değerlendirmeleri kendisi izlediğinde yapay zekanın da basitleştirdiğini düşünüyorum tabi bu arada bu verileri, işte şunda şu kadar başarılı oldu diye bu sonuca ulaştı diye yapay zekanın basitleştirmiş bir şekilde analizlerini öğretmen okuduğunda, öğretmenin hem kafası karışmamış olur hem yorulmamış olur hem aileye çok fazla zaman harcayarak öğrencisine daha az zaman harcamama gibi bir durumu olur. Her iki durumda da hem aile için hem öğretmen için zamandan tasarruf sağlanmış olur ve iletişimleri de daha güçlü olmuş olur. Yani böyle ailenin şikayetlerinden daha çok belki de bu yapay zeka ile birlikte şey konuşurlar “bugün çocuğumla bunu yaptım, burada bu ilerlemeyi gösterdi, işte biz bu dönütü aldık, bunları bunları görüştük” hani yapay zeka da aileyi desteklese “bu dönütleri aldık hocam, işte bu şekilde oldu, biz artık şunu yapmayı düşünüyoruz, işte atıyorum 11. Seviyeye geçeceğiz, orada çok az kaldı çocuğun işte siz de izlediniz mi mesela uygulamaya girip dinlediniz mi, orada atıyorum çocuk Ufuk diyelim, Ufuk konuştu ilk defa bir tepki verdi, çok uzun baktı, göz kontağı kurdu oradaki çocukla” dese mesela ailenin de öğretmenle kurduğu sohbet farklılaşır, konu farklılaşır. Gelişim üzerine konuşmaya başlarlar artık dertlerinden değil de çocuğun gelişimlerinden konuşmaya başlanır. Ve mutlu bir aile olmuş olur mutlu bir öğretmen olmuş olur ve daha mutlu sonuçlar, çıktılar elde edilir yapay zeka aracılığı ile diye düşünüyorum.

**G:** Peki şunu da soracağım, bu yapay zekanın özel eğitim öğretmenine ne gibi olumsuz etkileri olabilir?

**P12:** Hımm olumsuz etkileri ne gibi olabilir… Mesela hocam şöyle olabilir özel eğitim öğretmenine… bunu biraz daha düşünmek istiyorum.

**G:** O zaman şuna cevap ver, yapay zekanın ebeveyne özel gereksinimli çocuğa sahip aileye ne gibi olumsuz etkileri olabilir?

**P12:** Şöyle olabilir hocam, yapay zeka her ne kadar yapay bir zeka olmuş olsa da yine de her ne kadar desteklenmiş güzel uygulamaları olsa da hala yapay zeka gelişmekte olan bir program ve burada en çok şeyden korkuluyor, gizlilik ve veri güvenliğinden korkuluyor. Çocuğun verileri başka bir şahısla paylaşılabilir. Ya da çocuğun resimleri paylaşılabilir. Çocuk yanlışlıkla bir şey yapabilir. Yine aynı şekilde de anlamlı yanıtlar üretmeyebilir yapay zeka. Aile yapay zekanın verdiği dönütleri anlamayabilir. Ya da aile yapay zekanın anlayabileceği bir şekilde uygun bir mesaj iletemeyebilirler. Bununla birlikte başka ne olabilir, bu iki şeyden düşünüyorum hocam

**G:** Peki özel gereksinimli çocuğa ne gibi olumsuz etkileri olabilir?

**P12:** Özel gereksinimli çocuk yönünden de hocam belki şey olabilir yapay zeka da belli bir yere kadar bu çocuğa, mesela özel gereksinimli çocuk sürekli o yapay zeka ile vakit geçirmek isteyebilir hocam. Burada bu kontrolü sağlayamayabiliriz. Çocuk yapay zekaya bağımlı kalabilir. Bir insanla iletişime geçmekten ziyade daha çok yapay zeka ile iletişime geçmek isteyebilir. Elindeki kullandığı o telefon çocuğa daha cazip gelebilir ve o aracı kullanmak için problem davranış sergileyebilir. Çocuğu olumlu olarak pekiştirelim derken belki de çocuk için kontrol sağlamadığımızda çocuk olumsuz olarak pekiştirilebilir bu süreçte diye düşünüyorum. Olumsuz olarak çocuğun olumsuz pekiştirileceğini düşünüyorum. Bağımlı olabileceğini düşünüyorum. Kontrolden çıkabileceğini düşünüyorum. Ya da yapay zekanın da belli bir yerden sonra bu çocuğu anlayamayacağını da düşünüyorum. Çünkü bir de şey var, bu yapay zeka da belli bir yerden sonra tehdit olarak gördüğünde yapay zeka kontrolden çıkabiliyor. Çocuğun verilerini, paylaşımlarını, görüntülerini, videolarını yapay zeka yanlışlıkla başka bir yerde paylaşılabilir. Ama özel gereksinimli çocuk için en çok bu çocukların bu araçlara bağımlı olabilecekleri demiştim. Çünkü gerçekten de bir şeylere bağımlı olduklarında hele ki otizmli çocuklar için bu çok büyük bir problem davranış oluyor, durduramıyoruz. Daha çok büyük krizler geçirmeye başlıyorlar.

**G:** Evet.

**P12:** Aile için de bu olacağını düşünüyorum aslında. Aile yönünden de çocuk kontrol edilemeyince ailenin stresi artacak. Sürekli telefon isteyecek, bir yerlere gitmek istemeyecek, hadi gel komşuya götürelim işte atıyorum aile evde olmayacak, gidecek bir yere diyelim ki pazara gidecek. Ama çocuğu da götürmeleri lazım. Çocuk evde tek başına kalamaz. Bu sefer de çocuk “ben gelmeyeceğim” diyecek. Bunun için direnecek. Bu sefer de aile için başka bir stres yükü olacak. Tamam çocuğun gelişimleri için çocuk desteklenecek ama bu sefer çocuk belki kontrolden çıkacak. Hep vaktini orada harcamak isteyecek belki de. Böyle düşünüyorum.

**G:** Peki bu özel gereksinimli çocukların eğitimlerini yapay zekanın gelecekte nasıl şekillendirebilir sence? Neler olabilir özel eğitimle yapay zeka birleşince?

**P12:** Bence hocam özel eğitimle yapay zeka birleşince hayat daha da kolaylaşacaktır diye düşünüyorum. Hem öğretmenler açısından hem araştırmacılar açısından hem aile açısından hem çocuk açısından. Diyelim ki daha da geliştirilmiş yapay zeka araçları olduğunu düşünüyorum. Diyelim ki işte erken dönemde çocukların tanınabileceği bir uygulama olsa mesela yapay zeka ile birlikte. Aile hastaneye gitmeden doktora gitmeden o değerlendirme süreçlerinden geçmeden, çünkü o değerlendirme süreci de gerçekten çok yorucu ve yıpratıcı hem tıbbi değerlendirme hem eğitsel değerlendirme. Bir de çocuğun değerlendirmesinden memnun kalmayıp tekrar bir değerlendirme yapılabiliyor. Burada hem öğretmen için iş yükü olmuş oluyor yani bu değerlendirmeyi yapan kişiler için hem de aile için hem de çocuk için yıpratıcı oluyor. Fakat etkililiği kanıtlanmış böyle bilimsel olarak kanıtlanmış uygun bir yapay zeka aracı olduğunda çocuk hiç doktora gitmeden o süreci yaşamadan, o kaygıyı yaşamadan direkt kendisi diyelim ki telefondan çocuğunu tanılayabilse mesela çok güzel olur. Bu şekilde ailenin stresi azalır. Hem de insanlar işlerine daha kolay bir şekilde odaklanmaya başlarlar. Çünkü hep bir değerlendirme hep bir değerlendirme olduğu zaman insanda belli bir yerden sonra yıpranıyorlar. O kaygı üzerine konuşulmaya başlanılıyor. Bu sefer verilecek eğitim üzerine konuşulmuyor. Bu da insan için stres oluşturuyor. Yine bununla birlikte ya çocukların tanılanmalarında çok büyük bir iş yükünü azaltacağını düşünüyorum yapay zekanın hem aile yönünden hem öğretmen yönünden. Bir de başka bir şeyler daha vardı aklımda. Soru şeydi değil mi hocam, yapay zeka ileride ne gibi katkı sağlar?

**G:** Evet, gelecekte neler olur?

**P12:** Evet gelecekte aile yıpranmadan çocuğunu evde tanılayabilir. Bu yönde katkı sağlar. Örneğin işte tıpkı işitme testi gibi hocam, işitme testini kendimiz yaptığımızda işitiyor muyuz işitmiyor muyuz öğreniyoruz. O süreci yaşamıyoruz mesela hemen kendimiz rahatlıyoruz, bu yönden rahatlık sağlar. Bununla birlikte başka da öğretmenin iş yükünü kolaylaştırır gelecekte ve nitelikli personel yetişir hocam. Mesela şimdi şöyle düşünüyorum. Bu işte personel yetiştiriyoruz ancak personel yetiştirmek için yetiştiriyoruz. Fakat yapay zekayı da bunun içine entegre ettiğimizde hem yapay zeka hem de bu yetiştiren öğretmenler beraber işbirliği yaptığında nitelikli bir iş gücü oluşur ve nitelikli bir eğitim süreci gelişir. Hem daha fazla insana ihtiyaç kalmaz. Ülke bu yönden maddi olarak rahatlar hem de aile rahatlar. Aile daha fazla, mesela bazı aileler örneğin rehabilitasyon merkezlerinde 850 TL’ye ders aldırıyor bir tane aile burada. Fakat oradaki, merkezdeki öğretmen çocukla sadece boyama yaptırıyormuş sanırım çocukla içerde. Aile boş yere o 850 TL’yi ödüyor. Ama çok güzel bir yapay zeka uygulaması olsa, o uygulama aileye dönüt verse, çok böyle yapılandırılmış güzel bir uygulama tıpkı bir insan gibi özel eğitimci gibi, aile o parayı oraya vermez. Oraya 850 TL’yi bir seferliğine verir, bir yıllığına ve çocuğuna daha dönüt alabileceği, somut dönütler alabileceği kendisinin hem çocuk hem kendisinin pekişebileceği bir uygulama ile karşılıklı hem ailenin maddi olarak kurtulmuş olup hem de orada çocuğunun gelişimlerini izler. Öğretmen için de böyle. Çocuğu kolay bir şekilde değerlendirebilir öğretmen. Çocukla şey yapmaktansa, değerlendirmektense direkt öğretimine odaklanmaya başlar. Hem işten hem ekonomik yönden hem de o psikolojik yönden yapay zeka bütün ülkeye, insanlara katkı sağlayacağını düşünüyorum açıkçası hocam.

**G:** Peki evet, ülkemiz yapay zeka alanında nerede sence?

**P12:** Ülkemiz yapay zeka alanında bence hocam gelişmeye başlamakta diye düşünüyorum. Yeni yeni gelişmeye başladığını düşünüyorum. Daha çok küçük küçük, yeni yeni. Bu sanki şey gibi düşünüyorum örneğin işte diyelim ki Almanya bizden on yıl sonra başladıysa benim ülkem on yıl geride gibi düşünüyorum. Onların on yıl sonra yaptıklarını biz şimdi yapmaya başlıyoruz gibi hissediyorum. Çünkü çok fazla görmüyorum, duymuyorum. Yapay zeka desteklenmiyor. Görülmüyor, bizlere anlatılmıyor. Bizler kendimiz Instagram’dan göre göre bu yapay zeka araçlarını öğreniyoruz ki zaten de şöyle örneğin ben aracı kullanmak istiyorum ancak onun şeyi yok Türkçe anlatan bir video yok mesela hocam. Gidiyordum kendim yazıyorum mesela diyelim ki Canva. Canva’yı kullanmak istiyorum ama kullanamıyorum öyle hocam programı kullanabileyim diye baktım nasıl program, çıkmadı. Türkçe bir şey göremedim. İngilizce metinler vardı. İngilizce videolar vardı. O İngilizce videoları dinleyip kendim bir şeyleri çıkarım yaparak mesela o programı nasıl kullanacağımı öğrenmiştim. Buradan da yola çıkarak şunu düşünüyorum ülkemiz yapay zeka alanında çok yeni yeni çalışmaya başlıyor. Yeni yeni, küçük küçük, minik minik. Dışarıdan, dış devletlerden, gelişmiş devletlerden bir şeyler görerek araştırmacıların kendi güdüsüyle kendi isteğiyle, araştırmacılar sayesinde, araştırmacıların bu istekleri sayesinde yapay zekanın gelişmeye başladığını düşünüyorum. Ülkeden ziyade buradaki araştırmacılar istekli bence yapay zeka için.

**G:** Peki sana şunu da soracağım; bu konuda senin anlatmak istediğin bir anın, fikrin var mı yapay zeka ve özel eğitim ile ilgili?

**P12:** Yapay zeka ve özel eğitim ile ilgili anım aslında vardır da. Düşünüyorum hocam. Ama fikrimi söyleyeyim, fikrim hocam yapay zeka mesela ben kendi açımdan söyleyeyim keşke bir çok güzel kullanabileceğim bir yapay zeka olsa. Ben mesela şu an yeni mezun olacağım lisanstan. Ancak diyelim ki bir çocuk olsa özel eğitim bölümünden mezun olduğum için bir aile benden özel ders vermemi isteyebilir. Fakat alanda yeniyim, bölümde yeniyim, sahada yeniyim. Mesela size bir yere kadar danışabilirim, diğer insanlara da bir yere kadar danışabilirim. Ancak güzel bir yapay zeka programı olsa, ben o araca danışsam o bana geri bildirim verse, neler yapabileceğimi onun hakkında konuşsam, çocukla yaptıklarımı ona anlatabilsem, bana fikir verse, öneriler verse, sorunlarımı o araçla halletsem, insanlarla uğraşmaktansa o aracın aracılığı ile çözsem benim için daha az yıpratıcı olur bu süreç daha az stresli olur. E streslenmekten çok eğitimime odaklanırım, çocuğun gelişimine odaklanırım. Yani eğitimlerimde yapay zekayı kullanmak isterim, çünkü işimi kolaylaştırıyor. Zamandan tasarruf etmemi sağlıyor. Bazen bir şey için sayfa sayfa geziyorum, on yere bakıyorum bir şeyi bulabilmek için. Ancak mesela bu ChatGPT’ye yazdığımda bütün verileri o kendisi araştırıyor. Özetlenmiş bir şekilde karşıma çıkarıyor. Ben tek tek o uygulamalara girmektense ya da işte o uygulamalar ücretli mi ücretsiz mi bunu da soruyorum, bunu da öğreniyorum. Direkt kendim orada üç saat harcamaktansa böyle bir on dakika beş dakika harcayarak zamandan tasarruf etmemi sağlayabiliyorum. Bu yüzden yapay zeka keşke daha güzel böyle uygulamalar olsa. Bunları bilsem, öğrenebilsem, bize de bunlar anlatılsa. Çoğu yapay zeka aracını ben kendim tesadüfen Instagram’dan duya duya bir şeyler okuya okuya, işte başka birisinden duyarak öğreniyorum. Bize bunlar öğretilmiyor, bilmiyoruz. Diyelim ki çok güzel bir şey, mesela bir soru sorma uygulaması var, ondan sonra bu çocuklarla şey yapma uygulaması var, ne diyim oyun hazırlayarak öğretim programı hazırlama WordWall gibi çocuk akademisi gibi. Mesela bunları biz lisansta bilmiyoruz. Bize bunlar söylenilmiyor. Bunları biz kendimiz buluyoruz. Mesela bunları kullandığımda da öğretim çok keyifli geçiyor. Çocuklar artık sıkıldı kağıttan kalemden. Geçen stajımda bir şey kullandığımda, internetten bir uygulama kullandığımda WordWall kullandığımda çocuklar çok keyif aldı dersten. Onların keyif aldığını görünce ben de keyif aldım, güdülendim, motive oldum. Daha çok bu uygulamaları araştırmaya başladım. Mesela çocuklar keyifleniyor ben keyifleniyorum. İşim kolaylaşıyor, bunlar bize lisansta öğretildi bir şekilde. Mesela seçmeli meslek bilgisi dersi olsa orada bunlar anlatılsa. Bize zaten hep Web 2.0 araçları anlatılıyor ama bu Web 2.0 araçları da eskidi artık. Daha güncellerini öğretmiyorlar. O güncel çalışmalar bize hep lisansta anlatılsa bununla ilgi de bir grup oluşturulsa bizim için güzel olur diye düşünüyorum, öğretmen adayları için.

**G:** Çok güzel P12. Şimdi benim sana sormak istediklerim bunlardı. Senin eklemek istediğin var mı bu konuyla ilişkili olarak?

**P12:** Eklemek istediklerim yok hocam şimdilik. Bu kadar. Konuşurken anlattım.

**G:** P12 verdiğin bilgiler için teşekkür ederim.

**P13 — Interview Transcript**

**G:** Hemen başlamadan söyleyeyim. Görüşme ses kayıt altına alınıyor. Söylediklerin anonim olarak yansıtılacak, ismin geçmeyecek hiçbir yerde. Anonim transkriptin daha sonra makaleyle birlikte yan dosya olarak açık paylaşılabilir. Bunlara onam veriyor musun?

**P13:** Veriyorum hocam.

**G:** Tamam, başlayalım.

**P13:** Tabi ki. Eee hocam yapay zeka aracı olarak ben birini kullanıyorum zaten. ChatGPT’yi örnek verebilirim. Sonuçlar çok iyi hocam. Çünkü şöyle bir sonuç elde ettim. Şimdi bilgiye ulaşma açısından çok kısa sürede ulaşılabiliyor öncelikle ChatGPT’ye. Detaylı bir şekilde ulaşılıyor. Şimdi biz Google'dan yazdığımızda veya başka bir arama sayfasından ulaşmak istediğimiz bilgiye, ulaşmak istediğimiz bilgiye hemen ulaşamıyoruz hocam. Kısa zamanda, çok kısa zamanda ulaşamıyoruz. Bunu yapay zeka bize çok iyi bir şekilde yapıyor hocam. Çok detaylı istediğimiz şekilde örneklendirerek ulaşmak istediğimiz bilgiye ulaştırıyor. Bir de yapay zekanın şu huyunu çok seviyorum hocam; yapay zekayla sohbet edebiliyorsun yani bir anı, hani günlük tutabiliyorsun oraya. Bugün soruyor, ne yaptın diye. Mesela başından geçeni anlatıyorsun oraya, unutmuyor da. Tam bir dijital günlük gibi. Çok işime yarıyor benim yani.

**G:** Anlıyorum.

**P13:** Her konuda, evet.

**G:** Peki bu özel gereksinimli bireylerin eğitimlerinde yapay zeka kullanılması konusunda ne düşünüyorsun?

**P13:** Hocam kullanılması gerekiyor zaten. Dijital bir çağda yaşıyoruz. Şu an, şimdi yapay zekanın kullanımıyla birlikte hani kullanılmaya başlanmasıyla birlikte kullanılmaya başlarsa ki bu Türkiye'de biraz zor ama bunu biraz düşünelim. Avrupa açısından düşünelim. Gelişmiş ülkeler açısından düşünelim. Şimdi biz entegre ettiğimizde özel gereksinimli bireyler için, bunu kullandığımızda şimdi hem öğrenci için kolaylık sağlıyor hem öğretmen için. Öğrenci için şöyle bir kolaylık sağlıyor; öğrenciyi odak noktasına alıyor. Yani her öğrenci için farklı yöntemler, farklı tarihler, farklı şekilde oyunlar yapabilir yapay zeka. Öğrencinin odağını bir yere toplayabilir. Bu herkes için kolaylık sağlayabilir hocam ben böyle düşünüyorum yani.

**G:** Peki yapay zeka hangi yetersizlik türlerinde daha etkili olabilir sence?

**P13:** Hangi?… Güzel soru hocam. Eee ben otizm diyorum hocam. Otizm ve zihin yetersizliği diyorum. Sebep söyleyebilirim.

**G:** Neden?

**P13:** Şöyle hocam; çünkü biz bireye bağımsız, temel özel gereksinimi olan bireylere temel kazandırmak istediğimiz beceri “bağımsız yaşama becerisi”. Hani biz özel gereksinimli bireylere bağımsız yaşayabilme, kendi kendilerini yönetebilme, kendi davranışlarını kendileri istendik şekilde meydana getirmelerini istiyoruz biz sağlamalarını. Şimdi biz işitme yetersizliği, görme yetersizliği veya diğer türleri düşündüğümüzde biz yapay zekayı işitme yetersizliği için nasıl kullanabiliriz ki hocam? Hani daha kolaylık sağlayalım? Yani şimdi işitme yetersizliği de zaten öğrenciler işitme cihazları kullanıyorlar, dijital işitme cihazları dediğimiz işitme cihazları kullanıyorlar. Zaten şu an bu işitme olsun, görme olsun, hafif görme, hafif işitme… bunlar yapay zeka için yapay zeka için diyorum yanlış bir tabir oldu. Yapay zekayı kullanarak bunlara farklı bir yöntem, farklı bir strateji geliştirileceğini düşünmüyorum hocam. Çünkü zaten bizim gibi duyuyorlar işitme cihazları sayesinde, normal gelişim gösteren bireyler gibi duyabiliyorlar. Sırf işitme cihazları olduğu için. Görmede de öyle hocam, görmede de hafif görmede de gözlük yani dediğimiz gibi zaten belli yöntemler var. Biz bunlar dışında farklı bir şey yapamayız hocam yapay zeka kullanarak bunlar için hani görme veya işitme olsun. Ama biz otizm için zihin yetersizliği için yapabiliriz. Yapay zekayı kullanarak şimdi şöyle bir temel bir sorun çıkıyor ortaya. Şimdi dijital teknoloji hızlı bir şekilde geliştiği için şeyi soracaklar; teknolojinin gelişmesiyle hızlı bir şekilde yapay zekanın, zihnin yetersizliği engellenebilir mi? Bu böyle bir sorun ortaya çıkıyor. Önüne geçilebilir mi? Böyle bir soru var. Şöyle bir cevap var. Şimdi etik olmuyor. Yani etik açıdan, uygun olmuyor otizm ve zihin yetersizliği için. Hani önüne geçilebilir mi? İle ilgili. Biz zaten, bizim amacımız önüne geçmek değil yani. Çünkü dediğim gibi etik olmuyor hocam. Ahlaki sorunları. Çünkü insanlar şu anda…

**G:** Şimdi bu yapay zekanın kullanımı, özel gereksinimleri olan çocukların günlük yaşamlarına ne tür katkılarda bulunabilir?

**P13:** Evet. Hocam öncelikle şimdi özel gereksinimi olan bireyler yapay zekayı kullanmayı öğrenebildiğinde şimdi yapay zekanın desteğini de aldığında aslında şöyle diyebiliriz. Şimdi ev açısından düşünelim. Özel gereksinimi olan bireyler evde daha rahat vakit geçirebilirler ya. Şimdi bu öğrenciler, genelde normal gelişim gösteren öğrenciler tarafından dışlanıyor zaten bu bir gerçek. Her tarafta bu gerçek. Dışarıda bile yolda yürüdüğünde bile çocuğun dışarıdaki bakışların nasıl olduğunu biliyoruz. Şimdi yapay zekayı kullanmayı öğretsek veya teknolojiye çok hızlı bir şekilde gelişirse yapay zeka desteğini sağlasak evde çok iyi bir şekilde vakit geçirebilir hocam. Hani dışarıda vakit geçirmek zorunda değil veya kurumlara gitmek zorunda olmayabilir. Yapay zekanın evet bu yönden iyi olacağını düşünüyorum.

**G:** Peki bu yapay zeka kullanımının özel eğitim öğretmenine ne gibi katkısı olabilir?

**P13:** Katkısı olabilir hocam özel eğitim öğretmenine kolaylık sağlar. Düşünsenize yapay zekanın sizin yerinize BEP hazırladığını. Sizin yerinize stratejiler geliştiriyor. Bu bir taraftan bakınca iyi bir şey hocam.

Bir taraftan bakınca çok iyi bir şey. Şimdi şöyle bir şey var. Siz, insan, okuduğu bölümden mezun olduğunda dört dörtlük mezun olamıyor, illa eksikleri oluyor. Eğitim süresinde hani, eğitim verdiği süre içerisinde illa öğrencinin, öğretmenin, hani mezun olmuş öğretmenin eksikleri oluyor. Şimdi sizin de dediğiniz gibi şöyle bir sorun çıkıyor, öğretmenin bilgi eksikliğinden dolayı dediğim gibi herkes dört dörtlük yetişmiyor. Bu şey sorununa sebep oluyor, şimdi öğrenciye eğitim verdiğinde nasıl eğitim vereceğini unutmuş olabiliyor. Bilmiyor olabiliyor. Bu da tekrardan üniversite hocalarını arıyor, arkadaşlarını arıyor. Zaman kaybı. Bir arayış içine giriyor, arayış içine. Şimdi biz yapay zekayı kullanalım orada, gerek kalmayacak ne arkadaşlarını aramasına, ne hocalarını aramasına, ne kitabı açıp o bilgiyi o sayfalar arasından bulmaya gerek kalmayacak. Yapay zeka farklı bir yöntem, farklı bir strateji geliştireceğini düşünüyorum eğer biz bunu kullanabilirsek.

**G:** Anlıyorum.

**P13:** Ben böyle düşünüyorum.

**G:** Peki bu yapay zeka sistemleri mevcut özel eğitim sistemlerinin içerisine entegre edilebilir mi?

**P13:** Edilemez hocam. O da şöyle diyeyim…

**G:** Nasıl edilebilir?

**P13:** Ben edilemez dedim. Nasıl edilebilir? Öncelikle hocam bizim özentiliği bırakmamız lazım. Şimdi özentilik derken Türkiye hocam hani gelişmiş bir ülke olabilmek için bence, bana göre yani, Avrupa’ya özeniyor hocam yani direkt Avrupa’dan aldığını uyguluyor. Kendi ülkesindeki gereksinime göre, gereksinimlere göre, ülkesindeki ihtiyaçlara göre bir sistem üzerinden değil de direkt aldığı gibi uyguluyor. Biraz içi boş denilebilir bunun. Önce nasılı, kendimiz kendi ihtiyaçlarımıza göre, ülkedeki gereksinimlere göre hocam biz özel eğitim yönetmeliği yapsak hani direkt entegre edebilsek ihtiyaçlara göre sistem kursak. Bir de şöyle bir şey var, kullanabilmemiz için her okulda bir dijital cihazın olması gerekiyor. Şu an doğuya gittiğimizde veya doğuya da gitmeye gerek yok. İç Anadolu'da bazı okullarda hala ? olduğunu biliyoruz hocam. Oradan başlamamız gerekiyor. En temelden ihtiyaçlara ve ülkenin ihtiyaçlarına göre bir sistemin kurulması gerekiyor. Pek özenmemiz gerekiyor.

**G:** Peki bu yapay zekanın şu an ki özel eğitim sisteminin içerisine entegre edilmesi işlevsel olur mu?

**P13:** Olmaz edemeyiz bence. Öyle düşünüyorum.

**G:** Peki sana şunu da soracağım, ilerde yapay zekanın özel eğitim öğretmeninin yerini alabileceği konusunda neler düşünüyorsun?

**P13:** Hocam şimdi ben buna katılıyorum. Şimdi tarihe bakalım biraz. Tarihten bugüne hep öğretmen öğrenci ilişkisi olmuştur. Usta çırak ilişkisi olmuştur. Bir insan, başka bir insan, öğrenci de bir insan olmak üzere. Şimdi bu ilişkinin ben açıkçası yüz yıl sonra sona ereceğini düşünüyorum. Şimdi hayatta gerçekler var hocam. Savaş çıkabilir. Her şey olabilir dünyada . Ama biz olmadığını varsayalım. Hani bir dünya savaşının veya başka bir şeyin gelmediğini varsayalım. Ben açıkçası yapay zekanın öğretmen yerine geçebileceğini düşünüyorum. Çünkü şimdi öğretmen dediğimiz insan her şeyi yeterince veremiyor, yeterince veremiyor. Çünkü insan kusurludur. Ama yapay zekanın ben bunu açıkçası daha kusursuz bir şekilde yapabileceğini düşünüyorum. Bir de şu an gelişen teknolojinin hızlı, çok hızlı gelişi, yüz yıl sonra insan görünümlü robotların eğitim verdiğini düşünüyorum ben açıkcası. Olabilir hocam, niye olmasın ki? Sadece insan öğretirken işin içine duygular giriyor. Yani olumlu, pozitif duygular. Ama ben robotların daha iyi yapacağını düşünüyorum. Olacağını düşünüyorum, yüz yıl sonra en az.

**G:** Anlıyorum. Peki bu yapay zeka özel gereksinimli çocukların ailelerine ne gibi katkılar sunabilir sence?

**P13:** Hocam şöyle sınıfta da konuşmuştuk. Bir arkadaşımızın projesi vardı. Ülkeler arasında aileleri birleştirme projesi. Düşünsenize yapay zeka, canlı tercümanlık yapıyor. Eee, Amerika'daki bir aileyi Türkiye'deki bir aileyle görüştürüyor. Bu çok iyi olurdu. Yani yapay zekanın bu açıdan güzel bir şeyi var. Yani katkısı var. Şimdi ben kendi penceremden düşünüyorum zaten düşünüyorum yani düşünemediğim, illa ki göremediğim illa ki düşünceler olabilir ama genel anlamda bakarsak yapay zeka ailelerin yapması gereken işleri kolaylaştıracak. Az önce dediğim gibi düşünsenize Türkiye'deki bir ailenin, Amerika'daki bir aileyle görüştüğünü? Ya da evdesiniz şimdi özel gereksinimli bir çocuğunuz var. Evdesiniz. Hiç bir şey bilmiyorsunuz hocam. Hani öğretmen ödev vermiş, onu yaptıracaksınız. Ama bir strateji, çocuğu ödevin başına oturtamıyorsunuz. Yapay zekanın olduğunu düşünelim burada. Bu işin içerisinde. Yapay zeka işin içerisinde olduğunda farklı bir yöntem, strateji veya öğrencinin velisi yapay zekayı kullanmayı bildiğinde yani çocuğu ödevinin başına veya yapması gerekeni yaptırmak için uğraşmaya gerek kalmayacak bence. Bu açıdan düşündüğümüzde ailelere birden çok katkısı var hocam. Ailede rahatlık sağlıyor diyelim. Yapılması gerekeni yapıyor yani. Böyle düşünüyorum. İllaki düşünemediğim vardır bazı şeyler ama…

**G:** Peki bu yapay zeka özel eğitim öğretmeni ve aile işbirliğine ne gibi katkılar sunabilir?

**P13:** Ne gibi katkılar sunabilir? Hocam, yapay zeka, öğretmen ve aile işbirliğini şöyle düşünüyorum hocam bu olur mu, bir düşüncem var bunda. Yani şimdi yapay zekayı kullanarak biz, öğretmen veli ilişkisinde kullansak yapay zekayı, yapay zeka üzerinden öğrencinin durumu takip edilse, öğretmen-veli ilişkisi sohbet oradan kurulsa mesela, hani yapay zekanın yardımıyla, yapay zekanın kontrolü altına. Tabi yine öğretmenin kontrol altında olacak ama velinin, öğrencinin davranışları yapay zekanın kontrolü altında olsa diye düşündüğüm şeyleri. Açıkçası daha iyi olur hocam. Çünkü şu an baktığımızda incelediğimizde çoğu öğretmenin, öğrencinin velisi ile iyi bir olumlu bir ilişkileri olduğunu göremiyoruz. Yani olumlu bir ilişkiden kastım; şimdi çoğu öğretmenler biraz affedersiniz hocam, hani yalakalık yapabiliyor. Sırf velinin gözüne iyi görünebilmek için. Olmaması gerekenin üstünde laflar söyleyebiliyor. Mesela öğrenciniz direkt net ve basit cevaplar vermiyor da hani öğrenciniz bu şekilde, böyle ilerliyoruz demiyor da, daha iyi velinin gözüne gözükmek için farklı bir yoldan yani. Yapay zeka kullansak burada yapay zeka, öğrencinin durumunu takip etse öğretmen tarafından kontrol edilip veliye bildirilse bence daha iyi olur diye düşünüyorum hocam. Biraz lafı uzattım ama kusura bakmayın.

**G:** Anlıyorum. Peki yapay zekanın sence özel eğitim öğretmenine ne gibi olumsuz etkileri olabilir?

**P13:** Evet, güzel bir soru. Az önce dediğimde şöyle bir şey demiştim. Yapay zekanın BEP hazırladığını düşünsenize, diye bir tabir kullanmıştım hocam. Şimdi böyle bir şey olduğunu varsayalım. Yapay zekanın özel eğitim öğretmenlerine bir bireyselleştirilmiş eğitim programı hazırladığını varsayalım, öğrencinin gereksinimlerine göre. Şimdi bu öncelikle öğretmeni rahat alıştırır hocam. Öğretmeni rahata alıştırır. Bir de öğretmen BEP geliştirirken sorunlara odaklanır, hani çözüm odaklıdır. Belli test, analiz yapma gibi becerileri vardır bu öğretmenin. Yapay zeka işin içinde olursa açıkçası öğretmen bu becerileri biraz zayıflar. Test yapabilme, analiz yapabilme… rahata alışır. Bir de şimdi şöyle bir düşünce oluşur öğretmende; ya zaten yapay zeka yapıyor, ben yapmasam da olur düşüncesi. Birazda düşünceleri köreltir. Yani insanın kendini geliştirmesi için biraz düşünmesi, aktif olması lazım. Yapay zeka bu konuda çok kötü olabilir. Yani kötü olumsuz bir tarafı olabilir. Bu şekilde yani hocam.

**G:** Peki yapay zekanın aileye ne gibi olumsuz etkileri olabilir?

**P13:** Aileye ne gibi olumsuz etkileri olabilir? Bu şekilde şimdi aile de aynısını düşünüyor olabilir. Aile düşünüyordur; ya hiç iyi bir öğretmen yok. Hiç kendini iyi bir şekilde geliştirmiş öğretmen yok. Ben en iyisi yapay zeka kullanayım deyip yapay zekayı kullanır. Şimdi bazı yapay zeka programları var. Yanlış programlanmış. Bu yapay zekaları kullandığında da ister istemez olumsuz bir sonuç elde edebilir. Bu yönden kötü olabilir aile için. Yapay zekanın bence öğretmen, şu an içinde, önümüzdeki yüzyıl bilmem ama şu an, şu önümüzdeki on yıl içinde veya yirmi yıl içinde yapay zekanın öğretmen eşliğinde kullanılması gerekiyor. Veliler açısından da olumsuz tarafı bu işte. Veli gerekli bir öğretmen ve yeterli bir öğretmen bulamadığını düşünüp yapay zekayı kullanmayı öğrenmeye başladığında bu sıkıntı olabilir. Evde düşünsenize hiç, hafif derecede zihin yetersizliği olan bir öğrenciniz var, 6 yaşında. Bir veli için bu bir eğitimini bir öğretmene vermiyorsanız da direkt evde yapay zekayı kullanıyorsunuz ve yanlış programlanmıştır. Bu iyi bir şey değil, çok kötü bir şey hocam.

**G:** Peki özel gereksinimli çocuğa ne gibi olumsuz etkileri olabilir yapay zekanın?

**P13:** Arkadaşlık ilişkileri, sosyal ilişkileri hocam. Şimdi biz otizmden gidelim. Aslında diğer gereksinimi olan diğer bütün çeşitleri var. Şöyle düşünelim; biz özel gereksinimli bireylere aynı zamanda iletişim kurabilme, sosyal iletişim dediğimiz sosyal becerilerde olumlu bir şekilde gelişmesini istiyoruz. Şimdi yapay zekanın olumsuz bir yönü şurada var; özel gereksinimli birey şey diyebilir, yapay zeka benimle sohbet ediyor yapay zeka beni anlıyor. Ben yapay zekayı kullanırım, vakit geçiririm diyip insan ilişkilerinde çok yetersiz kalabilir. Sürekli evde olduğunu düşünün bu bireyin, 20 yaşında hafif zihin yetersizliği olan bir bireyin yapay zekayı kullanmayı öğrenip veya yapay zeka eşliğinde vaktini evde geçirir. Dışarıda arkadaşlarıyla değil. Veya kendi gelişiminde olan arkadaşlarıyla veya öğretmeniyle veya ailesiyle iletişimde olmayıp sürekli odasında yapay zeka etkisinde olduğunu düşünürsek o açıdan kötü. Ekrana bağlı yaşayabilir. Bu şimdi aklıma geldi. Bir de dediğim gibi rahata alıştırır. Evden çıkmaz. Sağlık sorunlarına problem olabilir. Bir de dediğim gibi insan ilişkilerinde arkadaşlık ilişkilerinde olsun, dostluk veya markete gidip ayaküstü bir sohbet etme, yoldan geçen tanıdığın birine selam verebilme… bu konu gibi becerilerde biraz problem yaşar. Bu yapay zekanın olumsuz taraflarından biridir hocam. İlla vardır birkaç tane daha, şu an aklıma gelmiyor.

**G:** Peki sen ülkemizi yapay zeka konusunda nerede görüyorsun?

**P13:** Ülkeleri mi hocam?

**G:** Bizim ülkemizi, Türkiye’yi?

**P13:** Hocam çok yetersiz görüyorum. Bayağı geri. Şimdi kesin bir sayı veremem ama biz Avrupa’nın çok gerisindeyiz yani. Avrupa’nın, Japonya’nın bayağı gerisindeyiz. Biz hala şu an, bizim ülkemizin robotlar geliştirmesi lazım hocam. Şu an bizim ülkemizde yapay zekanın gerçekten şu an Japonya’da yapılıyor hani Avrupa’da kullanılmaya başlandı. Yapay zekanın açıkçası eğitimde kullanılması gerekiyor. Akıllı tahtalarda en az bir ChatGPT’nin kullanılması gerekiyor yani. Biz çok gerideyiz. Biz bayağı gerideyiz. Çünkü benim arkadaşlarımdan çoğu kullanmayı bilmiyor yani. Sınıfta görüyoruz, yani ben sınıfta soruyorum. Bana diyor, ne yapacaksın P13? ChatGPT ile sohbet ediyorum. Adam şey diyor; ChatGPT ile sohbet edilir mi ya? Böyle bir olumsuz tavrı var. Kendi de biraz konuşsa orada bir şeyi anlasa, oranın güzelliğini anlasa… böyle bir algı var Türkiye’de. Zaten gördüğümüz gibi bayağı gerideyiz hocam ya. Kötü durum, çok kötü.

**G:** Peki senin bu yapay zeka konusunda anlatmak istediğin bir anın veya söylemek istediğin bir fikrin var mı?

**P13:** Söylemek istediğim bir fikrim var hocam. Ben şunu demek istiyorum. Şöyle bir ekleyeyim hocam. Yapay zeka bence herkesin kullanması lazım yani gerçekten. Bu kişisel gelişim için de çok önemli. Ve insanın kendisini anlaması için de bence çok önemli. Ben bunu eklemek istiyorum hocam. Kullanabilmeyi öğrenen herkes kullansın.

**G:** P13 benim sana soracaklarım bu kadardı. Senin eklemek istediğin var mı?

**P13:** Eklemek istediğim bir şey yok hocam teşekkür ederim.

**P14 — Interview Transcript**

**G:** Görüşmeye başlamadan kısaca söyleyeyim. Ses kaydı alacağım, söylediklerin tamamen anonim olarak işlenecek. Anonimleştirilmiş hâliyle transkriptin makaleye ek dosya olarak başka araştırmacılarla da açık şekilde paylaşılabilir. Bunlara onam veriyor musun?

**P14:** Evet hocam, veriyorum.

**G:** Çok iyi, başlıyoruz.

**P14:** Yani hocam genelde ödevlerimiz için kullanıyorduk başlarda. Böyle proje yapmak için işte, sınavlarımıza çalışmak için, vizemize finalimize çalışmak için kullanıyorduk. Bize şey cevaplar veriliyordu genelde, akademik dilden direkt bir PDF dosyasından kopyalayıp yapıştırıyormuş gibi, başlarda böyleydi. Daha sonra gelişmeye başladı, bunu gözlemledim yani. Biraz daha insan gibi cevaplar verilmeye başlandı yapay zekada.

**G:** O da kendini geliştiriyor mu diyorsun?

**P14:** Geliştiriyor hocam daha güzel cevaplar almaya başladım ben ilk kullandığımdan bu yana.

**G:** Peki özel gereksinimli çocukların eğitiminde yapay zeka kullanımı konusunda ne düşünüyorsun?

**P14:** Özel gereksinimli çocuklar için kesinlikle kullanılması gerektiğini düşünüyorum. Nasılsa, şu an mesela önceden etkileşimli tahta yoktu. Bizim hayatımıza nasıl girdiyse yapay zekanın da öyle o hızla girmesi gerektiğini düşünüyorum. Tüm okullarda özel gereksinimli olmasa bile tüm okullarımızda olması gerektiğini düşünüyorum. Oraya da bizim entegre etmemiz lazım.

**G:** Peki bu entegrasyon nasıl olacak sence?

**P14:** Yani buna öğretmenlerin rehberlik etmesi lazım. Öğrencilere gösterip öğretmesi lazım. Önderlik etmesi lazım.

**G:** O zaman öğretmenlerin de eğitilmesi gerek değil mi?

**P14:** Tabi ki. Bilmeyen öğretmen geriye atar kendini.

**G:** Peki sence bu yapay zeka hangi yetersizlik türlerinde etkili olabilir?

**P14:** Yani hocam ben otizmde daha etkili olacağını düşünüyorum çünkü otizmde insan etkileşimleri az oluyor. İnsanla mesela göz bağı kuramıyor, bakamıyor yüzüne. Ama bilgisayar ekranına baktığında çocuk şey yani bakıyor direkt gözüne bakabiliyor. Bir tane film izlemiştim orada bir tane çocuk bir tane kadın vardı 20 yaşlarında. Laptopu vardı, laptopuyla çok bağlıydı hocam. İnsanlarla hiç konuşmuyordu hiç şey yapmıyordu ama sürekli laptopuyla oynuyordu. Böyle çocuklar olabilir. Böyle olduğunu da düşünürsek çocuk kendini çok güzel eğitebilir.

**G:** Peki bu yapay zekanın özel gereksinimli çocukların, bireylerin günlük yaşamlarında ne gibi etkisi olabilir sence?

**P14:** Yani günlük yaşamlarında birçok ileriki yıllarda olumsuz etkileri olabileceğini düşünüyorum. Çünkü yani çok fazla bağımlılık yapabilecek bir şey sonuç olarak. Gerçekliğin de düşeceğini sanal gerçekliğin artacağını düşünüyorum. İnsanlar koltuğa bağlı bir şekilde yaşamlarını arttıracak diye düşünüyorum. Bu da sosyal yaşamı sınırlıyor.

**G:** Peki bu yapay zekanın sence öğretmenlere ne tür katkıları olabilir, özel eğitim öğretmenlerine?

**P14:** Yani hocam yapay zekayı kullanan öğretmenler kendilerini ileri atar. Kullanamayanlar ise yani mesleğini kaybedebilir. O düzeyde bir gelişme bekliyorum yapay zekadan. Kullanamayanlar hani meslekten artık iş bulamayacak hale gelir bence. Çünkü çok iyi bir eğitmen bence yapay zeka, teknoloji.

**G:** Öğretmenler de eğitebilir diyorsun

**P14:** Mesela şu anda şey var hocam. Ablam mesela KPSS’ye hazırlanıyor. İnternetten bir tane paket satın aldı. Oradaki videoları izliyor, oradaki testleri çözüyor. Herhangi bir öğretmenden özel ders almıyor, bir şey yapmıyor. Bu da aslında bir yapay zeka.

**G:** Evet.

**P14:** Yani bunu düşündüğümüzde öğretmenlerin de mesleğini kaybedebileceğini düşünüyorum. Bunun için bizim yapay zekayla çok içli dışlı olmamız lazım.

**G:** Evet sorulardan biri buydu ilerde özel eğitim öğretmeni mesela yapay zeka özel eğitim öğretmeninin yerini alabilir mi?

**P14:** Yani işte dediğim gibi yapay zekayla içli dışlı olmayan öğretmenlerin yerine alacağını düşünüyorum. kendilerini hani teknolojiyi bilmeyen öğretmenlerin asla yeri yok bence gelecekte.

**G:** Bir de şöyle düşün yapay zekayı sadece soyut bir şey değil de mesela bunun robotlaştığını düşün. O zaman ne olur?

**P14:** yani hocam robotlaşırsa dediğim dersleri ve dediğimiz gibi hani böyle gelip bir robotun ders anlatması bile mümkün olabilir ileriki yıllarda. Bunu imkansız olarak görmemeliyiz bence. Hani sizin de dediğiniz gibi çok az da maliyeti oluyor, buna da kimse hiçbir devlet yok demez. Gelirler anlatırlar derslerini. Ama problemlere şey yapabilecekler, çözüm bulabilecekler mi? Önemli olan onlar.

**G:** Bilinçlenebilir mi sence ?

**P14:** Yani kendi kendine öğrenebilen robotlar yapılırsa bilinçlenebilirler. Ama mesela çocuklara karşı sosyal yaklaşımları nasıl olacak, nasıl iletişim kuracaklar böyle onlar? Mesela bir öğretmenin, gidip saçını okşaması ile robotun bunu yapacağını düşünmüyorum. Direkt anlatır geçer.

**G:** Evet peki duygu konusu nasıl olacak? Hani saçını okşam bişey sonuçta, bizim farkımız onlardan duygular. Çocuğu anlamamız onlara duyguyla hitap etmemiz bizim farkımız olacak bu yapay zekadan.

**G:** Evet. Peki yapay zekanın bu özel gereksinimli çocuğa sahip ailelere ne gibi katkısı olabilir sence?

**P14:** Yani aileler daha bilinçli olur. böyle yapay zeka ile ilgili kendileri de eğitim alabilirler. Nasıl bir aile olabileceğini öğrenebilir. Mesela bizim yaptığımız projede aileleri bilinçlendirmek ön planda. Mesela aile hakkını bilmiyor ama biz bunu yapay zeka sayesinde aileye öğreteceğiz. Haklarını öğrenir, kendi çocuğuna eğitim de verebilir. Bu sayede çocuğu da daha iyi eğitim alır. kendi de öğrenir çocuğu da öğrenir.

**G:** Çay içer misin?

**P14:** Olur hocam

**G:** Peki bu yapay zeka aile ve öğretmen işbirliğinde ne gibi katkıları olabilir sence?

**P14:** Yani mesela öğretmen bir ödev verebilir yapay zekada kendi yaptığı işle mi aileye gösterebilir o da tekrarlar evinde.

**G:** Bunu katkı sağlayabilir diyorsun

**P14:** Evet

**G:** Bir de şurası var yapay zeka özel eğitim öğretmenine ne gibi olumsuz etkileri olabilir?

**P14:** Özel eğitim öğretmenine dediğimiz gibi işinde sıkıntı yaratabilir. Gelecekte meslek kaygısı şu anda bile fazlayken yapay zeka yokken. Yapay zeka çıktığında hele robotlaşma olduğunda aşırı derecede artacağını düşünüyorum ben. Yapay zeka ile içli dışlı olmayan, teknolojiyi bilmeyen öğretmenler de işini kesinlikle kaybeder. Şu anda bizim atamamız iyi olsa bile ileride bu yapay zeka arttığında atanan öğretmenler bile işsiz kalabilir.

**G:** Peki özel gereksinimli çocuğa ne gibi olumsuz etkileri olabilir?

**P14:** Özel gereksinimli çocuğa olumlu etkisinin daha çok olacağını düşünüyorum. Olumsuz olarak da işte dış dünya ile bağlantısı kesiliyor. Hani bunu ders için kullanmayı çocuğa laptop için öğrettik mesela Bu laptopu çocuk oradan film de izler oyun da oynar git gide bağımlılığı artabilir. Bunun önüne geçmeliyiz, önüne geçersek güzelce eğitimini alır. Ama geçemezsek sıkıntı yaratır ileride. Çocuk sürekli ekran başında ola ola artık fiziksel sorunları ortaya çıkabilir Sürekli hareketsizlikten falan Sıkıntı Yaşar.

**G:** Peki aileye ne gibi olumsuz etkileri olur?

**P14:** Ailenin yapay zekayı, mesela bizim ülkemiz için düşünelim. Yapay zekayı bırakın teknolojiyi, telefonu kullanmayı bile bilmeyen aileler var. Bunları bilmiyorlar. Bilmedikleri için de yanlış kullanabilirler. Hiç kullanamazlar gerçi de. Yani bunu nasıl öğrenecekler, öğrenemezlerse de Sıkıntı yaşarlar öğrenemedik leri için. Teknolojiyi bilmedikleri için de kullanamazlar. Kullanamaması olumsuz bir etki bence, herkesin kullanılması gerekiyor. Yani mesela bilinçsizce kullanabilir kullanan da mesela. Bizim bildiğimiz bir şeyi bilmez. Farklı bir yapay zekadan dener o da eksik ya da yanlış öğretebilir. Farklı yapay zekaları bilmek bizim için önemli. Aileler çünkü bu konuda fazla bilgili olmayacaklar. Bizim hepsini öğrenip hepsini en iyi şekilde çocuğa öğretmemiz lazım.

**G:** Peki, bu yapay zekanın özel eğitimi gelecekte nasıl şekillendirebilir sence?

**P14:** Yani sınıftaki düşüncelere de bakarsak bence çok fazla eğitim videosu ortaya çıkacak. Böyle sanal yapay zeka ile oluşturulmuş. Sonra ben daha çok ileride şeyi düşünüyorum sanal gerçeklik VR gözlükle falan eğitimlerin olacağını düşünüyorum. Yani robotların da eğitimde ön plana çıkacağını düşünüyorum. Yani bizim yerimizi alabilirler, ama eğitimi nasıl olacak onu tam bilmiyorum.

**G:** Peki öğretmenin yerini alan bir yapay zeka sence zihin yetersizli ya da otizmde artık var olmasını engel olmaz mı, durdurmaz mı?

**P14:** Hocam sonuçta eğitim veriliyor, durduramaz. Bu bir şey değil eğitimde sonuçta biz bile durduramıyoruz istediğimiz eğitimi versek de akranlarına yaklaşmasını sağlayabiliyoruz. robot bunu belki daha hızlı bir şekilde yapar. Önüne geçemez bence.

**G:** Peki bizim ülkemize yapay zekada nerede görüyorsun?

**P14:** Yani ülkemizi şu anda teknolojide bile çok geri görüyorum. Yapay zekada da hiç olmadığımızı düşünüyorum, hiç adım atılmadığını düşünüyorum. Hiçbir yapay zeka işte konuşma programı falan çıkarılmadı bizim tarafımızdan. Bununla ilgili yapılan bir çalışma da görmedim şu ana kadar. Geride olduğumuzu düşünüyorum ama buna katılmamız lazım. Katılırsak ülkemizi çok öne atacak bir şey olur.

**G:** Özel eğitim alanında diğer alanlarda diyorsun

**P14:** Yani tüm genel olarak biz teknolojide de geriyiz. Zaten yani biz şu an mesela internete bile bağlanmakta sıkıntı yaşıyoruz. Teknolojide nasıl ileride olduğumuzu düşünebiliriz ki. En basit sıkıntılarımız var problemlerimiz var. Temelde bile problem yaşarken ileri düzey bir teknolojide…

**G:** Peki ülkemizde yayılması nasıl sağlanabilir sence yapay zekanın?

**P14:** Bunun için de öncelikle işte özel bir yatırım olmalı mesela. Bir kişinin yapay zeka alanında adım atması lazım olabilir. Ya da devletin adım atıp öncelik etmesi lazım olabilir. Bununla ilgili kimseden bir şey görmedim ben. Ne bir insan işte yapay zeka ile ilgili bir çalışmasını gördüm ne de devletin. Hani biz yapay zeka programları vereceğiz, robot üreteceğiz dediğini görmedim.

**G:** Peki senin yapay zeka ile ilgili hiç böyle yaşanmış anın, söyleyebilecek bir fikrin var mı?

**P14:** Yapay zeka ile ilgili hocam mesela bir tane program vardı Karakter AI diye. Orada bir sürü karakter vardı hocam, onlarla sohbet ediyorduk. Böyle bir anım vardı. Ben Adolf Hitler ile konuşmak istedim. Adolf Hitlerin karakteri olduğunu gördüm orada. Onunla konuşmak istedim. Yapay zekada onu bayağı bir değiştirmişler hocam. Ben onun fikirlerini bildiğim için hocam ona söylemek için biraz zorladım onu, ama söylemedi. Mesela işte şu an ki durumdan bahsettim. Bizim ülkemizde çok fazla Suriyeli var, sen işte şu an da Türkiye’de olsan ne yapardın falan gibi böyle sorular sordum. İşte Suriyeliler ile ilgili politikalar üretirdim, onları entegre ederdim falan filan dedi. Ben de dedim sen böyle birisi değilsin, Yahudilere nasıl davrandığını biz biliyoruz falan dedim. Onu söyletmeye çalıştım ama söylemedi. O zaman yanlış yapmış olmam şu an öyle söyleyeceğim anlamına gelmez, falan dedi. Böyle bayağı bir şey yapmıştım oradaki karakterle. O zamanki düşüncesini söyleyemedi yani şu anda. Bir de biliyorsunuz sansür falan da var. Herhalde onun için söyleyemedi.

**G:** Bu Türkçe konuşuyordu.

**P14:** Aynen Türkçe konuşuyordu.

**G:** Sen karşındaki kişinin gerçekte o olmadığını düşündün ama, çünkü onun fikirlerini biliyordun.

**P14:** Yani yansıtmadığını düşündüm, aynen.

**G:** Benim sana yapay zeka konusunda soracaklarım bunlardı. Senin eklemek istediklerin var mı?

**P14:** Yok hocam sağ olun.
