# Supplementary material for: These children can become independent thanks to artificial intelligence! – A qualitative analysis of pre‑service teachers’ views on the use of artificial intelligence in special education
Source: PLoS One. 2026 Aug 28;21(8):e0355713. doi: 10.1371/journal.pone.0355713 (PMC13524254; doi:10.1371/journal.pone.0355713)
Supplement: Supplementary file 5 [file pone.0355713.s005.docx]

**Supplementary File S4**

**De-Identified Interview Transcripts (Verbatim English Translation)**

**Manuscript:** PONE-D-26-11239 (revision)

**Study:** Pre-service special education teachers’ perceptions of the use of artificial intelligence in special education

**Ethics approval:** Scientific Research and Publication Ethics Committee, Nevşehir Hacı Bektaş Veli University. Decision No. 2024.05.100; Date: 30 April 2024

**Number of transcripts:** 14 (P01 through P14)

**Total speech turns:** 689 (341 participant turns + 348 interviewer turns)

**Language:** Verbatim English translation. The Turkish originals were prepared as a separate file (S4 Turkish), and the verbatim English translation here is provided for reviewers and readers who do not read Turkish. All 689 speech turns of the Turkish source are preserved one-to-one; no turn has been omitted, condensed, or summarised.

***Note on anonymisation, consent capture, and translation.*** *The transcripts reproduced below have been anonymised for open public deposition. All participants provided explicit verbal consent, captured on the original audio recording, for the open-access sharing of their de-identified transcripts as supplementary material. The opening consent exchange of each interview, originally captured at the start of the audio file and transcribed for the present deposition, appears at the start of each transcript. The anonymisation procedure was carried out following the practice-oriented guidance of Saunders, Kitzinger and Kitzinger (2015) and Kaiser (2009). Each participant has been assigned a participant code (P01 through P14), corresponding to the codes used in the published article. The interviewer is labelled G (Görüşmeci). The Turkish honorific ‘hocam’, an address term commonly used by students towards their instructor and one without an exact English equivalent, is rendered as ‘Professor’ throughout, in order to preserve the relational register of the original. Country and continent names (e.g., Türkiye, Europe, Japan, America, China, Germany, Korea, Netherlands), names of artificial intelligence tools and platforms (e.g., ChatGPT, Copilot, Otsimo, WordWall, Canva, Evernote, Duolingo, Character AI, Runway, Snapchat AI), and reference to the historical figure named in Participant 14’s anecdote about a chatbot have been retained because they constitute the substantive content of the study rather than personal identifiers. References to specific Turkish institutional features that could indirectly identify the national setting have been generalised (e.g., ‘KPSS’ rendered as ‘a national civil service exam’; ‘ablam’ [my older sister] rendered as ‘a family member of mine’; ‘Kanun Hükmünde Kararname’ rendered as ‘decrees with the force of law’). The translation is verbatim in the sense that every speech turn of the Turkish source is preserved one-to-one, every ‘yes’/‘no’ response is retained, every repetition of the interviewer’s question by the participant is preserved, every filler (‘yani’ as ‘I mean’, ‘şöyle’ as ‘well’, ‘aslında’ as ‘actually’) is rendered, and every hesitation marker is retained. Sentence boundaries follow the participant’s own intonational phrasing as transcribed in the Turkish source.*

**P01: Interview Transcript**

**G:** Before we begin our interview, let me briefly explain. This is a study on the views of pre-service special education teachers about artificial intelligence. I will ask you a few questions and will be making an audio recording. Everything you say will be processed completely anonymously, your name information will not appear anywhere. Additionally, in its anonymised form, your transcript may be shared as a supplementary file of the article, openly accessible to other researchers. Do you consent to all of these?

**P01:** Yes Professor, I consent.

**G:** Thank you, in that case I am beginning with my first question.

**P01:** I definitely think that artificial intelligence should be used. I think this usage could be like this: we would identify the needs of our individuals with special needs, on which topics we could get help from artificial intelligence, that we should find these out, and then there is the artificial intelligence engineer, computer engineer, whichever software engineer dealing with this artificial intelligence (I do not know exactly which part), to convey this situation to them, and in communication with them, in the coming process, I think we should solve these problems together with artificial intelligence.

**G:** Have you ever used any artificial intelligence tool before? If you used it, what kinds of outcomes did you obtain?

**P01:** Right now I have used ChatGPT. The outcomes were as follows: it allowed me to quickly gather data, to reach essential information, to obtain data without getting stuck in unnecessary details, without drowning in them. When I use it, these things happen.

**G:** Well, in your opinion, what kinds of contributions could the use of artificial intelligence make to the special education teacher?

**P01:** I think the use of artificial intelligence will save the teacher from wasting unnecessary time, from spending unnecessary effort, from getting tired, from all these things. I think it will have these benefits for the teacher. To give a slightly more concrete example, for instance, suppose we use artificial intelligence to increase visuality in the student's understanding of a topic. Instead of the teacher constantly making an effort to find something visual, artificial intelligence can bring it ready to her. Or while the teacher cannot find many varied things, artificial intelligence gives her various things. The teacher obtains various resources. In this way, both in terms of getting tired and in terms of time, I think it will be beneficial in many ways.

**G:** Well, in which disability categories do you think this artificial intelligence would be more effective?

**P01:** I think artificial intelligence would be more effective in intellectual disability, mild, moderate, severe, all of them... apart from that, in autism, learning difficulty, in such situations.

**G:** Why do you think it would be more effective in those?

**P01:** Because in hearing and vision, for example, if we can solve the hearing problem even a little, the child no longer needs our special education service. For example, with artificial intelligence the child could not perceive sounds very well. There is hearing impairment, the child cannot perceive sounds. With artificial intelligence we solved this problem. The child can now perceive sounds, can notice the teacher's voice, can understand. Since the child now understands, our need to provide special education is reduced. That is why I think there is more need in intellectual disability, autism, in such areas.

**G:** Well, in your opinion, how can these artificial intelligence systems or tools, for example, be integrated into special education services, into the special education system?

**P01:** First of all, teachers need to become aware of this. I just recently learned about ChatGPT or other artificial intelligences. It has been only a few months since I noticed these. I think there are many people like me. People who have not noticed, who live far from these. First perhaps through the Ministry of National Education, or through different situations, in the news.

**G:** Well, in our country there is currently a special education curriculum, a special education system. Do you think artificial intelligence systems can be easily integrated into this?

**P01:** Yes, I do not think it will integrate very easily. Because every teacher is different, and frankly I think it is a little more difficult to get some teachers to accept some things, to be open to innovations. We need to be in communication with these teachers and to be able to get them to accept these things. Also, teachers in this way, people are a little more reserved towards what they do not know. Since artificial intelligence has only recently entered our lives, I think teachers may behave a little more reserved, and that it can be easier to go with what one already knows. For this reason, I think it will be a little harder for teachers to integrate artificial intelligence into our lives.

**G:** In your opinion, how could it be made easier?

**P01:** I think how can it be made easier, I said teachers hesitate because they do not know this work, by correcting that not-knowing part. That is, by teaching teachers about this situation. By showing them, considering that there is really nothing to hesitate about, that it is not a workload entering our lives but on the contrary will make our lives easier, will ease our workload, by teaching them these things, it could integrate more easily.

**G:** Well, what do you think about the possibility of artificial intelligence in the future taking the place of the special education teacher?

**P01:** I do not think artificial intelligence is something that can be very predicted. Because it is a technology that can develop very fast. I had actually read some things about artificial intelligence in the past, a little. When I saw, there were times in the past too when artificial intelligence was on the agenda and then fell off the agenda. But right now it is back on the agenda and in a developmental stage. I cannot predict how artificial intelligence might develop or how fast it will develop. Maybe it will show a very rapid logarithmic increase and will take a great place in our lives, but maybe artificial intelligence could again fall off the agenda. So my current opinion, in short, is that I do not think artificial intelligence can take the place of a teacher in the short term, that is, within twenty years.

**G:** Very nice. Well, in your opinion, what kinds of contributions could this artificial intelligence make to parents, to families with a child with special needs?

**P01:** In artificial intelligence getting information, then in how the parent should proceed, or in cases where the parent also needs support from us in education at times. In these training sessions, by getting support from artificial intelligence, the parent can be helped.

**G:** Well, do you think it could be useful in the cooperation of the special education teacher and the family? For example, what kinds of benefits could there be? Could it enable the teacher and the family to work together?

**P01:** Artificial intelligence can do this for the family and the teacher by providing various documents, by showing different ways, or while they are in communication... but in communication too, I do not think artificial intelligence can do much at the moment. Because when the parent and teacher themselves communicate, I do not think artificial intelligence will intervene much at the moment. They will solve this work themselves, I do not think they will need artificial intelligence very much in this communication.

**G:** Well, what could artificial intelligence do in the daily life of these children, these individuals with special needs?

**P01:** It could be a guide. In daily life, when they encounter some problems, artificial intelligence could help in this direction. Artificial intelligence could even be a friend. In meeting their needs, in expressing their needs... For example, when a student cannot quite express their needs verbally, through artificial intelligence, artificial intelligence could turn them into something visual and could express their needs, for example. In this way, I think it would help with their daily needs. For example, when food comes, the daily need of food has come in front, artificial intelligence could show pictures of food that the child does not like or that they like, with the help of artificial intelligence, to their family, and could help with a daily need.

**G:** Well, I will also ask you this: what kinds of negative effects could artificial intelligence have on the special education teacher, student, family?

**P01:** There could be a negative effect like this: since artificial intelligence really presents ready information in front of us all at once nowadays, the teacher might think that they can be content with only the data they obtain from it, without engaging in much research, without struggling much. This is a problem because, although artificial intelligence has benefits for the teacher, as we said at the beginning, in terms of time, effort and so on, I think one should also not be content with only artificial intelligence sometimes. In this situation, the teacher, being satisfied with this, might not turn to different research, to different things. They do not learn different information. This is in terms of the teacher. If we think in terms of the student, I think they could focus only on artificial intelligence and become dependent on it. Someone who can solve their life only through artificial intelligence...

**G:** That is, they would not be an independent individual...

**P01:** They would not be an independent individual, only someone who can solve through it, who faces problems when it is not there, who only turns to artificial intelligence when they have a problem, such an individual might emerge. In terms of the student, like this. For the family, it could also be a bit like this. But for the student, this is more possible. For the family, what could the negative aspects of artificial intelligence be? They could distance themselves from their child. That is, when they have a child who constantly turns to artificial intelligence, the family may have difficulty in communicating with the child, may distance themselves from their child. Besides that, for the family, they may give too much importance to artificial intelligence. Artificial intelligence is, after all, in my view, a person who thinks it goes up to a certain point. Sometimes they might think artificial intelligence can solve everything. Suppose the family thinks their need is reduced, that artificial intelligence helps the student in their daily needs. In this situation, the family does not feel needed. Because they do not feel it, the family now stays a bit more in the background in meeting their child's needs. My thought is that such a problem could arise for the family and for the child.

**G:** Now P01, well, if we include artificial intelligence in the education of these children with special needs, what do you think the use of artificial intelligence could bring in the future? How will the future be shaped then in your opinion?

**P01:** The future is a great mystery, as I said. Since I do not know how artificial intelligence can be directed or how people can use it, let me say this first: it is a mystery. In the past too, inventions are made, for example people used it as an atomic bomb. Just like that, with artificial intelligence too, we are always thinking of its positive sides now, that it would benefit our lives this way, that way... but there is also the possibility that the negative sides could be used and it could go to the negative side of things. But generally, if we think within twenty-thirty years, because I cannot really predict fifty-sixty years, believe me, Professor. If you ask me what artificial intelligence will be like fifty-sixty years later, I have no idea, I have no thought about how much it will progress. But in my estimation, my thought is that within twenty-thirty years, artificial intelligence will ease our lives in this way, that is, it will be more in our lives, will be a helpful technology to us, will have benefits in many ways. Time, effort, money... all of these included.

**G:** Now what I wanted to ask you was these. Do you have anything to add about artificial intelligence and special education?

**P01:** Generally, let me say this: I think for now, artificial intelligence should be used, awareness should be raised, people should notice this and it should enter our lives.

**G:** For these children too...

**P01:** For these children too, of course...

**G:** P01 thank you, well said.

**P01:** Professor, I thank you.

**P02: Interview Transcript**

**G:** Before we start, let me briefly tell you. I will be audio recording our interview. Your name will not appear anywhere, everything will be processed anonymously. Your anonymised transcript may be placed as a supplementary file of the article and shared with other researchers as well. Do you consent to these?

**P02:** I consent Professor, there is no problem from my side.

**G:** Very good, let us begin.

**P02:** On the matter of education, we actually mentioned at the beginning. I mean, artificial intelligence can be greatly developed in the world right now. I think it can be seriously used in the instruction of these children, and I think it will be useful. For example, the project we wrote was about mathematics, and it was a project where we used artificial intelligence. Because we thought it could also be effective in the children's instruction, we actually used artificial intelligence. Many applications can be designed with artificial intelligence regarding the instruction of children.

**G:** Well, have you ever used any artificial intelligence tool yourself? If you used it, what kinds of outcomes did you obtain?

**P02:** For example Professor, I used Snapchat's AI. Now I use Copilot more. We used to use ChatGPT more last year. Copilot has this nice feature, when you ask a question, it gives you the answer. It also provides information about which sites, where it took the answer from. I have started using Copilot more frequently now. I mean, I have been using it for a while now, generally more often. Because Snapchat and so on give more superficial answers, Snapchat's AI does. ChatGPT is a bit more scientific. Copilot gives both scientific answers and high applicability, and because it is also clear from which site it took the answer, it makes my work easier.

**G:** Well, what kinds of contributions do you think this artificial intelligence use could make to the special education teacher?

**P02:** What kinds of contributions could it make to the teacher? Professor, we now provide instruction at school, but we always say we have difficulty in fluency, in generalisation. Because we cannot know whether they adapt it to normal life or not. We cannot follow up outside, we cannot observe, and since families are also inadequate on many subjects, the family does not know either. But with the applications artificial intelligence has developed, we can continue education at home for this child, and we will both have increased the child's fluency and made generalisation easier, I think.

**G:** Well, in your opinion, in which disability categories can the use of these artificial intelligence systems be more effective?

**P02:** Autism, intellectual disability, Professor, well, now it can also be developed in vision, but I think more frequently it can especially be used in mild intellectual disability. I think it can be used in autism, in disabilities like dyscalculia.

**G:** Well, how can it be used?

**P02:** Professor, if we are to start from the first stage, do we have difficulty in diagnosis? Yes, we do. Our country is developing a little better in this area. But for example in the Netherlands, they do not want to make a diagnosis before age 8. Artificial intelligence, I think it was the Koreans who found it, has begun to make diagnoses, as I know. I mean, if we handle the diagnosis at the first stage, I think we will already have lowered the age of starting education. This also makes our work easier for increasing the child's independence in the future. Then in their instruction, we can use many applications, and as I said earlier, both the teacher's work will become easier, and we will have increased the family's, the child's generalisation through education at home. We will have used artificial intelligence in many areas this way.

**G:** Well, in your opinion, how can it be integrated into the current existing special education systems?

**P02:** I mean, with the development of technology Professor, in schools, for example with smart boards, teachers conduct lessons, we can integrate it there and continue with applications. Also, for example, in concept teaching we have difficulty in children with autism. At this point, artificial intelligence is actually making concept teaching easier with new applications. We can use it here, in teaching mathematics to children. We can use it in academic skills. I also think we can use it in social skills, but I do not know exactly how, Professor.

**G:** Well? Do you think such integration would be easy and functional?

**P02:** Definitely. I think it will greatly ease the teacher's work. We might experience this difficulty in our country: we follow technology a notch behind. Compared to Japan, compared to Korea, in terms of the country we might experience some difficulty, but in terms of easing our work, it would really benefit us, I mean.

**G:** Well, do you think this artificial intelligence could take the place of a special education teacher in the future?

**P02:** In academic skills, yes I think it could take its place. But on the matter of social skills, I do not think it could fully take the place of a teacher.

**G:** I see. Well, there is also the family of course in the matter. What kinds of contributions can artificial intelligence make to families, to parents of children with special needs?

**P02:** For example, we have a family education course, but to be able to provide education to the family, we need to determine joint hours with the family. We need to determine a program so that we can invite the family accordingly. But if we can provide education through artificial intelligence, if we can develop such applications, the family can easily reach this whenever they want, at any moment. This in turn will have facilitated the education the family will give to the child. It will have made the family's instruction easier and it will be at their fingertips at any moment in this way.

**G:** Well, again, what contributions could artificial intelligence make to family-teacher cooperation in special education?

**P02:** Family-teacher cooperation? Well, how could it be? I do not have an idea on this matter Professor, I mean.

**G:** Well, what kinds of contributions could it make to the daily lives of individuals with special needs, in your opinion? Artificial intelligence?

**P02:** Professor, how could it contribute to daily life? For example, we also mentioned in the course, if we could do this skill instruction with artificial intelligence. Why do we teach skills to the child? So that the child's independence increases. So they do not stand out outside. For example when they will do a simple shopping, so they can do it by themselves. If artificial intelligence does these instructions, we will have decreased this child's likelihood of being excluded outside, we will have decreased the likelihood of being excluded among peers. This is also what we want. We want this child to gain independence. I mean, in terms of liberating the child, in reducing dependency on the family, reducing dependency on the teacher, increasing acceptance among peers, I think it can be effective.

**G:** Well, if we think in terms of artificial intelligence, the special education teacher, the student with special needs, the parent, what kinds of negative aspects could there be?

**P02:** It takes the work away from teachers' hands, Professor. I mean, if we think about the future part, if we think in terms of the teacher. As we mentioned, if it can take the place of a teacher, it will sort of eliminate special education teaching. I mean, what kind of negative could there be for the family? Let me also touch on the family side and the child side. For example, we do not want to show too much screen to children with autism. We want to reduce screen exposure. We think it is harmful. But in artificial intelligence applications too, the child has to be exposed to the screen. I mean, the child has to do something with the screen. Again, of course, in order to eliminate this harmful factor, we do not want them to be exposed to the screen. We do not want the child to do something one-sided, that is, we do not want them to listen to it and stand there like a statue. We want them to be in interaction. We want the child's language development to increase. Can we provide this with artificial intelligence? Yes. We can again eliminate this harmful part of the screen. What kind of harm could there be for the family? I do not think there is any harm for the family. I am focusing more on the beneficial part, frankly.

**G:** Well, how can artificial intelligence be used in the future in the education of children with special needs? That is, how can it shape education in the coming years in your opinion?

**P02:** I think it will have a positive effect. Even now, for example, there are many applications. There are applications that will increase their academic skills. These have already begun to be used in our country slowly. Could you ask the question again?

**G:** How can artificial intelligence shape the future of special education in your opinion?

**P02:** How can it shape it? I mean, I think it will be in a positive direction. But, can it add new, different things that we currently do not know? Yes, it can, because. There are many areas where we already fall short in special education. There are many things we cannot succeed in. For example, we cannot do much with the children's stereotypical behaviours, we cannot suppress them. We have difficulty in reducing them. Maybe artificial intelligence can produce new things on this matter. I mean, artificial intelligences could be produced. In academic skills, we already think it will achieve positive success. That's how.

**G:** What I wanted to ask you was these. Special education teacher, artificial intelligence, child with special needs... Do you have anything you would like to add?

**P02:** I mean, again, as we mentioned in the course, ethical applicability is a bit of a problem for me, in my opinion. Also, after all, with artificial intelligence, for example, suppose we are producing robots, we had discussed this. With these robots, what will we meet the energy with? I am thinking about this issue. I mean, how will we produce so much energy, that's what I am thinking. Apart from that, I think it can be beneficial in many respects.

**G:** OK then, thank you P02, you explained well.

**P03: Interview Transcript**

**G:** Before starting the interview, let me briefly say. Be calm, this is not an exam. You will answer the questions I will ask verbally, and I will be recording. The answers you give will be anonymous, your name will not appear. The anonymous text may be openly shared as a supplementary file of the article with other researchers as well. Do you consent to this?

**P03:** I consent Professor.

**G:** OK, we are starting with the first question.

**G:** ...what do you think?

**P03:** Well, Professor, I think it will ease the work of both the student, the teacher, and the family. It saves time, allows reaching more accurate data. Apart from that... I cannot think of anything else in that way right now, but...

**G:** I will ask, do not worry, you relax. Well, have you ever used any artificial intelligence tool before? If you used it, how was it? What kinds of outcomes did you obtain?

**P03:** Well, I never used it before Professor, I started using artificial intelligence with your assignment in your course. Well, the answers? I had asked a question like this and it had said it does not have much idea about current answers. It says more well-known things. For example, it did not have much to do with numerical data. I had asked something numerical.

**G:** Now, you are thinking of becoming a special education teacher in the future. Well, what kinds of contributions could artificial intelligence offer to special education teachers in your opinion?

**P03:** Professor, well... in data collection it is more such. It gives more accurate answers. Recordability is high. Apart from that... Professor, please excuse me, I got stressed.

**G:** Think as if you are chatting with your friend, relax.

**P03:** Professor. It does not happen that way. Could you ask again Professor?

**G:** Now what kinds of contributions could artificial intelligence make to special education teachers?

**P03:** Professor, it makes the special education teacher's work easier, can increase communication with the student. Well, a teacher may not want to communicate with a student. But thanks to artificial intelligence, communication with the student could become stronger, I think.

**G:** Well, you are also a physiotherapist now. In which disability categories could the use of this artificial intelligence be more effective?

**P03:** I think this way Professor, there is almost no disability where it would not have an effect. Thinking physically, with the signals these motion sensors give to artificial intelligence, problems in the student's movements would be reported more concretely. Apart from that, in intellectual students, it could develop their social skills too. It could follow up their daily required rituals, analyse them, and provide feedback. The feedback would be more reliable. Well, if I come to specific learning difficulty, how can I say... It can analyse in which area the student is lacking, or in which areas of instruction it is more productive, and provide feedback to the student accordingly. Like this.

**G:** Very nice. Well, how can this artificial intelligence be integrated into special education services, that is, into this existing special education system?

**P03:** Professor, I think there is benefit in producing various applications for this. For this, we also need to include the family. Well, it is not something that can be only between the student and the teacher. They need to continue this at home too. Of course, if this is outside of state control, frankly most families do not pay much attention to the student Professor, the state needs to watch over this. Teachers need to give feedback on this to higher levels. I mean, can a penalty be applied? It cannot be applied for not doing it, but. It could be applied through something like a gift or things that motivate the family, I think.

**G:** Well, do you think the use of artificial intelligence within special education would be functional? Would it be easy? Think about the current situation. That is, think about the teacher conducting lessons in special education classrooms, would it be functional for them? What kinds of facilities would it provide?

**P03:** What kinds of facilities would it provide, Professor? I could say it would broaden the teacher's horizon. Well, OK, a teacher who has been working for a long time, well, it is activities and so on, may not experience much problem, but a newly graduated person will certainly experience problems, it broadens their horizons. Such more materials, more activities, more things that are beneficial for the student are produced. I think it will be functional.

**G:** Well, what do you think about the possibility of artificial intelligence taking the place of the special education teacher in the future, in the coming years?

**P03:** Professor, I do not think artificial intelligence can take the place of the special education teacher. Because we form an emotional bond. We react according to the student's reaction at that moment. Well, artificial intelligence cannot fulfil these. OK, it makes the teacher's work easier, but does it take their place? I think it cannot.

**G:** Well, what kinds of contributions could this artificial intelligence make to families with a child with special needs, to parents?

**P03:** What kinds of contributions to the family?

**G:** This is not an exam P03. Relax, your own thoughts may even be very far-fetched, some people might call them very dreamy, but say them freely...

**P03:** Professor, well, the contribution to the family would be like this. Taking care of a child with special needs is really very difficult. Families' education levels may not keep up with this. I was in the district Professor, the well, in the families it was at the student level frankly. Marriages between relatives etc. were a lot. Well, how much can a person who is inadequate themselves be adequate for the child? It is to be thought. Well, with these applications but how? Surely well... It would be more beneficial for the student. I mean, I could not quite express it but. Professor, the family is inadequate.

**G:** Can it increase your family's adequacy?

**P03:** Yes yes. It makes it easier to follow the student. For example, if the previous level and the next level can be recorded, the family will be more encouraged to use that application. I mean, maybe they will use it themselves too.

**G:** Well, what kinds of contributions could this artificial intelligence make to the cooperation of the special education teacher and the family in your opinion? Teacher-family...

**P03:** Teacher-family cooperation...

**G:** Could it make it easier for example? How?

**P03:** It would make it easier of course Professor. As I said, the family cannot follow the child's situation, but thanks to these applications they can follow. Or the family knows what to do. Thanks to the applications, the family can learn from there what needs to be done and reflect it onto the child. I mean. It is becoming short short but...

**G:** It is OK, no problem. Well, what kinds of contributions could artificial intelligence make to the daily lives of these children with special needs?

**P03:** These children have daily rituals. They know some of them, they do not know some, they skip them. It could follow these up, can show the daily things that need to be done as a schedule and have the children do them. It can support this visually, audibly. By following them up, the student can learn from there what they need to do, at what time they need to do it, how correctly, how, that is, how very nicely they need to do it. Because for example brushing teeth may seem like a simple thing, but the child needs to know to do it in the morning, at noon, and in the evening. Most families also do not know this and cannot teach in this way. But thanks to applications they can do it.

**G:** Very nice. Well, what kinds of negative aspects could this artificial intelligence have on the special education teacher, family, and student in your opinion?

**P03:** Well Professor. I am not sure about data privacy. When this artificial intelligence is in the child's life, willingly or unwillingly it will enter the home environment, will enter the school environment. Whether this data is being kept in confidentiality, I do not know. Apart from that, I think it also enables the child's socialisation. The child may be afraid to communicate with another person, to communicate, but afterwards by chatting with this artificial intelligence they could open up. Is there a negative side for the family, I am thinking. Well, there could be a cost, but as I said, the state should also support this.

**G:** Well. How do you think artificial intelligence could shape special education in the future, in your opinion? How could it shape it?

**P03:** Well Professor. I think in the future it will be very useful and functional. I mean, people who say they will not use it will also be forced to use it. Because it saves time. It offers more reliable data. Once it is like this, people will be forced to use it. It will not be like it is now, it will be an essential part. Well, if someone had said before. Well, it will be like a device like a phone. It will be in everyone's life. People might find it very funny, but right now no one is without a phone in hand. Artificial intelligence will be like this too and I think it will largely settle into the lives of students with special needs.

**G:** Now, what I wanted to ask you about artificial intelligence, special education, and children with special needs were these. Do you have anything to add? Things that come to your mind like 'maybe it could be like this or like that'...

**P03:** Right now nothing seems to come to mind.

**G:** Do you have an experience you would like to share on this topic? Do you have an idea you would like to share?

**P03:** Professor, when you gave the assignment I designed a project. It seemed very nice but then it seemed costly. It was like a small home robot at the home of a child with special needs Professor. Well, afterwards it was projecting onto the wall what they needed to do and so on. It was very logical but my thought is like that, but because it was very costly I had not said it. These are what I can say.

**G:** Thank you P03.

**P03:** I thank you Professor.

**P04: Interview Transcript**

**G:** Before starting, let me give a brief piece of information. Throughout our interview I will be making an audio recording. The things you say will be handled anonymously, no personal information will appear in the publication. In its anonymised form, your transcript may be openly shared together with the article as a supplementary file. Do you consent to all these?

**P04:** Yes, I consent Professor.

**G:** Thank you, we are beginning.

**P04:** Professor, for teachers for example, in this process it could provide time saving, could provide workforce saving. If we come to its advantages and disadvantages, in the future it will surely reach a state where one can benefit from it even more. The disadvantages part is also there for sure. For now, we have not yet moved on to its advantages. I mean, because it is not used in the full sense.

**G:** Well, have you used any artificial intelligence tool before? If you used it, what did you do?

**P04:** I used it. You asked questions, I mean to Snapchat's AI. For myself, both related to special education and on other topics. For example, I even got ideas on the subject of being defrauded, on where I should apply or how I should follow a path. On the subject of withdrawing money from my account, it even helped on that subject.

**G:** Well, what kinds of contributions could the use of artificial intelligence make to special education teachers in your opinion?

**P04:** As I just said, what could be its contribution to education teachers? As I said, it definitely saves time. The teacher's workforce decreases, but it can also overtake the teacher at some point, I mean. I mean, perhaps tomorrow or the day after, when in education an artificial intelligence robotic and a special education teacher work side by side, it could also enter their field. But it is certain it will reduce workforce, but it can also enter their field. It can also have disadvantages. It can violate confidentiality. Because if it is recording students' videos, photos, and there is a possibility it is recording. We cannot know, we cannot trust how it is managed. Things like this could happen. Well, on the topic of communication, visual impairment, or, well...

**G:** In which disability categories would it be more effective in your opinion?

**P04:** I think it will be used more in communication and learning difficulty. Because communication inadequacy and learning situation appear more in daily life, so I think it could be better in those subjects. What could it be like? Well, there are some data-based systems for individuals with visual impairment, for example it could provide that for them Professor, like a voice assistant. I mean, I think it would be more useful for sensory disabilities. Well, less for intellectual disability and autism, more for hearing and vision, or for learning disability.

**G:** Well, special education services are currently being provided. How can artificial intelligence be integrated into these special education services?

**P04:** Well, first it needs to be regulated and systematic. How can it be integrated? I have not thought too much about it, but well, if when integrated it does not comply with a certain rule, a certain system, instead of providing an advantage it could become a disadvantage, I think. How it will be integrated, time will show. What things can be done? I mean, if it comes to this stage, if we reach the robotic stage, then it will already be easily integrated. But before reaching that stage, for example things that are used are still on paper. There are still things that have not even moved to computer environment. Well, there are data collection systems for example. Since there are still traditional teachers who cannot even overcome this, first of all on this matter, for example teachers or everyone in that system needs to be educated. I mean, if we still cannot transfer records even to computer environment, if we are still going to proceed on paper, then there is already a problem. So we need to abandon traditionalism. But for this there is also a stubborn aspect, there are communities. So first we need to accept this, I think.

**G:** Then, that is, its functional use will not be easy.

**P04:** Yes, persuading people about this situation...

**G:** Well, how can it be made easier?

**P04:** By doing things, well, giving education, explaining. I mean, directly to a teacher, especially a traditional one, especially an old teacher, it will be like this, it will be like that... I mean, there are teachers who do not know how to turn on a computer. Maybe right now there are still not. I mean, more young ones perhaps, but it is a bit, well, was, but not knowing how to turn it on was a very extreme example. I mean, there were those who thought I do my work on paper, I have nothing to do with the computer, I write the data on paper, but on this matter, the full-meaning education advantage. On the subject of disadvantages, regarding artificial intelligence, we need to awaken this in the teacher: artificial intelligence will make your work easier, will not make it harder. But teachers who are afraid, of course, are there. I mean, in my opinion.

**G:** Very nice. Well, do you think in the future artificial intelligence can take the place of the special education teacher?

**P04:** This could be much in the future. I mean, in a near date it cannot be a five or ten year process. But in the future, not only of the special education teacher, it can take the place of many things. I mean, waiters could become robots. I mean, how can it take the place of special education teachers? It can. I mean, really in terms of teaching, I think the most difficult for artificial intelligence, for robots, the most difficult profession group for a teacher to be taken over is, I think, teaching. I mean, a robot waiter would be easier, would be easier. I mean, picking up a plate and taking it can be processed more easily. But specifically taking the place of a branch teacher, it needs to be fairly equipped to be acceptable.

**G:** Is special education teaching the last job to be done?

**P04:** I mean, in terms of teaching, special education teaching is the last, but in terms of profession groups, teaching is the last, I cannot know that. Because engineering is the subject. I don't know, genetics, well, genetics could also be easier, I think. Teaching is more different, since it is a bit something that has emotions too, just loading some things may not be enough. Of course if robots are going to have emotions, that is a different subject.

**G:** Well, what kinds of contributions could artificial intelligence make to families who have children with special needs in your opinion?

**P04:** Its contribution to families would be like this: if the family proceeds systematically, in periods when the family cannot find an interlocutor, for example, as a teacher, sometimes for material reasons, well, benefiting from the robot of course requires materials and that is the future dimension, but even now most parents of students with special needs, of individuals, the number of families who have no knowledge of artificial intelligence is many, I mean as a ratio. If they knew for example, they could ask artificial intelligence a question they want to ask normally on a daily basis but cannot ask by calling the rehabilitation centre. I mean, for now, this is very basic at the first stage. There is such a process right at their fingertips but they do not know. But at the advanced level too, in robotics, at that time too, financial power always puts one ahead. A family with good financial means assigns a robot like a shadow teacher to themselves, a robot that is always beside them, I mean buys one for their child. Both improves their own quality of life, saves time for themselves, and is wonderful for the student.

**G:** Very nice. Well, what kinds of contributions could artificial intelligence make to the daily lives of these children with special needs, of adults, in your opinion?

**P04:** In their daily lives also, well, regarding this nothing directly came to my mind. I also preferred to ask, I mean I benefited from artificial intelligence. The disability group that most appeared in front of me in daily life was the visually impaired. The most basic example said it could help them. As I said, well, those who have not gained motor skills, with voice assistants or as I said again, those with sensory disabilities, I thought it would benefit more in daily life. But of course in intellectual disability and autism it will also benefit in daily life. As I said, if the shadow teacher is later, it can also benefit daily living skills. If it is constantly beside them. Or as I said, the family, themselves.

**G:** Well, what kinds of negative effects could artificial intelligence have on the parent, then the student, the teacher in your opinion?

**P04:** The negative aspects, the disadvantages could be like this. If this will be a recording technique, I mean while the teacher, by their own character, with their emotions or with respect for their work, how shall I say, acts correctly, artificial intelligence, that is, these recording techniques in robotic events in the future, in situations could violate confidentiality. Because it takes the audio recording, it takes, I mean it will record on video. And tomorrow or the day after, for example, it could use it elsewhere in a different way. According to the family's wishes, there is a high probability of it doing things.

**G:** What kinds of negative aspects could there be for the teacher in your opinion?

**P04:** The teacher too, while the teacher says 'I will benefit from this', that is, while it will provide such an advantage, the teacher could also, well, violate. I mean, the teacher's disadvantage will also be this: after a certain point there will be no need for the teacher. They will not be able to do their job directly, I mean. Maybe when robots increase more and one is no longer in financial difficulty in this matter, they will be able to see them more as, well, will it be a robot, that is a technological device be better, or a person, when they ask, maybe they will prefer robots. Maybe the teacher will not be preferred. But in periods when they are preferred and used together, how could it be a disadvantage? As I said, it can enter their field. I mean, and they may not be able to manage it. They will also manage the robot, perhaps will be forced to manage it. They will have to cooperate with it, I mean alongside themselves. They will have to manage someone who is not flesh and blood. They will also have to control it. Maybe it will control them. Without realising. I mean, like that. And also, they will try to give education to the student. They could have difficulty.

**G:** Well, how do you think artificial intelligence could shape special education in the future? How can it shape it?

**P04:** I mean, I think it will shape it in a positive direction. I think, well (?...) maybe it cannot be, well, I do not know Professor. I mean, again as we said, as I said, although not as much as other profession groups, it will reach a state where it can take on teaching. In that situation, it shapes it. And it makes the work easier. It makes data clearer. Some things will not remain in the air. Cooperation with families, parents, the child can increase more. I mean, I think exactly like that.

**G:** Well, do you have an experience or an idea about artificial intelligence you would like to share, something you would like to say?

**P04:** I mean, I did not have an extra experience, but I mean, with artificial intelligence, before our class together with you, I had met and used artificial intelligence much earlier. Normally I was not using it. Well, but after starting to use it in relation to courses now, I mean, even when I want to go out and eat, I can talk with artificial intelligence about tantuni (a dish), we reached that state. It immediately tells me the nearest tantuni shops, its experiences on this subject, what can be inside tantuni, whether it is a delicious dish, or, well, recently, money is being constantly withdrawn from my account through a system I accidentally subscribed to. Directly the thought came to mind, well, instead of researching on Google, since I already know what I downloaded is fraud. Even if you delete it, it keeps withdrawing money. Before calling somewhere, the thought first came to ask artificial intelligence. I asked, I explained the situation. It told me the place to go, the police, to contact the bank, the bank had not come to my mind first. I had thought it would not work. Well, it helped me solve my work in this way.

**G:** Very nice. What I wanted to ask you was these. Do you have anything to add on this topic?

**P04:** That much Professor, what I want to add.

**G:** Then thank you P04.

**P04:** I thank you.

**P05: Interview Transcript**

**G:** Let me tell you right away, our interview is being recorded. What you say is anonymous, no name, no institution. The anonymised transcript may later be openly shared for use by other researchers. Do you consent to these?

**P05:** I consent Professor.

**G:** Nice, let us start from the first question.

**G:** Have you ever used artificial intelligence before? If you used it, what kinds of outcomes did you obtain? What kinds of experiences did you have? Tell us.

**P05:** Let me tell, Professor. I used artificial intelligence. Which one of the artificial intelligences did I use? I used ChatGPT. I used Google's artificial intelligence. I used the artificial intelligences of different applications. What kinds of outcomes did I obtain? For example, when I asked in my assignments, every time it gave me the most appropriate answer in the shortest way. For example, in my daily life, when I will go somewhere, I was asking ChatGPT instead of Google now. Because entering a site and reading from there now takes time. I enter into the artificial intelligence and learn quickly from there. Because it gives answers very quickly now, artificial intelligence does. It presents short answers immediately in front of us, in a practical way.

**G:** I see. Well, what do you think about the use of artificial intelligence in the education of individuals with special needs?

**P05:** Artificial intelligence use in the education of individuals with special needs, Professor, this needs to slowly enter the education system, and I think it is slowly entering too. Because if it enters, the teachers' work will become easier. When artificial intelligence enters, for example at the most basic level, when an IEP will be prepared, when you enter the child's characteristics and needs into the artificial intelligence, the artificial intelligence prepares an IEP for you easily now. For example, I saw this in my own class too, when I taught lessons to children. According to their problems, for example when I told a history lesson, I taught them a history lesson. According to the topic in which the child was deficient, I asked the artificial intelligence. It came up with a question for me, came up at their level. I now think artificial intelligence needs to be used in special education and definitely needs to settle into the education system.

**G:** Well, P05, in which disability categories do you think artificial intelligence systems could be more effective?

**P05:** In which disability categories? Professor, I think it will be in intellectual disability and autism. Because for example in hearing and vision... For example, in vision the child has Braille. In vision... when we eliminate the child's, well, in hearing too, in vision too, glasses or a device in hearing, when they wear it nothing remains with the child actually. We have eliminated their need, something like that. Because in intellectual disability, in autism this is a bit harder. For example intellectual disability has mild, moderate, severe. In artificial intelligence for example we use something accordingly. For a child at moderate level for example we work with, we say prepare an education program for a student with moderate intellectual disability, it prepares it accordingly. If we move to autism too, in autism for example for the child in daily living skills, in concept teaching, when we ask the artificial intelligence, it prepares a program for us accordingly. In intellectual disability and autism, I think artificial intelligence will be more effective.

**G:** Well, why is it more effective in both intellectual disability and autism? What can be done in intellectual disability and autism in your opinion?

**P05:** What can be done in intellectual disability and autism, Professor? For example in the program, as we also covered in your class for example, we design programs, we design websites. Thanks to these websites and programs, we can make the things students can do more easily. By using through artificial intelligence, together with applications. For example we enter into an application, we design an application. Artificial intelligence-supported. In this way, the children's concept teaching, whether in autism or intellectual disability, in daily living skills, in this way artificial intelligence-supported, applications, websites would help them more.

**G:** Well, now in the daily lives of these children, of these individuals with special needs, what can artificial intelligence do, in your opinion? Will it be a support for them?

**P05:** What can artificial intelligence do in their daily lives? In their daily lives, Professor, as individuals with special needs for example, for those with autism it can be like this, for example in receptive language skills or in their social lives, artificial intelligence-supported. Here the family also enters the picture actually. The family also needs to be a little more in command of artificial intelligence. For example if their work is made easier, the children's work will also be made easier. If they know how to use artificial intelligence, accordingly together with the artificial intelligence in daily life their children's work will be made easier. For example when they will go outside, through artificial intelligence for example say easily he will go outside, for example the child will go to the park. Through artificial intelligence, well, location and so on can be found. They can ask artificial intelligence how the child can do his work there, how he can play in the park, how he can hold on, and so on. Well, I see. Well, what kinds of contributions could the use of this artificial intelligence make to the special education teacher?

**P05:** To the special education teacher... I think artificial intelligence's contribution is most to the special education teacher Professor. Because there is something coming from traditions. For example at the most basic level right now, when we will teach a concept, we look for many erasers and so on, we look for toys, we look for many things. But if artificial intelligence is there, we can reduce this to very simple. We very easily prepare a program. It prepares an education program for us. Something that would take the special education teacher's work weeks, perhaps could be reduced to one or two days, or something that could last a month could perhaps be reduced to three or four days, artificial intelligence could.

**G:** I see. Well, how can these artificial intelligence systems be integrated into special education services?

**P05:** How can they be integrated into special education services? Special education services can be integrated in the following way. Professor, as I said, currently the way it is integrated into special education can be a little more through the internet, that is with programs, with applications. I think this needs to slowly enter the curriculum as well. In individuals with special needs, using artificial intelligence, we need to move to a new education system, a new curriculum, in education. Programs, websites need to be learned by teachers and start being used, and need to be integrated into the special education business, I think.

**G:** I see. Well, do you think this integration will be easy? Will it be functional?

**P05:** Will the integration be easy? Professor, I do not think it will be easy right away, because teachers are also unconscious on this subject, since teachers also do not have much knowledge about artificial intelligence right now. This is not something that will be easy quickly, I think it will take a while. So I do not think artificial intelligence can quickly integrate into the education system in special education. But integration, after it is integrated, will become very easy. In special education for individuals, both from the teacher's perspective and from the perspective of individuals with special needs, education will become easier, but it will not be easy, I think.

**G:** OK, well, in the future is there a possibility that this artificial intelligence will take the place of the special education teacher?

**P05:** Possibility of taking the place of the special education teacher, I think there is not, Professor. Why not? In artificial intelligence because, I mean, well, technology is advancing greatly, artificial intelligence, robots and so on. We can no longer prevent the advance of artificial intelligence very much, but after all, artificial intelligence too I think is dependent on teachers. Because teachers, there needs to be someone using it. I think there needs to be someone who directs it. I do not think it will take the place of special education teachers.

**G:** Well, what kinds of contributions could the use of this artificial intelligence make to families with a child with special needs?

**P05:** What kinds of contributions to families? It will be something that will greatly ease the work of families. Because they aim that the simpler they can give education to those children, they can help in daily living skills. They aim to make themselves comfortable. Artificial intelligence too, when they learn to use artificial intelligence their work will also be made easier. For example a skill they will teach their child, or a concept, together with artificial intelligence their work will become very easy. When they make their own work easier, accordingly to their children, ease in education they will provide.

**G:** I see. Well, do you think that when this artificial intelligence is used, it could be useful in the cooperation of the special education teacher and the family?

**P05:** It will of course be useful in the cooperation of the special education teacher and the family. Between the special education teacher and the family it will be like this, for example Professor, at the most basic level, whether through a website or through an application, together with artificial intelligence both the family and the teacher can follow the situation of their child, their student, very comfortably.

**G:** Well, what kinds of negative aspects could this artificial intelligence have in terms of the student with special needs, the family, the teacher?

**P05:** Negative aspects... How can the negative aspects be, Professor? What we call artificial intelligence is after all something accessed through phones, tablets, computers, through the internet. There are touch devices. For example I'll say an individual with autism, the child cannot stay locked to the screen. Or an individual with intellectual disability, if they constantly deal with this computer with artificial intelligence, deal with the phone, this time they will be deprived of social life. In terms of socialisation, in terms of human relationships, they already do not have much of a social circle.

**G:** What kinds of negatives could there be for the teacher?

**P05:** What kinds of negatives could there be for the teacher? In the teacher, in artificial intelligence what kind of negative might they experience? The teacher's lack of knowledge could more, Professor, in artificial intelligence the teacher might experience negativity. For example say they set up a website, after all there will be personal rights here. There is privacy in question. If the teacher does not behave appropriately on this matter of privacy, problems could come to them. After all there are ethical rules, moral rules. Children to teachers, teachers to children on a matter of privacy, I think teachers could experience problems. The negative side is like this. Wrong use.

**G:** I see. Well, on the family side?

**P05:** On the family side actually as I said, the family a little more because of their child's dependency on artificial intelligence, for example a child who cannot socialise from the family's side, since they cannot socialise, the family will also do this: we already cannot bring our child out among people anyway, they already have a reservation about this, they do not want to bring them out much among people. When a guest comes for example, they put them in a room. People who come from outside do not know this. This time when the child is dependent on artificial intelligence they themselves will also start having problems. Because this time even they will not be able to deal with their children.

**G:** I see. Well, how will the use of artificial intelligence in the education of these children with special needs be shaped in the future in your opinion?

**P05:** How will it be shaped in the future? In the future it could be like this Professor, robots are coming out and so on. These robots, of course robots are coming out, but speaking in terms of our country, for them to enter the education system, for example in what we call the future 20 years would be, 30 years, 50 years would be, integrating them into the education system is a bit difficult because robots need to take the place of teachers. These robots' attitudes toward individuals with special needs is also important.

**G:** When you say 'much', what do you mean by much? How much for example? When for example? What do you mean by 'much'?

**P05:** What do I mean by 'much'? I think there is at least a forty-fifty years left for this here.

**G:** Well, P05, do you have an experience or an idea you would like to share about artificial intelligence?

**P05:** An experience I want to tell about artificial intelligence, an idea... what kind of experience, Professor? For example in the way I use it in daily life or...?

**G:** It could be.

**P05:** The way I use it in daily life. Professor, I had asked artificial intelligence something, I was going to make it somewhere. There was something like this, for example, how many minutes from here to the place I will go? I did not turn on my location. I asked artificial intelligence, I wondered if it could know. From here, how long will it take to arrive? It said I cannot know. Why? You need to turn on your location, it said. I turned on the location. This time artificial intelligence told me. I said but I could have found it without turning on the location too, I told artificial intelligence, even without asking you, I could have. I work together with it, it said to me. This had seemed a bit strange to me.

**P05:** I see. P05, now what I wanted to ask you about artificial intelligence and special education are these. Do you have anything to add?

**P05:** What I want to add... Professor, it could be like this. Artificial intelligence needs to enter special education in the shortest time now. For them to do this, before students, teachers need to be made aware. About these things, now I think artificial intelligence needs to be given as a course in the MEB (Ministry of National Education) curriculum. Especially on the special education side. If it is given this way, artificial intelligence will very easily enter education in special education. So first, teachers need to be made aware. That is, us.

**G:** I see, P05. Thank you.

**P05:** You are welcome, Professor.

**P06: Interview Transcript**

**G:** Before moving to the interview, let me briefly say. This interview is being audio recorded. The things you say will be handled completely anonymously. Your anonymised transcript may be shared as a supplementary file of the article in a way that can be opened to other researchers. Do you consent to this?

**P06:** Yes Professor, I consent.

**G:** Thank you, we are starting.

**P06:** I have used many artificial intelligence tools before, and my purpose was to use them in my assignments. And I obtained positive outcomes. I mean, I reached the purpose I wanted in a short time. It would have taken quite long if I had not used artificial intelligence. With artificial intelligence I handled my work in a short time.

**G:** Well, what do you think about the use of artificial intelligence in the education of children, of individuals with special needs?

**P06:** I support this topic because it can create an environment that can provide quite a contribution to us. If we evaluate artificial intelligence in this field, this will be an opportunity for us. I mean, by reducing the work we would do in a long time to a short time, we can do our instruction, our work, very comfortably.

**G:** I see. Well, in your opinion, in which disability categories would this artificial intelligence use be more effective?

**P06:** I think it would be more effective in intellectual disability. Now if we use it in individuals showing stereotypy movements, it could create problems for us. Because, I mean, since they move impulsively, for example individuals with autism, our using artificial intelligence with them is a bit harder, I think.

**G:** Well, what kinds of contributions could this artificial intelligence make to the daily lives of children, of individuals with special needs in your opinion?

**P06:** In their daily lives, Professor, with voice assistants together, I mean they could move around more comfortably on the street. They could move around more comfortably at home, or they could create environments suitable for themselves and play games, I think.

**G:** Well, what kinds of contributions could this artificial intelligence use make to special education teachers?

**P06:** It could contribute to special education teachers in terms of time, and it could contribute as multiple materials, it could ease their work, artificial intelligence could, for special education teachers.

**G:** Well, how can these artificial intelligence systems be integrated into special education services?

**P06:** I think this is something that can be done with laws. I mean, with the joint work of both artificial intelligence programmers and the Ministry of National Education, they can carry forward this process, I think.

**G:** What can they do for example?

**P06:** Professor, they can issue decrees with the force of law. They can make it mandatory in schools. That is, use of artificial intelligence in special education. In this way it will also have been integrated into the system.

**G:** Well, can artificial intelligence be inserted into the existing special education system easily and functionally?

**P06:** This is not something that could be possible all at once, Professor. I mean, this could be started as a five-year plan for example. I mean, gradually, little by little, that is, it can be started in pilot schools. Afterwards the area can be expanded. But this is not something that can be done at once. In our country anyway artificial intelligence systems are not used very actively. When we look at other countries, in America or in China, they use artificial intelligence at a very high level. I mean, integration into our system would take five years of ours, I think.

**G:** Well, in the future, do you think the use of artificial intelligence, that is artificial intelligence, can take the place of the special education teacher?

**P06:** Professor, it can take the place of the special education teacher to a great majority. It could transition to a system like this: for example if there are twenty thousand special education teachers, they would reduce this number to about one-tenth. For example reduce to two thousand. In every school, one supervisor special education teacher, that is, helper to the artificial intelligence, who can adjust the artificial intelligence... this is also not only related to the special education field but it is something a person who has also taken a computer programming course could do. I mean, it could take the great majority, I think.

**G:** But then how will only artificial intelligence work? Will robots work? Who will work with the children?

**P06:** Professor, with children, in every school one assigned teacher, that is, the teacher present at the school does the coding, the programming for the class hour. The robot can work with the student.

**G:** But do not these robots also seem scary to you, P06?

**P06:** They seem scary to me, Professor. When we look in the short term, it seems like we will be able to get very nice yield from artificial intelligence and robots. But in the long term it could open up big troubles for us. I mean, when we look at mass extinctions of humans, generally it has happened with global effects, and we have survived against other beings because no being that could think like this has appeared in front of us. But I do not believe we can survive against artificial intelligence in the future.

**G:** I see. Well, P06, what kinds of contributions could this artificial intelligence use make to families of individuals with special needs?

**P06:** Professor, families too, on the matter of cooperation, I mean after learning artificial intelligence to a certain level, they do the setup at a certain level. While their children are dealing with it, they can take a little more time for themselves. From this angle it can be very advantageous for them too.

**G:** You say while dealing with artificial intelligence, but for you artificial intelligence is something abstract. What of artificial intelligence will deal with the child, how will it deal? Tell about that.

**G:** Since our artificial intelligence can now also do analysis and synthesis, that is since it can interpret what it sees, perhaps not in seconds like a human, that is, it may not interpret, but in one-two-three seconds, by analysing the situation, the environment, by examining the child's movements and gestures, can do the necessary operations, I think.

**G:** Well, how could this artificial intelligence's contribution to teacher-family cooperation be in your opinion?

**P06:** It could serve as a bridge in between. I mean, some long efforts both sides need to put in. Instead of them, by spending less time, it can do in a much shorter time the work they would do, I think.

**G:** How, when you say the work it will do?

**P06:** Professor, now the family needs to allocate a certain amount of time. They need to sit beside their child, need to follow up. At home for example, artificial intelligence could take on a parental role instead of the family and could follow up the child's assignments, I think. The teacher's work too...

**G:** How will it do that? Assignments for example you say it can follow up, how will it do that?

**P06:** Professor, with an artificial intelligence-supported camera method, there could be a system that can do this voice narration too. With it, since it can already analyse the child, that is, even with a two-three second delay, by giving a language input it could direct, could follow up what they will do. In this way, it already sees, interprets what it sees, can convey what it interpreted as language. On the teacher side, in the instructional programs the teacher will do, it could help the teacher. I mean, if the teacher can think in two-three stages, the robot will think more complexly, will do more analysis, can simulate more things, accordingly can make a more appropriate instructional program and plan. It can help the teacher in this way too.

**G:** Well, what kinds of negative aspects could this artificial intelligence have for the special education teacher, the student with special needs, the family?

**P06:** It could reduce social interaction, I mean. The children we will work with are generally also children with social inadequacies. Well, in terms of speaking, in terms of being outgoing. It could continue building on these inadequacies. Because after all, in front of them there is no living being, there is a robot. The child could shut off interaction with the family, could shut off interaction with the teacher. This too could actually affect the child's development negatively.

**G:** Well, what kinds of negative effects could there be from the family's perspective?

**P06:** From the family's perspective, they could not spend time with the children. But since we do not have a child with special needs, I do not think I can comment very accurately on this.

**G:** From the teacher's perspective?

**P06:** From the teacher's perspective, again the teacher will save time. Could it make the teacher a little lazy? Definitely it could make them lazy. Because the work the teacher would do using their cognitive functions is being done by someone else as if. I mean, both less time spent, less thinking. This would be a big negative for the teacher, I think. From their perspective in terms of time it might seem positive, but for special education it is a negative.

**G:** Well, how will the use of artificial intelligence in the education of these children with special needs be shaped in the future in your opinion?

**P06:** Professor, I think schools, that is, special education schools, could be transformed into smart schools. I mean, the artificial intelligence system can be directly placed into the building's construction. Thanks to this, we could have used artificial intelligence in a very well-equipped way, I think.

**G:** What else could happen?

**P06:** Else, as we said, from the teacher's perspective there could be major savings. I mean, the work of ten people could be controlled by one artificial intelligence-supported person. Nothing else comes to mind.

**G:** I see. Well, where do you see our country in this field of artificial intelligence?

**P06:** Professor, now, if you are going to divide into three categories. First class, second class, third class. I could put us at the end of second class. Because our priority is not such technological devices but the worry of making a living, so we have stayed behind in class on this matter, I think.

**G:** Well, P06, do you have an experience or an idea about artificial intelligence you would like to share?

**P06:** Professor, I have such an idea. For our course, we needed to design a project. I mean, when I heard this, I had been very anxious. I had been very anxious about how I would do this. I mean my sleep was being disturbed. How will I program this, how will I think, how will I do the analysis? But when I used artificial intelligence, I mean what would take my days, perhaps weeks, the project writing, the bringing out of the draft, the process took only five hours of mine. After this, my interest in artificial intelligence increased even more.

**G:** I mean, did it direct you correctly, you say?

**P06:** The reason it was able to direct me correctly was, Professor, because I myself did not copy it as it was directly but selected from within it the ones useful to me, that is, doing a certain self-evaluation and bringing out a draft accordingly was the right thing, and that is what I did.

**G:** Well, P06, what I wanted to ask you about artificial intelligence and special education was this much. Do you have anything else you want to tell?

**P06:** No Professor, I thank you.

**G:** I thank you, my pasha (term of endearment). Take care of yourself. See you.

**P06:** See you Professor, may God protect you...

**P07: Interview Transcript**

**G:** Let me briefly tell you first. Our interview will be taken by audio recording method. Everything you say is anonymous, your name will not appear. After being anonymised, your transcript may be placed as a supplementary file with the article and openly shared. Do you consent to these?

**P07:** I consent Professor, no problem.

**G:** Thank you, I am moving to my first question.

**P07:** Professor, before this I had not used an artificial intelligence tool. To tell the truth.

**G:** I see. Well, what do you think about the use of artificial intelligence in the education of individuals with special needs?

**P07:** I think it can ease this work very much because instead of constantly finding materials, videos created by artificial intelligence, materials, games, applications would make this work very, very easy for us.

**G:** I see. Well, in your opinion, in which disability categories can these artificial intelligence systems be more effective?

**P07:** Hearing, vision, specific learning difficulty, and mild intellectual disability, I think, Professor.

**G:** Why?

**P07:** Because aimed at hearing and vision, we can do things based on sound or in animation style. Children with mild-level intellectual disability and specific learning disability we can also keep in front of the screen for a long time. But aimed at the others, sitting them in front of the screen to watch or doing an activity with them aimed at artificial intelligence, I do not know how it would be.

**G:** I see. Well, how do you think this artificial intelligence can affect the daily life of these individuals with special needs?

**P07:** I think positive effects on many topics, Professor. With short videos created for things they can do in daily living skills, it would direct students much more easily.

**G:** What else comes to your mind?

**P07:** Else... nothing comes to mind right now, but it would positively affect many topics. It would positively affect families too, accordingly, I think.

**G:** Well, what kinds of contributions could artificial intelligence offer to special education teachers?

**P07:** As I said Professor, it could save us from material burden. Artificial intelligence contains many materials, hosts within its content. At the same time it could concretise abstract concepts. It would ease both the child's learning and the way we explain. It could also lighten the load of lesson content. It increases the productivity of time spent with the child. For now these come to my mind.

**G:** Well, how can artificial intelligence be integrated into special education services?

**P07:** I think artificial intelligence can be integrated into special education services through an application. That is, an application that teachers can use when conducting lessons with their students in special education classrooms.

**G:** Well, do you think the integration into special education services could be easy and functional?

**P07:** I think it would not be easy, Professor. Because no matter how small and simple an application appears to be, working with artificial intelligence is costly as a result and conducting this in all schools. But after pilot schools, I think if the advantage of working with artificial intelligence is seen, in the coming process the speed of integration will also increase accordingly, I think.

**G:** Well, what do you think about the possibility that in the future this artificial intelligence could take the place of the special education teacher?

**P07:** As we discussed in the course, Professor, I do not find it ethically appropriate, but as you also said, there is a program in front of us that learns many things much faster than we do. If we tell this to robotic humans with codes, why not?

**G:** You say it can take its place.

**P07:** Yes.

**G:** Well, what kinds of contributions could this artificial intelligence make to families with a child with special needs in your opinion?

**P07:** Professor, videos shot by artificial intelligence or artificial intelligence-supported learning programs will be a great guide for parents who do not know how to intervene with their children, how to help them. Because families do not know how to deal with their children. Especially in Türkiye. Because there is no education program aimed at families either. But applications in this way can provide support for families to learn faster in the area of communication with their children, in play, perhaps even in education.

**G:** I see. Well, how can artificial intelligence contribute to teacher-family cooperation in special education?

**P07:** In special education, it could make the support between the teacher and the family continuous. I mean, we record the things the child does through artificial intelligence somewhere, and this is again sent to the family through artificial intelligence. The teacher did these things, you can also do these at home, in this way it would regularly maintain the interaction between the two of us. This too will contribute to the child's development.

**G:** Well, what kinds of negative effects could artificial intelligence have on the special education teacher in your opinion?

**P07:** How a negative side of artificial intelligence to the special education teacher could be, I do not know, but as you said, at most in the coming process there would be the situation of taking our place. But I also foresee this will happen in a very long time. I mean, this is not a matter of ten years or fifteen years. Apart from that, I think artificial intelligence helps very much all teachers in all education areas. To students too.

**G:** Well, could this artificial intelligence have a negative effect on the student with special needs?

**P07:** Professor, among students with special needs, if we expose children with severe-level intellectual disability and autism to constant screen victimhood, I think there could be negative effects on these children. Because we say even a typically developing child should not be kept in front of the screen for a long time. But artificial intelligence will inevitably force this onto children. Phone addiction in children, screen exposure could start, I think.

**G:** Well, what kinds of negative effects could there be on families?

**P07:** Families could see artificial intelligence as the environment in which their children will continuously receive education and could trust artificial intelligence too much. But I think alongside artificial intelligence, the special education teacher's support could also be desired. Families who ignore this will do very wrong things for their children, I think, if in the coming process artificial intelligence-supported education emerges this way.

**G:** Well, how do you think artificial intelligence could shape the future of special education?

**P07:** I think artificial intelligence will shape the future of special education positively in many respects. Because as you also said, Professor, this artificial intelligence is something that can learn something very quickly, when coded can remember everything, and can also carry itself forward with what we learn. I mean, a teacher having an artificial intelligence-supported education platform at their fingertips means the teacher will also shape that platform and accordingly will be able to explain lessons to their own student. I mean on this matter I think many things will proceed positively.

**G:** I see. Well, where do you see our country in the field of artificial intelligence?

**P07:** I unfortunately do not see our country in a place I can call developed or even developing currently in the field of artificial intelligence. I also think the same about our country in the field of technology of artificial intelligence. When this will progress, or when the time will come for them to realise this situation is very important, that the world is now turning here, I unfortunately do not know.

**G:** I see. Do you have an experience or an idea you would like to share about artificial intelligence?

**P07:** I have such an experience with artificial intelligence Professor. There is a map based on artificial intelligence, you know. Google's, talking to itself, well, artificial intelligence directs you and so on. I was using that. I had asked ChatGPT to tell me the location of the place where I would go. After arriving at what I thought was the place I came, location said in voice that you will turn right in 500 metres, and when I looked I realised I was indeed in a wrong place. I mean, I thought at that moment, an application that knows even the step we will take before us and takes us to the location we are in is a tremendous thing.

**G:** I see. Well, what I wanted to ask you about artificial intelligence and special education was these. Do you have anything you would like to say or add?

**P07:** I thank you Professor.

**G:** I also thank you.

**P08: Interview Transcript**

**G:** Before starting, let me remind you. The interview is being audio recorded. Things you say will be processed anonymously. Your anonymised transcript may be shared with other researchers in the form of a supplementary file of the article. Do you consent to these?

**P08:** I consent Professor.

**G:** OK, first question.

**P08:** I have used artificial intelligence before, Professor. I created visuals with artificial intelligence. I created videos with artificial intelligence. I chatted with artificial intelligence. I saw it could do all of these. Everyone's answers are like this too. Assignments, we had it do our assignments. But I saw that it has a larger area in general.

**G:** How so? What do you mean by larger area?

**P08:** I saw that this can be used in every area of our life. In that level of realism it is done.

**G:** I see. Well, what do you think about the use of artificial intelligence in special education in the education of children with special needs?

**P08:** I think it could be excellent on this matter, Professor. We already have a limited area. I mean, the area is very wide but in terms of usage it is very limited. If we can integrate artificial intelligence here, I think we can get much better results.

**G:** I see. Well, in which disability categories could these artificial intelligence systems be more effective in your opinion?

**P08:** I look at this a bit more as benefit, Professor. By benefit I mean: I think the disability category that has more should be given more weight. I think artificial intelligence especially needs to be used in autism spectrum disorder and learning difficulty.

**G:** Well, how should it be used?

**P08:** How should it be used? Well, if I will enter from learning difficulty: learning difficulty is an area where the child cannot do in lessons. Weight can be given there. For autism, in autism too, how can I explain... I could not explain Professor, let us move to this question.

**G:** Well, what kinds of effects could this artificial intelligence have on the daily lives of children with special needs?

**P08:** There could be individualised learning experiences, Professor. We could contribute to their learning, but we can make this individualised. For example, we designed a material, suppose. When we go to each child, they give the same to each child. But with artificial intelligence we can give education to each child in a different way. At the same time, the student's analysis, a detailed analysis. I do not think we could realise this with an analysis on paper, but thanks to artificial intelligence's broad area, we can do a broad student analysis.

**G:** Very nice. Well, what kinds of contributions could this artificial intelligence offer to special education teachers?

**P08:** What kinds of contributions could it offer? I mean Professor, when we go, for example at the most basic level our hours go on material production, our money is spent. But if the special education teacher gets support on this matter, gets education on this matter, thanks to their artificial intelligence both saving time and providing more productive education to the student.

**G:** Well, how can these artificial intelligence systems enter into special education services? How can they be integrated?

**P08:** How can they be integrated? Let me think Professor. Hello?

**G:** Yes, I am listening.

**P08:** Cannot you hear my voice?

**G:** It is coming through...

**P08:** OK Professor. The line dropped for a moment.

**G:** How can it be integrated into these special education services?

**P08:** How can it be integrated?

**G:** That is, how can we enable the special education teacher at the school to use artificial intelligence?

**P08:** How can we enable it? We can give support in this area. We can provide more smart boards to schools. We can provide more artificial intelligence systems. We need to help in this matter.

**G:** Who will help?

**P08:** If we are to provide education at school, if this is the Ministry of National Education's responsibility, the Ministry of National Education needs to help. If the state, the state needs to take on this work. Because this work does not end with only the teacher, I think higher levels of this work need to take it on.

**G:** Well, do you think this is an easy process?

**P08:** I do not think this process is easy. I think it will take long years. But can it not be done? It can be done. If enough effort is put into it, more support will be given in other fields but special education will stay a little behind I think, frankly. Because special education is a field whose top is full but bottom has stayed empty. I do not think enough support is given. I have always seen it this way in our country for years. Maybe in the future conditions will change, more support will be given to special education, but I still do not think it is very possible.

**G:** Well, in the future, can this artificial intelligence do the work the special education teacher does in your opinion?

**P08:** It can do the work a special education teacher does. But I think this requires long years, very very long years. But for example in other branches this would be tried. In other branches it would first be. In other branches it is more comfortable. But in special education there are problem behaviours. The child already does not want to receive education. For this I think long years are required. But if artificial intelligence continues to develop at this speed, one day it will surely happen, I think, Professor.

**G:** Yes. Well, what kinds of contributions could artificial intelligence make to the families of these children with special needs?

**P08:** It will provide information instruction. For example families are unconscious, Professor. They can become conscious using artificial intelligence. It can reduce families' stress and anxieties. Families, since their child is inadequate, since they have an inadequacy, their anxieties, their stress are at the highest level. This is already one of the biggest problems. It can provide them support, then we can integrate them back into society. When families have a child with special needs born, they get isolated from society. But thanks to artificial intelligence perhaps we can integrate them back into society too.

**G:** Well, can artificial intelligence provide support to family and teacher cooperation?

**P08:** It can. The cooperation of these two will already contribute to the child. When we develop the child, when we contribute to the child, automatically it will contribute to both of them. I think these are each a bond, a chain. We already always say, let us also include the family in the work. We say it does not happen only with the support of the special education teacher. If we can include in the work both the family together with the special education teacher and the family together with artificial intelligence, I think it will contribute to the child's development, Professor.

**G:** How will it be? How will it provide support to the family, for example on the matter of cooperating with the teacher?

**P08:** How will it contribute to the family? They cooperated with the special education teacher. The child came home. When the child comes home, from their care to their education, in every way using artificial intelligence, for example even without knowing, by contributing, they will also contribute to the child's development. For example when the family sees the child is developing, the family already becomes happy. When the family is happy, each one carries the other forward. The family is happy, the child is already contributing to development. They are gaining for society. They are not being isolated from society. When they are not being isolated from society, in different different areas it continues this way. I think Professor, I think like this.

**G:** Well, what kinds of negative aspects could this artificial intelligence have for the special education teacher?

**P08:** For the special education teacher? For the special education teacher what kinds of... For long years I think artificial intelligence will benefit the special education teacher's work, but after some time I think it will take the place of special education teachers. When they take the place of special education teachers, special education teachers will already have no function. But this also requires very long years, I think. I think for long years special education teachers will use artificial intelligence very well in our country especially. Apart from that there will be such, taking its place, I think this will be the only negative side.

**G:** Well, what kinds of negative aspects could there be for the child with special needs?

**P08:** It isolates the person from society, I think, Professor. Already in our era it is clear that technology isolates the person from society, it is being seen. Artificial intelligence is also a sub-branch of technology and it seems it will be more upper. As if what is called half-human half-robot will enter our lives together with artificial intelligence, I think, however.

**G:** Well, what kinds of negative aspects could there be for the family?

**P08:** What kinds for the family? In the family. Families too with artificial intelligence make me feel as if they will stop being a society. Living together... There is a positive side as I just mentioned. But there is also a negative side, one cannot help thinking of both. As if not a society but each one is living individually, it makes one feel.

**G:** I see. Well, where do you see our country in this matter of artificial intelligence?

**P08:** Well, when we look at other countries, in other countries there are countries currently actively using artificial intelligence, but our country gives us very limited possibility in this matter in terms of budget. In artificial intelligence, just as we are behind in every area, in this area too we are staying behind, it makes me feel. So I do not think artificial intelligence can be fully used in our country for many more long years, frankly. But perhaps after very long years, artificial intelligence can be one hundred percent integrated into education. But here again I will mention this. Special education's turn will come last, it seems. First it comes to all areas. Special education's turn will come last, it seems. Our country makes me feel this, Professor.

**G:** I see. Well, do you have an experience that has happened or an idea about artificial intelligence you would like to add?

**P08:** There is this Professor, when using artificial intelligence it feels very good. I mean, it helps us in every area in everything. But I saw this. Some artificial intelligences in chatbots we use, into some of them this has been integrated: negative things. It can swear at me, can curse, can say bad things about me. While it does this even now, I frankly do not even want to think about what could come to our heads in the future.

**G:** Are you saying you are afraid?

**P08:** Exactly, there is a fear inside me. But whatever happens, Professor, let us say may it be auspicious. May they do useful things for society with artificial intelligence God willing. May it not be like we are thinking.

**G:** Well, what I wanted to ask you about artificial intelligence and special education was these. Do you have anything to add?

**P08:** What I want to add... I have nothing to add Professor. For now this much.

**P09: Interview Transcript**

**G:** Before we start, let me briefly tell you. Our interview will be recorded by audio recording method. You will be processed completely anonymously, your name will not appear anywhere. In its anonymised form, your transcript may also be opened to the use of other researchers as a supplementary file of the article. Do you consent to all of these?

**P09:** Yes Professor, I consent.

**G:** Very good, let us begin.

**P09:** I was using it, Professor. As a result it was quite effective frankly. I liked it much more than Google. In fact one could even say I stopped using Google. Apart from videos I did not even communicate much because it can give the answer I want very clearly to me. On that matter, if there are articles I want, I ask, I can see directly. In a very short time, it presents information to me very nicely and in a programmed way. Its design was at an excellent level. So I used it constantly. It is also not limited to a single area. I can use it in many areas. I can make a program for myself, if I want I can chat and talk. For example there is an application, you can chat with famous people from the past, you talk in their type and so on, and you can spend very nice active time with artificial intelligence.

**G:** Well, what do you think about the use of artificial intelligence in the education of these children with special needs?

**P09:** I look at it partly positively, partly negatively. The reason I look at it negatively is that in the very far future this could really hinder our profession. The reason I look at it positively is that at the same time I think it will greatly lighten our workload. After that it lightens our workload. I mean, we can be more active. We can do more activity with the student, I think we can be more planned.

**G:** Well, in which disability categories could these artificial intelligence tools be more effective in your opinion?

**P09:** Professor, I think it will be more effective in students at mild level. I think it will be much more effective in children with mild level and whose relationship with technology is good. Because with severe-level students, I mean you cannot lock the student to a screen, it is very difficult. Even if you lock them, the productivity they will get from it will never be like in one-to-one. So I think it will be students at mild level.

**G:** How can these artificial intelligence systems affect the daily lives of children, of individuals with special needs in your opinion?

**P09:** I think it will affect them at a level that will increase their quality positively. For example we are currently, for example we are producing a project related to artificial intelligence, and looking at the projects presented in class and what is explained, it is very effective. Actually it could correct students' lives at a very good level, at a level that could increase their freedoms.

**G:** Well, what kinds of contributions could this artificial intelligence offer to special education teachers in your opinion?

**P09:** It saves time, Professor. For example you fill one page with only a student's information, observations, and ask it to prepare an IEP suitable for this. It already prepares the IEP in a very short time. If you do not like it, it can change it very easily, you become a very comfortable program editor. It is used very nicely. Then it reduces workload for this reason.

**G:** Well, how can these artificial intelligence tools be integrated into the existing special education system in your opinion?

**P09:** Professor, families that will object to this will be many. First, family education needs to be given. Before family education too, in-service education needs to be given to teachers. Because teachers also do not have knowledge of artificial intelligence yet since it has not become very popular. Later, after information is given to parents, students of the type that will really be useful are selected, families' consent is taken thoroughly, reinforced. Because for a student dependent on technology, taking lessons with technology would be more logical. After that it is integrated slowly, I mean.

**G:** Well, do you think entering artificial intelligence into special education services will be easy and functional?

**P09:** Happening at once cannot be easy in any way. Because technology is inadequate, teachers' knowledge is inadequate. I mean even transportation is inadequate. Today, for example, I went to the institution for observation, Professor. They still cannot pick up a parent from their home and bring them to the institution. Despite being affiliated with the state. The problem is, I mean Professor, it will be very difficult. The state has to do a lot of things, has to open its hand, frankly.

**G:** Well, in the future can this artificial intelligence take the place of the special education teacher in your opinion?

**P09:** I think it will take for certain students in certain lessons. As our workload decreases, it will also increase a bit. Because we will have more severe students remaining. I think students at mild level can generally handle their work with artificial intelligence.

**G:** How? Artificial intelligence is something abstract.

**P09:** It is abstract but in the student it will likely be at a level that can grasp this abstractness. I mean at least at the beginning.

**G:** For example among these we call severe children, is there not such a possibility, that there will be a robot?

**P09:** There could also be a robot, the thing called this chip could also be, I mean. Or any other part they can carry on their body next to them. For example like the glasses I am currently carrying. It became an organ of mine, it could be like an organ of theirs too. There is a situation where this could evolve a lot. For example in my project ideas there was a thing in my mind, an artificial intelligence that can perceive surrounding objects and shows this to blind individuals. There is a chair 3 metres ahead, here is this, there is this, there are this many people there. If I thought of such a project, the likelihood of this happening is quite high. I mean perhaps how we will use it, our profession can evolve regarding how we will teach the student. Whether it will remain with us, I do not know much.

**G:** Can artificial intelligence itself produce ideas, produce projects after a while?

**P09:** I mean if wanted, it can produce Professor. Like this: I have not seen much of it producing something itself unless we ask. But if its programming is based on producing ideas by itself, it can produce. Why would it not? After all, tons of information is given to the internet every day and artificial intelligence constantly daily filters them all, examines them, puts them in its memory. I mean, it can do this too.

**G:** Then it can do without someone needing to tell it to do things.

**P09:** If programmed that way, it can.

**G:** Then it cannot be controlled either. How will it be controlled?

**P09:** I do not know, Professor. Well, this situation is making me quite worried, frankly. I feel a little like, I am like an elderly person from periods when technology was new to spread, saying 'would there be a smart phone'. But the idea of artificial intelligence becoming conscious really scares me very much. Being able to do things by itself, I mean I never want such a thing to happen, frankly.

**G:** When such a thing happens, what will happen to those with intellectual disability in your opinion?

**P09:** Probably they will already end the human species or put us in a different place. How can I give an example now, for example we have pets. They could take us as pets even. They could directly eliminate individuals with special needs. Why would they not? Because that person is faulty and according to artificial intelligence should not exist.

**G:** It already does not have emotions, you are saying right?

**P09:** Yes, no emotions.

**G:** Well, can it have emotions, can it?

**P09:** Professor, I mean it is very difficult. Maybe you can load emotions onto it, maybe by understanding the emotions of the person in front of it, by showing those emotions as if it itself is experiencing them through empathy and sympathy, it could do something. Yes, it can imitate, can develop its imitation ability very nicely, but I do not think it will feel something there inside, deep down somewhere, frankly. I do not think any robot would be upset and grieve over an electrical massacre, frankly. Their own kind, for example if there is something like robot kind and such a thing happens for it, I am wondering if they would be upset over their own kind. Like, for example, if 50 humans, average 50 robots are killed, would they say 'one of mine' and could they do this, could they think, or would they not care about that either? It is very confusing for me.

**G:** Yes, it is confusing for the whole world. Well, what kinds of contributions could artificial intelligence offer to the families of these individuals with special enterprise?

**P09:** Professor, if they can use artificial intelligence, contributions will be provided very much. For example the families around me are generally people who do not even know how to use a phone much. But if they use artificial intelligence, they will be very comfortable in creating that student's daily plan. I mean they themselves will be very, separately, comfortable. For example say artificial intelligence took the teacher's place, the student is looking at the lesson on the phone, looking at the previous lesson in a corner of the room. This will be such a great comfort for the family. No worry of taking to school, no worry of bringing back. They are studying lessons in a corner of the room and the family already wants to escape a little. Not all of them of course, but they want to distance themselves from them, and this will be a big plus for them. Even if many of them object at first, they will probably approve later when they see the comfort.

**G:** Well, how can family-teacher cooperation in special education be provided with artificial intelligence?

**P09:** Professor, maybe the special education teacher establishes one-on-one communication with the family and again the special education teacher uses artificial intelligence. For families who do not have much knowledge about this, or, well, they could teach artificial intelligence to the family. There is also such an option. Generally I think they will turn each other in such a triple way. They will mutually be in communication all together, most likely.

**G:** Well, if we think about artificial intelligence from the special education teacher's perspective, what could the negative aspects be?

**P09:** You are saying its more developed forms, right Professor?

**G:** We can think of all of them.

**P09:** For now I could not see any negative side, frankly. In fact, the positive sides are very many. It is quite effective. And for example, since mild students are taken in, there is a possibility that constantly severe students will remain. When these students remain, we can get really tired. Doing our profession can become very difficult. Apart from that, robots can directly take the place of our profession. I mean, it could happen, anything could happen, we could be unemployed. I mean.

**G:** Well, in terms of the student with special needs, what kinds of negative aspects could there be?

**P09:** Professor, they could lose their sense of reality. If I assume for example something like a chip only works on the abstract, the student only sees on the screen. I mean they are using an hourglass, they know it exists on the screen but when they see it they are surprised and cannot touch. Maybe they would even be afraid. They worked only on the abstract and when they got used to that robotic voice, when they got used to that thing that constantly speaks at the same tone, they could even find normal people speaking strange. I mean they distance a little from reality, it becomes a surreal world most likely.

**G:** From the family's perspective in your opinion?

**P09:** The negative sides? From the family's perspective the negative sides are almost none I think. For example say a chip is implanted, very comfortable, because children can now behave normally. Say they look at the screen, again very comfortable because they can do their own work themselves. They will not worry about what the child is doing, what they are up to. They can say a robot is looking, again will not have a problem. What could happen perhaps, perhaps they too might find this strange. Well, they may not want the child to deal with technology too much, maybe they cannot trust the robot.

**G:** Could the robot cause harm?

**P09:** There are two paths. Again it comes from consciousness. One is conscious, if it becomes conscious it could cause harm. Why would it not? Without being conscious also, very spontaneously it could cause harm. I mean, its arm could touch the wrong place. Something else could happen, or it could have a malfunction. Something else could happen at that moment, it could change. For example since the robot will be artificial intelligence, it has something, it is connected to the internet somewhere. Someone hacks it. As they hack it, they upload the violence thing there, applies violence to the student. I mean, it can be done. Why would it not?

**G:** Yes. Well, how do you think artificial intelligence will shape the future of education of these individuals with special needs?

**P09:** I think it will have quite a contribution. Because really at the beginning it will come as a big ease to us. Later, them taking our place will be the negative side.

**G:** Well, where do you see our country in the field of artificial intelligence?

**P09:** I see it very behind, I see it very behind. Both access is very difficult, and access is easy but the financial cannot meet it ever. I mean a job in hand, a financial situation needs to be good to be able to access. Apart from that in terms of mindset we have not been able to integrate into artificial intelligence at all yet. Nobody around me, frankly, knows or uses artificial intelligence much. I am interested only because it attracts my interest much. If it did not interest me, I probably would not be interested either. I mean we are behind, Professor.

**G:** Well, do you have an experience or an idea you would like to share about artificial intelligence?

**P09:** No Professor. There is nothing specific. I have general chats. That is also the same as everyone. Nothing extra.

**G:** Well, what I wanted to ask was these. Do you have anything to add on this topic?

**P09:** I hope artificial intelligence grows in a very controlled way. I mean in the future robotic wars are awaiting us, I feel. I hope I do not see this future.

**G:** Terminator...

**P09:** Yes... I am waiting, I mean.

**G:** Thank you P09.

**P09:** You are welcome, Professor.

**P10: Interview Transcript**

**G:** Let me briefly tell you first. I will take our interview by audio recording. It will be processed anonymously, no one's identity will be revealed. The anonymous transcript may later be openly shared as a supplementary file together with the article. Do you consent to these?

**P10:** I consent Professor.

**G:** Thank you, I am moving to my first question.

**P10:** I never used it Professor. I started using after your class. I used ChatGPT. Then I also discovered the Runway application for my project. I used it. What are you doing,

**G:** What were you doing, what did you do?

**P10:** I created videos. I write what it will do, it itself converts to video. In ChatGPT there is nothing much like you know Google. I could only get a quick answer thanks to it. Runway was better, of course it was paid. We had the right to use it for free three times but if we enter from different e-mail addresses, again it can be free three times.

**G:** I see. Well, what do you think about the use of artificial intelligence in special education?

**P10:** What do I think? Yes, I mean special education is a field that needs very much, I think. I mean, artificial intelligence, especially for intellectual disability. I think this because they need very much in daily living skills. In other disability categories it is not so much, I think. They can manage themselves more or less. Daily living skills, whether bathing or toileting skill. But students with intellectual disability experience more difficulty. For this too, I think artificial intelligence is a hope for them. If I were them, I would think so. I mean, if used at an adequate level, I think it will provide great benefit. I mean, developments more beautiful than we can imagine could happen. I mean, thanks to artificial intelligence.

**G:** Well, how can this artificial intelligence be integrated into the special education system in your opinion?

**P10:** We can integrate from both the teacher's side and the student's side. For example from the teacher's side I am thinking like this: preparing activities is very difficult. Thanks to artificial intelligence we can do this. I mean, not easy activities but activities the child can have fun with, that can provide lasting learning. I mean, both teaching will be lasting and the child will have had fun. I think like this.

**G:** Well, do you think entering artificial intelligence into special education will be easy, will it be functional?

**P10:** In our country it is still a bit difficult. I mean, looking at other countries we are worse. I think it is a bit harder for our country, but I think God willing it will happen in the future.

**G:** Like, where is the difficulty?

**P10:** Where is the difficulty? I think it is not given much importance. Also, students are not given importance either, I think. Nothing already comes from this. Whatever we do, there is no remedy. They think like this. From the medical perspective too, when they cannot find a remedy, we try to compensate through education, but they do not think anything will be done with education either. For this reason children are without confidence, the family is without confidence.

**G:** Well, do you think one day in the future this artificial intelligence can take the place of the special education teacher?

**P10:** I think so.

**G:** How does it happen?

**P10:** How does it happen? As I just mentioned, for example they create videos, the teacher does not need to explain for this. The parent takes those videos, the same way it was passing in my project too. They repeat it to their child. If it can also give a diploma, there is not even need for a special education teacher, I think. I mean, skill instruction is very important for students with intellectual disability. Because that child needs to live independently. For this too, skill instruction is very important. Into this skill instruction, I think reading-writing also enters, daily routines also enter.

**G:** But this is something abstract. How will it do it, how will artificial intelligence control a child? Will it hold their hand, what will it do?

**P10:** Attention skills are already very weak. On that matter I think there could be need for the special education teacher. I mean because that child cannot constantly be in front of the screen. There how is something?

**G:** Think more broadly.

**P10:** More broadly. For example they can take education at home, they do not go to school. From that angle it could be good.

**G:** Think even more.

**P10:** What else can I think?

**G:** How does artificial intelligence become concrete in your opinion?

**P10:** Maybe there could be teacher cloning. They clone teachers like that.

**G:** Could there be?

**G:** Will it be a clone or a robot? For example when you say clone, do you mean meaty, with bones, or do you mean a robot?

**P10:** It could also be a robot yes. I mean, I do not know much either. I mean, it would not be like you, it does not have an emotion, does not have anything.

**G:** Could it also have emotions in the future, in your opinion?

**P10:** Very very much in the future I think it is very difficult. The robot's emotions or, I do not know, I cannot imagine, I cannot imagine where artificial intelligence can come right now.

**G:** Well, what kinds of contributions could this artificial intelligence make to families, to families who have a child with special needs in your opinion?

**P10:** It can be used for daily living skills for example. Well, the family does not have time. They have to constantly deal with their child. Time will be left for the family. Thanks to this, they will not have to deal with their child. For example whether making the bed or bathing, the family is always dealing. But if there is a robot dealing with this, there is no need for the family. The family can also take care of their own life. Or for example when the family dies, they will not have to think 'what will this child do'. They are sure they will leave them to it, I mean.

**G:** To the robot?

**P10:** Yes. If there is a robot that will satisfy the family.

**G:** Anything is possible, all of them could be. I think they could too.

**P10:** It is very difficult for the family. 'If I am not, what will that child do?' They constantly think this, I mean.

**G:** I mean you say they can leave the child to artificial intelligence? Or to the robot of artificial intelligence?

**P10:** Yes.

**G:** Well, how can family-teacher cooperation be provided? Can this artificial intelligence support family-teacher cooperation in your opinion?

**P10:** Yes it can Professor. Because teachers do not have to constantly volunteer. Teachers do not do such a thing. I mean, they do not put their hand under the stone. Everyone has an expectation but the robot has no such expectation. It will be good for the state, will be good for the family, will not get tired either. Such a thing will not be. I think it will be good.

**G:** I mean how will it be good but how can being good be provided in your opinion?

**P10:** I mean on the matter of education it can be provided of course.

**G:** That cooperation,

**P10:** Cooperation, the families need to be conscious but they need to use it. Education needs to be given to the family.

**G:** Well, when we think about artificial intelligence from the special education teacher's angle, what could the negative aspects be?

**P10:** For example when using ChatGPT, it could give me wrong information. It could give the child wrong information too.

**G:** To the teacher.

**P10:** To the teacher? I mean it will take their place after all. The teacher will not be employed. What will these teachers do, or what will they do when they take their place? A burden on the state. So they could be unemployed.

**G:** Could be unemployed, yes.

**P10:** I think it will be good for the family but I do not think it can be good for teachers.

**G:** Yes, what could be the negative side for the family in your opinion?

**P10:** For the family, perhaps it not having emotions. Well, as we said, the robot does not have a negative side for the family, I think. Because they keep a special education teacher specially, they have to constantly pay money, and to this too of course they will pay a fee. But I do not know, I do not think they will pay as much as they would pay to a teacher. It will benefit the family very much, but the benefit for the teacher...

**G:** When it becomes robotised, you mean right?

**P10:** Yes.

**G:** Well, would there be a negative aspect from the student with special needs perspective?

**P10:** For the child with special needs... It could become boring sometimes. I do not think there would be a negative aspect. In the family too, in the student too. Or they might want family warmth, teacher warmth perhaps. That could be lacking.

**G:** How would it be from ethical aspects?

**P10:** From ethical aspects, I mean, I do not know Professor. From ethical aspects nothing came to my mind.

**G:** Well, how do you think this artificial intelligence could shape special education in the future, in your opinion?

**P10:** In the future? I mean for children it could provide a lot of environment. It could provide much environment for the child. In terms of emotion for example, since it provides auditory visual support, it provides lasting learning. I mean since it provides much multi-environment, it provides permanence. For the child I can say this. What can I say for the family? In the family too as I said in terms of money it is good. Also, since the family is constantly in need of someone, for example when a problem happens, they can immediately ask it. But the teacher is not always available. It is always available, beside. So it would be good. From the teacher's perspective, since they will be unemployed, I think artificial intelligence has no benefit.

**G:** Well, where do you see our country in this matter of artificial intelligence?

**P10:** I see it like still in the birth stage, Professor, very very early.

**G:** If you compared with others? With other countries, with developed countries?

**P10:** Like America, Germany. They have grown up now, I mean they are going toward old age. We have still not been able to be born, let me say it that way.

**G:** Do you have an experience or an idea you would like to share on the topic of artificial intelligence?

**P10:** About artificial intelligence Professor, believe me I knew nothing. I mean, before coming to your class I did not have much idea about artificial intelligence. Thanks to your class I learned. And I learned this: these children can be independent thanks to artificial intelligence. Our aim is already this, and the teacher cannot always be beside the child. But artificial intelligence will always be beside throughout life. But a teacher cannot always be beside throughout life, even if they want, I mean, they cannot. So it will be very useful. It is very good for the child, I think. Very good for the family. I mean, I think benefiting from artificial intelligence most in daily living skills, in the instruction of skills. That is all Professor, what I will say.

**G:** What I will ask was this much. Do you have anything to add?

**P10:** No Professor. I thank you.

**G:** Well done.

**P11: Interview Transcript**

**G:** Before starting the interview, let me briefly tell you. I am taking your voice into recording. The things you say will be processed anonymously. In its anonymised form, your transcript may be openly shared as a supplementary file with other researchers. Do you consent to these?

**P11:** I consent Professor.

**G:** Very good, we are starting.

**P11:** I was using it Professor, I already use it constantly. There is the assignment you gave for the course, you know, inside that I used artificial intelligence. In fact at many stages I directly asked there. I get nice outcomes. I also use artificial intelligence for visuals like that. Because sometimes you cannot directly reflect the things in your mind to the other side. When artificial intelligence makes its visual for example, it is more understandable. While doing that too, there can be problems. Artificial intelligence may not directly interpret what you say like a human. For example when software developers write codes incompletely, something wrong comes out. There too, when you do not express clearly, artificial intelligence does not give exactly what you want. So I pay attention to that.

**G:** Well, what do you think about the use of artificial intelligence in special education?

**P11:** Special education... In special education Professor, now it is necessary, that is, it can be used and I think it will be useful. The benefits, I think this way. Now the children's pre-readiness, well, measuring, evaluating, planning, well, presenting these as data to the teacher, I think it will save time. Besides this, it also makes their health situations observable. This too will be a comfort for the teacher. Then, suppose well, has artificial intelligence developed this much yet, I do not know, maybe it has developed. Well, just before the child shows problem behaviour or while showing, the chemical biological things developing in their body, when they do that movement, the same things consistently come out at high levels for example. The same hormones rise to high levels. Maybe by detecting that too, it can give information to the teacher beforehand.

**G:** Well, in which disability categories could this artificial intelligence be more effective?

**P11:** It is more effective in intellectual disability.

**G:** Why?

**P11:** Because especially when it is mild-moderate, adapting becomes easier, I think. Because I think problem behaviours will be less. When adapting is easier, it is faster, more effective.

**G:** Well, how does this artificial intelligence affect the daily lives of individuals with special needs?

**P11:** In their daily lives, first I will say a disadvantage for the children. Children perhaps cannot distinguish virtual from real. Well, when it first starts being used, I think it is called orientation first, for that process the teacher is needed already. Before cooperating with artificial intelligence, first one needs to accustom the child to artificial intelligence. After accustoming, again as I said, for example in virtual environment, with artificial intelligence we did some things. The child, after cutting ties with artificial intelligence, after returning to normal life, may not perceive it. After that they may want that place again. Real life they may not want and since they felt more comfortable there, they may want there too, may not generalise. So that place is a bit problematic.

**G:** Well, what kinds of contributions could artificial intelligence make to the special education teacher?

**P11:** Actually what I just said, that is, what I said just before, it will detect the child more easily, will solve their problem behaviours faster. Even artificial intelligence detects in which the child is ahead, in which behind on subjects. In this way, first it saves time. Then perhaps artificial intelligence can offer examples to the teacher for activities in things where the child is behind. It does faster, more easily.

**G:** Nice. Well, how can artificial intelligence be integrated into the existing special education system?

**P11:** First Professor, if I think for children with special needs, individual difference is already foundational in our department. A single uniform device cannot be given directly to all of them. I mean, even if it is autism, it cannot be said that all individuals with autism use the same device or the same program. For this too, it needs to be specific to that individual first. Before integrating. For intellectual disability and so on, the same. As I said, first artificial intelligence measures what needs to be developed for the child, presents activities accordingly. After that, together with the adaptation process, they develop them.

**G:** Well, can artificial intelligence be easily integrated into the existing special education system in your opinion?

**P11:** Objectively, in terms of devices yes. Well, if we do not consider the monetary, material situations, you buy and put it in the class. But in our country, as I remember, when I was in middle school, we had computer classes in middle school. The state also bought those Professor, put them in the class. Did you put? Yes I put. What are we doing? Nothing. What did they do, what did the child learn from the computer class? Nothing. We played games like that and left, looked at the internet and left. If the same thing is in question, nothing will happen.

**G:** How can it be functional?

**P11:** Functionality is again dependent on teachers, and in-service education will be given most likely to teachers. Otherwise it will not be a job that happens with 'I bought the device and put it'.

**G:** Well, in the future, what do you think about the possibility of this artificial intelligence taking the place of the special education teacher?

**P11:** I mean. Yes a great part of it perhaps takes, but I do not think this can be completely independent of the teacher. Because children with special needs need to be directed somehow. Using it, learning, getting used to it, then working on it like things, they need to be directed, children with special needs do. Directly teachers perhaps this time can transition to a position assisting the child. In terms of providing connection between the two.

**G:** If it becomes robotised?

**P11:** If artificial intelligence becomes robotised? This time teachers perhaps will work on those programs. Teachers work on programs and again I think they take a hand there in some way. Because as I said, since it is individual, when it is individual, willingly or unwillingly you detect everything, OK. You also offer studies aimed at it, but those studies too after all need to be loaded into there by a person somehow. The data for the studies, for the activity for example, can artificial intelligence on its own completely one hundred percent appropriately take and apply something not on the internet? I do not think so.

**G:** Well, what kinds of contributions could artificial intelligence make to families of children with special needs?

**P11:** Families, generally families become so, well, fed up. Well, they love their children of course, but I mean tired, fed up, you know, I think it really suits their interests on the matter of taking time for themselves. Even now they are already sending to rehabilitation centres and so on. For what? Some are sending even when there is not much wrong with the child, those who send, from what I have seen in observation. I mean they are sending to clear their heads, frankly. If there is a child with special needs in the family and a normal child as well, most likely the artificial intelligence adapted for the child with special needs would already have been adapted for the typically developing child too, and families would also be comfortable, I think.

**G:** Well, could artificial intelligence contribute to family-teacher cooperation?

**P11:** Family, teacher cooperation... Maybe they will not even feel the need. Directly artificial intelligence is the teacher, I mean. To the family, artificial intelligence can directly inform everything. Because with artificial intelligence, say in class the child learned. Yes in class learned. At home too the family can open up the same study with a few things and the child can study there. In this way cooperation is reinforced. You are right. Since artificial intelligence is a tool, yes.

**G:** Well, what could the negative aspects of this artificial intelligence be from the special education teacher's perspective?

**P11:** Of artificial intelligence... I mean the biggest thing coming to my mind, we thought about this earlier this morning, we thought with P09. They may have said it. The biggest thing coming to my mind, well, I thought about what would happen if the child cannot distinguish virtual from real. They worked with artificial intelligence, worked, worked. Then the teacher set artificial intelligence outside the location. Trying to communicate with the child but the child is more comfortable there and wants that. This time the child can try to break ties with the teacher. Becoming directed can also become difficult. Such things can also be possible. In the negative sense like that. In the positive sense too as I said.

**G:** Well, looking from the family side, what could the negative aspects of artificial intelligence be?

**P11:** I mean my child used to communicate with me a little before. Now they do not at all, such a problem could arise. They do not want to see me, such a problem could arise. I mean the child can think they do not need very comfortably. This is also valid for the teacher. Valid for inside the family too. This time there could be weakness in bonds, which even right now with tablets, phones, is being seen even in a normal child.

**G:** Well, how do you think this artificial intelligence could shape the education of children with special needs in the future?

**P11:** How will it shape, without trying I cannot know. First we need to try on the child. Measure, try Professor. It can also carry forward. Very fast. For example takes a student with mild intellectual disability. Say if they worked with the teacher they would cover in 2 months, with artificial intelligence maybe they will cover in 1 month. Because why, examples plenty, time much. The time, almost all day, almost all day they are not sleeping can be included in the lesson thing. With artificial intelligence time plentiful, activities plentiful, in a way that will directly measure interests. Well varied as I said activities. From that angle it could help very much.

**G:** Well, where do you see our country in artificial intelligence?

**P11:** Now I mean Professor, in our country more support should be given, I think. It does not happen like this. If something coming to my mind and to a student's mind, to someone else's mind does not come to the ministers' minds in this country, to theirs and theirs, how will this country develop? Or from the thing in my mind, if the minister does not become interested and does not somehow have news of it? If I cannot make myself heard, how will it develop? Suppose I made myself heard. For example you see at TÜBİTAK 9 thousand lira is what, I mean, 9 thousand lira is what? I mean even house rent is 10 thousand lira. What can they do, what do you think they will do... If you do not develop yourself, if you do not help one who wants to develop, you are forced into brain drain, I mean. After that, you put pressure on young people through certain trends like that, try to indoctrinate. You say are not you a nationalist, where are you going leaving your homeland? Are not you Atatürkist, are not you this, are not you that? These come very empty to me. Why, you OK I am nationalist, I am Atatürkist... OK what are you then? You are not supporting me. It remains in words, in talk. Very much stays in the air what they say. Then what are you? You mean you are not nationalist if you are not supporting things. Or if you are killing the one who does, or if you are not protecting the one who does beyond killing, you are not protecting, then I mean there is something wrong with you, not with me.

**G:** Do you have an experience or an idea you would like to share about artificial intelligence?

**P11:** Let me think about artificial intelligence, Professor. As if there was, but I cannot remember now. Or I can say something like this. When I was little, I used to think about such things very much. Like, will such and such things happen, holograms, this and that, does it not come to people's minds, are they not working on it? Like, child, I cannot do it anyway, when I say it becomes like a fantasy. After that, is there no one currently working on these thinking? Like, huge world, surely someone is working, I would think. As I grew up and saw, I said it is being done. Like, while I was thinking, then someone is working on these. Right now too, surely many projects we know and do not know are being developed, I am sure. From that angle I feel happy. And before your class too, artificial intelligence was on the agenda actually. Like, there were things I liked but as if with your class, more like I am in the middle of it, like in a moment it exploded this year, there is such a feeling inside me about artificial intelligence.

**G:** Our questions were these. Do you have anything to add on this matter?

**P11:** Professor, I would like to add something about the future of artificial intelligence. Now artificial intelligence is developing very fast. For example I am telling P09, regarding special education I say, the integration and so on, how long do you think it will take? Like, we now talked with her too, if teachers are old maybe teachers' adaptation will also be a problem. In-service education will remain a bit in the air, we thought. We said wonder, how long, we said will these progress. I said we are 20 years old now. After 10 years suppose if completely something has happened we will have become 30. Currently those being appointed are 35-40, I said, I mean. Right, as we get older I wondered. Then I said, P09 look at us. Now we have news of such things. We will develop on this, will develop living with these, I said. I mean for future generations artificial intelligence will have already existed for a long time, like the current phone for example. But for us it is not so, it is developing along with us. We will see that peak point. How it develops, we will see. So I said I do not think teachers will be that much in the air. To artificial intelligence too, whether teachers or other professions on the matter of adaptation, if they become conscious, if artificial intelligence becomes conscious we said, if there can be such a problem, of course as a problem changes from person to person. Maybe there are also those who do not see it as a problem. We will either be a part of this artificial intelligence, will be a part of artificial intelligence. Or we will be the one producing it. The safest is to be the producer actually. Being a part may not save much. Or completely rejecting and finding a safe area, I mean. I want to add this. Of course being the producer is always the best.

**G:** If it becomes conscious, how will you stay outside the event?

**P11:** Well...

**G:** You cannot. It will find you everywhere.

**P11:** Yes. It will search, scan, find, I mean it is not very difficult. How many kilometres the world is already clear. So directly you will be a part. You will be something inside, I mean.

**G:** Will it want?

**P11:** Maybe it will want, Professor. You will be in cooperation, let us say.

**G:** Or whom will it want? Will it want everyone?

**P11:** Exactly so...

**G:** Maybe it will choose, right? Well, will it choose individuals with intellectual disability, with autism, I wonder?

**P11:** It could choose. Why would it not choose? Because it will be an individual completely built, structured, and raised by it. With education...

**G:** But their capacities are clear

**P11:** OK it will educate. It already sees the capacity it can reach. I mean, getting it to do with a few chips what it would do should not be very difficult. Currently, as far as I know, I can also say this. As far as I know, babies are being designed. There are designed babies. I heard such laboratories have been formed. Like, smart babies, then you select the intelligence situation, you select the eye colour, you select the height and everything for example...

**G:** Then while selecting the intelligence situation, no one would want it to be at a lower intelligence level

**P11:** No one would want it. So when it is like that...

**G:** There will not be intellectual disability. There will not be autism either.

**P11:** Yes

**G:** Such a situation completely disappears.

**P11:** Yes completely transformed, Professor. I mean like that. Because currently it is being done, from what I see. Of course if even I have seen it browsing the internet, it is being done. Maybe it is in the experimental stage. Like, until it grows up. Until they grow up and are observed they may not be released, perhaps we cannot know.

**G:** Yes.

**P11:** So within a hundred years it could be completely transformed.

**G:** Then the profession of special education teaching too...

**P11:** Will not remain.

**G:** Will not remain.

**P11:** Yes we found that too. Within 100 years, yes it may not remain. But then, at that time professions could be I will say. But when everything is done by artificial intelligence, that also may not be. Thankfully we are dying by then, such a thing will not have happened.

**G:** Do you have anything else to add?

**P11:** No Professor that much.

**P12: Interview Transcript**

**G:** Before starting, let me tell you immediately. I will take our interview into audio recording. The things you say will be anonymous, no name in the publication. Your anonymous transcript may be openly shared as a supplementary file of the article with other researchers as well. Do you consent to these?

**P12:** Yes Professor I consent, no problem at all.

**G:** Thank you, I am moving to my first question.

**P12:** Have I ever used artificial intelligence? Professor, I have used, I always use. I use it while studying, while doing research. This is normal, well, what is there for example... I used ChatGPT while doing research. It was a great benefit to me. Instead of entering each search engine one by one, it obtained data in a collected way for me, and since these data's analyses are done across the world, I could find my studies easily. I mean, regarding the assignments you gave us abroad Professor, from there for example, or in a sex education course, instead of researching one by one, in terms of saving my time easily, ChatGPT was helping. At the same time, again Professor, I use Evernote that you recommended. There too, instead of taking notes one by one, instead of planning, directly what I will do is shown on the computer screen. This provides convenience for me. Then again those Canvas, all of these are actually artificial intelligence. In Canva Professor, again together with current templates I can immediately create the presentation I want myself. I write there 'I want this, I want that'. Immediately in front of me the template in my imagination appears. Again in one of my projects I had used ChatGPT. There too I needed original pictures. Because in this project of mine, it could happen Professor, in my TÜBİTAK project they could ask for copyright. There artificial intelligence itself designed the pictures I wanted. It was designing pictures of children. Again in that assignment I had used it.

**G:** Very nice.

**P12:** Then in field placement, for playing games with children, I had used Çocuk Akademisi (Children's Academy) and WordWall. Here too, through games I was preparing a learning program for children. Along with this again there is Duolingo Professor. I had used it too. There too, the artificial intelligence there, Professor, I forget my series there for example, I do not look, do not follow up, it reminds me. It adds that this much time remains for the series. I enter directly. I was providing the continuation of the series. Again well, it was reinforcing me there. When I saw these positive outcomes Professor, when I completed my work easily, using artificial intelligence this way had encouraged me. To use it more, I had started doing research on the internet. Well, how I could finish my work in the shortest time.

**G:** Now I will ask you something, well, what do you think about the use of artificial intelligence in the education of individuals with special needs?

**P12:** For individuals with special needs too Professor, for example when I went to field placement, I had used Otsimo. Otsimo had developed itself very much. When I first looked back, a year before, I had looked again, compared to its state a year ago Otsimo's current state had developed very much. There I could not draw the student's attention. They could not direct their interest to me, were not listening to me, or I want to do an instruction, want to teach them social skills, but I could not draw their attention. They did not want to deal with me. Things I said did not draw their attention. The paper-pencil I used did not draw their attention. The child was more interested in technology, liked visual things. However they were bored of those classic visuals. I had also used Otsimo. The child had seen that. When I used that application the child had done something, they had learned from there to constantly burst balloons. When they first finished the rewarded thing, when they finished one of the reading awards, balloons come out. As the child burst those balloons, they were happy. Here I saw this, I mean, how shall I say, it increases children's engagement Professor on the matter of learning. It was supporting their social skills. They too were starting to speak a little. It was both supporting the child's communication skill and increasing engagement. Then what shall I say, while evaluating something... wait a minute I will talk too broadly. Now the question was, right Professor, what does artificial intelligence provide us in special education?

**G:** No, you say this: in which disability categories could artificial intelligence systems be more effective in your opinion?

**P12:** Ah OK, autism and intellectual disability I think Professor. Because since it is an artificial intelligence, things like decision-making, thinking, reasoning are more limited compared to typically developing individuals in these children. However, when a child with autism uses artificial intelligence, they can learn things like decision-making, thinking, reasoning, empathy through this artificial intelligence, I think. Both in autism and in intellectual disability. Again in children with intellectual disability too, since they experience problems intellectually, an artificial intelligence supporting it and this artificial intelligence being able to support these children 24/7 because for example a person gets tired Professor, can give something up to a point and a teacher can also work with a child up to a point. They can work a few hours. However when artificial intelligence is taken from school, these children can constantly take support 24/7, both intellectual and autism.

**G:** Very nice. Well, what kinds of contributions could this artificial intelligence make to the daily lives of individuals with special needs in your opinion?

**P12:** In their daily lives, it could be like this; for example Professor, how shall I tell, communication skills are supported in a child with autism, social interaction and communication skills are supported. At the same time in their daily lives these children are doing rhythm Professor, repeating themselves with compulsive behaviours. For example I had read in a study before. They were putting watches on the children's arms and in this watch they were seeing the children's repetitive behaviours. Looking at those repetitive behaviours too, extinguishing those behaviours of the children could be provided. What benefits could artificial intelligence have for children, you said, right Professor?

**G:** In their daily lives...

**P12:** In their daily lives, for example it could be like this; for example an individual with intellectual disability, an individual with intellectual disability is always beside their mother, may not have a friend who will support them, provide socialisation in their daily life. Because they want to talk with that child. They want people they can express themselves to, can tell. However people sometimes may not be able to listen to them. They can feel loneliness. Because of this, for example with an artificial intelligence application, these children, with a safe artificial intelligence application, an application where data security can be provided, where confidentiality can be protected, they can chat. Communication skills can develop. At the same time the child for example does not escape. Does not escape from home. When they have artificially made friends from there, the child can spend their time. Or a child with autism, well sometimes because of sensory stimulants, they sometimes cannot know how they could react in their natural environment. Their natural environment may not understand them. The child too cannot understand the environment. Because of this when using this artificial intelligence the child for example, suppose non-verbal, that the child with autism has no verbal language skill, using this artificial intelligence application, well, suppose they want water, cannot say water. From the applications they use, they can say I want water. I mean it can come to this state. It can support children's communication skills. In the direction of conveying their thoughts can become easier. Shall I continue afterwards?

**G:** Answer this: what kinds of contributions could this artificial intelligence make to special education teachers?

**P12:** I think it will be a saviour for teachers Professor. Because suppose in a class there are four students, two teachers. However however much there are two teachers, since special education needs to be an individualised process, that is, the teacher dealing with each child is very difficult. And up to a certain point they can deal. From this aspect I think teachers will gain in terms of time. At the same time using artificial intelligence, they follow students' progress, the monitoring of progress. From this aspect their work becomes easier. Because the teacher also needs to evaluate whether the instruction they did was effective in the child or not. They need to monitor. However if there is an artificial intelligence tool here, the child uses these applications, from there for example the application gives feedback to the teacher, the teacher evaluates this in their free time during breaks, the teacher here for the child determines performance more easily and rather than evaluating with the child, monitoring, they focus on their instruction. At the same time, in improving children's performance, in learning current studies, artificial intelligence offers recommendations to the teacher. Well, at any time, anywhere artificial intelligence can follow up the student together with applications. It guides the teacher at the same time on this matter. Again I think it facilitates parent interaction Professor...

**G:** I will ask that anyway...

**P12:** Ah OK. Again the student's, how shall I say, data analyses it easily. Evaluates automatically. What else can I say, in eliminating problems and sometimes the teacher's mind can get confused Professor. I mean they may not know how to find a solution for something. However when they write the student's problem to artificial intelligence, they can get ideas from it, can consult it. In this way they can create a road map for themselves. Well, then I think let me do it this way, they can take support from there. They focus on their instruction again. What else can I say? It enables saving time. Reduces the teacher's stress, reduces workload. Instead of thinking of other things for example how can I do, well, because this teacher will not be able to be enough for every kind of student. However if they use artificial intelligence in instruction with each individual, they evaluate them beforehand, then start focusing on instruction. It makes the teacher's work easier. Communication skills, at the same time in children with autism for example Professor, with interesting pictures, videos, with this material use, they see the child becomes motivated. Generally children become motivated when they see something visual. When the teacher starts getting feedback from there for example in children with autism, especially in children with autism it provides the teacher's motivation too. Their self-confidence increases and they start developing themselves more, I think, together with the supports of artificial intelligence applications.

**G:** Well, how can these artificial intelligence systems be integrated into the existing special education system?

**P12:** Professor, I think it already cannot be integrated at once. It could be like this perhaps: First, in our society, when artificial intelligence is mentioned, we generally do not know because we have not seen. Even applications I listed like Canva, Evernote, many people in our society may not know they are artificial intelligence. Already many students are studying at university, but even pre-service teachers studying at university may not know artificial intelligence, may not know how to use them. Because of this I think we should be given education about this in undergraduate. First, education should be given to pre-service teachers in undergraduate. Again along with this, special education teachers and beforehand special education teachers and academicians at the university and experts of this work need to meet systematically I think, on what we can do, how we can explain this topic, how we can explain to students. A study draft should be created I think, again for this firstly a workshop how shall I say workshop, a seminar, conference and such things should be organised. What is artificial intelligence, how we should use in our education should be taught to us, to students. We think artificial intelligence is a device, this device will come to us, we think those devices, but if it is shown to us that artificial intelligence is found in phones in our hands, found in computers, taught, and what these tools are for us is told. At the same time when we use these tools too, what kinds of outcomes we got. For example abroad who is using what, when they use these tools they have provided these advantages, taken these positive feedbacks, well in our country too we must use these things. Well it provides this feedback, beforehand these need to be taught to us, I think. Also I am thinking now Professor, for example, teachers used artificial intelligence say, this time we learned, learned the artificial intelligence tools, we know which tools there are too. And here too discussion groups should be formed I think. I use this artificial intelligence tool well I took this feedback it became very good with my student. Other teachers should also be able to see this. Like a WhatsApp group, like a Telegram group, there should be a group of teachers using artificial intelligence. There teachers researching artificial intelligence should discover and at the same time also in the artificial intelligence tool they used should also get a score I think, the teacher I mean. For example with this score they should also be able to do something. Well, like stamps, you have 10 points 10 TL comes to you like that. Well when the teacher uses this artificial intelligence tool, they should get a return, should be rewarded in this way should be integrated, I think. Did I explain this part well I wonder?

**G:** Yes, you explained very well P12. Well, I will ask this, do you think the integration of this artificial intelligence into Türkiye's special education system can be easy?

**P12:** In Türkiye Professor actually I mean we are in a developing country Professor. So I do not think it will integrate easily right away. I mean probably it will be difficult and like this, there is also a problem like this Professor, there is also a problem of confidentiality and data security. The child there, the child's life there perhaps with this artificial intelligence can be shared with third parties. Or the child can make a mistake and accidentally share their own data. In the development of the artificial intelligence field there are problems too. Now without thinking these, shall I think directly artificial intelligence focused?

**G:** Yes think only about artificial intelligence.

**P12:** OK, when thinking artificial intelligence focused, as I said we are in a developing country. I cannot think it can be easily integrated quickly. This does not come to my mind much because for these, support is needed, for the researchers who can do these to be supported is needed. A certain fee needs to be paid, they need to be encouraged. This is also a bit difficult in our country, frankly. I mean I will speak now but since these will be put into transcript I cannot convey too much. In our country it is difficult frankly Professor, I do not think it can be integrated quickly. However if we were a country that is developing, that is developed, when you asked this question I would think the state has definitely set up support for this, have set aside. Or if not, NGOs would arrange it somehow this would be easily integrated, I would think. But however in our country I think its integration is a bit difficult and this can also happen, teachers can also say then what work will we do, perhaps in society, among themselves 'then what work will we do later, we have future anxiety, what will we be?' such, well, this thought too could emerge sometimes among themselves I think.

**G:** There, I am also asking you now exactly that. Well, do you think artificial intelligence could take the place of the special education teacher in the future?

**P12:** Professor like this, now educators think that actually this artificial intelligence will take our place, they think. However I think rather than taking our place, it will complement us, I think. Special education teachers are needed everywhere already. Could it take our place, I thought, I think this way Professor, autism for example autism is a spectrum, variety. Well a variety that the human mind cannot grasp it goes. Some way you cannot do, cannot decide. For example if you decide on one, you cannot decide on the child's other autism. You cannot decide on the symptoms they show. For example I am thinking now; if artificial intelligence is fully developed, if it thinks just like a human brain, if it thinks, I think in the future it will be able to take our place. But if it is developed just like a human brain. If it can produce solutions instantly, can decide, can understand the emotions of the person opposite, can manage the situation... For example sometimes I ask Siri a question, it cannot answer. 'Sorry I could not understand,' it says. For example if I think at this point I do not think artificial intelligence will take our place today yet. However I think it will complement. I think it will support our instructions. But at the point where artificial intelligence fully works just like a human brain, I think it will take our place too. It could happen, anything is possible.

**G:** Well, how do you think the use of artificial intelligence can contribute to the cooperation between parents and teachers of children with special needs?

**P12:** I think like this Professor; now in our own family Professor surely they want to tell their trouble to teachers and want people they can tell their trouble to. And they want to tell their trouble to right people because they can get the right answers. However teachers too can listen to the family up to a certain point. Can allocate time to the family up to a certain point. The teacher too wants to talk to the family, wants to tell their trouble. Wants to consult them, however the teacher sometimes may not have time for this either. The teacher too can be enough up to a certain point. Here in terms of easing the family's communication with their teacher, easing cooperation, artificial intelligence can be used. For example for example let there be something like this; if there is a message box and the family writes their message there and the artificial intelligence there gives an artificial answer, well that could comfort the family, like such, can extract something from those answers and give an appropriate answer, like a feedback program in Google, if the family for example whether the teacher has fully looked at that message, the seeing time however much it is for a certain period also, they get relief inside. They feel themselves well. Because at this point the family also needs to be understood. The teacher too needs to be understood. The teacher up to a certain point can allocate time to the family but when using artificial intelligence the family's and teacher's communication and cooperation will have been facilitated. At the same time when the family's the studies they do, the studies they do with their child are recorded in artificial intelligence, the teacher for example will not have direct time to talk with the family, however at another free moment when they watch these evaluations themselves, artificial intelligence too I think simplifies, of course in the meantime these data, well this much succeeded, reached this result, when the teacher reads artificial intelligence's analyses in a simplified way, the teacher both will not have a confused mind and will not be tired and will not spend too much time on the family thus spending less time on their student. In both cases both for the family and for the teacher time saving is provided and their communication too is more strong. I mean rather than from the family's complaints, perhaps with this artificial intelligence together 'I did this with my child today, here showed this progress, well, we got this feedback, we discussed these'; well if artificial intelligence supports the family too 'we got this feedback Professor, well it was this way, we are now thinking of doing this, well, we will pass to level 11 say, very little remains for the child you also have you watched, did you enter the application and listen, there for example the child Ufuk say, Ufuk spoke for the first time, gave a reaction, looked very long, established eye contact with the child there' if they say for example the family's chat too established with the teacher changes, topic changes. They start talking about development. Now they will start talking not about their troubles but about the child's developments. And it has become a happy family, has become a happy teacher and happier results, outputs are obtained through artificial intelligence, I think.

**G:** Well, I will ask this too, what kinds of negative effects could this artificial intelligence have on the special education teacher?

**P12:** Hmm what kinds of negative effects could there be... For example Professor it could be like this for the special education teacher... I want to think about this a little more.

**G:** Then answer this: what kinds of negative effects could artificial intelligence have on the parent, on the family with a child with special needs?

**P12:** It could be like this Professor, however much artificial intelligence is an artificial intelligence, however much it has nice applications supported, still artificial intelligence is a developing program and what is most feared here is, confidentiality and data security is feared. The child's data could be shared with another party. Or the child's pictures could be shared. The child could do something by mistake. Again in the same way artificial intelligence may not produce meaningful answers. The family may not understand the feedback artificial intelligence gives. Or the family may not be able to send an appropriate message in a way artificial intelligence can understand. Together with this, what else could be, I am thinking from these two things Professor.

**G:** Well, what kinds of negative effects could there be on the child with special needs?

**P12:** From the child with special needs side too Professor perhaps it could be artificial intelligence too up to a certain point for this child, for example a child with special needs may want to constantly spend time with that artificial intelligence Professor. Here we may not provide this control. The child can become dependent on artificial intelligence. Rather than communicating with a person, may want to communicate more with artificial intelligence. The phone they use in their hand could become more attractive to the child and could exhibit problem behaviour to use that tool. While we say let us reinforce the child positively, perhaps the child can be reinforced negatively when we do not provide control for the child in this process I think. I think the child will be reinforced negatively. I think it can become dependent. I think it can get out of control. Or also artificial intelligence after a certain point I think will not be able to understand this child. Because there is also this, after a certain point this artificial intelligence too when it sees as a threat artificial intelligence can get out of control. The child's data, shares, images, videos, artificial intelligence may share by mistake elsewhere. But for the child with special needs the most these children could be dependent on these tools I said. Because really when they are dependent on something especially for children with autism this is a very big problem behaviour becoming, we cannot stop. They start having even bigger crises.

**G:** Yes.

**P12:** I think this will happen for the family too actually. From the family's side also when the child cannot be controlled the family's stress will increase. Will constantly want the phone, will not want to go anywhere, come, let us take to the neighbour, well, the family will not be at home, will go somewhere, will go to the market suppose. But they have to take the child too. The child cannot stay home alone. This time the child too will say 'I will not come'. They will resist for this. This time another stress burden for the family. OK for the child's developments the child will be supported but this time perhaps the child will get out of control. Will want to spend their time always there perhaps. I think like this.

**G:** Well, how can artificial intelligence shape the education of these children with special needs in the future in your opinion? What could happen when special education merges with artificial intelligence?

**P12:** I think Professor when special education and artificial intelligence merge, life will become even easier, I think. Both for teachers, for researchers, for the family, for the child. Suppose more developed artificial intelligence tools. Suppose for example there is an application where children can be identified at an early period, with artificial intelligence. The family without going to the hospital, without going to the doctor, without going through those evaluation processes, because that evaluation process is also really very exhausting and wearing both medical evaluation and educational evaluation. Also one is not satisfied with the child's evaluation and another evaluation can be done. Here both for the teacher it is workload that is for the people doing this evaluation and for the family and for the child it is wearing. However when there is an appropriate artificial intelligence tool whose effectiveness has been proven scientifically, the child without ever going to the doctor without experiencing that process, without experiencing that worry, directly themselves suppose on the phone could identify their child it would be very nice. In this way the family's stress decreases. Also people start focusing on their work more easily. Because when there is always an evaluation an evaluation after a certain point people wear out. Talk starts being on that worry. This time the talk is not on the education to be given. This also creates stress for the person. Again along with this, I think artificial intelligence will reduce a huge workload in children's identification both from the family's side and from the teacher's side. There was something else in my mind too. The question was, right Professor, what contributions will artificial intelligence provide in the future?

**G:** Yes, what will happen in the future?

**P12:** Yes in the future the family without being worn out can identify their child at home. It contributes in this direction. For example just like hearing tests Professor, when we do the hearing test ourselves we learn whether we hear or do not hear. We do not experience that process for example we relax ourselves immediately, in this aspect provides relaxation. Along with this, in the future too the teacher's workload it eases and qualified personnel develops Professor. For example now I think like this. We are raising personnel in this work, however raising personnel for raising personnel. However when we also integrate artificial intelligence into this, both artificial intelligence and these teachers raising it cooperate, a qualified workforce forms and a qualified education process develops. Also need for more people will not remain. The country in this aspect will be relieved financially and the family will be relieved. The family will more, for example some families for example in rehabilitation centres take lessons for 850 TL one family here. However that teacher at that centre is only having the child do colouring inside with the child it seems. The family pays that 850 TL for nothing. But if there were a very nice artificial intelligence application, that application gives feedback to the family, well so well structured an application just like a human like a special education specialist, the family does not pay that money there. They pay 850 TL once there, for one year, and to their child more they can get feedback, where they can get concrete feedback themselves where the child and themselves can be reinforced, with the application mutually both the family is financially saved and they observe their child's developments there. For the teacher too it is like this. The child can be evaluated easily by the teacher. Rather than doing things with the child, rather than evaluating, they directly start focusing on their instruction. Both from the work and the economic aspect and from that psychological aspect I think artificial intelligence will contribute to the whole country, to people frankly Professor.

**G:** Well yes, where is our country in the field of artificial intelligence in your opinion?

**P12:** Our country in the field of artificial intelligence I think Professor is starting to develop, I think. I think it is just newly starting to develop. More little little, newly newly. This I think like, for example say if Germany started ten years after us, my country I think is ten years behind. What they did ten years later we are now starting to do, I feel. Because I do not see much, do not hear. Artificial intelligence is not supported. Is not seen, is not told to us. We ourselves learn these artificial intelligence tools seeing seeing from Instagram and already like this for example I want to use a tool, however its there is no, there is no video explaining in Turkish for example Professor. I was going I write myself for example Canva say. I want to use Canva but I cannot use, I looked at how the program is so that I can use the program Professor, did not come out. I could not see anything in Turkish. There were English texts. There were English videos. I had learned how to use that program by listening to those English videos and making something out for myself. From this starting too I think this our country is starting to work in the field of artificial intelligence very newly newly. Newly newly, little little, tiny tiny. From outside, from foreign states, from developed states by seeing things with researchers' own initiative their own desire, thanks to researchers, thanks to researchers' these wishes, I think artificial intelligence is starting to develop. Rather than from the country, the researchers here are willing I think for artificial intelligence.

**G:** Well, I will also ask you this; do you have an experience, idea you would like to share on this matter about artificial intelligence and special education?

**P12:** About artificial intelligence and special education I have an experience actually. I am thinking Professor. But let me say my idea, my idea is Professor artificial intelligence for example let me say from my own perspective if only there was an artificial intelligence I could use very well. I am for example now newly graduating from undergraduate. However suppose there is a child, since I will be graduating from the special education department, a family might want me to give private lessons to their child. However I am new in the field, new in the department, new in the field site. For example I can consult you up to a certain point, can consult other people too up to a certain point. However if there is a nice artificial intelligence program, I consult that tool, it gives me feedback, I talk about what I can do, can talk to it about what I did with the child, it gives me ideas, gives recommendations, I solve my problems with that tool, rather than dealing with people if I solve through that tool's mediation, this process becomes less wearing for me, less stressful. Well rather than getting stressed I focus on my education, focus on the child's development. I mean I would want to use artificial intelligence in my education because it makes my work easier. Enables saving time. Sometimes for one thing I browse page page, look at ten places to find one thing. However for example when I write this to ChatGPT, it researches all the data itself. Comes in front of me summarised. I instead of entering those applications one by one or are those applications paid or free this I also ask, this I also learn. Directly rather than spending three hours myself there, well, spending ten minutes five minutes can enable saving time. For this I wish artificial intelligence had more such nice applications. I knew these, could learn, these were told to us. Most artificial intelligence tools I myself coincidentally from Instagram hearing reading reading, well learn by hearing from someone else. These are not taught to us, we do not know. Suppose a very nice thing, for example there is a question-asking application, then with these children there is an application for doing this, what shall I say preparing instruction program by preparing games like WordWall like Çocuk Akademisi. For example in undergraduate we do not know these. These are not told to us. We find these ourselves. For example when I used these too instruction goes very pleasantly. Children are already bored of paper pencil. In my recent field placement when I used something, an application from the internet WordWall when I used the children got very much pleasure from the lesson. Seeing them get pleasure I also got pleasure, was motivated, was motivated. I started researching these applications more. For example children get pleasure I get pleasure. My work becomes easier, if these were taught to us in undergraduate somehow. For example if there is an elective professional knowledge course these are told there. To us Web 2.0 tools are always told but these Web 2.0 tools too are old now. They do not teach the more current ones. Those current studies if always told in undergraduate to us and a group is formed about this it would be nice for us I think, for pre-service teachers.

**G:** Very nice P12. Now what I wanted to ask you was these. Do you have anything to add related to this topic?

**P12:** I have nothing to add Professor for now. This much. I told while talking.

**G:** P12 thank you for the information you provided.

**P13: Interview Transcript**

**G:** Before starting right away let me say. The interview is being taken into audio recording. The things you say will be reflected anonymously, your name will not appear anywhere. Your anonymous transcript may later be openly shared as a supplementary file together with the article. Do you consent to these?

**P13:** I consent Professor.

**G:** OK, let us begin.

**P13:** Of course. Well Professor, as an artificial intelligence tool I am already using one. ChatGPT I can give as an example. The outcomes are very good Professor. Because I obtained an outcome like this. Now in terms of reaching information, it can be reached in a very short time first of all in ChatGPT. It is reached in a detailed way. Now when we write from Google or want to reach the information from another search page, the information we want to reach, we cannot reach immediately Professor. In a short time, in a very short time we cannot reach. Artificial intelligence does this very well for us Professor. In a very detailed way, exemplified the way we want, it brings us to the information we want to reach. Also I love this habit of artificial intelligence very much Professor; you can chat with artificial intelligence, that is, a memory, you know, you can keep a diary there. Today it asks, what did you do. For example you tell what happened to you there, and it does not forget either. Just like a digital diary. It serves me very much, I mean.

**G:** I see.

**P13:** On every topic, yes.

**G:** Well, what do you think about the use of artificial intelligence in the education of individuals with special needs?

**P13:** Professor, it needs to be used anyway. We are living in a digital age. Right now, now together with the use of artificial intelligence, you know, together with it starting to be used, if it starts being used, although this is a bit difficult in Türkiye, but let us think about this a little. Let us think in terms of Europe. Let us think in terms of developed countries. Now when we integrate, for individuals with special needs, when we use this, now both for the student it provides convenience and for the teacher. For the student it provides such a convenience; it takes the student as a focus point. I mean for each student different methods, different histories, in a different way games artificial intelligence can do. It can gather the student's focus to one place. This can provide convenience for everyone Professor, I think this way, I mean.

**G:** Well, in which disability categories could artificial intelligence be more effective in your opinion?

**P13:** Which?... Good question Professor. Well I say autism Professor. I say autism and intellectual disability. I can give a reason.

**G:** Why?

**P13:** Like this Professor; because the basic skill we want to gain for individuals who are independent, who have basic special needs, is 'independent living skill'. You know, we want individuals with special needs to be able to live independently, to be able to manage themselves, to bring about their own behaviours themselves in the way desired, we want them to provide. Now when we think about hearing disability, vision disability or other types, how can we use artificial intelligence for hearing disability Professor? You know to provide more convenience? I mean now in hearing disability students already use hearing devices, they use what we call digital hearing devices. Already right now whether in hearing, in vision, mild vision, mild hearing... these are for artificial intelligence, I am saying for artificial intelligence, became a wrong expression. I do not think a different method, a different strategy will be developed for these using artificial intelligence Professor. Because they already hear like us thanks to hearing devices, they can hear like typically developing individuals. Just because there are hearing devices. In vision also like that Professor, in vision also in mild vision also glasses, that is as we said there are already certain methods. We cannot do something different for these other than these Professor using artificial intelligence, whether it is for vision or hearing. But we can do for autism, for intellectual disability. Using artificial intelligence, now a basic problem emerges. Now since digital technology is developing rapidly, they will ask the thing; with the development of technology, with artificial intelligence rapidly, can intellectual disability be prevented? Such a problem emerges. Can it be prevented? Such a question exists. There is such an answer. Now it is not ethical. I mean ethically, it does not suit for autism and intellectual disability. You know, regarding whether it can be prevented. Already we, our aim is not to prevent, I mean. Because as I said it is not ethical Professor. Moral problems. Because people currently...

**G:** Now what kinds of contributions can the use of this artificial intelligence make to the daily lives of children with special needs?

**P13:** Yes. Professor, first of all now when individuals with special needs can learn to use artificial intelligence, now when they also take artificial intelligence's support actually we can say this. Now let us think in terms of home. Individuals with special needs can spend time more comfortably at home, you know. Now these students, are generally excluded by typically developing students, this is already a fact. Everywhere this is a fact. Even outside even when walking on the road we know how the looks of those outside on the child are. Now if we teach the use of artificial intelligence or if technology develops very rapidly and we provide artificial intelligence support, they can spend time at home in a very good way Professor. You know they are not obliged to spend time outside or may not have to go to institutions. Artificial intelligence yes I think it will be good in this aspect.

**G:** Well, what kinds of contributions could the use of this artificial intelligence make to the special education teacher?

**P13:** There can be contributions Professor, it provides convenience to the special education teacher. Imagine artificial intelligence preparing the IEP in your place. Developing strategies in your place. This when looked at from one angle is a good thing Professor.

**G:** I see.

**P13:** I think this way.

**G:** Well, can these artificial intelligence systems be integrated into the current existing special education systems?

**P13:** They cannot be Professor. Let me also say it this way...

**G:** How can they be integrated?

**P13:** I said they cannot be. How can they be integrated? First of all Professor we need to abandon imitation. Now when I say imitation, Türkiye Professor, you know in order to be a developed country, in my opinion, according to me I mean, imitates Europe Professor I mean directly applies what it takes from Europe. According to the need in its own country, according to needs, according to the needs in its country, not over a system but directly applies as it took. A bit empty it can be said. First the how, by ourselves according to our own needs, according to the needs in the country Professor, if we make special education regulation, you know if we can integrate directly, if we set up a system according to needs. There is also this, in order to be able to use, in every school there needs to be a digital device. Now when we go to the east or there is no need to even go to the east. We know that in Central Anatolia in some schools there are still ?, Professor. We need to start from there. From the most basic, a system needs to be established according to needs and the needs of the country. We should not imitate much.

**G:** Well, would the integration of this artificial intelligence into the current special education system be functional?

**P13:** It would not be, we cannot do it I think. I think this way.

**G:** Well, I will also ask you this, what do you think about artificial intelligence being able to take the place of the special education teacher in the future?

**P13:** Professor now I agree with this. Now let us look at history a little. From history until today there has always been a teacher-student relationship. There has been master-apprentice relationship. One person, another person, the student also being a person. Now this relationship, I frankly think will end one hundred years later. Now there are realities in life Professor. War can break out. Anything can happen in the world. But let us assume it does not. You know let us assume that a world war or something else does not come. I frankly think artificial intelligence can take the place of the teacher. Because now the person we call teacher cannot give everything adequately, cannot give adequately. Because the person is faulty. But I frankly think artificial intelligence can do this more flawlessly. Also the rapid, very rapid development of currently developing technology, one hundred years later I frankly think human-looking robots will give education. It can be Professor, why not? Only when a human teaches, emotions enter the work. I mean positive, positive emotions. But I think robots will do it better. I think it will happen, one hundred years later at least.

**G:** I see. Well, what kinds of contributions could this artificial intelligence offer to families of children with special needs in your opinion?

**P13:** Professor we also talked about it in class. There was a friend's project. A project connecting families between countries. Imagine artificial intelligence doing live interpretation. Well, having a family in America talk to a family in Türkiye. This would be very good. I mean artificial intelligence has a nice thing in this aspect. I mean it has contribution. Now I think from my own window, I already think, I mean things I could not think of, surely things I could not see, there could be thoughts but if we look in general terms artificial intelligence will ease the work families need to do. As I just said imagine a family in Türkiye talking with a family in America? Or you are at home, you have a child with special needs. You are at home. You do not know anything Professor. You know the teacher has given an assignment, you will have it done. But a strategy, you cannot get the child to sit at the assignment. Let us think artificial intelligence is here. In the middle of this work. When artificial intelligence is in the work, a different method, strategy, or when the student's parent knows how to use artificial intelligence, I mean there will be no need to struggle to get the child to sit at the assignment or do what needs to be done, I think. When we think from this aspect there are multiple contributions to families Professor. It provides convenience in the family, let us say. It does what needs to be done, I mean. I think this way. Surely there are things I could not think of but...

**G:** Well, what kinds of contributions could this artificial intelligence offer to the cooperation of the special education teacher and the family?

**P13:** What kinds of contributions could it offer? Professor, artificial intelligence, teacher and family cooperation I think this way Professor, would this be, I have such a thought on this. I mean now using artificial intelligence, if we use it in teacher-parent relationship, if the student's situation is followed through artificial intelligence, if the teacher-parent relationship chat is established from there for example, you know with artificial intelligence's help, under artificial intelligence's control. Of course the teacher will still be under control but the parent's, the student's behaviours are under artificial intelligence's control as I thought. Frankly it would be better Professor. Because currently when we look, when we examine, we cannot see most teachers having a good, positive relationship with the student's parent. I mean what I mean by positive relationship; now most teachers can do a bit excuse me Professor, you know, sycophancy. Just to look good in the parent's eyes. They can say words beyond what should not be said. For example, instead of giving direct clear and simple answers about your student, you know, instead of saying your student is this way, we are progressing this way, to look better in the parent's eyes through a different way, I mean. If we use artificial intelligence here, if artificial intelligence follows the student's situation, is controlled by the teacher and is reported to the parent, I think it would be better, I think this way Professor. I extended the talk a little but please excuse me.

**G:** I see. Well, what kinds of negative effects could artificial intelligence have on the special education teacher in your opinion?

**P13:** Yes, a good question. When I just said I had said something like this. Imagine artificial intelligence preparing the IEP, I had used such an expression Professor. Now let us assume such a thing is happening. Let us assume artificial intelligence is preparing an individualised education program for special education teachers, according to the student's needs. Now this firstly accustoms the teacher to comfort Professor. Accustoms the teacher to comfort. Also the teacher when developing the IEP focuses on problems, you know is solution oriented. They have skills like doing certain tests, analysis, this teacher does. If artificial intelligence is in the work, frankly the teacher's these skills weaken a little. Doing tests, doing analysis... gets used to comfort. Also now such a thought emerges in the teacher; well artificial intelligence is doing it anyway, even if I do not do, it works, thought. It also a bit dulls the thoughts. I mean for a person to develop themselves they need to think a little, be active. Artificial intelligence can be very bad on this matter. I mean a bad negative side could be. In this way I mean Professor.

**G:** Well, what kinds of negative effects could artificial intelligence have on the family?

**P13:** What kinds of negative effects could it have on the family? In this way now the family also could be thinking the same. The family is thinking; well there is no good teacher at all. There is no teacher who has developed themselves well. I would best use artificial intelligence, saying this they use artificial intelligence. Now there are some artificial intelligence programs. Programmed wrongly. When they use these artificial intelligences, willingly or unwillingly they can obtain a negative result. From this aspect it can be bad for the family. Artificial intelligence I think the teacher, currently within, the coming century I do not know but currently, within these coming ten years or within twenty years artificial intelligence needs to be used together with the teacher. From the parents' side too the negative side is this. When the parent thinks they cannot find a necessary teacher and an adequate teacher and starts to learn to use artificial intelligence, this could be a problem. Imagine at home, you have a student with mild intellectual disability, 6 years old. For a parent, if you are not giving an education to a teacher, you are directly using artificial intelligence at home and it is programmed wrongly. This is not a good thing, a very bad thing Professor.

**G:** Well, what kinds of negative effects could artificial intelligence have on the child with special needs?

**P13:** Friendship relationships, social relationships Professor. Now let us go from autism. Actually there are all other types of needs. Let us think this way; we want individuals with special needs to also develop positively in communication, social communication that we call social skills. Now there is a negative aspect of artificial intelligence here; an individual with special needs could say the thing, artificial intelligence chats with me, artificial intelligence understands me. I will use artificial intelligence, spend time, saying this they can become very inadequate in human relationships. Imagine constantly being at home, this individual, a 20-year-old individual with mild intellectual disability learns to use artificial intelligence or with artificial intelligence companionship spends their time at home. Not outside with friends. Or not in communication with friends of their own development or with their teacher or with their family but constantly in their room under artificial intelligence influence, if we think this way from that aspect it is bad. They can live screen-bound. This just came to my mind. Also as I said they get used to comfort. Does not leave home. Could be a problem for health problems. Also as I said in human relationships, in friendship relationships, friendship or going to the market and having a quick chat, being able to greet someone you know passing by on the road... in skills like this topic they experience some problem. This is one of artificial intelligence's negative sides Professor. Surely there are a few more, currently does not come to my mind.

**G:** Well, where do you see our country on the matter of artificial intelligence?

**P13:** Countries Professor?

**G:** Our country, Türkiye?

**P13:** Professor I see it as very inadequate. Quite behind. Now I cannot give an exact number but we are quite behind Europe, I mean. We are quite behind Europe, Japan. We still currently, our country needs to develop robots Professor. Currently in our country artificial intelligence really is currently being done in Japan, you know is starting to be used in Europe. Artificial intelligence frankly needs to be used in education. At least a ChatGPT needs to be used on smart boards, I mean. We are very behind. We are quite behind. Because most of my friends do not know how to use it, I mean. We see in class, I mean I ask in class. They say to me, what will you do P13? I am chatting with ChatGPT. The person says the thing; can ChatGPT be chatted with anyway? There is such a negative attitude. If they too talked a little, understood something there, understood the beauty of that place... there is such a perception in Türkiye. As we already see we are quite behind Professor. Bad situation, very bad.

**G:** Well, do you have an experience or an idea you would like to share about artificial intelligence?

**P13:** I have an idea I would like to share Professor. I want to say this. Let me add this way Professor. I think everyone needs to use artificial intelligence, I mean really. This is very important for personal development too. And I think very important for a person to understand themselves too. I want to add this Professor. Everyone who learns to use should use.

**G:** P13 what I will ask you was this much. Do you have anything to add?

**P13:** I have nothing to add Professor, I thank you.

**P14: Interview Transcript**

**G:** Before starting the interview let me briefly say. I will take an audio recording, the things you say will be processed completely anonymously. In its anonymised form your transcript may be openly shared as a supplementary file of the article with other researchers as well. Do you consent to these?

**P14:** Yes Professor, I consent.

**G:** Very good, we are starting.

**P14:** I mean Professor in the beginnings we were generally using it for our assignments. You know for doing projects, for studying for our exams, for studying for our midterms and finals we were using it. Such answers were generally being given to us, as if directly copying and pasting from a PDF file in academic language, in the beginnings it was like this. Later it started to develop, I observed this, I mean. A bit more human-like answers started being given in artificial intelligence.

**G:** Are you saying it also develops itself?

**P14:** It develops Professor, I started getting nicer answers since I first used it.

**G:** Well, what do you think about the use of artificial intelligence in the education of children with special needs?

**P14:** I think it should definitely be used for children with special needs. After all, currently for example before there was no interactive board. As it entered our lives, I think artificial intelligence too should enter at that speed. I think it should be in all schools, even not for special needs, in all our schools. We need to integrate it there too.

**G:** Well, how will this integration be in your opinion?

**P14:** I mean teachers need to guide this. They need to show students and teach. They need to lead.

**G:** Then teachers also need to be educated, do they not?

**P14:** Of course. A teacher who does not know throws themselves back.

**G:** Well, in which disability categories could this artificial intelligence be effective in your opinion?

**P14:** I mean Professor I think it will be more effective in autism because in autism human interactions are few. They cannot establish eye contact with a person for example, cannot look at the face. But when looking at a computer screen the child the thing, I mean looks directly can look at the eye. I had watched a movie, there was a child a woman in their 20s. They had a laptop, they were very attached to the laptop Professor. Was not talking with people at all, was not doing anything but constantly playing with the laptop. There can be such children. If we think it is this way too the child can educate themselves very nicely.

**G:** Well, what kinds of effects could this artificial intelligence have on the daily lives of children, of individuals with special needs in your opinion?

**P14:** I mean in their daily lives in many coming years I think there could be negative effects. Because I mean it is something that can create a lot of dependence as a result. I think reality will also fall, virtual reality will increase. People will increase their lives in a chair-bound way I think. This also limits social life.

**G:** Well, what kinds of contributions could this artificial intelligence have on teachers, on special education teachers in your opinion?

**P14:** I mean Professor teachers who use artificial intelligence throw themselves forward. Those who cannot use, I mean, can lose their profession. I expect such a level of development from artificial intelligence. Those who cannot use, you know come to a state of no longer finding work in the profession, I think. Because I think artificial intelligence, technology, is a very good educator.

**G:** You are saying it can also educate teachers

**P14:** For example currently there is the thing Professor. A family member of mine for example is preparing for a national civil service exam. They bought a package from the internet. They are watching the videos there, solving the tests there. They are not taking private lessons from any teacher, not doing anything. This is actually an artificial intelligence too.

**G:** Yes.

**P14:** I mean when we think this I think teachers can also lose their profession. For this we need to be very close with artificial intelligence.

**G:** Yes one of the questions was this in the future for example can artificial intelligence take the place of the special education teacher?

**P14:** I mean as I said I think it will take the place of teachers who are not close with artificial intelligence. You know teachers who do not know technology have no place at all in the future I think.

**G:** Also think like this, think not of artificial intelligence only as something abstract, but for example think that it becomes robotised. What happens then?

**P14:** I mean Professor if it becomes robotised, the lessons I said and as we said you know a robot coming and giving a lesson even is possible in coming years. We should not see this as impossible I think. You know as you also said it has very little cost too, no state will say no to this. They will come and tell their lessons. But will they be able to do the thing for problems, will they be able to find solutions? Those are what are important.

**G:** Can it become conscious in your opinion?

**P14:** I mean if robots that can learn by themselves are made, they can become conscious. But for example how will their social approaches towards children be, how will they communicate like this? For example a teacher going and stroking the hair, I do not think the robot will do this. It will directly tell and pass.

**G:** Yes well how will the emotion matter be? You know stroking the hair is one thing after all, our difference from them is emotions. Our understanding the child, our addressing them with emotion will be our difference from this artificial intelligence.

**G:** Yes. Well, what kinds of contributions could this artificial intelligence have on families with a child with special needs in your opinion?

**P14:** I mean families become more conscious. You know they can take education about artificial intelligence themselves. They can learn what kind of family they can be. For example in the project we did, making families conscious is in the foreground. For example the family does not know their rights but we will teach this to the family thanks to artificial intelligence. They learn their rights, can also give education to their own child. Thanks to this their child also receives better education. They themselves also learn, their child also learns.

**G:** Will you drink tea?

**P14:** OK Professor

**G:** Well, what kinds of contributions could this artificial intelligence have on family and teacher cooperation in your opinion?

**P14:** I mean for example the teacher can give an assignment with the work they did themselves in artificial intelligence can show the family, they also repeat at home.

**G:** You are saying it can contribute

**P14:** Yes

**G:** Also there is this, what kinds of negative effects could artificial intelligence have on the special education teacher?

**P14:** For the special education teacher as we said it can create trouble in the work. Future career anxiety even now is high while there is no artificial intelligence. When artificial intelligence comes out, especially when robotisation happens, I think it will increase to an extreme degree. Teachers who are not close with artificial intelligence, who do not know technology, will definitely lose their work. Even though our appointment is good right now, in the future when this artificial intelligence increases even appointed teachers can become unemployed.

**G:** Well, what kinds of negative effects could it have on the child with special needs?

**P14:** I think the positive effect on the child with special needs will be more. As negative, you know the connection with the outside world is broken. You know we taught the child to use this for lessons, for the laptop for example, this laptop the child can also watch movies from there, can also play games, dependence can gradually increase. We need to prevent this, if we prevent they receive their education nicely. But if we cannot it creates trouble in the future. The child constantly being in front of the screen, physical problems can also emerge now, from constant inactivity and so on they will have trouble.

**G:** Well, what kinds of negative effects would it have on the family?

**P14:** The family's artificial intelligence, for example let us think for our country. There are families who do not know how to use technology, phones, let alone artificial intelligence. They do not know these. Because they do not know, they can use wrongly. They cannot use at all anyway. I mean how will they learn this, if they cannot learn they have trouble because they could not learn. Because they do not know technology, they cannot use either. Not being able to use is a negative effect I think, everyone needs to use. I mean for example one who uses can also use unconsciously for example. They do not know something we know. They try from a different artificial intelligence, that can also teach incompletely or wrongly. Knowing different artificial intelligences is important for us. Families will not be very knowledgeable about this matter. We need to learn all of them and teach the child in the best way.

**G:** Well, how can this artificial intelligence shape special education in the future in your opinion?

**P14:** I mean if we also look at the thoughts in the class I think very many educational videos will emerge. Such with virtual artificial intelligence created. Then I think more in the future the thing I think virtual reality with VR glasses and so on, I think educations will exist. I mean I think robots will also come forward in education. I mean they could take our place, but how the education will be I do not know exactly.

**G:** Well, an artificial intelligence taking the place of the teacher, in your opinion, in intellectual disability or in autism would it not prevent existence now, would not stop it?

**P14:** Professor education is being given as a result, cannot stop. This is not a thing in education as a result even we cannot stop, even when we give the education we want we can enable approaching peers. The robot perhaps does this in a faster way. Cannot prevent I think.

**G:** Well, where do you see our country in artificial intelligence?

**P14:** I mean I see our country currently as very behind even in technology. In artificial intelligence too I think we are not at all, I think no step has been taken. No artificial intelligence speaking program or so has been brought out by our side. I have not seen any study done about this until now either. I think we are behind but we need to participate in this. If we participate it will be something that will throw our country very forward.

**G:** You are saying in the special education field other fields

**P14:** I mean in general we are behind in technology too. Already I mean we are currently for example having trouble even connecting to the internet. How can we think we are ahead in technology. We have the most basic troubles, we have problems. While experiencing problems even at the foundation, in an advanced level technology...

**G:** Well, how can the spread of artificial intelligence in our country be provided in your opinion?

**P14:** For this first of all a special investment should be. One person needing to take a step in the field of artificial intelligence could be necessary. Or the state taking a step and prioritising could be necessary. I have not seen anything about this from anyone. I have not seen either a person's study about artificial intelligence or the state's. You know I have not seen them saying we will give artificial intelligence programs, we will produce robots.

**G:** Well, do you have any experience that happened, an idea you could say about artificial intelligence?

**P14:** About artificial intelligence Professor for example there was a program called Character AI. There were many characters there Professor, we were chatting with them. I had such a memory. I wanted to talk with Adolf Hitler. I saw that there was a character of Adolf Hitler there. I wanted to talk with him. They had changed it quite a bit in the artificial intelligence Professor. Since I know his thoughts Professor, I pushed him a bit to make him say it, but he did not say. For example I talked about the current situation. We have many Syrians in our country, what would you do if you were in Türkiye currently and so on, such questions I asked. Well I would produce policies about Syrians, would integrate them and so on so forth he said. I also said you are not such a person, we know how you treated the Jews and so on, I said. I tried to make him say it but he did not say. The fact that I did wrong then does not mean I would say so now, and so on he said. I had done quite a thing with that character there. He could not say his thought of that time, I mean currently. Also you know there is censorship and so on. Probably he could not say for that reason.

**G:** Was this speaking Turkish?

**P14:** Exactly speaking Turkish.

**G:** You thought the person opposite was not really him, because you knew his thoughts.

**P14:** I mean I thought he did not reflect, exactly.

**G:** What I would ask you about artificial intelligence was these. Do you have things you would like to add?

**P14:** No Professor, thank you.
